# “Vitamin D and Human Health: from the Gamete to the Grave”: Report on a meeting held at Queen Mary University of London, 23rd–25th April 2014

**DOI:** 10.3390/nu6072759

**Published:** 2014-07-22

**Authors:** Adrian Martineau, David Jolliffe

**Affiliations:** Centre for Primary Care and Public Health, Queen Mary University of London, Yvonne Carter Building, 58 Turner Street, London E1 2AB, UK; E-Mail: d.a.jolliffe@qmul.ac.uk

## 1. Preface

The inaugural Vitamin D and Human Health conference was held on the London Whitechapel campus of Queen Mary University’s Barts and The London Medical School, from the 23rd to 25th of April, 2014. This three-day meeting set out to achieve two main aims: to create a forum for researchers to meet and forge new collaborations, and to provide a state-of-the-art overview of the latest findings from clinical research in the field of vitamin D. Over 300 clinical researchers, students and commercial representatives attended. Thirty international experts in the field of clinical vitamin D research presented talks organised into a programme spanning the human life course. Commencing with a session of talks providing overviews of randomised trials of supplementation and global vitamin D status, the meeting proceeded with a session on pre-birth related vitamin D research—evolution, genetics & fertility—which led into several talks in the area of child health. Sessions on respiratory health, immune function, cancer biology, and neurodegenerative diseases preceded an overview of research in the area of ageing-related health outcomes, including musculoskeletal health and metabolic diseases. Finally sessions on the economy of vitamin D and public health, along with future directions for research were held. Several themes emerged during the course of the meeting. The anticipation of results from very large (*n* > 5000) randomised controlled trials of vitamin D supplementation (“mega-trials”) and Individual Patient Data (IPD) meta-analyses were hot topics of discussion. Mega-trials have the potential to detect small effect sizes of vitamin D supplementation on end-points such as incidence and mortality from cardiovascular disease and cancer. IPD meta-analyses have the potential to investigate the causes of heterogeneity often seen in the results of individual primary trials by allowing clinically important subgroup effects of vitamin D supplementation to be elucidated. The existence of a U-shaped relationship between vitamin D status and risk of certain health outcomes was another area of discussion. A third emerging theme, also relating to vitamin D dose–response relationships, was the potential differential effect of daily *vs.* intermittent bolus dosing on biological outcomes. Finally, the meeting also addressed strategies to tackle vitamin D deficiency at the population level, by alteration of sun-seeking behaviour, use of nutritional supplements and food fortification. The following 156 abstracts featured in the meeting as either a poster or an oral presentation.

## 2. Summary of Scientific Presentations

### 2.1. Sex-Specific Associations between 25-Hydroxy Vitamin D_3_ and Serum Lipids in Elderly German Subjects without Intake of Lipid-Modifying Drugs

#### Jungert, A.; Neuhäuser-Berthold, M.

Background: Although emerging evidence indicates an association between vitamin D and serum lipids, the data are still inconsistent. The aim of this study was to investigate whether 25-hydroxy vitamin D_3_ [25(OH)D_3_] is independently related to serum lipids in elderly women and men, who were participants in the longitudinal study on nutrition and health status in senior citizens from Giessen, Germany (GISELA study).

Methods: Fasting 25(OH)D_3_ serum levels were assessed along with serum lipids [triacylglycerols (TAG), total cholesterol (TC), high-density lipoprotein cholesterol (HDL) and low-density lipoprotein cholesterol (LDL)], body composition and lifestyle factors in a cross-sectional study of 145 well-functioning German elderly (104 females and 41 males; age: 66–96 years; BMI: 27 ± 4 kg/m^2^). Stepwise multiple regression analyses were performed to examine associations between 25(OH)D_3_ and serum lipids including age, percentage total body fat, smoking, physical activity, sun exposure and intake of alcohol and saturated fatty acids as covariates. Effect modification by sex was evaluated by stratified analysis and tests for statistical interaction by adding a product term (sex × 25(OH)D_3_) to the multiple regression model.

Results: Median (25th–75th percentiles) vitamin D status was 64(52–72) nmol/L. Dyslipidaemia, defined as TC ≥ 240 mg/dL, TAG ≥ 200 mg/dL, LDL ≥ 160 mg/dL and/or HDL < 40 mg/dL (men) and <50 mg/dL (women), respectively, was found in 38% of the participants. Subjects with dyslipidaemia had significantly lower 25(OH)D_3_ levels than subjects whose serum lipids were in the reference range (median: 60 nmol/L *vs.* 65 nmol/L; *p* < 0.05). Substantial sex differences existed regarding the associations between 25(OH)D_3_ and serum lipids, and effect modification by sex was significant with respect to log HDL, LDL/HDL and TC/HDL (*p* < 0.05). After adjusting for age, percentage total body fat, smoking, physical activity, sun exposure and intake of alcohol and saturated fatty acids, 25(OH)D_3_ was an independent predictor of LDL/HDL (β = −0.241; *p* = 0.014) and TC/HDL (β = −0.250; *p* = 0.010) in women, whereas men showed no independent associations.

Conclusions: Sex-specific associations of 25(OH)D_3_ with serum lipids may exist in elderly subjects, and an adequate vitamin D status may have a beneficial impact on dyslipidaemia in elderly women.

### 2.2. Associations between Vitamin D and Biomarkers of Oxidative Stress in Community-Dwelling Elderly suBjects: A Cross-Sectional Study

#### Jungert, A.; Neuhäuser-Berthold, M.

Background: Some studies suggest that vitamin D may have anti-oxidant and anti-inflammatory properties (Asemi, Z., et al. *J. Nutr.* 2013, *143*, 1432-1438. Vanoirbeek, E., et al. *Best Pract. Res. Clin. Endocrinol. Metab.* 2011, *25*, 593-604). Advanced ageing is associated with an increased level of oxidative stress, which is characterised by a greater production of reactive oxygen species without an appropriate rise in anti-oxidant defence mechanisms (Wang, C.H., et al. *Exp. Biol. Med. (Maywood)* 2013, *238*, 450-60). The present study examined the associations between 25-hydroxy vitamin D [25(OH)D] and biomarkers of oxidative stress in community-dwelling elderly subjects and whether body composition and smoking behaviour modulate the assumed associations.

Methods: Cross-sectional data of 228 women aged 62 to 92 years who were participants in the GISELA study (longitudinal study on nutrition and health status in senior citizens from Giessen, Germany) were analysed. Concentrations of 25(OH)D, total anti-oxidative capacity (TAC) and activities of glutathione peroxidase (GPx), catalase and superoxide dismutase (SOD) were assessed in fasting blood samples. We performed simple and stepwise multiple regression analyses to examine the influence of 25(OH)D on TAC, GPx, catalase and SOD with adjustments for age, relative fat mass, physical activity and smoking. TAC and GPx underwent lg10 transformation to achieve normal distribution.

Results: Median levels of 25(OH)D, TAC, GPx, catalase and SOD were 55 nmol/L, 1.5 mmol/L, 26 U/g haemoglobin (Hb), 659 kU/g Hb and 3100 U/g Hb, respectively. Subjects with a 25(OH)D status ≥75 nmol/L had higher levels of SOD (median: 3137 *vs.* 3085 U/g Hb; *p* = 0.002) and lower levels of catalase (median: 637 *vs.* 666 kU/g Hb; *p* = 0.016) than subjects with lower 25(OH)D levels, whereas TAC and GPx were not significantly different. In simple regression analyses, 25(OH)D was significantly associated with SOD (β = 0.161; *p* = 0.015), but no association was found with TAC (β = 0.045; *p* = 0.497), GPx (β = 0.113; *p* = 0.090) or catalase (β = −0.124; *p* = 0.063). After multiple adjustments, 25(OH)D was a significant independent predictor of SOD (β = 0.159; *p* = 0.027), but exhibited no influence on TAC, GPx or catalase. When stratifying the cohort according to smoking status and BMI, the association between 25(OH)D and SOD was present only in never smokers (*n* = 155; β = 0.167; *p* = 0.048) and in subjects with a BMI < 30 kg/m^2^ (*n* = 181; β = 0.197; *p* = 0.015), whereas current/ex-smokers and obese individuals showed no association. Furthermore, 25(OH)D was inversely associated with catalase only in current/ex-smokers (*n* = 59; β = −0.294; *p* = 0.034).

Conclusions: The present investigation indicates that the vitamin D status may function as an independent positive predictor of SOD, especially in non-obese, non-smoking elderly women. More research is required on the underlying mechanism and potential health consequences.

### 2.3. Vitamin D and All-Cause Mortality in Australia: The Melbourne Collaborative Cohort Study

#### Heath, A.K.; Williamson, E.J.; Kvaskoff, D.; Baglietto, L.; Hodge, A.M.; Ebeling, P.R.; Giles, G.G.; Eyles, D.W.; English, D.R.

Background: Evidence from epidemiological studies suggesting that circulating 25-hydroxy vitamin D (25OHD) levels are inversely associated with mortality is largely based on studies conducted in Europe and the USA. It is unclear whether the relationship is similar in regions with higher UVB irradiance. We investigated whether 25OHD was associated with reduced risk of all-cause mortality in Melbourne, Australia.

Methods: This was a case-cohort study nested within the Melbourne Collaborative Cohort Study (MCCS). The MCCS recruited 41,514 people aged 40–69 years between 1990 and 1994. Eligibility for this study was restricted to MCCS participants with dried blood spots from baseline blood samples and without a pre-baseline cancer diagnosis. Deaths were identified by linking to the National Death Index. We included all deaths that occurred between baseline and 31 December 2007 (*n* = 2403) and selected a sex-stratified random sample of participants (*n* = 2996). Concentrations of 25OHD in dried blood spots were measured using liquid chromatography-tandem mass spectrometry (LC-MS/MS). Cox regression, with the Barlow weighting scheme to account for the case-cohort design, was used to estimate hazard ratios (HRs) for all-cause mortality for each quintile of 25OHD with adjustment for age, sex, country of birth, education, socioeconomic status, physical activity, waist circumference, smoking, alcohol, parity, dietary patterns, energy intake, diabetes at baseline, and prior heart attack, stroke, angina or hypertension. The reliability of the LC-MS/MS assay was assessed using replicate measurements from 500 case-cohort participants. A separate calibration study was undertaken, including 62 MCCS participants who had both dried blood spots and plasma stored from baseline, in order to calibrate measurements from dried blood spots against plasma.

Results: There was good agreement between measurements of 25OHD from dried blood spots and plasma (*r*-squared = 0.73). The LC-MS/MS assay showed high reliability; the intra-class correlation from 500 replicate measurements was 0.78 (95% CI: 0.75, 0.82). In the case-cohort study the mean follow-up was 13.7 years. The HRs for all-cause mortality by quintile of 25OHD were (from lowest to highest) 1.41 (95% CI: 1.12, 1.78), 1.27 (95% CI: 1.01, 1.60), 1.30 (95% CI: 1.04, 1.61) and 1.10 (0.88, 1.38) relative to the highest quintile (*p* for trend = 0.002).

Conclusions: We found an inverse association between 25OHD and mortality. These findings from our Australian cohort were similar to those from cohort studies conducted in Europe and North America.

### 2.4. Quantification of Serum Vitamin D_3_, 25-Hydroxy D_3_ and 24,25-Dihydroxy D_3_ in Human Serum by LC-M/MS

#### Burild, A.; Frandsen, H.L.; Jakobsen, J.

Background: Serum 25-hydroxy vitamin D (25(OH)D) is the established biomarker of vitamin D status. Assessment of serum 25(OH)D is routinely used in the clinic (Higashi, T., et al. *J. Chromatogr. B* 2010, *878*, 1654-1661. Jones, G. *Scandinavian J.Clin. Lab. Invest.* 2012, *72*, 7-13.), as well as in intervention studies and epidemiological studies (Heaney, R.P., et al. *Am. J. Clin. Nutr.* 2008, *87*, 1738). Large inter-individual variation in serum 25(OH)D response to oral supplementation is however observed in populations given the same dose (Vieth, R. *Am. J. Clin. Nutr.* 1999, *69*, 842-856). CYP2R1 and CYP24A1 are capable of the 25-hydroxylation and 24-hydroxylation, respectively, of vitamin D. It is reported that different single nucleotide polymorphisms of CYP2R1 and CYP24A1 are associated with the 25(OH)D status (Wang, T.J., et al. *The Lancet* 2010, *376*, 180-188). Therefore we hypothesized that the ratio between serum vitamin D_3_, 25(OH)D_3_ and 24,25-dihydroxy D_3_ (24,25(OH)_2_D_3_) may be of interest to understand the kinetics of serum 25(OH)D_3_. No methods are to our knowledge capable for the simultaneous quantification of serum vitamin D_3_, 25(OH)D_3_ and 24,25(OH)_2_D_3_.

Method: We developed and validated an LC-MS/MS methods for the simultaneous quantification of serum vitamin D_3_, 25(OH)D_3_ and 24,15(OH)_2_D_3_. 100 µL of serum was subjected to HybridSPE to selectively remove phospholipids. The analytes were derivatized with 4-phenyl-1,2,4-triazoline-3,5-dione to enhance the ionization of the analytes in the mass spectrometer equipped with an electrospray ion source.

Results: The method was validated for human serum and the limit of quantification was <0.2 ng/mL for vitamin D_3_, 25(OH)D_3_ and 24,15(OH)_2_D_3_. Precision was <6.5% for vitamin D_3_ and 25(OH)D_3_ and <10% for 24,25(OH)_2_D_3_.

Conclusion: A more holistic approach to the vitamin D_3_ status and metabolism could be enabled by including assessment of serum vitamin D_3_ and serum 24,25(OH)_2_D_3_. The described method has the potential to bring new insight to the kinetics of serum 25(OH)D_3_ in human and animal sciences. We are currently using the method for the quantification of serum vitamin D_3_, 25(OH)D_3_ and 24,25(OH)_2_D_3_ in human interventions studies as well as animals studies.

### 2.5. Increased Distal Radial Trabecular Volumetric Bone Mineral Density with Higher Vitamin D Status in UK Dwelling Postmenopausal South Asian Women

#### Darling, A.L.; Hakim, O.A.; Berry, J.L.; Lanham-New, S.A.; Hart, K.H.

Background: Previous research has found an association between vitamin D status (25-hydroxy vitamin D, 25(OH)D) and volumetric Bone Mineral Density (vBMD) in Caucasians (Pedone, C., et al. *Bone* 2010, *46*, 1063-1067. Sayers, A., et al. *Osteoporos. Int.* 2012, *23*, 2117–2128.). However, there has been little research assessing this relationship in South Asians, with no data in older South Asian women. Therefore, the aim of this work was to assess whether serum 25(OH)D is associated with bone geometry in postmenopausal South Asian and Caucasian women.

Methods: In summer 2010, 18 South Asian and 48 Caucasian women (aged 58 to 75 years) had pQCT scans (Stratec X2000L) undertaken of the radius (4% and 66% sites) and tibia (4%, 14% and 38% sites). Fasting blood samples were obtained for assessment of serum 25(OH)D.

Results: Partial correlations assessed the relationship between 25(OH)D and bone geometry, adjusting for body mass index (BMI). At the 4% (distal) radius site, in Caucasians, there was a positive correlation between 25(OH)D status and bone mineral content (BMC) (*r* = 0.404, *p* = 0.008), total area (*r* = 0.327, *p* = 0.035), and trabecular area (*r* = 0.327, *p* = 0.034). For Asians, there was a significant positive relationship between 25(OH)D concentration and trabecular vBMD (*r* = 0.547, *p* = 0.035). In Asians there was no significant correlation between vitamin D status and any tibial bone parameter (*p* > 0.05). However, at the 38% site in Caucasians, there were significant correlations between 25(OH)D concentration and bone mass (*r* = 0.304, *p* = 0.050). There were also significant positive associations between 25(OH)D and cortical area at the 14% site (*r* = 0.353, *p* = 0.022) and between 25(OH)D and trabecular area at the 4% site (*r* = 0.336, *p* = 0.029).

Discussion: Overall, in Caucasians vitamin D status appears to be positively correlated with radial and tibial bone mass and size. In South Asians, vitamin D status appears to be positively correlated with distal radial trabecular density. Vitamin D was not correlated with tibial bone parameters in the Asians, but was associated with some tibial mass and area parameters in Caucasians. Further analysis is underway to assess possible explanations for these varying relationships between vitamin D status and bone geometry by ethnicity and bone site.

### 2.6. Vitamin D Status and Sleep Quality in UK dwelling South Asian and Caucasian Women

#### Darling, A.L.; Hart, K.H.; Middleton, B.; Lanham-New, S.A.; Skene, D.J.

Background: Both vitamin D deficiency and insomnia are prevalent clinical problems in Western societies. Despite the fact that vitamin D deficiency increases musculoskeletal pain (Heidari, B., et al. *Int. J. Rheum. Dis*. 2010, *13*, 340-346) and that musculoskeletal pain has a deleterious effect on sleep quality (Molina, J., et al. *Clinics (Sao Paulo)*. 2012, *67*, 1139-1144), no published research has specifically examined whether there is a link between vitamin D and sleep. Therefore, in this study, associations between sleep quality and vitamin D status in UK dwelling South Asian (A) and Caucasian (C) women were investigated.

Methods: Actiwatches are a validated method for assessing sleep wake cycles (Marino, M. et al. *Sleep* 2013, *36*, 1747-1755), using a watch like device to estimate the degree of wakefulness based on the amount of movement of the person’s arm. In September to October 2010, Actiwatches were worn and a daily sleep diary completed by *n* = 47 women aged 39–75 years (*n* = 27 C; *n* = 20 A), over a 14-day period. After exclusion of subjects who did not have valid Actiwatch data for ≥7 days, *n* = 42 participants remained (*n* = 27 C; *n* = 15 A). Participants completed a sleep questionnaire (the Pittsburgh Sleep Quality Index; PSQI *n* = 27 C; *n* = 13 A), and gave fasted blood samples for HPLC measurement of 25-hydroxy vitamin D (25(OH)D) (*n* = 27 C; *n* = 14 A).

Results: In terms of objective actigraphic measures of sleep characteristics, there were no significant associations between sleep-wake cycles and 25(OH)D in the Caucasians (*n* = 27, *p* > 0.05). However, in the Asians there was a significant negative association between 25(OH)D and sleep latency (time taken to fall asleep) (*r* = −0.562, *p* = 0.036, *n* = 14). For the sleep diary data, partial correlations showed no significant associations between 25(OH)D and the self-reported time “try to sleep”, sleep duration or wake time in either ethnic group (*p* > 0.05; *n* = 27 C; *n* = 14 A). Last, for the PSQI data, Spearman’s Rho correlations indicated in Caucasians that increased 25(OH)D concentration was associated with increased overall PSQI score (*r* = 0.385, *p* = 0.047, *n* = 27), as well as increased score for the sleep latency sub-scale (*r* = 0.439, *p* = 0.022, *n* = 27). This suggests poorer overall self-reported sleep quality and longer self-reported sleep latency with higher 25(OH)D concentration in Caucasians. In Asians, there were no significant associations between 25(OH)D and PSQI scores.

Conclusion: This research provides preliminary evidence that vitamin D might be associated with sleep quality. Although there is some suggestion that self-reported sleep quality and sleep latency might be associated with vitamin D status, results appear to vary by ethnicity. This study is limited by the small sample size, particularly in the Asian group and so it will be important to replicate this work in a larger sample.

### 2.7. Vitamin D, Vitamin D Receptor Expression, Vitamin D-Related Gene Variants and Colorectal Cancer Progression: A Systematic Review

#### Kunzmann, A.T.; Cantwell, M.M.; Murray, L.J.; Ng, K.; Carroll, D.; Coleman, H.G.

Background: Recent experimental, ecological and epidemiological evidence links vitamin D with an anti-carcinogenic effect. However, the relationship between vitamin D related measures and survival amongst colorectal cancer patients remains unclear. The objective of this systematic review was to assess the association between vitamin D related markers and colorectal cancer prognosis.

Method: Using terms for vitamin D and colorectal cancer, the Medline, Embase and Web of Science databases were systematically searched for studies published, in any language, until March 2013. Random effects meta-analyses were used to calculate pooled Hazard Ratios (HR) and 95% confidence intervals (CI) for the association between vitamin D measures and colorectal cancer-specific survival and overall survival.

Results: A total of 10 studies met the inclusion criteria. Higher vitamin D status (per 25 nmol/L increase in serum/plasma 25(OH)D) was associated with improved colorectal cancer specific survival (adjusted HR = 0.92 (95% CI: 0.86–0.98, *p* < 0.001) and overall survival (pooled adjusted HR = 0.87 (95% CI: 0.79–0.95, *p* < 0.001) in pooled estimates of 5 prospective studies. However, there was a large degree of heterogeneity in the results for overall survival but not for colorectal cancer specific survival. High vitamin D receptor expression in tumour cells relative to normal cells was associated with improved disease-free survival in one prospective study. No significant associations were observed between total vitamin D intake and disease-free survival (*n* = 1 prospective study), between supplemental vitamin D intake and colorectal cancer mortality (*n* = 2 randomised controlled trials, where colorectal cancer mortality was a secondary outcome), or between Vitamin D related polymorphisms (Bsml and Fokl) and colorectal cancer specific survival (*n* = 1 prospective study).

Conclusions: Epidemiological evidence supports an association between increased vitamin D status and improved survival amongst colorectal cancer patients. Further studies assessing the effect of vitamin D supplementation, and related molecular and genetic factors, on colorectal cancer survival are warranted.

### 2.8. 3-epi-25 Hydroxy Vitamin D in Pregnancy

#### Bennett, S.E.; Casey, C.; McPeake, J.; McCance, D.R.; Manderson, J.G.; McGinty, A.

Background: Vitamin D deficiency has been reported during non-diabetic and Type 1 diabetes mellitus (T1DM) pregnancy. Both non-diabetic and T1DM neonatal (cord) 25OHD levels are significantly reduced in women classified as obese *vs.* normal weight pre-pregnancy. In T1DM women, HbA1c at booking is significantly negatively correlated with maternal 25OHD, suggesting a potential role for this vitamin in maintaining glycaemic control (1). 25OHD_2/3_ and 3-epi-25OHD_2/3_ share an identical structure (and molecular weight) and differ only in the stereochemistry of the hydroxyl group at the C-3 position. The physiological importance of 3-epi-25OHD_2/3_ is uncertain and there have been limited studies determining the levels of these epimers in human populations. The aims of the current study were to, (1) determine 3-epi-25OHD_2/3_ levels throughout non-diabetic and T1DM pregnancy; (2) to examine the relationships between 25OHD and 3-epi-25OHD; (3) to assess the impact of maternal BMI on 3-epi-25OHD and examine associations with markers of glycaemic control.

Methods: This was an observational study of 52 pregnant controls without diabetes and 65 pregnant women with T1DM in a university teaching hospital. Maternal serum 25OHD and 3-epi-25OHD were measured serially throughout pregnancy and post-delivery. 25OHD and 3-epi-25OHD were measured in cord blood obtained at delivery. 25OHD and 3-epi-25OHD were measured by liquid chromatography tandem mass spectrometry (LC-MS/MS).

Results: 3-epi-25OHD was found in 90.2% of control (median 0.9 nmol/L; range 0.1–5.9 nmol/L), and in 94.5% of T1DM, women (median 1.4 nmol/L; range 0.1–10.5 nmol/L). In the control group, 3-epi-25OHD at trimesters 1 and 2 significantly positively correlated with 25OHD at all 3 trimesters (*p* < 0.009). 3-epi-25OHD at trimester 3 significantly positively correlated with trimester 3 25OHD (*p* < 0.001) and cord blood 25OHD (*p* = 0.015). Cord 3-epi-25OHD also significantly positively correlated with 25OHD at all 3 trimesters (*p* < 0.031) and with cord 25OHD (*p* < 0.001).In the T1DM group, 3-epi-25OHD at trimester 1 significantly positively correlated with 25OHD at trimesters 1 and 2 (*p* < 0.003). 3-epi-25OHD at trimesters 2 and 3 significantly positively correlated with 25OHD at all 3 trimesters (*p* < 0.05) and cord 25OHD (*p* < 0.002). Cord 3-epi-25OHD also significantly positively correlated with 25OHD at all 3 trimesters (*p* < 0.024) and with cord 25OHD (*p* < 0.001).Seasonal variation in maternal 3-epi-25OHD levels was evident, Summer levels were significantly higher than all other seasons in the control group (*p* < 0.001) and significantly higher than Spring (*p* = 0.003) and Winter (*p* < 0.001) in the T1DM group. The T1DM group had higher 3-epi-25OHD levels in Autumn *vs*. Winter (*p* = 0.013). When compared within season, levels of 3-epi-25OHD were significantly higher in the T1DM *vs.* control group in both Spring (*p* = 0.045) and Autumn (*p* = 0.022). Increased maternal BMI significantly reduced cord 3-epi-25OHD (BMI < 25 kg/m^2^ *vs.* >30 kg/m^2^, *p* = 0.04). HbA1c was significantly negatively correlated with 3-epi-25OHD at trimesters 1 and 2 (*p* = 0.049; *p* = 0.001) and with cord 3-epi-25OHD (*p* = 0.012).

Conclusion: Maternal 3-epi-25OHD exhibits seasonal variation and, in common with cord 3-epi-25OHD, correlates with 25OHD throughout both non-diabetic and T1DM pregnancy. In T1DM women 3-epi-25OHD is associated with a key marker of glycaemic control.

### 2.9. The Impact of Seasonal Vitamin D Deficiency and Vitamin D Supplementation on the HIV-1 Immune Response

#### Coussens, A.K.; Naude, C.; Goliath, R.; Chaplin, G.; Jablonski, N.; Wilkinson, R.J.

Background: Cape Town, South Africa, at latitude 33 degrees south has a highly seasonal pattern of UVB exposure, peaking in summer. Consequently, as UVB penetration of the skin is required for vitamin D production, citizens are at risk of vitamin D deficiency in winter. This risk is enhanced in persons with dark skin pigmentation as melanin absorbs UVB. Vitamin D has been shown to inhibit HIV-1 replication in macrophages, *in vitro*, while seasonal vitamin D variation in Europeans has been associated with decreased lymphocyte counts and enhanced pro-inflammatory responses in winter. We therefore aimed to determine whether seasonal vitamin D deficiency occurs in two healthy young adult populations with moderate or dark skin pigmentation in Cape Town, whether this had an effect on the number and function of circulating blood cells, their ability to control to HIV-1 infection and whether vitamin D supplementation modifies the investigated responses.

Methods: HIV-1 uninfected healthy young (18–25 years) adults, either darkly pigmented and of Xhosa ancestry (*n* = 50) or moderately-pigmented and of mixed ancestry (Cape Coloured, *n* = 50) were recruited for follow-up in summer and in winter, 6 weeks following solstice, from two nearby areas of Cape Town. At winter follow-up, participants of Xhosa ancestry (*n* = 33) received 6 weekly doses of 50,000 IU cholecalciferol and were followed-up 1 week after the last dose (*n* = 30). At each visit, blood was taken for serum 25(OH)D and vitamin D binding protein quantification, full blood count, whole-blood immunophenotyping by flow cytometry and *in vitro* infection with HIV-1, followed by functional flow cytometry.

Results: Serum 25(OH)D was significantly lower in both populations in winter compared to summer (median 25(OH)D: Xhosa, 45.3 nM *vs.* 72.5 nM, *p* < 0.0001; Cape Coloured, 43.7 nM *vs.* 65.4 nM, *p* < 0.0001). Notwithstanding similar median levels between populations (*p* > 0.73), Cape Coloured participants had significantly greater levels of deficiency (<50 nM) in summer, 16% *vs.* 4% in Xhosa participants (*p* = 0.01), with neither showing severe deficiency (<30 nM). Conversely, in winter, Xhosa participants had a trend for greater levels of severe deficiency 18% *vs.* 12% in Cape Coloured, with the later still having a trend for greater deficiency overall (70% *vs.* 64%, *p* = 0.13). Winter deficiency correlated with significantly lower circulating red blood cells (RBC) and RBC distribution width (RDW), in both populations. Vitamin D supplementation increased total white blood cell count, lymphocyte count, neutrophil count, RBC and RDW.

Conclusions: The majority of healthy young adults with moderate or dark skin pigmentation in Cape Town become vitamin D deficient in winter. Vitamin D supplementation increases circulating cell numbers and it will be determined whether supplementation affects the *ex vivo* ability of blood cells to control HIV-1 replication.

### 2.10. Cord Blood Vitamin D Levels and the Subsequent Risk of Lower Respiratory Tract Infections in Early Childhood Results of the Ulm Birth Cohort

#### Łuczyńska, A.; Logan, C.; Nieters, A.; Elgizouli, M.; Schöttker, B.; Brenner, H.; Rothenbacher, D.

Background: Lower respiratory tract infections (LRTI), primarily pneumonia and bronchiolitis, are a leading cause of morbidity and mortality in early childhood. Infancy in particular is a time of increased infection susceptibility and severity. Current evidence suggests that immunomodulatory properties of vitamin D may influence LRTI risk. The objective of this study was to examine whether 25-hydroxy vitamin D [25(OH)D] concentrations in cord blood influence susceptibility to LRTI in the first year of life.

Methods: We analyzed data from a prospective birth cohort of 777 mother-infant dyads based in Ulm, Germany. The samples were measured by OCTEIA 25-hydroxy vitamin D enzyme immunoassay (IDS, Immunodiagnostic systems Ltd., Boldon, UK). Relative risks (RRs) of LRTI in relation to 25(OH)D cord blood levels were estimated by log-binomial regression after adjustment for potential confounders (gender, siblings under 5 years of age, premature delivery and breastfeeding duration). To account for seasonal variation in vitamin D levels, as well as seasonality of infections, we examined the association within different season strata. Analyses were conducted using predefined-cutpoints, quartiles, and season-standardized 25(OH)D quartiles.

Results: Mothers were predominantly born in Germany (82.6%), educated for 10 years or more (79.2%), and non-smokers at six weeks after giving birth (88.2%). 51% of infants were boys and 49% girls. We observed a statistically significant association between 25(OH)D status in cord blood and the risk of LRTI across the year when using predefined cutpoints. The adjusted RR of LRTI for individuals with vitamin D deficiency (25(OH)D < 25 nmol/L) in comparison to the referent category (>50 nmol/L) was 1.32 (95% confidence interval (CI) 1.00,1.73). Stratification by season of blood draw revealed an inverse association between 25(OH)D and LRTI for individuals whose blood was collected in the fall months. The adjusted RR was 1.68 (95% CI 0.98, 2.86) using a priori defined cutpoints, 1.91 (95% CI 1.10, 3.32) for the lowest vs highest vitamin D quartiles and 1.59 (95% CI 0.95, 2.67) in the lowest quartile in the season standardized model. Upon stratification by maternal allergy status, a more pronounced association between 25(OH)D and LRTI across the year was observed in infants born to mothers with no reported allergy RR = 1.45 (95% CI 1.03, 2.03). The effect was largely driven by a strong association in infants born in fall 3.07 (95% CI 1.37, 6.87). A similar pattern of results was observed when the data was examined by quartile cutpoints RR = 3.51 (95% CI 1.55, 7.95) and season standardized cutpoints RR = 2.01 (95% CI 1.02, 3.95).

Conclusions: Our findings suggest that vitamin D deficiency at birth is associated with increased risk of LRTI particularly in infants born to mothers without allergy. The association appears to be strongest in infants born in fall. Strategies aimed at improving maternal vitamin D status during pregnancy, especially in the winter months, are needed and might decrease the risk of LRTI in early childhood.

### 2.11. Vitamin D Significantly Changes the Immune Transcriptome and Augments CD4 Recall Responses in HIV

#### Lachmann, R.; Bevan, M.A.; Kim, S.; Patel, N.; Banga, R.; Chandra, N.; Narayan, Rao. S.; Hawrylowicz, C.; Vyakarnam, A.; Peters, B.

Background: *In vitro* studies, including our laboratory, highlight the active metabolite of Vitamin D, Calcitriol, to have pleiotropic effects on innate and adaptive immunity. More recently, *in vivo* Vitamin D supplementation showed beneficial effects by promoting resolution of inflammation. HIV progression, which is associated with excessive immune activation and impaired T-cell immunity, is also associated with low serum vitamin D level although biological plausibility and causality have yet to be established. This study was therefore designed to assess the impact of Vitamin D supplementation on T-cell function in both HAART treated and treatment naïve HIV+ subjects.

Methods: This prospective controlled study enrolled 28 subjects with low plasma vitamin D (Vitamin D) (<20 ng/L, 50 micromol/L) comprising 17 HIV+ patients (11 on HAART, 6 treatment naive) and 11 healthy controls. A single dose of 200,000 IU oral cholecalciferol was administered. Blood samples were analysed at baseline and one month. Advanced multi-colour flow cytometry was used to assess T-cell signalling, T cell effector responses, and markers of T-cell activation and homeostasis. In addition the whole blood transcriptome was analysed along with the primary Vitamin D receptor, VDR.

Results: Plasma Vitamin D levels were restored to normal at one month. The following statistically significant results were found: CD4 T-cell responses to CMV and SEB were markedly augmented in HAART+ subjects and similarly HIV-specific responses in HAART Naïve subjects. Specifically MIP1β+ CD4 T-cell frequency increased in HAART+ subjects, concomittant with an increase in plasma MIP1β+, which correlated with plasma Vit D levels. An associated increase in promimal T-cell pERK mobilisation following PMA stimulation was noted. T-cell CD38 expression was downregulated only in HAART+ subjects; however no significant changes to T-cell CD39 or Treg and IL-17 numbers were noted. These specific changes to the T cell compartment were associated with changes to some 250 genes at the whole blood level in HAART naive and healthy controls. However, 10-fold fewer genes were altered in HAART+ subjects associated with significantly lower basal VDR expression. Pathways impacting T-cell function were altered in all three groups but a common gene signature spanning all three groups was not identified.

Conclusions: Vit D therapy may be a useful adjunct to HAART therapy in HIV infection by improving the quality of the T-cell response. The potential therapeutic benefit of this cheap, safe, easily administered therapy on slowing progression and reducing mortality in HIV merits testing in adequately powered clinical trials.

### 2.12. Symptoms in Swedish Female Primary Health Care Patients with Vitamin D Deficiency

#### Björk, A.; Andersson, Å.; Johansson, G.

Background: Possible symptoms of vitamin D deficiency have previously been described to be increased falls, fractures, myopathy, and depression. Loss of apetit, leading to low intake through food and skin problems are assumed to cause deficiency. The aim was to find possible clinical indicators of vitamin D deficiency in Swedish and immigrant women at a primary health care center in Sweden.

Methods: 61 women, aged 18 to 75 years, were interviewed about the following symptoms: Pain in joints and muscles, balance problems, depression, skin problems and loss of appetite. Blood samples were collected from all patients. Patients with low plasma levels of 25-OH-vitamin (25(OH)D) < 25 nmol/L (<10 ng/mL) were compared to those with higher levels.

Results: Pain in muscles and/or joints and balance problems were more frequent in patients with vitamin D deficiency. Depression was not more frequent in patients with deficiency. Loss of appetite and skin problems were not more frequent for deficient patients.

Conclusion: Pain in muscles and joints as well as balance problems may be clinical indicators of vitamin D deficiency. Depression, skin problems and loss of appetite do not seem to distinguish patients with deficiency.

### 2.13. Vitamin D Status during Acute Phase of Burn Injury: Is 25(OH)D Measurement an Accurate Marker?

#### Rousseau, A.F.; Damas, P.; Ledoux, D.; Carlisi, A.; Lukas, P.; Gadisseur, R.; Cavalier, E.

Background: Burn patients are at increased risk of hypovitaminosis D because of skin damages. Based on the skeletal and non skeletal effects of vitamin D (VD), it seems reasonable to optimize their VD status. Circulating VD is predominantly bound to vitamin D binding protein (VDBP) and albumin (ALB) and hence assessment of VD status during acute phase of critical illness may be hazardous. During acute burn injury indeed, concentrations of VDBP and ALB may fluctuate widely because of fluid resuscitation and systemic inflammation, leading to analytical interferences with current 25(OH)D assays. The aim of our observational study was to compare VD status of healthy adults and adult burns in the acute phase of burn care.

Methods: From 03/12 to 01/13, adult burns admitted within 24 h after injury with burn surface area (BSA) ≥10% were included. Liver or renal failure was considered exclusion criteria. Blood sample was collected at admission. During autumn 2012, blood samples were collected from healthy adults working in our lab. We measured serum levels of 25(OH)D using chemoluminescent immunoassay (Liaison^®^ DiaSorin^®^, coefficient of variation <6%), ALB using spectrophotometry (Cobas^®^, Roche^®^) and VDBP using ELISA (R&D^®^). Free 25(OH)D was calculated using VDBP and ALB concentrations according to published equations (Powe, C.E., et al*. J. Bone Miner. Res*. 2011, *26*, 1609-1616.). Free fraction, expressed as %, was considered as the ratio free 25(OH)D/total 25(OH)D (*i.e.*, sum of free and bound fractions). Data are expressed as median (min-max). Data were compared using unpaired *t* test or Mann-Whitney test according to results of Shapiro-Wilk normality test (*p* < 0.05 significant).

Results: 24 burn adults (BA) with 15(10–85)% BSA and 29 healthy adults (HA) were included. BA were older: 46(19–86) years *vs.* 26(22–60) years. Body mass index was not different between groups: 24.5(19.4–34.5) kg/m^2^ in BA *vs.* 23.5(17.4–32.9) kg/m^2^ in HA. Blood sample from BA was collected 6(1–16) h after injury. Serum 25(OH)D levels were significantly lower in BA: 13.5(6–42) ng/mL *vs.* 19.8(7.8–35) ng/mL in HA (*p* = 0.0176). Similarly, ALB was significantly lower in BA: 36(20–47) g/L *vs.* 49(44–90) g/L in HA (*p* < 0.0001), as was VDBP levels in BA: 221.3(89.3–319.9) mg/L *vs.* 253.6(63–691) mg/L in HA (*p* = 0.0269). There was no significant difference in calculated free 25(OH)D between groups (*p* = 0.5723): 4.88(2.1–16.45) pg/mL in BA *vs.* 5.65(2.44–21.91) pg/mL in HA. Free fraction was significantly higher in BA (*p* = 0.0413): 0.033(0.023–0.07)% *vs.* 0.028(0.011–0.08)% in HA.

Conclusions: Based on a single immunoassay during fluid resuscitation, 25(OH)D levels would suggest that hypovitaminosis D was more pronounced in our burn adults than healthy ones while free 25(OH)D levels were similar. Definition of VTD deficiency and clinical research are currently based on 25(OH)D levels and not on free 25(OH)D levels. At present, immunoassay measurement of free 25(OH)D is not routinely available and not widely validated. However, when referring to VD physiology, it is not yet established if free 25(OH)D is clinically more relevant than 25(OH)D. Particularly in burn patients, further studies should necessarily include VDBP measurement and free 25(OH)D assessment in order to build knowledge on such VD status approach.

### 2.14. Effect of Optimized Cholecalciferol and Calcium Intakes on Bone Mineral Density: A Randomized Controlled Trial in Adult Burns During Sequelae Stage

#### Rousseau, A.F.; Foidart-Dessalle, M.; Remy, C.; Ledoux, D.; Damas, P.; Cavalier, E.

Background: Severe burn injury may lead to osteopenia due to immobilization, systemic inflammatory and neuro-endocrine responses. Moreover, burn patients (BP) are at risk of hypovitaminosis D (hVD) because of sun exposure limitation, impaired biosynthetic function of burn scar and adjacent normal skin, and abnormalities in the calcium (Ca)—parathormone axis. Data about burn related hVD mainly concern children. At present, there are no data about vitamin D (VD) supplementation, osteopenia and bone mineral density (BMD) in adult burn patients. Aim of our randomized controlled trial was to assess effect of 1 year cholecalciferol (VD3) and Ca substitution on BMD in adult burns in sequelae stage.

Methods: BP > 18 years with thermal burns dating from 2 to 5 years were randomized in 2 groups before any VD status assessment. During 12 months, they either received a quaterly IM injection of 200,000 IU VD3 and daily oral Ca (500 mg or 1 g according to basal daily intakes) in group D (GD) or placebo in group P (GP). At initiation (M0) and completion (M12) of the study, serum level of 25(0H)D and 3rd generation parathormone (PTH) were measured using Liaison^®^ (DiaSorin^®^). Total Ca level was assayed using Cobas^®^ automate (Roche^®^). Dual X-ray absorptiometry (DXA) was performed on lumbar spine and femoral neck. Results are expressed as T-score. DXA reference site was spine for BP <50 years and hip for BP ≥ 50 years. Data were expressed as median (min-max) and compared using Mann-Whitney or Wilcoxon test as appropriate. Correlation between burn surface area (BSA) and bone status was assessed using non parametric Spearman test. A *p* < 0.05 was considered significant.

Results: 15 BP of 51(25–64) years were included at 3(2–6.5) years post-injury. BSA was 30(10–60)%. There were more women in GP (3/7 *vs.* 1/8 in GD). Age, BSA and body mass index was similar in both groups. At MO, 25(OH)D levels were not statistically different between groups: 21(10–30) ng/mL in GD and 13(7–36) ng/mL in GP. At M0, 86.6% of the global population presented hVD (25(OH)D < 30 ng/mL). At M12, 25(OH)D increased in GD, reaching 40(37–61) ng/mL (*p* = 0.0078), but did not change in GP (15(8–39) ng/mL). In 1 year, 25(OH)D increased by 111(57–270)% in GD while level change ranged from −14% to 100% in GP (*p* = 0.0011). PTH was higher in GP at inclusion: 41(29–77) pg/mL *vs.* 23(13–50) pg/mL in GD (*p* = 0.015). At M12, no significant variation in PTH levels was observed in both groups. Ca was within normal range in both groups at M0 and M12. T-score at M0 was similar in both groups: −1(−1.9–1.5) in GD and −0.5(−2.9–1.4) in GP (*p* = 0.84). At M0, 53% of all BP (8 BP) were osteopenic (T-score ≤ −1). Among them, 2 men were <30 years and 1 man was <40 years: BSA was <30% for 2 of them. No correlation was found between BSA and initial T-score. No significant increase in T-score was detected at M12 in both groups, including osteopenic BP.

Conclusions: Despite increasing 25(OH)D levels around 40 ng/mL, a 1 year treatment combining high VD3 doses and optimized Ca intakes failed to increase BMD in burn adults in sequelae stage. These results may be due to mild severity of burn injury or limited duration of the protocol. However, occurrence of osteopenia in young BP is worrying, especially as our VD and Ca regimen seemed to have no therapeutic effects. This finding should be of concern to design further investigations.

### 2.15. The Darker Side of Brightness—Vitamin D Deficiency in Pregnancy and Childhood with Inadequate Treatment Options in Wellington, New Zealand

#### Judkins, A.M.

Background: Working in a high needs community with a large refugee population living at 41degrees South, in 2004 we diagnosed 3 children with rickets within a month. This resulted in a research project within our practice, where we identified and treated vitamin D deficiency in pregnancy to try and prevent rickets in our community. Due to the unavailability of subsidized daily doses of vitamin D and the unaffordability of vitamin D testing in New Zealand we have established a less than ideal process for treating and preventing vitamin D deficiency in our identified high risk women and children, using monthly doses of 50,000 IU Cal-D forte for women and vitadol-C (multivitamin) for breast feeding babies. We have decreased the incidence of rickets in our community but with limited subsidised treatment options in a poor and at risk community we are still concerned about the impact of vitamin D deficiency on our vulnerable children.

Method: Identified as high risk low vitamin D community we engaged with Massey University’s research into vitamin D deficiency in preschool children in NZ. 47 children had their vitamin D levels measured within our practice in August and September 2012. Primary and secondary medical service contacts were compared between those children who had a vitamin D level <25 nmol/L and those >25 nmol/L over a 2 years period.

Results: Vitamin D levels ranged from 6–84 nmol/L. 19 (40%) children had levels <25 nmol/L. African and Pacific children had the lowest levels measured although there were Middle Eastern, Maori, European and Asian children with levels <25 nmol/L. Children with levels <25 nmol/L had on average 19 health professional contacts over the 2 years whilst children with levels >25 nmol/L had 12 contacts over the same time period. Most of these contacts were for respiratory tract infections. Failure to thrive is identified. Although no children were taking vitamin supplements at the time of the study, the average vitamin D level in African children who had ever been prescribed vitadol-C was 34 nmol/L whilst those who had never been prescribed vitadol-C was 16 nmol/L.

Conclusion: We have decreased the incidence of rickets in our community and raised awareness of the need for vitamin D supplementation in at risk communities in NZ. However this window-shot identifies ongoing cross cultural vitamin D deficiency in our community. Dissection: Should we spend resources on testing vitamin D level or treat universally? How hard should we fight for improved affordable access to daily vitamin D supplementation? Is second or third best practice OK?

### 2.16. Vitamin D Economy?—Dietary Calcium Does not Interact with Vitamin D_3_ in Terms of Determining the Response and Catabolism of Serum 25-Hydroxy Vitamin D during Winter in Older Adults

#### Hayes, A.; O’Donovan, S.M.; Zhang, Y.; Kinsella, M.; Galvin, K.; Kiely, M.; Seamans, K.M.; Cashman, K.D.

Background: Interactions between dietary calcium and vitamin D may have implications for the regulation of serum 25-hydroxy vitamin D (25(OH)D) and its catabolism, and consequently for the dietary requirement for vitamin D ((IOM) Institute of Medicine. The National Academies Press : Washington, DC, USA, 2011).We investigated whether different levels of habitual calcium intake influenced serum 25(OH)D and indices of vitamin D activation and catabolism during winter and in the context of both adequate and inadequate vitamin D intakes.

Methods: A 15-week winter-based, randomized, placebo-controlled, double-blind vitamin D_3_ intervention (20 μg/day) study in free-living men and women aged ≥50 years (*n* = 125) stratified according to calcium intake (“moderate-low” (<700 mg/day) or “high” (>1000 mg/day)). Serum 25(OH)D concentrations (measured via LC-tandem MS) was the primary outcome and serum Ca, PTH, 1,25(OH)_2_D, 24,25(OH)_2_D, vitamin D binding protein and free 25(OH)D were exploratory outcomes.

Results: Repeated measures ANOVA showed that there was no significant (*p* = 0.2) time × vitamin D treatment × calcium intake grouping interaction effect on mean serum 25(OH)D concentration over the 15-week intervention period. This finding was also the case when a “low” (<550 mg/day) calcium intake was used in place of the <700 mg/day cut-off. Serum 25(OH)D increased and decreased in the vitamin D_3_ and placebo groups, respectively, and of similar magnitudes in those with calcium intakes <700 and >1000 mg/day. The response of serum PTH, 1,25(OH)_2_D, 24,25(OH)_2_D and free 25(OH)D concentrations significantly differed among the vitamin D_3_ (generally increase in vitamin D metabolites) and placebo groups (decrease in vitamin D metabolites and increase in PTH), but not by calcium intake grouping.

Conclusion: We did not find evidence of a vitamin D sparing effect of high calcium intake. Thus, recent dietary vitamin D requirement estimates will cover the vitamin D needs of even those in the population who do not have adequate calcium intakes.

### 2.17. Anaphylaxis Admissions in Chile Are Strongly Associated with Higher Latitude and Lower Solar Radiation

#### Hoyos-Bachiloglu, R.; Morales, P.; Cerda, J.; Talesnik, E.; González, G.; Camargo, C.A., Jr.; Borzutzky, A.

Background: Recent studies suggest an association between higher latitude, a proxy of vitamin D (VD) status, and allergic diseases. Chile provides an ideal setting to study this association due to its latitude span and high rates of VD deficiency in southern regions. The aim of this study is to explore the associations of latitude and solar radiation with anaphylaxis admission rates.

Methods: We reviewed anaphylaxis admissions in Chile’s national hospital discharge database between 2001 and 2010 and investigated associations with latitude, solar radiation and sociodemographic factors. Anaphylaxis was defined by the following ICD-10 codes: T78.0 (food-induced anaphylaxis), T78.2 (anaphylaxis, unspecified), and T88.6 (drug-induced anaphylaxis).

Results: 2316 anaphylaxis admissions were registered, with the following diagnoses: food-induced anaphylaxis in 230 cases (10%), drug-induced anaphylaxis in 208 cases (9%), and unspecified anaphylaxis in 1878 cases (81%). Median age of patients was 41 years and 53% were female. National anaphylaxis admission rate was 1.41 per 100,000 persons per year. We observed a strong north-south increasing gradient of anaphylaxis admissions (β = 0.04, *p* = 0.01), with increasing rates south of latitude 34°S. A significant association was also observed between solar radiation and anaphylaxis admissions (β = −0.11, *p* = 0.009). Latitude was associated with food-induced (β = 0.05, *p* = 0.02), but not drug-induced (β = −0.002, *p* = 0.27), anaphylaxis. The association between latitude and food-induced anaphylaxis was significant in children (β = 0.01, *p* = 0.006), but not adults (β = 0.003, *p* = 0.16). Anaphylaxis admissions were not associated with regional sociodemographic factors like poverty, rurality, educational level, or ethnicity.

Conclusions: Anaphylaxis admission rates in Chile are highest at higher latitudes and lower solar radiation, used as proxies of VD status. The associations appear driven by food-induced anaphylaxis. Our data support a possible role of VD deficiency as an etiological factor in the high anaphylaxis admission rates found in southern Chile.

### 2.18. Sun Exposure, Dietary Intake, and Nutritional Supplement Use among Children with Atopic Dermatitis in Santiago, Chile

#### Borzutzky, A.; Le Roy, C.; Iturriaga, C.; Vera, C.; Silva, S.; Cifuentes, L.; Cristi, F.; Hoyos-Bachiloglu, R.; Navarrete, C.; Camargo, C.A. Jr.

Background: Low vitamin D (VD) status is common among patients with atopic dermatitis (AD) and some studies suggest an association between VD deficiency and increased AD severity. We hypothesized that lifestyle habits of patients with AD may adversely affect VD status.

Methods: We conducted a cross-sectional study of 68 children with AD, who were recruited from a large university-affiliated allergy and dermatology clinic in Santiago, Chile. Inclusion criteria included age younger than 18 years and AD as defined by Hanifin and Rajka. Exclusion criteria were known VD deficiency and disorders of VD metabolism. AD severity was assessed by Scoring Severity of AD (SCORAD) index. Patients’ parents were asked by survey about the patient’s AD characteristics and about several lifestyle-related factors that affect VD status, such as sun exposure, dietary intake and nutritional supplement use. Data analysis used descriptive statistics, *t*-test, and chi-square. A two-tailed *p* < 0.05 was considered statistically significant.

Results: The mean age of the enrolled AD patients was 5.7 ± 4.5 years and 53% were female. The mean SCORAD index was 34 ± 19, with 34% classified as having mild AD, 44% moderate, and 22% severe. 62% reported allergic comorbidity: 35% allergic rhinitis, 25% asthma, and 19% food allergy. A history of skin infections was present in 55% of patients. Regarding seasonal variation of AD, 19% worsened in fall-winter, 35% worsened in spring-summer, and 44% said that they were not affected by seasons. 41% of parents considered that sun exposure worsened AD severity in their child, while only 12% of parents considered sun exposure improved their AD. Winter and summer seasonality of AD flares was significantly associated with parental belief about sun exposure improving or worsening AD severity, respectively (*p* = 0.001). 81% of patients used sunblock frequently or always in summer, and 19% used sunblock frequently or always during other seasons. Most patients (57%) performed no outdoor activities or less than 4 h/week on a regular basis. Almost one-third of patients watched TV screen/computer/videogame more than 2 h per day. Regarding dietary intake of VD, 9% of patients consumed VD-fortified milk formula, but only one patient (1.5%) had at least 400 IU VD intake daily in milk. Of children older than 1 year of age, 16% did not eat fish (a potentially good source of vitamin D) and 40% ate fish less than once per week. Only 8% of patients took multivitamin or vitamin D supplements daily. None of these VD-related factors (seasonality of AD, sun exposure, dietary intake or nutritional supplementation) were associated with SCORAD index (all *p* > 0.05).

Conclusions: Children with AD in Santiago, Chile appear to have little sun exposure and, in a significant proportion, a parental belief that sunlight worsens AD. In addition, a very low dietary intake of VD-rich and VD-fortified food was observed. Moreover, use of VD-containing nutritional supplements was rare. Low exposure to VD sources was not significantly associated with AD severity. Together, these lifestyle factors put these children with AD at increased risk for VD deficiency, a condition that potentially may worsen their AD severity. Upcoming work with this group of patients will include 25OHD testing, and a randomized, double-blind, placebo-controlled trial that will test the effect of weekly vitamin D supplementation on AD severity in children.

### 2.19. Seasonal Variation in Foetal Lateral Ventricular Diameter: Does Maternal Vitamin D Deficiency Impair Foetal Neurodevelopment?

#### Gandhi, B.; Aquilina, J.; Khan, K.S.; Hooper, R.L.; Marleen, S.; Martineau, A.R.

Background: There is growing evidence from observational epidemiological and animal studies that foetal vitamin D deficiency may be a risk factor for the development of neuropsychiatric disorders in adulthood, and that this may be associated with increased lateral ventricle diameter (ventriculomegaly) in the foetal brain. In the UK, maternal serum 25-hydroxy vitamin D concentrations exhibit seasonal variation, with peak levels after summer and the lowest levels in spring. If vitamin D status does indeed impact on neurodevelopment, seasonal variation in lateral ventricular diameter might be expected. We therefore conducted a retrospective observational study to determine whether there is seasonal variation in lateral ventricular diameter among foetuses undergoing routine ultra-sound scans at 18–26 weeks of gestation.

Methods: Data from all pregnant women (*n* = 35,594) having a routine foetal anomaly scan at 18–26 weeks gestation at the Royal London Hospital, UK, between February 2000 and February 2012 were examined. Isolated ventriculomegaly was defined as a lateral ventricle diameter ≥10 mm and ≤16 mm. Foetuses with a lateral ventricle diameter >16 mm, known neurological pathology or pathology affecting ventricular diameter were excluded. A total of 49,609 anomaly scans met inclusion criteria. Cosinor analysis was used to determine the presence or absence of seasonal variation in mean lateral ventricular diameter.

Results: We found evidence of a small but highly statistically significant (*p* < 0.001) variation in mean lateral ventricular diameter, highest in October-December (6.59 mm), and lowest in April-June (6.53 mm). Mean lateral ventricular diameters in January–March (6.54 mm) and July–September (6.57 mm) were intermediate. Isolated ventriculomegaly was found in 118 foetuses (approximately 2 per 1000 pregnancies, consistent with reports from other settings). No seasonal variation in the proportion of foetuses with ventriculomegaly was found.

Conclusions: To our knowledge, this is the first demonstration of seasonal variation in foetal lateral ventricular diameter. Foetal ventricular size at the time of routine anomaly scans is likely to reflect in utero conditions one to two calendar year quarters previously. Therefore, the larger mean foetal lateral ventricle size seen in October–December is likely to relate to adverse exposure in June–September, when maternal vitamin D status is highest. Our data do not therefore support a role for vitamin D in protection against foetal lateral ventriculomegaly. Alternative explanations for our findings should be considered: seasonal variation in maternal concentrations of folic acid, which plays a key role in neurodevelopment and which is degraded by cutaneous exposure to ultraviolet radiation, may be implicated.

### 2.20. Interactions of Vitamin A with the Effects of Vitamin D and Their Potential for Confounding Investigations on the Effects of Vitamin D on Human Health

#### Boucher, B.J.

Background: Ligand-bound vitamin D: retinol-X receptor heterodimers are important vitamin D effectors. Retinol-X receptors heterodimerize with retinol-A receptors and the availability of RXR falls in the presence of excessive vitamin A. Westernized populations often ingest more vitamin A than is advisable and up to 30% have circulating concentrations above the recommended maximum level (e.g., in NHANES111). Populations in developing countries are often deficient in vitamin A. U or J shaped curves for benefits of increases in vitamin D supplementation in are a growing concern, as reported in the Western world. Retinoid formation from carotinoids is self-regulated and avoids vitamin A toxicity. High retinoid intakes have been associated with increases in mortality. 2 large Randomized Controlled Trials (RCTs) of vitamin A have had to be stopped due to an increased incidence of lung cancer and in cardiac deaths.

Methods: Review of the literature (using original literature identified and accessed through PubMed *etc.*), and the examination of mechanistic, experimental and epidemiological data.

Results: Retinol does not affect vitamin D metabolism directly. Vitamin D does not affect serum retinoids directly. High dose retinol prevents toxic effects, including death, in vitamin D poisoning, experimentally. High-intakes of retinol induces rickets in normal animals and are associated with increased osteoporotic risks in older women and may be associated with increased fracture risks in some population groups. High intakes (above recommended daily amounts) of vitamin A have been reported to counteract the effects of vitamin D (including effects in RCTs). For Example: Vitamin D-induced calcium absorption by the gut is reduced by vitamin A in fish, animals and humans. GWAS data shows raised retinol levels in those with specific retinol binding protein gene polymorphisms. Overall reductions in lung cancer risks in never-smokers in the Women’s Health Initiative (1771 cases in 128,779 women) were seen with vitamin D intakes >400 IU/day (OR = 0.37, 95% CI: 0.18–0.77); however, RCT data for the 12% of women given vitamin D supplements showed lower lung cancer risk only in those with low intakes of retinol (OR = 0.69, 95% CI, 0.5–0.96). RCTs of retinyl palmitate increased lung cancer risk in the large CARET trial. Lower death rates from malignant melanoma are seen with higher vitamin D intakes (OR = 0.77, 95% CI, 0.64–0.93) but this benefit is smaller in those with high retinol intakes (OR = 0.99, 95% CI, 0.72–1.36).

Conclusions: (1) Since the use of supplements containing vitamin A as well as vitamin D is common, whether in cod liver oil or multi-vitamins, especially in developed countries, vitamin A status is likely to confound RCTs of vitamin D in Westernized populations; (2) In particular, high vitamin A intakes could account for J or inverse U shaped curves for outcomes in RCTs, as could gene polymorphisms increasing circulating retinol/retinoids; (3) Conversely, poor vitamin D status may be expected to confound RCTs of vitamin A in developing countries; (4) As well as vitamin A intakes, other lifestyle factors that are potential confounders of the effects of vitamin D should be considered in the planning of future RCTs of supplemental vitamin D, including intakes of other nutrients, tobacco usage, and also betel-chewing which may increase catabolism of vitamin D and is a habit used by 10% of the world population.

### 2.21. The Associations of Serum 25-Hydroxy Vitamin D with Measures of Lung Function in a Prospective Study of Danish adults

#### Thuesen, B.H.; Husemoen, L.L.; Skaaby, T.; Jørgensen, T.; Linneberg, A.

Background: Vitamin D may influence tissue modelling and repair in the lungs throughout life and thus low levels of vitamin D may have a negative impact on lung function. In addition, an association between inadequate vitamin D levels and impaired lung function could be explained by an increased risk of respiratory infections or a negative influence on muscle function. The purpose of this study was to investigate potential associations of serum levels of 25-hydroxy vitamin D (25(OH)D) with measures of lung function in a prospective study of Danish adults.

Methods: This study included 4999 persons from a general population-based cohort of adults aged 30–60 years at the baseline examinations in 1999–2001. 3032 of those included at baseline also participated at a follow-up examination 5 years later. Serum levels of (25(OH)D) were measured by high-performance liquid chromatography (HPLC) at baseline. Lung function (FEV1 and FVC) was measured by spirometry at baseline and follow-up. Predicted FEV1, FEV1/FVC and measured FEV1 relative to predicted FEV1 (FEV1% pred) was calculated. Potential associations were evaluated by linear and logistic regression models adjusted for confounding by age, sex, smoking, BMI, physical activity, dietary habits, and month of blood sampling.

Results: We found that low levels of 25(OH)D was associated with lower FEV1%pred in the cross-sectional analyses. The odds ratio (OR) of FEV1% pred <80% among participants in the highest quartile of serum 25(OH)D compared to those in the lowest quartile was 0.66 (95% confidence interval (CI): 0.49–0.74). In contrast, prospective analyses indicated an association between high levels of 25(OH)D at baseline and adverse changes in lung function after 5 years. OR (95% CI) of incident FEV1% pred <80% was 1.73 (1.06–2.82) in the highest quartile of serum 25(OH)D compared to the lowest quartile. Also linear regression analyses showed that high levels of 25(OH)D were associated with adverse changes in FEV1/FVC (*p* = 0.01). Associations between 25(OH)D and lung function were more pronounced among smokers than non-smokers.

Conclusions: High serum levels of 25(OH)D were associated with higher FEV1% pred in cross-sectional analyses. However, this association was not supported by the longitudinal analyses since high serum levels of 25(OH)D at baseline appeared to be associated with detrimental changes in lung function at 5-year follow-up. The associations between serum levels of 25(OH)D and lung function were more pronounced among daily smokers than never smokers. Randomized controlled trials are needed to fully elucidate the potential relations of vitamin D with measures of lung function.

### 2.22. Trial of Vitamin D Supplementation in Adults with Inhaled Corticosteroid-Treated Asthma

#### MacLaughlin, B.; Kilpin, K.; Jolliffe, D.A.; Hooper, R.L.; Timms, P.M.; Grigg, J.; Choudhury, A.; Rajakulasingam, R.K.; Simcock, D.; Barnes, N.; Corrigan, C.; Hawrylowicz, C.M.; Griffiths, C.J.; Martineau, A.R.

Background: Exacerbations of asthma are commonly precipitated by viral upper respiratory infections (URI), but interventions to prevent these are lacking. Vitamin D metabolites support anti-viral activity and enhance responsiveness to corticosteroids for production of the anti-inflammatory cytokine interleukin-10 *in vitro*. Vitamin D deficiency has been reported to associate with increased risk of asthma exacerbation and increased risk of URI in patients with asthma. Randomised controlled trials of vitamin D supplementation in children with asthma for prevention of exacerbation and URI have reported positive results; such trials have not previously been conducted in adults.

Methods: We conducted a multi-centre double-blind randomised placebo-controlled trial of vitamin D supplementation in adults with inhaled corticosteroid (ICS)-treated asthma in London, UK. 250 patients were allocated to receive the intervention (vitamin D_3_ 3 mg 2-monthly per os) or control (placebo 2-monthly) over the course of one year. Co-primary outcomes of the trial were time to first severe exacerbation (*i.e.*, one requiring oral corticosteroids or emergency treatment, or resulting in 25% dip in peak expiratory flow rate for ≥2 days) and time to first URI (determined by a validated acute respiratory symptom score recorded prospectively in a symptom diary). Secondary outcomes included mean St George’s Respiratory Questionnaire (SGRQ) score, asthma control test (ACT) score, mean exhaled nitric oxide (eNO) concentration, mean % predicted FEV1 and serum 25-hydroxy vitamin D (25(OH)D) concentration.

Results: 125 participants were allocated to the intervention arm of the trial, and 125 to the control arm. Mean baseline serum 25(OH)D) concentration was 49.6 nmol/L. Vitamin D supplementation was effective in elevating serum 25(OH)D concentration in the intervention *vs.* control arm (69.2 *vs.* 46.2 nmol/L respectively at one year, *p* < 0.001), but it did not influence time to first severe exacerbation (adjusted Hazard Ratio [aHR] 1.02, 95% CI 0.69 to 1.53, *p* = 0.91) or time to first URI (aHR 0.87, 95% CI 0.64 to 1.16, *p* = 0.34). Vitamin D supplementation was associated with improved quality of life, as evidenced by a modest but statistically significant reduction in total SGRQ score in intervention *vs.* controls arms at 2 months (3.9 point difference, *p* = 0.005), 6 months (3.7 point difference, *p* = 0.038) and 12 months (3.3 point difference, *p* = 0.060). No effect of the intervention was seen on mean ACT score, mean eNO concentration or mean % predicted FEV1 (*p* > 0.05 for all). The effect of the intervention on co-primary outcomes did not differ according to baseline vitamin D status.

Conclusions: Intermittent bolus-dose vitamin D supplementation significantly improved vitamin D status but did not influence time to exacerbation or time to URI in a population of adults with ICS-treated asthma and a high prevalence of vitamin D deficiency at baseline. Vitamin D supplementation was associated with modest improvement in quality of life as measured by SGRQ.

### 2.23. Efficacy of 25(OH) Vitamin D Supplementation and Physical Activity in Improving Musculoskeletal Health in Individuals with Chronic Kidney Disease (CKD). A Multicenter Randomized, Controlled Trial

#### Ahmed, B.; Kashif, W.; Iqbal, R.; Habib, A.; Nasir, K.; Mehmood, A.; Sarwar, S.

Background: We investigated the role of vitamin D (VD) and targeted physical activity in improving musculoskeletal health among chronic kidney diseases (CKD) patients.

Methods: An open-label, randomized controlled trial of VD replacement in stage 2–4 CKD patients, attending two nephrology clinics of Karachi, Pakistan was undertaken. 2637 subjects were contacted, 115 found to be deficient for 25 hydroxy vitamin D (25OHD) levels were block randomized to either 4000 IU of daily oral vitamin D_3_ (cholecalciferol) or combination of daily drops along with targeted physical therapy for three months. A predesigned questionnaire along with IPAQ was administered to assess the physical activity. Baseline biochemical testing for calcium, Phosphate, bone alkaline phosphatse, iPTH were performed. Musculoskeletal health was assessed by trained physical therapist by measuring bicep strength, back flexibility and fat composition and hand grip assessment. The outcome was improvement in musculoskeletal health to be assessed by plasma iPTH, calcium, bone specific alkaline phosphatase and hand grip strength assessment and improvement in serum 25OHD levels. Analysis was by intention to treat.

Results: Of the 115 VD deficient subjects enrolled at baseline, 42 were lost to follow-up (47% in vitamin D alone *vs.* 52% in vitamin along with physical activity group). The mean 25OHD level at the baseline was comparable in both the arms. At follow-up, 25OHD status was 85.0 ± 8.9 nmol/L in the VD group and 95.8 ± 6.6 nmol/L in VD along with physical activity group. There was significant improvement in the musculoskeletal health in the second arm compared to VD alone as well as within the group improvement (*p* value ≤ 0.03).

Conclusion: Treatment with both VD and targeted physical activity was found to be effective for improving musculoskeletal health (strength, muscle force, or power) in CKD patients.

### 2.24. Low Serum Vitamin D Concentration among the Patients with Chronic Mechanical Ventilation

#### Svirsky, B.; Kaykov, E.; Ben-Israel, J.

Background: The serum Vitamin D concentration measurement is of much importance in chronically ventilated patients because they usually are at risk to develop deficiency and Vitamin D can influence morbidity, mortality, respiratory function, tracheobronchial structure and even weaning process. The purpose of our study was to examine the prevalence of Vitamin D deficiency and to find correlation with outcome.

Methods: We studied 152 (mean age 71 ± 14 years) chronically ventilated patients in long-term care department to access the prevalence of Vitamin D deficiency. All patients received 200–400 IU of Vitamin D in diet. We used Liaison 25-OH Vitamin D (DiaSorin^®^) immunoassay to measure serum concentration of 25-OH vitamin D. The patients were followed for major outcomes after one year: weaning from mechanical ventilation and death.

Results: Low Vitamin D concentration (15.6 ± 7.6 ng/mL) was found in 95 percent of patients when 28.5% were in Vitamin D deficient state (<10 ng/mL) and 66.4% in Vitamin D insufficiency state (<10–30 ng/mL). COPD group of patients and patients who develop tracheomalacia were found to have lower Vitamin D levels than others (32.2 ± 18 ng/mL *vs.* 42 ± 18 ng/mL (*p* < 0.01) and 36 ± 18 ng/mL *vs.* 41 ± 18 ng/mL (*p* < 0.131) respectively). Patients with enteral tube feeding had significantly higher levels of vitamin D than patients with oral food intake (42 ± 16 ng/mL *vs.* 33 ± 20 ng/mL, *p* < 0.005). Age, length of hospitalization and drugs were found not to be risk factors to develop Vitamin D deficiency. The Vitamin D deficiency was correlated with overweight and weight gain (*p* < 0.027). We did not found correlation between the outcome and Vitamin D levels.

Conclusion: our study found that almost all patients with chronic mechanical ventilation and especially COPD patients are in Vitamin D insufficient state, but there is now correlation with successful weaning and death. Further investigations should be performed to estimate the influence of Vitamin D replacement on morbidity, mortality and weaning in chronic ventilated patients.

### 2.25. Vitamin D Supplementation during Pregnancy and Infancy Reduces Primary Care Antibiotic Use for Acute Illnesses during Infancy

#### Grant, C.C.; Stewart, A.; Scragg, R.; Milne,; Rowden, J.; Ekeroma, A.; Wall, C.; Mitchell, E.; Kaur, S.; Waymouth, E.; Trenholme, A.; Crane, J.; Camargo, C.A., Jr.

Background: Antibiotic prescribing in primary care is one of the most important drivers of antibiotic resistance. Strategies to reduce antibiotic prescribing are a current focus of health care quality improvement. We aimed to determine whether vitamin supplementation during pregnancy and infancy reduces antibiotic prescribing for acute illnesses during infancy.

Methods: We performed a randomised, double-blind, placebo-controlled trial in Auckland, New Zealand (latitude 36oS). Pregnant mothers, from enrolment at 27 weeks gestation to birth, and then their infants, from birth to age 6 months, were assigned to receive placebo or one of two dosages of daily of oral vitamin D_3_. The enrolled woman/infant pairs were randomised to: placebo/placebo, 1000 IU/400 IU, or 2000 IU/800 IU. Serum 25-hydroxy vitamin D (25(OH)D) concentration was measured at enrolment, 36 weeks gestation, on cord blood samples, and at 2, 4 and 6 months of age. We audited the primary care records of enrolled infants to a median age of 18 months, identified all acute visits and determined whether oral antibiotics were prescribed at each visit. Study investigators, parents and all treating physicians remained blinded to group allocation until after completion of the primary care audit.

Results: 260 pregnant women were randomised to placebo (*n* = 87), lower dose (*n* = 87) or higher dose (*n* = 86) vitamin D. In comparison with placebo, serum 25(OH)D concentrations were higher during pregnancy and infancy in both the lower and higher dose vitamin D groups. During infancy, mean serum 25(OH)D was greater in the higher than the lower dose group at 2, 4 and 6 months of age.1 Primary care data, collected to a median (25th–75th centile) age of 18 (14 to 21) months, were available on 238 (92%) of the children. At least one acute primary care visit was made by 232 (98%) of the children with the median (25th, 75th centile) number of acute visits being 8 (4, 13). Vitamin D supplementation did not affect the proportion making any acute visits (placebo 98%, lower dose 100%, higher dose 95%, *p* = 0.12), nor the median number of acute visits (placebo 8, lower dose 8, higher dose 8, *p* = 0.43). In comparison with placebo, the proportion of children prescribed antibiotics at any acute primary care visit did not differ for the lower dose vitamin D group (85% *vs.* 77%, *p* = 0.24) but was smaller in the higher dose group (85% *vs.* 71%, *p* = 0.04). The number of acute visits at which an antibiotic was prescribed did not differ between groups (placebo 2, lower dose 2, higher dose 3, *p* = 0.50)

Conclusions: Vitamin D supplementation at 2000 IU/day from 27 weeks gestation until birth and then at 800 IU/day from birth to age 6 months may reduce the proportion of children prescribed antibiotics at acute primary care visits during the first 18 months of life.

### 2.26. Does Prenatal Exposure to Vitamin D Fortified Margarine and Milk Alter Birth Weight? A Societal Experiment

#### Jensen, C.B.; Berentzen, T.L.; Gamborg, M.; Sørensen, T.I.A.; Heitmann, B.L.

Background: Several studies have investigated the association between vitamin D in pregnancy and birth weight, but the conclusion of the studies are not clear. Aim: The present study examined if exposure to vitamin D from fortified margarine and milk during prenatal life influenced birth weight.

Methods: The project was based on the Danish vitamin D fortification programs (mandatory fortification of margarine 1961–1985 and voluntary fortification of low fat milk 1972–1976). The influence of vitamin D exposure during prenatal life on birth weight was investigated among 59,411 Danish children by comparing birth weight among individuals born before, during, and after fortification. Children born around the periods of fortification were identified in the Copenhagen School Health Record Register in which information of birth weight were available for all school children in Copenhagen.

Results: Mean birth weight (95% CI) was lower among exposed than non-exposed children around all periods (Milk initiation: −20.3 g (−39.2 to −1.4). Milk termination: −25.9 g (−46.0 to −5.7). Margarine termination: −45.7 g (−66.6 to −24.8)) except around the initiation period of margarine fortification, where exposed children were heavier than non-exposed children (Margarine initiation: 27.4 g (10.8 to 44.0)).

Conclusion: Exposure to vitamin D from fortified margarine and milk during prenatal life altered birth weight, but the associations were inconsistent. We speculate that the effect of vitamin D exposure in pregnancy is modified by the exposure prior to conception.

### 2.27. Prospective Study of Peri-Pregnancy Vitamin D Status, Season of Birth, and Risk of Peanut or Tree Nut Allergy during Childhood

#### Camargo, C.A., Jr.; Young, M.C.; Rifas-Shiman, S.L.; Frazier, A.L.

Background: In 2007, Camargo *et al.* reported a strong north-south gradient in EpiPen prescriptions in the USA (Camargo, C.A., et al. *J. Allergy Clin. Immunol.* 2007, 120, 131). This novel finding supported an a priori hypothesis that low UVB exposure and low vitamin D status have an etiologic role in food allergy. Subsequent studies confirmed this latitudinal gradient in Australia. We also identified that fall/winter births, in both USA and Australia, were more likely to develop food allergy. The current analysis, based on two large cohort studies, examined the association between peri-pregnancy vitamin D status in mothers, season of birth of their offspring, and the risk of incident peanut/tree nut (P/TN) allergy during childhood.

Methods: We included 7834 participants in the Growing Up Today Study 2, born between 1988 and 1994, who are offspring of women in the Nurses’ Health Study II. In 2006, we asked the offspring about doctor-diagnosis of food allergy. Mothers (who are registered nurses) were asked to confirm the diagnosis and to help provide medical records, including allergy test results. Two pediatricians, including a board-certified allergist/immunologist, independently reviewed each potential case and assigned food allergy status. We chose a priori to focus on P/TN because of its large number of cases, less confusion with non-allergic disorders (e.g., lactose intolerance), and >80% persistence beyond childhood. Maternal 25OHD level was predicted using a validated model (Bertrand, K.A., *et al.*, *Br. J. Nutr.* 2012, *108*, 1889–1896). Unadjusted and multivariable logistic regression was used evaluate associations between peri-pregnancy estimate of maternal 25OHD, season of birth, and incident P/TN allergy. We formally tested for effect modification of the exposure-outcome association by maternal history of atopy.

Results: Among the 7834 children, we identified 308 cases of food allergy (any food), including 139 cases of P/TN allergy. By quartile, the mean predicted 25OHD (range) in mothers was: 70(38–75), 78(75–80), 83(80–85), and 88(85–97) nmol/L. Unadjusted analysis showed a U-shaped association with maternal 25OHD, and higher risk among offspring born in fall/winter. In a multivariable model adjusting for both factors and 6 covariates, the adjusted OR (95% CI) for maternal 25OHD, by increasing quartile was: 1.00 (ref), 0.90 (95% CI, 0.54–1.52), 0.44 (0.23–0.84), and 0.95 (0.57–1.58). Compared to birth in spring/summer, the adjusted OR (95% CI) for P/TN allergy for fall/winter was 1.62 (1.11–2.38). Stratifying mothers into those with (*n* = 2673) or without (5161) atopy revealed a likely interaction between maternal 25OHD level and maternal atopy status (*p* interaction = 0.07). Among mothers with atopy, the adjusted OR (95% CI) for maternal 25OHD, by increasing quartile was: 1.00 (ref), 0.62(0.31–1.24), 0.25(0.11–0.61), and 0.86(0.46–1.63). By contrast, among mothers without atopy, the comparable values were: 1.00 (ref), 1.59, 1.02, and 1.08.

Conclusions: Peri-pregnancy vitamin D status of mothers had a U-shaped association with risk of P/TN allergy in offspring, with lowest risk for mothers with predicted 25OHD between 80–85 nmol/L. Moreover, children born in fall/winter were at higher risk of P/TN allergy. The U-shaped association between maternal 25OHD and child P/TN allergy may be limited to women with atopy. These prospective data support the hypothesis that maternal vitamin D status and child season of birth contribute to risk of childhood P/TN allergy.

### 2.28. Maternal 25-Hydroxy Vitamin D Concentrations in Early Pregnancy are Associated with Body Composition at Birth

#### Ní Chaoimh, C.; O’Donovan, S.M.; Murray, D.M.; Kenny, L.C.; Hourihane, J.O’B.; Kiely, M.

Background: Obesity is a risk factor for low circulating 25-hydroxy vitamin D (25(OH)D), which is the biomarker of vitamin D status, and there are consistent negative associations between 25(OH)D and measures of adiposity in adults and children. Relatively lower maternal circulating 25(OH)D concentrations during late pregnancy have been associated with lower fat mass at birth and higher adiposity during early childhood (Crozier SR, Harvey NC, Inskip HM, *et al.* Maternal vitamin D status in pregnancy is associated with adiposity in the offspring: findings from the Southampton Women's Survey. Am J Clin Nutr 2012; 96(1): 57-63). To date, little is known about the effect of low vitamin D status at birth on body composition during the neonatal period. The aim of this study was to examine the associations between maternal and umbilical cord 25(OH)D concentrations and body composition at birth.

Methods: Serum 25(OH)D was quantified at 15 weeks gestation and in umbilical cord in 1050 maternal-infant dyads participating in the Cork BASELINE Birth Cohort Study, using liquid chromatography-tandem mass spectrometry (LC-MS/MS), using a method which is traceable to the NIST higher order reference measurement procedure (Sempos, C.T., et al. *Scand J. Clin. Lab. Invest. Suppl.* 2012, 243, 32-40. Tai, S.S., et al. *Anal. Chem.* 2010, *82*, 1942-1948). Fat mass (FM) (kg) and fat free mass (FFM) (kg) were measured for 850 infants within 4 days of birth using air displacement plethysmography (PEA POD). Fat mass index (FMI) and fat free mass index (FFMI) (kg/m^2^) were calculated. Binary variables were developed to describe antenatal and socio-demographic data and univariate analysis was used to explore the predictors of infants being in the highest quartile for FMI and FFMI at birth. On the basis of these analyses, predictive models were developed using binary logistic regression.

Results: In the subgroup of 850 maternal-cord dyads in the current analysis, the median (IQR) maternal serum 25(OH)D concentration at 15 weeks gestation was 56(40–77 nmol/L) and 16 and 42% were <30 and <50 nmol/L respectively ((IOM) Institute of Medicine. Dietary Reference Intakes for Calcium and Vitamin D. Washington, DC: The National Academies Press, 2011). The median (IQR) cord serum 25(OH)D was 31 (20–46 nmol/L) and 35 and 66% were <25 and <40 nmol/L, respectively. On the basis of the correlation between maternal and cord 25(OH)D concentrations collected at the time of delivery (Kiely M, Personal communication. Við Streym, S., et al*. Eur. J. Clin. Nutr.* 2013, *67*, 1022-1028), maternal 25(OH)D at 30 and 50 nmol/L would reasonably lead to a detection of cord 25(OH)D at ~25 and 40 nmol/L, respectively. Cord serum 25(OH)D concentrations were not associated with being in the highest quartiles for either FMI or FFMI. Infants whose mother’s 25(OH)D was <30 nmol/L at 15 weeks gestation were less likely to be above the 80th percentile for FFM (kg) at birth (OR 0.475 (95% CI; 0.237, 0.954), *p* = 0.036) or in the highest quartile for FFMI (OR 0.614 (95% CI; 0.372, 1.011), *p* = 0.055) compared to those with maternal concentrations ≥50 nmol/L. Similarly, maternal 25(OH)D < 50 nmol/L reduced the odds ratio of an infant being in the highest quartile for FMI at birth (OR 0.686 (95% CI; 0.492, 0.950), *p* = 0.026).

Conclusions: This study has found that low 25(OH)D in early pregnancy reduced the odds of offspring being in the upper end of the distribution for both FMI and FFMI at birth. We will continue to examine links between vitamin D status throughout early childhood and body composition.

### 2.29. Vitamin D Status of Māori and Non-Māori New Zealanders of Advanced Age

#### Bacon, C.J.; Kerse, N.; Moyes, S.; Teh, R.; Hayman, K.J.; Dyall, L.; Kepa, M.

Background: Possible consequences of low vitamin D status include osteoporosis, falls, cardiovascular disease, cancer, and diabetes, all of which are likely to be more serious for men and women of advanced age (>80 years) compared to younger adults. The purpose of this study was to assess vitamin D status and its cross-sectional determinants in this vulnerable population.

Methods: Levels of 25-hydroxy vitamin D (25(OH)D), creatinine, and parathyroid hormone (PTH) were measured at enrolment in 566 Māori (aged 80–90 years) and non-Māori (aged 85 years) men and women living within defined regional boundaries of the Bay of Plenty and Lakes District of New Zealand. Individual 25(OH)D levels were adjusted for the day of year of measurement using annualised sine curve relationships generated for demographic subgroups. Habitual physical activity and health-related quality of life (HRQoL) were assessed via interview, during which use of vitamin D-containing supplements or medications was checked and recorded. Body composition indices and grip-strength were also measured.

Results: Levels of 25(OH)D were 69 ± 30 nmol/L (mean ± SD), with 15% of individuals >100 nmol/L and 6 individuals >150 nmol/L. Levels in Māori (59 ± 26 nmol/L) were lower than those in non-Māori (75 ± 30 nmol/L; *p* < 0.001), and this difference was maintained when 25(OH)D levels were adjusted for measurement day-of-year. Gender differences only became apparent after this seasonal adjustment [males 77(55–93); females 64(46–89) nmol/L; *p* < 0.001]. Vitamin D supplementation was reported by 98 (18% of) participants: including a greater proportion of women (24%) than men (11%; *p* < 0.001), and of non-Māori (24%) than Māori (7%; *p* < 0.001). Of those taking vitamin D, 49% took high oral doses ≥25 µg (1000 IU) vitamin D daily equivalent, and 5 individuals took >50 µg (2000 IU) equivalent daily dose. Levels of 25(OH)D were lowest in the highest quintile of socio-economic deprivation (*p* = 0.03 for ANOVA) but this effect disappeared when ethnicity (dichotomised as Māori *vs.* non-Māori) was included in the model. Similarly, 25(OH)D levels were inversely correlated with PTH (*r* = −0.3; *p* < 0.001), percent body fat (*r* = −0.1; *p* = 0.02), body mass index (*r* = −0.2; *p* < 0.001), and physical HRQoL (*r* = −0.09; *p* = 0.04), with addition of creatinine additionally positively correlated (*r* = 0.09; *p* = 0.04) and physical HRQoL excluded for seasonally-adjusted levels. When regression models included ethnicity, only PTH (−ve), creatinine (+ve) and ethnicity itself (Māori ethnicity −ve), were included as predictors of seasonally-adjusted 25(OH)D levels (*r*^2^ = 0.15 for model), with an addition of grip-strength (−ve) when levels were non-adjusted (*r*^2^ = 0.15 for model).

Conclusions: Frequent use of high-dose oral vitamin D appears to have resulted in a high overall vitamin D status in older New Zealanders, which is in stark contrast to the relatively low status reported previously. Poorer vitamin D status in Māori may reflect darker skin tone or inequitable supply of oral vitamin D. Primary care practitioners should be aware of the availability and common use of high-dose vitamin D preparations, and should ask about over-the-counter supplement use before prescribing pharmaceutical vitamin D in those of advanced aged.

### 2.30. Feasibility and Acceptability of Assessing Vitamin D Status in Primary Schoolchildren Using a Finger-Prick Dried Capillary Blood Spot Method

#### Griffiths, C.J.; Wood, H.E.; Cross, L.; Smithers, H.; Kalsi, H.; Walton, R.; Layla, A.; Bremner, S.; Dundas, I.; Grieve, A.; Grigg, J.; Jamaludin, J.; Kelly, F.J.; Lee, T.; Marlin, N.; Mudway, I.S.; Sheikh, A.; Martineau, A.R.

Background: Vitamin D status of inner city children may influence their respiratory health, but performing venesection in large numbers of children is challenging, especially among young children.

Methods: As part of a study examining the impact of the introduction of the London Low Emission Zone on children’s respiratory health, we assessed the feasibility and acceptability of a finger prick dried blood spot method for assessing vitamin D status (Pro-Diagnostics, London, UK).

Results: Of 320 Year 4 children (aged 8–9 years) at 9 schools visited in November–December 2013, 195 (61%) participated in the health assessment and 88 of those (45%) had the finger prick test. The parents of 32 children did not give permission for the finger prick test; 90 children were not tested for a variety of reasons, including 15 from one school which asked us not to offer the finger prick test. Mean age was closely similar between those who had the finger prick test and those who did not (8.8 ± 0.3 years and 8.7 ± 0.3 years, respectively (±SD)). There was one instance of a child feeling faint after the test; otherwise there were no adverse events. Children were much more enthusiastic about having the finger prick test than we expected, and parents were for the most part willing to give their consent (84% of all participants). At the time of writing, samples had been analysed for 68 children (33 boys, 35 girls). The mean 25(OH) vitamin D was 65.7 ± 35.1 nmol/L, with a range of 15.8–140.0 nmol/L, which is within the range of published values. 29 children had a 25(OH) vitamin D level defined as deficient or insufficient (*i.e.*, <50 nmol/L).

Conclusions: A finger-prick dried blood spot method is feasible and acceptable way of assessing the vitamin D status of primary schoolchildren. In our experience, when conducted as part of a suite of tests, a dedicated member of staff was required to do the finger prick test as it was quite time-consuming.

### 2.31. Performance Evaluation of 25-Hydroxy Vitamin D Assays with Chronic Kidney Disease Patients

#### Vogl, C.; Roth, H.J.

Background: Vitamin D is a key player in regulating bone and mineral homeostasis. The highly regulated interplay of metabolism and catabolism of Vitamin D is disrupted in patients with chronic kidney disease (CKD) resulting in a decreased concentration of 1,25(OH)_2_D. Due to renal dysfunction and altered Vitamin D metabolism, the serum matrix and metabolite composition of this patient cohort differ from the healthy, which represents a challenge in the accurate determination of their vitamin D status. International guidelines such as KDIGO and KDOQI suggest measuring 25(OH)D levels in patients with impaired kidney function from stage 3 and correcting suboptimal Vitamin D status with the same treatment strategies recommended for the general population. Four different automated 25(OH)D assays were evaluated using samples from CKD patients at stages 3–5 and on dialysis for their comparability to LC-MS/MS and differences between methods.

Methods: The population in this investigation consisted of the following three groups: CKD3-5 (68 patients, not on dialysis), dialysis (108 patients) and healthy controls (99 apparently healthy individuals). All samples were tested by LC-MS/MS and by four automated 25(OH)D total assays: Abbott Architect, Diasorin Liaison, Roche Elecsys and Siemens Centaur. Method comparisons were assessed using Pearson’s coefficient of correlation (r) as determined by Passing-Bablok regression analysis. Subjects in each cohort were classified as having optimal or suboptimal vitamin D status based on a cut-off of 30 ng/mL as recommended by many experts. This classification was also used to assess the different assays performance in comparison with LC-MS/MS. The standardization of the Roche Elecsys assay was verified by using the serum reference panel “Ref!25OHD” from Bioclin Oy—Labquality, which contains 20 serum samples from single donors certified by reference measurement procedure (RMP) based on isotope dilution-liquid chromatography/tandem mass spectrometry (ID-LC-MS/MS, Prof. Linda Thienpont, University of Ghent).

Results: The traceability of Elecsys Vitamin D total (Roche) to NIST and a higher order RMP was confirmed by the serum reference panel “25OHD”. The difference plot of the Elecsys assay against the reference values showed a mean bias (3.9%) that was constant across the measuring range. Applying Passing Bablok regression slope, intercept for the three groups are as followed:

(a) Healthy: Elecsys (1.17; −3.63), Abbott (0.899; 2.90), Siemens (0.698; 1.82), Diasorin (0.641; 0.567)
(b) CKD3-5: Elecsys (0.959; −3.4), Abbott (0.72; 4.11), Siemens (0.725; 2.2), Diasorin (0.584; 1.28)
(c) Dialysis: Elecsys (0.722; −1.23), Abbott (0.508; 5.31), Siemens (0.660; 3.17), Diasorin (0.528; 1.26)

LC-MS/MS classified as having optimal 25(OH)D status 28% of healthy subjects, 41% of CKD3-5 and 36% of dialysis patients. The percentages obtained in the three cohorts respectively by the automated methods were: Elecsys 25%–26%–13%; Architect 25%–25%–8%, Centaur 6%–18%–14% and Liaison 5%–4%–6%.

Conclusions: A high variation between the methods was observed. Compared to LC-MS/MS, the Centaur and the Liaison assays show a high overall under-recovery of both CKD patients and healthy controls, leading to a different classification based on the cut-off used (30 ng/mL). The Elecsys assay shows overall good comparability to higher order RMP and routine LC-MS/MS and the best performance among automated assays in the CKD patient population.

### 2.32. Effects of Osteoporosis-Inducing Drugs on Vitamin D-Metabolizing Enzymes in Osteoblast-Like Cells

#### Wegler, C.; Wikvall, K.; Norlin, M.

Background: Glucocorticoids and anti-retroviral drugs used in HIV-treatment have been associated with an increased risk of osteoporosis and decreased vitamin D_3_ levels. Vitamin D is activated in two hydroxylation steps, firstly into the main metabolite in blood circulation, 25-OH-vitamin D_3_, by CYP2R1 and CYP27A1. The second hydroxylation step, performed by CYP27B1, forms the active metabolite, 1,25-(OH)2-vitamin D_3_. CYP24A1 regulates the levels of vitamin D_3_ by inactivating 25-OH-vitamin D_3_ and 1,25-(OH)2-vitamin D_3_. Vitamin D_3_ is mainly metabolized in the liver and kidney. However, 25-OH-vitamin D_3_ can also be metabolized into 1,25-(OH)2-vitamin D_3_ in the bone. Serum levels of 1,25-(OH)2-vitamin D_3_ regulate the expression of CYP24A1 in the kidney. CYP24A1 in bone has been reported to be unaffected by changes in serum levels of 1,25-(OH)2-vitamin D_3_. This finding suggests that the locally produced metabolite affects the expression levels of the enzyme in bone. 25-OH-vitamin D_3_ and 1,25-(OH)2-vitamin D_3_ also regulate proliferation and mineralization in osteoblasts. The aim of the study was to investigate the effect of osteoporosis inducing drugs on vitamin D_3_-metabolizing enzymes in bone cells in an effort to investigate causes of drug-induced osteoporosis.

Methods: The human osteoblast-like cell line MG-63 was cultured and treated with the glucocorticoid, dexamethasone, and the anti-retroviral drug, efavirenz, respectively with and without vitamin D_3_ and its metabolites. mRNA was extracted and relative mRNA expression of vitamin D metabolizing enzymes were investigated using real time-PCR.

Results: Treatment with vitamin D_3_ slightly increased CYP24A1 mRNA expression while the 25- and 1,25-hydroxylated metabolites significantly increased the mRNA expression. When co-treated with efavirenz and dexamethasone respectively, the 1,25-(OH)2-vitamin D_3_-mediated induction was abrogated. CYP27B1 mRNA expression was significantly increased only by 25-(OH)-vitamin D_3_, an effect that also was counteracted by co-treatment of efavirenz. Interestingly, CYP27B1 mRNA expression was significantly decreased by 1,25-(OH)2-vitamin D_3_, which might be a possible effect of autoregulation similarly as known in the kidney.

Conclusions: The effect on the CYP24A1 mRNA expression, mediated by 1,25-(OH)2-vitamin D_3_, was abrogated by co-treatment with efavirenz and dexamethasone respectively. This suggest that the local regulation of vitamin D_3_ in bone cells is disturbed by the antiretroviral drug and glucocorticoid, which might be a contributing factor to the drug-induced osteoporosis observed in clinical studies.

### 2.33. Vitamin D Standardization Program (VDSP)

#### Sempos, C.T.

Background: Laboratory measurement of serum 25-hydroxy vitamin D [25(OH)D] concentration is used to assess an individual’s vitamin D status. However, there is substantial assay variation in 25(OH)D within and among the different assays whether commercially available or developed by individual laboratories. Such assay variation confounds clinical assessment and it makes the pooling of research results from clinical research, epidemiological studies and clinical trials difficult if not impossible. 25(OH)D assay standardization is, therefore, essential to the development of evidenced-based clinical and public health guidelines for vitamin D.

Methods: Vitamin D Standardization Program (VDSP) is an international program designed to promote the standardized laboratory measurement of 25(OH)D. It was established in 2010 by the US NIH Office of Dietary Supplements in collaboration with US National Institute of Standards and Technology (NIST), CDC, and Ghent University. The objectives of the VDSP are to: (1) Develop a reference measurement system (RMS) to standardize routine assays to the reference measurement procedures (RMP); (2) Evaluate the commutability of serum materials used to standardize assays; and (3) Conduct an international research program devoted to improving the laboratory measurement of 25(OH)D and to documenting and studying differences in standardized 25(OH)D concentrations among national surveys worldwide. The objectives are meant to promote the standardized 25(OH)D measurement by commercial & laboratory-based assays.

Results: VDSP developed an RMS consisting of NIST and Ghent University RMPs, NIST standard reference materials (SRM 972a), CDC’s Vitamin D Standardization Certification Program, DEQAS accuracy-based scheme and study designs for standardizing 25(OH)D results measured in the past. This RMS is being used to standardize past, current, and future measurements of 25(OH)D. In this context, standardization amounts to calibrating routine assay 25(OH)D values to values from the NIST and Ghent RMPs or to the true concentration of 25(OH)D. Performance criteria for routine laboratories, *i.e.*, CV £ 10% and Bias £ 5%, were established using biological criteria. Commutability of NIST SRM 972a, and CAP and DEQAS materials was evaluated and those materials were found to be commutable. Eight national surveys from Australia, Canada, Germany, Ireland, Mexico, Korea, UK and USA are participating in an effort to characterize vitamin D status around the world. Using data from the Irish National Adult Nutrition Survey (NANS), a case study was conducted to evaluate VDSP methods for standardizing 25(OH)D values measured in the past. In it estimated standardized distribution of a systematically selected sample of 100 serum samples using the VDSP algorithm was compared with re-measuring all 1118 samples using an assay traceable to NIST and Ghent RMPs. The prevalence of Irish adults with a 25(OH)D concentration <30 nmol/L using the original immunoassay, the VDSP algorithm and re-measuring all samples was 6.5%, 11.4% and 11.2%, respectively.

Conclusions: The VDSP has developed an effective RMS to standardize the laboratory measurement of 25(OH)D. Commercial assay manufacturers are collaborating with the VDSP. Methods to standardize 25(OH)D data collected in the past have been demonstrated to work. This is a multi-year effort, which needs to the support of vitamin D researchers around the world.

### 2.34. Vitamin D and Incidence of Mortality, Morbidity, and Growth Failure Among a Prospective Cohort of HIV-Infected and HIV-Exposed Tanzanian Infants

#### Sudfeld, C.R.; Duggan, C.; Aboud, S.; Kupka, R.; Manji, K.P.; Kisenge, R.; Fawzi, W.W.

Background: Vitamin D is known to be a potent immunomodulator, but its role in mortality and morbidity among infants remains unclear.

Methods: Serum 25-hydroxy vitamin D (25(OH)D) was quantified by HPLC-MS/MS at 5–7 weeks of age in 253 HIV-infected and 948 HIV-exposed (uninfected) infants enrolled in a randomized trial of multivitamins (not including vitamin D) conducted in Tanzania during 2004–2007. Infants were followed at monthly clinic visits for a total of 24 months. Physicians performed a clinical exam every 3 months or when an illness was noted. Proportional hazard models were used to analyze binomial mortality and growth outcomes. Mixed effects models were used to assess anthropometry continuously, while poisson regression analyzed physician diagnosis of morbidities.

Results: Mean ± SD of serum 25(OH)D concentration at 5–7 weeks of age was 18.6 ± 10.3 and 18.1 ± 9.2 ng/mL for HIV-infected and HIV-exposed uninfected infants, respectively. The association of 25(OH)D with mortality appeared to be U-shaped for both HIV-infected and HIV-exposed infants. After multivariate adjustment, 25(OH)D ≥ 30 ng/mL was significantly associated with increased mortality as compared to the 25(OH)D ≥ 20 and < 30 ng/mL reference category for HIV-infected (HR: 2.47; 95% CI: 1.13–5.44; *p* = 0.02) and HIV-exposed infants (HR: 4.00; 95% CI: 1.67–9.58; *p* < 0.01). 25(OH)D levels <10 ng/mL also appeared to be associated with increased risk of mortality as compared to the reference, but multivariate results were not statistically significant for HIV-infected (HR: 1.43; 95% CI: 0.74–2.78; *p* = 0.29) and HIV-exposed infants (HR: 1.56; 95% CI: 0.60–4.03; *p* = 0.36). As for morbidities among HIV-exposed infants, 25(OH)D ≥ 30 ng/mL was significantly associated with increased physician diagnosis of clinical (IRR: 1.34; 95% CI: 1.06–1.70; *p* = 0.02) and confirmed malaria (IRR: 1.71; 95% CI: 1.15–2.54; *p* < 0.01), after multivariate adjustment. 25(OH)D < 10 ng/mL was associated with increased risk of oral candidiasis diagnosis (*p* = 0.046). There was no association of 25(OH)D with physician diagnosis of ALRI or maternal report of respiratory morbidity (*p* > 0.05). Among HIV-exposed infants, baseline 25(OH)D < 10 ng/mL was significantly associated with incidence of wasting (weight-for-length *z*-score [WLZ] <−2) as compared to 25(OH)D ≥ 20 and < 30 ng/mL (HR: 1.71; 95% CI: 1.20–2.43; *p* < 0.01). In continuous analysis of WLZ, infants with 25(OH)D < 10 ng/mL exhibited a rapid decrease in WLZ from 10–38 weeks of age, but then experienced catch-up with comparable WLZ by 110 weeks. There was no association of 25(OH)D with length-for-age and weight-for age *z*-score trajectories nor incidence of stunting or underweight. There was no significant evidence of effect modification by exclusive breastfeeding duration, maternal or child use of antiretrovirals, maternal wasting, or randomized multivitamin regimen for any analysis.

Conclusion: There appears to be a delicate balance of 25(OH)D with morbidity, growth failure, and mortality for infants of HIV-infected mothers in this setting. Randomized controlled trials of vitamin D supplementation may be warranted for infants with low 25(OH)D during the initial months of life, but careful selection of the regimen with close monitoring of safety for those who reach high 25(OH)D levels will be necessary.

### 2.35. Vitamin D Metabolites Reduce Rhinovirus Infection in a Respiratory Epithelial Cell Line

#### Greiller, C.L.; Suri, R.; Grigg, J.; Johnston, S.L.; Martineau, A.R.

Background: Vitamin D has been shown to have various immunomodulatory effects, with deficiency seen to be associated with risk of respiratory infection, and supplementation suggested to prevent respiratory tract infections. The respiratory epithelium is vital in the defence against such infections, and thus may be an important focus as to where vitamin D has its immunomodulatory actions. As well as its function as a physical barrier to viruses and bacteria, the respiratory epithelium can initiate innate and adaptive immune responses, and provides the receptors (such as Intercellular Adhesion Molecule (ICAM)-1) required for viral and bacterial attachment and cellular entry).

Methods: A549 cells were cultured and mono-layers were pre-treated with 25-hydroxy vitamin D (25(OH)D), 1,25-dihydroxy vitamin D (1,25(OH)_2_D) or vehicle (0.1% ethanol) at a concentration of 10^−7^ M for 48 h. Cells were then stimulated with media, rhinovirus-16 (MOI 1), filtered RV-16 or UV-inactivated RV-16 for 6 h. Supernatants were collected for analysis by multiplex ELISA, and cells were lysed for mRNA analysis by RT-PCR.

Results: Levels of viral mRNA were reduced 3-fold in the presence of 25(OH)D and 1.5-fold in the presence of 1,25(OH)_2_D when compared to ethanol control (*p* = 0.0004 and 0.008 respectively). ICAM-1 mRNA was increased 60-fold following RV-16 stimulation (*p* = 0.005), with RV-induced ICAM-1 expression attenuated following co-incubation with 25(OH)D and 1,25(OH)_2_D (*p* = 0.004 and 0.02 respectively). IκBα mRNA was increased by 25(OH)D and 1,25(OH)_2_D in the presence of RV-16 infection, both by 2-fold, although statistical significance was not attained. Both LL-37 and IFN-α expression were inhibited by RV-16 (*p* = 0.006 and 0.03), with co-incubation of A549 cells with 1,25(OH)_2_D increasing LL-37 3-fold (*p* = 0.02) and with 25(OH)D increasing IFN-α 2-fold (*p* = 0.003). Finally, RV-16 infection induced a 2.5-fold increase in PafR expression (*p* = 0.004) which was attenuated by 25(OH)D and 1,25(OH)_2_D (both 5-fold, *p* = 0.001 for both).

Conclusions: We show for the first time that co-culture with physiological concentrations of 25(OH)D, and a positive control of 1,25(OH)_2_D, renders the respiratory epithelium resistant to rhinovirus infection. This effect was associated with attenuation of RV-induced ICAM-1 expression and a trend towards an increase in IκBα expression, raising the possibility that inhibition of NF-κB activity may be a mechanism by which 25(OH)D and 1,25(OH)_2_D exert this action. Additionally, 25(OH)D and 1,25(OH)_2_D were demonstrated to increase the anti-viral agents IFN-α and LL-37, and decrease the bacterial receptor PafR, providing potential beneficial effects both by enhancing the immune response to viral respiratory pathogens and by preventing the occurrence of secondary bacterial infections.

### 2.36. Placental Amino Acid Transport may be Regulated by Maternal Vitamin D and Vitamin D-Binding Protein: Results from the Southampton Women’s Survey

#### Simner, C.L.; Barton, S.J.; Lillycrop, K.A.; Inskip, H.M.; Cooper, C.; Hanson, M.A.; Godfrey, K.M.; Harvey, N.C.; Lewis, R.M.; Cleal, J.K.; the SWS Study Group

Background: Facilitated transporters, accumulative transporters and amino acid exchangers mediate transfer of amino acids across the placental syncytiotrophoblast into the fetal circulation, and the mRNA levels of several amino acid transporters relate positively to measures of fetal growth. These transporters contain putative vitamin D receptor response elements within their promoters, suggesting that amino acid transporters may be modulated by maternal vitamin D status, a measure previously shown to relate to adiposity and bone health of the offspring. We therefore aimed to establish whether maternal vitamin D and vitamin D-binding protein (DBP) levels might relate to the expression of amino acid transporters in human placenta.

Methods: We used data and samples from the Southampton Women’s Survey, a cohort of study of 3159 pregnancies with information collected from the mothers before conception. With informed consent and ethical approval maternal serum 25-hydroxy vitamin D [25(OH)D] and DBP levels were measured at 34 weeks gestation by radioimmunoassay and placental samples were collected within 30 min of delivery. A subset of tissue samples (*n* = 91) were used for this analysis; selected based on availability of neonatal DXA data and maternal serum measures. Quantitative real-time PCR was used to measure amino acid transporter mRNA expression; all normalized to appropriate housekeeping genes. Pearson’s correlation (rp) was used to explore the relationship between maternal 25(OH)D (*n* = 91) and DBP (*n* = 85) concentrations and placental amino acid transporter mRNA.

Results: Maternal 25(OH)D levels correlated positively with mRNA expression of the amino acid exchangers ASCT1 (rp = 0.23, *p* = 0.029) and y + LAT1 (rp = 0.32, *p* = 0.002) and the facilitated transporter LAT3 (rp = 0.31, *p* = 0.003). DBP levels correlated positively with mRNA expression of the facilitated transporters LAT3 (rp = 0.22, *p* = 0.04), LAT4 (rp = 0.28, *p* = 0.01) and TAT1 (rp = 0.20, *p* = 0.06) as well as the exchanger y + LAT2 (rp = 0.23, *p* = 0.033).

Conclusion: These results suggest that maternal 25(OH)D and DBP might regulate the expression of placental amino acid transporters and potentially influence the transfer of amino acids to the fetus. The correlations between DBP levels and several amino acid transporters suggest that vitamin D delivery may be important for placental function, but further work is now required to establish whether these associations are causal.

### 2.37. Knowledge of Vitamin D on the Postnatal Wards- D-Disastrous or D-Lightful?

#### Wood, C.L.; Embleton, N.D.

Ackground: In the UK, over 50% of whites and 90% of South Asians have insufficient vitamin D levels. Vitamin D is essential for calcium homeostasis and deficiency is most likely during rapid growth periods, during pregnancy and while breastfeeding. Newborn status is largely determined by maternal status. Exclusively breastfed infants are at special risk as breastmilk may not meet requirements, especially after 6 months. There is wide variation in the content and availability of vitamin D supplements, and despite NICE guidelines on vitamin D supplementation implementation remains uncertain.

Methods: We conducted an audit to determine the level of information given to pregnant and breastfeeding women regarding vitamin D and to gauge the knowledge of midwifery and medical staff on postnatal wards.

The audit was based on two standards: (1) Antenatal standard NICE 62- At booking women should be given information and advice on the importance of taking a 10 microgram vitamin D supplement per day during pregnancy and whilst breastfeeding. Health professionals should ensure that women at greatest risk of vitamin D deficiency are asked about vitamin D supplementation; (2) NICE public health guideline 11- All infants and young children aged 6 months to 5 years should take a daily supplement containing vitamin D.

We surveyed 20 mothers and 15 health professionals on the postnatal wards. The staff were asked whether they knew why vitamin D is important, who should be offered vitamin D supplementation and at what dose. The patients were asked similar questions to ascertain their knowledge of vitamin D and the level of information they had been given, whether they were taking or plan to take supplements, whether any other young children in their family take supplements and their at-risk status.

Results: Postnatal mothers: Less than half the women surveyed were given information after vitamin D supplementation during pregnancy. Only 65% women took supplements during pregnancy. No-one had been given information and advice about vitamin D supplements whilst breastfeeding and only 20% breastfeeding mothers were planning to start supplements (all as part of Healthy start or pregnancy multivitamin). A further 10% said that they would if they knew the dose to take. Only 40% women could give a reason why vitamin D supplements were important- all of them had taken supplements. 15% (3/20) women surveyed said they had risk factors for vitamin D deficiency, but only 2 were specifically asked about vitamin D deficiency. None of their other children took supplements. Staff: Only 67% staff were able to answer why vitamin D was important. No one could correctly highlight all the at risk groups from a list provided. Only 7% knew which children should be offered supplementation. Only one-third of staff identified (from options given) the correct dose of vitamin D supplement for high-risk women.

Conclusion: Current practice is better at fulfilling antenatal than postnatal standards (although health visitors may partly fulfill this role after discharge) and there is much scope for improvement. Knowledge levels appear to correlate with use of vitamin D supplementation, suggesting that appropriate information provision is vital. Education for midwives and doctors must also be a priority as we have demonstrated significant knowledge gaps. We plan to provide an NHS Choices leaflet in the booking packs and provide training to staff before re-auditing.

### 2.38. The Impact of Vitamin D Supplementation on Muscle Strength and Function in Young People: A Systematic Review

#### Wood, C.L.; Pearce, S.H.; Cheetham, T.D.

Background: The role of vitamin D in bone metabolism is well known but its’ actions on muscle are incompletely understood. Clinical and laboratory evidence suggest that muscle is a target tissue for vitamin D, with myopathy a recognised feature of D deficient individuals and receptors present in skeletal muscle. Most data regarding the effects of vitamin D on non-skeletal tissues in young people has been obtained from cross-sectional studies, with their associated limitations.We have therefore undertaken a systematic review of randomised controlled trials (RCTs) that has examined the impact of vitamin D on muscle strength and function in young people.

Methods: The review was carried out according to Centre for Reviews and Dissemination guidelines. Trials were identified from an electronic search of Medline, Embase, Pubmed and the Cochrane Central Register of Controlled Trials. Review articles, reference lists and related citations were also hand-searched. To qualify for inclusion, studies had to be RCTs, carried out in children or young people aged between 1 month and 20 years of age from 1946 to December 2013 and include components of muscle strength and/or function as an outcome measure. The search terms used were: Intervention- Vitamin D OR ergocalciferol OR cholecalciferol, Outcome- muscle OR musc*, Study design- random* controlled trial OR controlled.

Results: From 1758 records identified, 3 studies of 425 participants met the inclusion criteria (Goswami, R., *et al. JCEM*. 2012, *97*, 4709-4716. El-Hajj Fuleihan, G., et al*. JCEM*. 2006, *91*, 405-412. Ward, K.A., et al*. JCEM* 2010, *95*, 4643-4651). Most studies were excluded by abstract as they did not include young people. Baseline 25(OH)D levels were all ≤35 nmol/L in the included studies. Various therapeutic regimens were used to replenish vitamin D levels to ≥56 nmol/L by the end of the study. All 3 studies used grip strength as an outcome variable. In contrast to observational studies that show a beneficial effect of vitamin D on grip strength, there was no significant increase seen in grip strength in any of the included studies. Ward *et al.* also used jumping mechanography to demonstrate a 5% increase in the efficiency of movement in those who received vitamin D (Ward, K.A., et al. *JCEM* 2010, *95*, 4643-4651). They attributed this improvement to an increase in both jumping velocity and height, suggesting greater flexibility and muscular co-ordination after vitamin D supplementation.

Conclusions: This systematic review found that correcting vitamin D deficiency in young people did not result in improved muscle strength. Despite vitamin D deficiency primarily affecting proximal muscle function, the three studies that met the inclusion criteria all looked predominantly at distal muscle function (hand grip or pinch grip strength.) The results of this review are at odds with findings from some observational studies, which have suggested that vitamin D status affects hand-grip strength. Well-designed RCTs that include assessments of proximal muscle function are needed to clearly determine the effect of vitamin D on muscle function in young people.

### 2.39. The Spine and Vitamin D in Children with Sickle Cell Anaemia

#### Huang, C.; Michie, C.A.

Background: Back pain is relatively common in young patients suffering painful crises from sickle cell anaemia. In a proportion of these patients micro-infarctions in the vertebral body lead to changes that can be seen on standard radiographs: the vertebral central bodies collapse, leading to an H-type or fish-mouth appearance. The frequency, pattern and time-course of these bony changes is unclear, as is their relationship to other measures of bone health. Sickle cell patients frequently have low levels of vitamin D. How significant is this vitaminin maintaining vertebral structure?

Methods: A retrospective series of all radiographs from the paediatric sickle cell clinic at a single west London Hospital were reviewed visually for morphological changes in the vertebral column. Results were linked to biochemistry, haematology and vitamin D measures in the patients. 28 patients with HbSS received 40 radiographs or MRI studies of their chests or spines between 2010 and 2012. 4 patients with HbSC and HbSBthal had 10 radiographs in this time.

Results: No unusual vertebral shapes were found in any patient aged 12 years or younger in the clinic series. Two patients with HbSS developed “H-type” or “fish-mouth” vertebrae between 12 and 16 years of age. Both had multiple admissions with painful crises as adolescents, they had normal trans-cranial Doppler investigations. Both showed significant levels of intravascular haemolysis and vitamin D levels of <10 nmol/L were documented. 6 other patients had similarly deficient levels of vitamin D, but they did not have frequent admissions, or lower levels of haemolysis. They did not show visible changes in their vertebrae.

Conclusions: Vertebral bodies rarely show morphological changes in young children with sickle cell disease in this cohort. Those patients requiring frequent admissions for painful crises, together with vitamin D deficiency, can develop vertebral changes. However patients with low vitamin D but few painful crises had normal vertebral morphology. Such observations from a relatively small clinic suggest vitamin D deficiency acts as a comorbid factor in leading to changes in the vertebral bodies. Larger studies with measures such as bone densitometry, estimates of parathyroid hormone or glycoproteins related to bone remodeling such as osteopontin may help clarify this pattern.

### 2.40. Tooth Development in Children with Vitamin D Deficiency: The Rachitic Tooth in History

#### Foster, T.; Michie, C.A.

Background: The formation of tooth dentine and enamel is a mineralization process that biochemically closely resembles ossification in bone. To what degree does vitamin D influence dental health in children? OBJECTIVE: To ascertain whether vitamin D deficiency is linked to dental health.

Methods: Review of publications from the British Dental Association (BDA) and the Medical Research Council (MRC) who piloted research in this field between 1890 and 1950.

Results: Dental health deteriorated towards the end of the 19th century in Britain, alongside increasing urbanization. School inspections in the 1890’s suggested that the majority of children suffered caries by the age of 10–12. Approximately 6% of volunteer military recruits to the Boer war were rejected on the grounds of poor dental health. The phrase “Can’s bite, can’t fight” was employed. Higher rates of dental caries were linked with poverty and rickets. The government founded the Committee on Physical Deterioration in 1903; ten years later the first research grant was given to study rickets by the MRC. Studies carried out in a number of types of dogs by May Mellanby showed that vitamin D deficiency damaged the development of teeth at the same time as bones and that this could be reversed with a diet high in vitamin D. She conducted a series of early randomized controlled trials in orphanages too that showed lower rates of caries in children under 12 who were given diets higher in vitamin D. Recent research in children with specific syndromes and in gene knockout mice have confirmed a central role for vitamin D in tooth mineralization with effects on tooth structure, as described by May Mellanby. Vitamin D appears to have other significant effects on the ecological balance between a child’s gums and oral bacteria.

Conclusions: Early work with dietary manipulations in a dog model together with trials in institutionalized children suggested that vitamin D is an important protective factor against dental decay. These historical studies support the existence and concept of the rachitic tooth. Although dental caries rates are much improved today, caries persist in a proportion of the UK population according to BDA surveys. What is less clear is the relevant significance of vitamin D deficiency to tooth development, at a population level, in the UK at present.

### 2.41. Congenital Rickets due to Vitamin D Deficiency in the Mothers

#### Paterson, C.R.; Ayoub, D.

Background: Recent reviews have suggested that vitamin D deficiency rickets cannot begin to occur before birth (Kovacs, C.S. *Semin. Fetal. Neonatal. Med.* 2013, *18*, 129-135).

Methods: To explore this issue we have reviewed clinical reports of vitamin D deficiency in neonates from 1930 onwards. We found 25 cases with radiological and/or histological evidence of rickets identified within the first two weeks of life.

Results: Presentations of the infants included craniotabes, rachitic rosaries, enlargement of wrists, tetany and convulsions. In two cases rickets had been suggested from ante-natal X-rays. In four cases histological examination of deciduous teeth showed clear abnormalities. In six cases spontaneous fractures occurred in the first month of life. Of the 16 infants with serum calcium assays 15 had values lower than 2.2 mmol/L. Of 13 infants with serum alkaline phosphatase assays 12 had abnormally high levels. All the seven infants with assays for serum 25-hydroxy vitamin D had values lower than 25 nmol/L. There was evidence of maternal deficiency in 24 cases and in 16 of these the diagnosis of the rickets in the infants led to the identification of symptomatic osteomalacia in the mothers.

Conclusions: It is important to recognise that overt bone disease can be present at and before birth as a result of maternal deficiency.

### 2.42. Fractures in Rickets due to Vitamin D Deficiency

#### Paterson, C.R.

Background: Older authors from at least 1906 onwards were clear that vitamin D deficiency rickets could cause fractures. In one recent study (Perez-Rossello, J.M., et al*. Radiology* 2012, *262*, 234-241) the conclusion was that fractures in early childhood were unlikely to be caused by vitamin D deficiency.

Methods: To examine this view reports of fractures at less than two years of age in rickets due to vitamin D deficiency have been reviewed.

Results: 38 patients were identified in 21 publications between 1918 and 2011. Their ages ranged from birth to 23 months (median 5 months). There were at least 26 fractures of ribs, 13 of radii, 10 of femora, 10 of tibiae, 9 of ulnae, 6 of clavicles. 3 of fibulae, 2 of vertebrae and one of a humerus. There were multiple metaphyseal lesions, some of which could have represented true fractures. There was one skull fracture found soon after birth and many cases with skull abnormalities of uncertain nature. Most of the diaphyseal fractures were undisplaced, most were asymptomatic and most were apparently spontaneous. It was sometimes not possible to distinguish undisplaced transverse fractures from pseudofractures.

Conclusions: It is important to include vitamin D deficiency rickets in the differential diagnosis of fractures in young children.

### 2.43. Prevalence and Determinants of Vitamin D Deficiency in Patients with Chronic Obstructive Pulmonary Disease

#### Jolliffe, D.A.; James, W.Y.; Islam, K.; Timms, P.; Rowe, M.; Venton, T.; Mein, C.; Hoti, M.; Griffiths, C.J.; Martineau, A.R.

Background: Vitamin D deficiency has been reported to associate with susceptibility to upper respiratory infections in patients with chronic obstructive pulmonary disease (COPD) in the USA. Studies investigating the prevalence and determinants of vitamin D deficiency among COPD patients in the UK are lacking.

Methods: We conducted a cross-sectional study in 278 COPD patients aged 40–85 years who were screened for enrolment in a clinical trial of vitamin D supplementation conducted in London, UK. Participants completed a questionnaire detailing potential demographic and lifestyle determinants of vitamin D status, and gave a blood sample for analysis of serum 25-hydroxy vitamin D (25(OH)D) concentration and DNA extraction. Serum 25(OH)D concentration was determined by liquid chromatography—tandem mass spectrometry. Thirty-seven single nucleotide polymorphisms (SNPs) in 13 genes (DBP, DHCR7, CUBN, LRP2, CRTAM, LTA4H, CYP2R1, CYP3A4, CYP27A1, CYP27B1, CYP24A1, VDR, RXRA), involved with vitamin D transport, metabolism and signalling were typed using Taqman allelic discrimination assays. Logistic regression was used to identify environmental and genetic factors independently associated with risk of vitamin D deficiency defined as serum 25(OH)D concentration <50 nmol/L.

Results: Mean serum 25(OH)D concentration was 45.4 nmol/L (SD 25.3), and 171/278 (61.5%) participants were vitamin D deficient. The following demographic factors were found to independently associate with increased risk of vitamin D deficiency: BMI > 30 kg/m^2^ (OR 1.87, *p* = 0.04); blood draw during winter and spring seasons (OR 3.00, *p* < 0.01; OR 2.50, *p* < 0.01, respectively). The following lifestyle factors were found to independently associate with reduced risk of vitamin D deficiency: consumption of a vitamin D supplement, 100–400 IU/day (OR 0.42, *p* < 0.01); a sunny holiday abroad defined as: a trip to any location within a latitude 51 degrees North or South of the equator, during the local sunny season, 2 months prior to blood draw, for a duration of ≥1 week (OR 0.27, *p* = 0.02). None of the 37 SNPs investigated independently associated with vitamin D deficiency.

Conclusions: Vitamin D deficiency was highly prevalent among COPD patients screened for a clinical trial in London, UK. Obesity and sampling during winter and spring were risk factors for deficiency. Recent travel to a sunny country and consumption of vitamin D supplements were protective. Genetic variants in the vitamin D pathway that have previously been shown to associate with risk of vitamin D deficiency in healthy adult populations were not associated with deficiency in this patient group.

### 2.44. Concentrations of 25-Hydroxy Vitamin D in Umbilical Cord Blood and Achievement of Gross Motor Outcomes in Infants

#### McCarthy, E.K.; O’Donovan, S.M.; Murray, D.M.; Kenny, L.C.; Hourihane, J.O’B.; Kiely, M.

Background: Persistence of the influence of vitamin D status at birth into infancy has recently been shown (Við Streym, S., et al*. Eur. J. Clin. Nutr.* 2013, *67*, 1022-1028). Apart from its well-documented role in bone mineralisation, vitamin D is implicated in muscle strength and severe vitamin D deficiency can delay gross motor development, such as walking in children (Agarwal, A., et al*. Indian J. Pediatr.* 2009, *76*, 269-272). Associations between low circulating 25-hydroxy vitamin D (25(OH)D), the biomarker of vitamin D status, and muscle weakness in older adults (Bischoff-Ferrari, H.A., et al*. Am. J. Clin. Nutr.* 2004, *80*, 752-758) have been replicated in adolescents (Ward, K.A., et al*. J. Clin. Endocrinol. Metab.* 2009, 94, 559-563). Our aim was to explore associations between maternal and cord 25(OH)D concentrations and vitamin D supplementation during pregnancy and infancy and gross motor development in infants.

Methods: The Cork BASELINE Birth Cohort Study collected socio-demographic, dietary, anthropometric and supplement use data in maternal-infant dyads, from 15 weeks gestation. Blood from mothers at 15 weeks gestation and umbilical cords at delivery were processed to serum within 3 h and stored at −80 °C. Serum 25(OH)D concentrations in maternal-cord dyads (*n* = 1050) were quantified using liquid chromatography-tandem mass spectrometry (LC-MS/MS), using a method which is traceable to the NIST higher order reference measurement procedure (Sempos, C.T., et al*. Scand J. Clin. Lab. Invest. Suppl.* 2012, *243*, 32-40. Tai, S.S., et al. *Anal. Chem*. 2010, *82*, 1942-1948.). Fat mass (kg) and fat free mass (kg) were measured in 850 infants within 4 days of birth using air displacement plethysmography (PEA POD). Data on attainment of gross motor outcomes; sitting independently, crawling, standing with support and standing independently, in accordance with WHO milestones (World Health Organisation. *Acta paediatrica* 2010, *450*, 86-95), were collected. Factors associated with achievement of gross motor outcomes were explored using univariate analysis. Predictive models were developed using binary logistic regression.

Results: A subgroup of 379 term infants had complete data for this analysis, of whom 99% were Caucasian. Of the mothers in this subgroup 90% had a tertiary education, compared with 55% of the cohort overall. The main determinants of the ability to sit independently at 6 months, expressed as adjusted OR (95% CI), were a fat free mass at birth >80th centile (3.25 kg) (1.95 (1.17, 3.24), *p* = 0.011) and a maternal BMI at 15 weeks gestation >30 kg/m^2^ (0.50 (0.26, 0.98), *p* = 0.041). There were no associations between maternal serum 25(OH)D or maternal or infant vitamin D supplementation and any of the gross motor outcomes specified. On the basis of the correlation between maternal and cord 25(OH)D concentrations collected at time of delivery (Við Streym, S., et al*. Eur. J. Clin. Nutr.* 2013, *67*, 1022-1028. Kiely M. Personal communication), maternal 25(OH)D at 30 and 50 nmol/L would reasonably lead to a detection of cord 25(OH)D at ~25 and 40 nmol/L, respectively. Infants with cord serum 25(OH)D < 40 nmol/L were less likely to sit independently at 6 months ((0.64 (0.41, 1.01), *p* = 0.051).

Conclusions: The current data suggests a link between cord 25(OH)D, lean body mass and achievement of the first gross motor outcome which warrants further investigation as it may have implications for skeletal and muscle development in childhood.

### 2.45. Maternal Vitamin D Supplementation Reduces the Risk of Infection and Pre-Term Birth: A Population Study on 36,181 Pregnancies

#### Hyppönen, E.; Cavadino, A.; Williams, D.; Czeizel, E.

Background: Adequate vitamin D nutrition has been suggested to be essential for the successful maintenance of pregnancy, with increasing interest on its effects on placental function, infection risk and inflammatory responses. This study was done to evaluate the association between maternal vitamin D supplementation during pregnancy, the risk of bacterial and viral infections, and their possible contribution to the risk of pre-term birth.

Methods: We used information on 36,181 control pregnancies in the Hungarian Case-Control Surveillance on Congenital Abnormalities (HCCSCA) carried out from 1980 to 1996. Information on birth outcomes and maternal health during pregnancy, including bacterial and viral infections and the use of vitamin D supplementation was obtained from medical records, “maternity logbooks” filled in during visits to prenatal care clinics, or through a retrospective structured questionnaire sent to the mother after birth. Infections were restricted to those starting after the first note of supplement use, and all associations were adjusted for parity, maternal age, and social class.

Results: From the mothers, 24.9% took single vitamin D preparations (>3000 IU/week) with additional 4.7% taking multivitamins containing lower dosages of vitamin D (100–400 IU/day). There were 12,521 (34.6%) mothers with at least one recorded infection. Compared to mothers who did not take vitamin D supplements, risk of any infection was lower for mothers taking vitamin D as a single nutrient preparation (OR 0.28, 95% CI 0.27–0.30, *p* < 1.0e−250) and for those using multivitamin only (0.53, 0.52–0.55, *p* = 1.1e−18). Vitamin D supplementation was strongly associated with viral infections such as common cold *p* = 1.2e−57) and influenza (*p* < 0.1.3e−57 for both), but also with bacterial infections including vaginosis and bacteriuria (*p* < 2.1e−48 for both). Risk of premature birth was notably lower for mothers who took vitamin D supplementation during pregnancy (0.53, 0.43–0.66 for multivitamin; 0.44, 0.40–0.49 for single preparation), with the association remaining unaffected by adjustment for infections. However, a stronger reduction in the risk of pre-term birth by single preparation vitamin D was seen in pregnancies complicated with maternal infection (0.39, 0.29–0.52, *p* = 6.8e−12), compared to prematurity following an infection-free pregnancy (0.46, 0.41–0.52, *p* = 7.6e−39).

Conclusions: Maternal vitamin D supplementation during pregnancy was associated with notable reductions in the risk of infections and pre-term birth. These data suggest that the related effects are strong and consistent across different types of infections, highlighting the need to prevent vitamin D deficiency in pregnant mothers.

### 2.46. Association of Vitamin D and Serum Levels of Bone Turnover Markers in 10-Year Old Children. Results from the GINIplus&LISAplus Birth Cohorts

#### Thiering, E.; Brüske, I.; Kratzsch, J.; Hofbauer, L.C.; Heinrich, J.; for the GINIplus and LISAplus Study Groups

Background: Vitamin D deficiency is known to cause osteomalacia in adults and rickets in children. Epidemiological studies in adults showed that bone mineral density as well as bone turnover markers are associated with vitamin D levels. Moreover clinical practice guidelines emphasize the importance of vitamin D in bone health. However randomized controlled trials in children and adolescents did not show significant effects on total body bone mineral density after vitamin D supplementation. Therefore, we aimed to investigate the association between serum levels of 25(OH) vitamin D and bone turnover markers in a population-based sample of children with serum calcium levels.

Methods: 25(OH) vitamin D, calcium (Ca), osteocalcin (bone Gla protein, BGLAP), a specific marker of bone formation reflecting osteoblastic activity, and β-Crosslaps (β-CTx) a marker of bone resorption were measured in 2798 ten-year-old children from the German birth cohorts GINIplus and LISAplus. Vitamin D levels were corrected for date of measurement to normalize for seasonal variability using generalized additive models with thin plate regression splines as implemented in the statistical software R package “mgcv”. Linear regression was used to determine the association between bone turnover markers and vitamin D levels in serum.

Results: A 10 nmol/L increase in season-adjusted 25(OH) vitamin D was significantly associated with a 0.14 nmol/L increase (*p* = 0.01) in osteocalcin after adjustment for study, city, fasting status, daytime of blood draw, personal characteristics (age, sex, BMI, detectable sex hormones, total physical activity level) and socioeconomic factors (parental education, single parent status and income). For β-CTx alone no significant association with 25(OH) vitamin D was observed (−0.58 ng/L, *p* = 0.84), whereas the β-CTx to osteocalcin ratio was inversely associated with 25(OH) vitamin D levels (−1.1% change, *p* = 0.003). When stratifying the analyses by serum calcium levels, both associations were stronger in the strata with low (defined as smaller than the median 10.2 mg/dL) Ca levels (osteocalcin: 0.19, *p* = 0.02 (Ca low) *vs.* −0.02, *p* = 0.83 (Ca high); ratio β-CTx to osteocalcin −1.7%, *p* = 0.005 (Ca low) *vs.* −0.6%, *p* = 0.25 (Ca high)).

Conclusions: This study showed associations between markers of bone turnover and 25(OH) vitamin D which are dependent on serum calcium level in school-aged children. Higher levels of 25(OH) vitamin D were associated with slightly increased bone formation, and a decreased bone resorption to bone formation ratio.

### 2.47. Cross-Sectional Study on Different Characteristics of Physical Activity as Determinants of Vitamin D Status; Inadequate in Half of the Population

#### Van den Heuvel, E.G.H.M.; van Schoor, N.; de Jongh, R.T.; Visser, M.; Lips, P.

Background: Physical activity (PA) may have an impact on vitamin D status. The aim of the present study is to assess the contribution of different characteristics of PA (duration, intensity as estimated by energy expenditure, location) to vitamin D status.

Methods: The study was conducted in 1255 community-dwelling older men and women of the Longitudinal Aging Study Amsterdam (LASA). Cross-sectional relationships between PA and serum 25-hydroxy vitamin D (25(OH)D) concentrations were examined.

Results: Total PA, both indoor and outdoor PA, expressed in kcal/d was positively associated with 25(OH)D in women (*p* < 0.05) but not in men. The total time spent on these activities was not associated. As compared with the lowest tertile, both men and women in the highest tertile of cycling activity (≥6.4 min/day or 34.7 kcal/day) had a ~6 nmol/L higher 25(OH)D (*p* < 0.05). For men and women in the highest tertile of gardening (≥8.6 min/day or 87.6 kcal/day), these levels were 14.2 nmol/L (*p* < 0.001) and 5.8 nmol/L 25(OH)D (*p* < 0.05), respectively. Walking showed no association.

Conclusions: Daily time spent on total PA is often included when studying the association between sum of PA and 25(OH)D, while our study showed that energy expenditure might be a better unit. Individual types of outdoor PA with a high intensity, such as gardening and cycling, were associated with 25(OH)D.

### 2.48. Hypovitaminosis D and Its Relationship with Prevalent Cardiovascular Risk Factors among Saudi Postmenopausal Women

#### Alissa, E.M.; Ferns, G.

Background: Vitamin D deficiency is prevalent worldwide, and in Saudi Arabia in particular. In Saudi Arabia this is due to a combination of putative factors including: urbanization, demographic shifts, sedentary lifestyle, nutritional deficits, avoidance of sunlight because of the extreme heat, and decreases in the cutaneous production of vitamin D within an aging population. There is growing evidence that hypovitaminosis D is involved in the pathogenesis of cardiovascular diseases (CVD). Estrogen deficiency in the postmenopausal period is a risk factor for both CVD and osteoporosis.

Methods: We determined concentrations of serum 25 hydroxy-vitamin D in relation to several other metabolic biomarkers including total cholesterol, low-density lipoprotein cholesterol (LDL-C), high density lipoprotein-cholesterol (HDL-C), triglycerides, atherogenic index, glucose, C-reactive protein (CRP), measures of adiposity, and blood pressure values in a cross-sectional analysis in 180 Saudi postmenopausal women aged 48 to 88 years. These subjects were recruited from the Department of Internal Medicine at King Abdulaziz University Hospital. Participants completed a detailed questionnaire and fasting blood samples were collected. Comprehensive profiling of demographic, anthropometric, and biochemical variables was performed, together with assessment of serum 25 hydroxy vitamin D and CRP levels.

Results: Vitamin D deficiency, as assessed by serum 25 hydroxy vitamin D, was common, affecting 83% of individuals. Higher serum 25(OH) vitamin D levels were consistently found among subjects with no prevalent cardiovascular risk factors (*p* > 0.05) except for those subjects with serum CRP level ≥3 mg/L, TC/HDL-C ratio ≥5, and those with 4 or more pregnancies. Degree of hypovitaminosis D was inversely correlated with DBP (*r* = −0.172, *p* = 0.021), TC (*r* = −0.185, *p* = 0.013), LDL-C (*r* = −0.161, *p* = 0.031), and TC/HDL-C ratio (*r* = −0.147, *p* = 0.049) within the group of postmenopausal women. There appeared to be no significant impact of hypovitaminosis D on the inflammatory marker CRP, levels of which were similar among vitamin D sufficient and deficient subjects. 

Conclusion: However, hypovitaminosis D was significantly related to dyslipidemia, hypertension, and diabetes in a group of Saudi postmenopausal women. The precise nature of this association and the optimum levels of vitamin D for cardiovascular health remain to be elucidated.

### 2.49. National Surveillance Study of Hypocalcaemic Seizures Secondary to Vitamin D Deficiency in Children in the UK

#### Basatemur, E.; Sutcliffe, A.

Background: Reports suggest that vitamin D deficiency (VDD), and its clinical complications, have increased in prevalence among children in the UK. However, there is very limited epidemiological data available to quantify these concerns, existing studies being limited to single or multi-centre case series. This study provides the first national incidence estimates for a clinical manifestation of childhood VDD; hypocalcaemic seizures. We describe the characteristics and clinical outcomes of affected children.

Methods: Prospective 2-year national surveillance study across the UK and Ireland using the British Paediatric Surveillance Unit (BPSU) system. All Consultant Paediatricians were contacted monthly between September 2011 and September 2013. Case reporting criteria: any child < 16 years of age with a suspected seizure in the presence of a serum corrected calcium < 2.0 mmol/L and a serum 25-hydroxy vitamin D < 50 nmol/L. Exclusion criteria: previous hypocalcaemic seizure or an underlying condition causing secondary VDD (e.g., renal & liver disease). A number of reports had no 25-OH-D level available (usually due to an insufficient sample). These were classified as “probable” cases if they otherwise met the inclusion criteria and had at least one of the following; (1) high alkaline phosphatase; (2) high parathyroid hormone; (3) radiological features of rickets. BPSU reporting rates in 2012 averaged 93.3%. Population estimates from ONS (UK) and CSO (Republic of Ireland) were used to calculate incidence rates.

Results: Of 137 case notifications, 79 were confirmed cases, 10 probable cases, 32 reported in error or duplicates, 14 unconfirmed cases (details pending), and 2 lost to follow-up. 77 of the 89 confirmed and probable cases were infants (86.5%), with seven aged 1–2 years (7.9%) and five aged 11–15 years (5.6%). This equates to an incidence of 3.4 per million per year in children age 0–15 years, and 4.3 per 100,000 per year in infants.There was a male predominance of 82%. The majority of cases were from high-risk ethnic groups, with incidence estimates of 41.5 per 100,000 per year in Black infants and 37.4 per 100,000 per year in Asian infants. 61% had multiple seizures, and 20% had seizures lasting >10 min. 81% did not exhibit other clinical features of VDD, whilst 15% had clinical rickets. Median 25-OH-D = 11.2 nmol/L, ALP = 667 IU/L, PTH = 21.8 pmol/L, maternal 25-OH-D = 19 nmol/L. Mean serum calcium = 1.42 mmol/L. I.v. calcium gluconate was given in 45% and anti-convulsant medication in 26%, 48% did not receive any acute treatment. 12 cases (14%) received neither cholecalciferol nor ergocalciferol. Alfacalcidol was prescribed in 14% of cases. Mean length of admission was 3.9 days. There were no deaths, and only one child had sequelae on discharge; an extravasation burn from i.v. calcium gluconate.

Conclusions: Hypocalcaemic seizures secondary to VDD are a rare, but preventable, cause of morbidity in UK children. We confirm previous reports that VDD presents with hypocalcaemic symptoms in infancy and adolescence. We report an unexpected male predominance in cases, and hypothesize that differences in skeletal growth could increase susceptibility to hypocalcaemia in boys. A minority of cases did not receive the recommended type or dose of vitamin D treatment, suggesting a need to improve paediatricians’ knowledge regarding VDD management. Further studies are needed to investigate the epidemiology of rickets more broadly.

### 2.50. A Randomized, Double-Blinded, Parallel Study to Evaluate the Dose-Response of a Monthly Supplementation of 25,000, 50,000 or 100,000 IU of Vitamin D_3_ in Subjects with Deficiency in Vitamin D

#### Cavalier, E.; Jandrain, B.; Da Silva, S.; De Niet, S.; Vanderbist, F.; Scheen, A.

Background: Approximately one billion people worldwide have low serum levels of vitamin D. The aim of this study was to evaluate the dose-response effect in order to determine the level of supplementation of vitamin D required in patients with deficiency in vitamin D concentration (≤20 ng/mL).

Method: A randomized, parallel and double-blinded study was performed according to Good Clinical Practice in Belgium between December 2012 and May 2013. One hundred and fifty individuals (65 men and 85 female) aged over 18 years with a body mass index between 18 and 30 kg/m^2^ were selected because their vitamin D concentrations were between 5 ng/mL and 20 ng/mL at inclusion, consistent with vitamin D deficiency. The DiaSorin Liaison was used to measure serum levels of 25(OH)D (Stillwater, MN, USA). The selected subjects were randomized into 3 groups of 50, each to receive a low, moderate or high dose of D-CURE^®^ once a month. D-CURE^®^ consists of an ampoule containing an oily solution of 25,000 IU vitamin D for oral use. Three dosing schedules were compared: subjects received a loading dose of 50,000, 100,000 or 200,000 IU of vitamin D according to their group at Week 0, followed by 25,000, 50,000 or 100,000 at Week 4 and Week 8. The total study duration was 12 weeks. A total dose of 100,000, 200,000 and 400,000 IU, respectively, was therefore administered over 12 weeks. Drug administration was supervised by the study staff allowing a 100% patient’s compliance. Main endpoints were serum level of 25(OH), percentage of patients and time to reach the levels of 20 and 30 ng/mL over 12 weeks.

Results: Baseline levels of 25(OH)D were similar in the three groups (13.94 ± 3.75 ng/mL, 12.98 ± 3.85 ng/mL, 13.66 ± 3.57 ng/mL; *p* = 0.42). A rather linear dose-response relationship was observed between the 3 groups, with increases in 25(OH)D levels proportionate to the dose administered. Mean increases in 25(OH)D levels were 7.92 ± 0.87 ng/mL, 13.04 ± 0.87 ng/mL and 20.18 ± 0.86 ng/mL, respectively, for 100,000 IU, 200,000 IU and 400,000 IU after 12 weeks of supplementation. Overall, 98% of the subjects supplemented with 400,000 IU of vitamin D had their level of 25(OH)D above 20 ng/mL at Week 8, while 84% and 52% of patients receiving 200,000 IU or 100,000 IU reached that level after 12 weeks of treatment. Likewise, at week 12, 64% of individuals attained the target of 30 ng/mL in the 400,000 IU group compared to the lower dosage supplementation groups (24% and 4% for the 200,000 IU and 100,000 IU, respectively). No clinically relevant adverse events were observed after 3 months of supplementation, even with the higher doses. The individual maximum serum 25(OH)D concentration observed was 64 ng/mL which is far below 150 ng/mL, considered as the potentially “toxic” limit.

Conclusion: This study demonstrated a dose-response between vitamin D administration and plasma levels after monthly administrations of 25,000, 50,000 or 100,000 IU of vitamin D (D-CURE^®^ ampoule). Serum concentration of 25(OH)D > 20 ng/mL was obtained for all subjects (98%) in the group receiving the high dose of vitamin D only (100,000 IU per month). Starting the treatment with a high loading dose allows faster correction of the vitamin D deficiency.

### 2.51. Vitamin D in the General Population of Young Adults with Autism in the Faroe Islands

#### Kočovská, E.; Andorsdóttir, G.; Fernell, E.; Minnis, H.; Gillberg, C.

Background: Vitamin D deficiency has been proposed as a possible risk factor for developing autism spectrum disorder (ASD). Understanding gene-environment interaction in autism is currently a very important topic for research into early neurodevelopment. Given the genetic isolate character with specific environmental exposures, the Faroe Islands constitute an interesting environment in which to conduct epidemiological studies.

Methods: Design/Setting: 25-hydroxy vitamin D_3_ (25(OH)D_3_) levels were examined in a cross-sectional population-based study in the Faroe Islands. Effects of gender, age, month/season of birth, IQ, various subcategories of ASD and ADOS score were also investigated. Participants: The case group consisted of a total population cohort of 40 individuals with ASD (aged 15–24 years), and the control group included their 62 typically-developing siblings, their 77 parents, and 40 healthy age and gender matched comparisons.

Results: The ASD group had significantly lower 25(OH)D_3_ levels (24.8 nmol/L) than healthy comparisons (37.6 nmol/L, *p* = 0.002), and also lower than their siblings (42.6 nmol/L, *p* < 0.001) and parents (44.9 nmol/L, *p* < 0.001). Parents had the highest levels. Males in the ASD (*p* = 0.12) and sibling (*p* = 0.03) groups had lower 25(OH)D_3_ levels than females. There was no association between vitamin D and age, month/season of birth, IQ, subcategories of ASD, or ADOS score. Among the ASD group, 60% were severely deficient (<30 nmol/L) and 84.2% of the whole study sample (*n* = 219) had deficient/insufficient levels (<50/<75 nmol/L).

Conclusions: The present study, demonstrating an association between low levels of 25(OH)D_3_ and ASD, is the first to be based in a total population and to use siblings, parents and general population control groups. It adds to similar findings from other regions of the world, indicating vitamin D deficiency in the population and especially in individuals with ASD. As all groups were exposed to low levels of sunlight, the very low 25(OH)D_3_ in the ASD group suggests that some other underlying pathogenic mechanism may be involved. An important next step will be to replicate these findings in larger samples, possibly requiring international collaboration.

### 2.52. The Prediction of Vitamin D Deficiency by Simple Patient Characteristics

#### Sohl, E.; Heymans, M.W.; de Jongh, R.T.; den Heijer, M.; Visser, M.; Merlijn, T.; Lips, P.; van Schoor, N.M.

Background: Vitamin D status is currently diagnosed by measuring serum 25-hydroxy vitamin D (25(OH)D). This study aimed to develop a risk profile that can be used to easily identify older individuals at high risk for vitamin D deficiency.

Methods: This study was performed within the Longitudinal Aging Study Amsterdam, an ongoing cohort study of a representative sample of the Dutch older population (*n* = 1509 for the development sample and *n* = 1100 for the validation sample). Prediction models for serum 25(OH)D levels <50 nmol/L and <30 nmol/L were developed using backwards logistic regression. Risk scores were calculated by dividing the individual regression coefficients by the regression coefficient with the lowest beta in order to create simple scores.

Results: Serum 25(OH)D < 50 nmol/L and < 30 nmol/L was present in 46.2% and 17.5%, respectively. The model for the prediction of levels <50 nmol/L consisted of thirteen easily assessable predictors, whereas the model for levels <30 nmol/L contained ten predictors. The resulting Areas Under the Curve were 0.78 and 0.80, respectively. The AUC in the external validation data set was 0.71 for the model <50 nmol/L. At a cut-off point of 58 (range 8–97), the model predicted <50 nmol/L with a sensitivity of 61% and specificity of 82%, whereas these parameters were 61% and 84%, respectively, at a cut-off point of 110 (range 6–204) in the model for <30 nmol/L. Sensitivity and specificity differ by changing the cut-off points in total risk scores.

Conclusion: Two total risk scores, including thirteen or ten predictors that can easily be assessed, were developed and are able to predict serum 25(OH)D below 50 nmol/L and 30 nmol/L accurately. These risk scores may be useful in clinical practice to identify persons at risk for vitamin D deficiency.

### 2.53. The Association between Vitamin D Status and Quantitative Ultrasound at the Calcaneus Is Modified by Body Mass Index

#### Sohl, E.; de Jongh, R.T.; van Schoor, N.M.; Lips, P.

Background: Vitamin D is related to bone health. Studies found inconsistent results regarding the relationship of vitamin D status and bone mineral density (BMD) and Quantitative Ultrasound measurements (QUS). This study has two aims: (1) to examine the association between vitamin D status and QUS and BMD; and (2) to examine whether gender, age, physical activity or body mass index (BMI) modify these associations.

Methods: Data from three different cohorts were used: two cohorts of the Longitudinal Aging Study Amsterdam (LASA-I, 1995/1996, *n* = 1260; LASA-II, 2008/2009, *n* = 365) and the baseline measurement of the B-vitamins for the PRevention Of Osteoporotic Fractures (B-PROOF) study (2008–2011, *n* = 1319). QUS measurements (Broadband Ultrasound attenuation (BUA) and Speed of Sound (SOS)) were performed at the calcaneus in all three cohorts; BMD was measured at several sites of the body by DXA in B-PROOF. Multiple linear regression was used to assess whether vitamin D status was related to QUS and BMD and whether age (below *vs.* above the median age), gender, BMI (≤26.5 *vs.* >26.5 kg/m^2^) or physical activity (low *vs.* high) modified these relationships.

Results: The mean age of the participants was 75.5 (SD 6.5), 65.6(2.9), 73.5(6.3) years in LASA-I, LASA-II, and B-PROOF, respectively. Low vitamin D status (<25 nmol/L) was significantly associated with lower BUA as compared to the reference group (≥50 nmol/L); this association was modified by BMI (*p* < 0.1). In persons with low-to-normal BMI, individuals with serum 25(OH)D < 25 nmol/L had −7.7 (95% confidence interval-13.6; −0.5) lower BUA as compared to the reference group in LASA-I and this was −8.0 (−13.5; −2.6) for B-PROOF. In persons with normal-to-high BMI no significant associations were found. In LASA-II no associations of vitamin D status with BUA were found. Vitamin D status was not associated with BMD and SOS and these relationships were not significantly modified by sex, age, physical activity or BMI.

Conclusion: The association between vitamin D status and BUA was modified by BMI in the older cohorts, with the strongest effect in person with low-to-normal BMI (<26.5 kg/m^2^). No significant associations of vitamin D status and SOS and BMD were found.

### 2.54. Adjusted Calcium Concentration, Phosphate Concentration, Alkaline Phosphatase Activity and Parathyroid Hormone Concentrations in Plasma Are not Useful in Predicting Vitamin D Status in Children

#### Ivison, F.M.; Mughal, Z.M.; Berry, J.L.; Chaloner, C.

Background: The incidence of both symptomatic and asymptomatic vitamin D deficiency is growing, especially among certain ethnic groups and those with particular dietary preferences. There are an increasing number of disorders thought to be linked to vitamin D deficiency. Several lifestyle factors, including shift work and mode of dress, have been identified as causes for vitamin D deficiency. Maternal deficiency has also been recognised as a risk factor for neonatal complications including hypocalcaemia and rickets. These studies have led to increased demand for measurement of blood concentrations of 25-hydroxy vitamin D (25OHD), but there are barriers to effective use of the vitamin D service: (i) limited laboratory capacity; and (ii) inappropriate test requesting. To ensure that test availability is matched to clinical need, the Paediatric Biochemistry Laboratory has historically assessed any abnormalities in other standard biochemical tests of bone health before a decision is made whether to refer a sample for 25OHD analysis. The aim of this retrospective survey was to assess the relationship between the analytes included in a bone chemistry profile and parathyroid hormone (PTH) with 25OHD status in an unselected paediatric population in order to determine which test might be the best predictor of 25OHD deficiency during routine assessment.

Methods: Requests made in 2 twelve month periods (2007 and 2010) were reviewed in the laboratory information management system and adjusted calcium, phosphate, alkaline phosphatase and PTH were recorded. The proportion of abnormal results for each marker was compared across 25OHD status defined as deficient (<25 nmol/L), insufficient (25.1–50 nmol/L) or sufficient (>50 nmol/L). Only results available before subjects started vitamin D replacement were included in the 2007 dataset but they were not excluded from the 2010 dataset.

Results: 130 and 1418 unique requests for the two time periods were identified, an almost 11-fold increase. The percentage of patients with 25OHD < 50 nmol/L fell (from 70% in 2007 to 58% in 2010), perhaps as a result of including those who had started vitamin D replacement. A high proportion of 25OHD deficient patients had no abnormalities in bone biochemistry or PTH (40% in 2007 and 53% in 2010). At least one biochemical abnormality was observed in 50% (2007) and 23% (2010) of patients with sufficient 25OHD where results for all four markers were available.

Conclusions: PTH is the most likely marker to be outside the reference range (raised) if the patient is vitamin D deficient but the literature suggests the relationship between 25OHD and PTH may be complicated by glomerular filtration rate. Where abnormalities in routine biochemistry are identified, these may be monitored as an individual is treated. An absence of abnormal results, particularly in apparently asymptomatic subjects, will not exclude 25OHD deficiency.

### 2.55. Vitamin D Status of Rural Gambian Mother-Infant Pairs at Three Months Lactation

#### Billing, G.; Jarjou, L.M.A.; Nigdikar, S.; Prentice, A.; Goldberg, G.R.

Background: Vitamin D (VD) activity is assumed to be low in breast milk (Thiele, D.K., et al*. J. Hum. Lact.* 2013, *29*, 163-170) and case reports of VD deficiency in exclusively breastfed infants may deter mothers from breastfeeding. However, current data are only from women with marginal VD status or in high-dose supplement trials. Relationships between maternal and infant VD status during breastfeeding are inconsistent. This observational study was conducted in a rural subsistence farming population in The Gambia (13°N), where UVB-containing sunlight is present all year. Mothers and their infants are constantly together, and women have face, neck, shoulders, arms and feet exposed during fieldwork and gardening.

Methods: Participants were 30 healthy rural Gambian mother-infant pairs (mean ± SD maternal BMI 21.7 ± 2.6 kg/m. sq.; infant length 61.8 ± 4.9 cm, and weight 5.9 ± 1.0 kg). Mean ± SD maternal age was 25.6 ± 6.4 years (range 16.7–36.2). All mothers were unsupplemented and reported breastfeeding exclusively. Median parity was 2 (range 1–9). All infants were singleton-born, 47% were male, mean ± SD age was 13.6 ± 1.8 (range 10.8–16.4) weeks. Blood samples were collected by venepuncture from 100% of pairs: 15 mL from mothers (overnight fasted) and 3 mL from infants. Samples were stored at −70 °C and transported frozen to Cambridge, UK. Mother and infant VD status was assessed by serum concentration of 25-hydroxy vitamin D (25OHD) by chemiluminescent immunoassay (Diasorin Ltd., intra-assay CV 3.5% estimated from duplicates, DEQAS accredited).

Results: Mean infant 25OHD was 75.8 ± 16.1 nmol/L (range 46.4–116.0). Mean maternal 25OHD was 58.8 ± 15.9 nmol/L (37.3–100.5). All subjects were above 25 nmol/L, 17 mothers and 18 infants between 50–80 nmol/L, and 3 mothers and 11 infants above 80 nmol/L. There was no significant correlation between mother and infant 25OHD concentration (*r* = 0.2, *p* = 0.2).

Conclusions: This is the first report of VD status in Gambian breastfed infants, whose mothers have unrestricted exposure to tropical sunlight. Maternal VD status was similar to our previous data at 13 weeks lactation (Jarjou, L.M., et al*. Am. J. Clin. Nutr.* 2010, *92*, 450-457). These data suggest that rural Gambian breastfed infants have sufficient VD status for bone health, despite impressions that BM contains little VD. There was no significant correlation in VD status between Caucasian, supplemented mother-infant pairs at 4 months in Denmark (Við Streym, S., et al*. Eur. J. Clin. Nutr.* 2013, *67*, 1022-1028), or unsupplemented pairs at 15 weeks in Finland (Ala-Houhala, M., et al*. Am. J. Clin. Nutr.* 1988, *48*, 1057-1060). VD metabolite transfer in utero, direct infant UVB-sunlight exposure and direct infant supplementation may be important contributors to infant VD status. Further data from this study on potential determinants of VD status are being analysed, including UVB exposure using dosimeter badges (Scienterra Ltd.), VD-binding protein concentration in plasma and BM (ELISA, Pathway Diagnostics), VD and 25OHD in BM (Jakobsen, J., et al*. Br. J. Nutr*. 2007, *98*, 908-913), calcium and phosphorus in BM (Laskey, M.A., et al*. Ann. Clin. Biochem*. 1991, *28*, 49-54), and BM intake by dose-to-the-mother method (da Costa, T.H. *et al. J. Nutr*. 2010, 140, 2227-2232). Comparative studies on mother-infant pairs in contrasting geographical locations are lacking (Dawodu, A., *Adv. Nutr*. 2012, 3, 353-361). A parallel study is planned in Britain, where UVB-sunlight is seasonal and exclusive breastfeeding not widely practiced.

### 2.56. No Effect of High Dose Vitamin D on Muskle Strength and Balance—A Randomized Controlled Trial

#### Grimnes, G.; Emaus, N.; Almaas, B.; Jorde, R.

Background: Observational studies have suggested an inverse relation between serum 25-hydroxy vitamin D (25(OH)D levels and muscular strength and function in human. We wanted to study whether intervention with high dose vitamin D would improve muscular strength or balance in postmenopausal women.

Methods: The study was a 1-year randomized double-blind controlled trial including postmenopausal women with a BMD T-score ≤−2.0 in either lumbar spine (L2–4) or total hip. The participants were randomized to 6500 IU vitamin D_3_/day (20,000 IU twice per week + 800 IU/day; high dose group) or 800 IU vitamin D_3_/day (placebo twice per week + 800 IU/day; standard dose group). Both groups were given 1000 mg elemental calcium/day. Muscular strength was measured at baseline and after 12 months using hand grip strength. Proximal femoral strength (Cybex^®^) was measured in those with T-scores >−2.5 only, as osteoporosis was a relative contraindication to this examination. Balance was measured by tandem test. Serum was stored from baseline and 12 months in aliquots at −70 °C for later analyses of serum 25(OH)D using a liquid chromatography double mass spectrometry (LC-MS/MS) method.

Results: A total of 297 women were included, and 275 completed the 12 months control. Serum 25(OH)D levels increased significantly from 70.7(23.0) to 185.4(24.1) nmol/L in the high dose group and from 71.2(22.3) to 89.2(16.7) nmol/L in the standard dose group. Muscular strength was similar at baseline in the two treatment groups (hand grip dominant side 55.0(16.5) and 53.5(14.4) kPa, hand grip non-dominant side 51.9(17.8) and 51.5(15.5) kPa, and proximal femoral strength (*n* = 105) 73.0(16.2) and 70.6(15.4) (isokinetic peak torque, Nm) in the high and standard dose group, respectively), and did not change during the intervention period. The proportion achieving full score (30 s) in the tandem test was similar in the two groups at baseline (89% in the high dose and 86% in the standard dose group), and did not change significantly during the intervention period. The results were similar in those with serum 25(OH)D levels below 50 nmol/L (*n* = 42).

Conclusion: One year treatment with high dose vitamin D had no effect on muscular strength and balance in postmenopausal women with osteopenia or osteoporosis as compared to a standard dose. The study was limited by the already sufficient vitamin D levels at baseline.

### 2.57. Effect of Vitamin D Supplementation in Infancy on Bone Mineral Density of Children Aged 3–6 Years: Follow-Up of a Randomized Controlled Trial

#### Arora, H.; Rajput, M.; Rehman, A.M.; Kaur, M.; Chugh, R.; Kurpad, A.V.; Sachdev, H.P.S.; Filteau, S.; Trilok, Kumar, G.

Background: Vitamin D status or parent-selected vitamin D supplementation in infancy have been associated with better bone growth and mineralization in later childhood. However, the data from randomized trials is scarce and conflicting. A recent meta-analysis showed inconsistent effects of vitamin D supplementation on bone mineral density of healthy children. In the Delhi Infant Vitamin D Supplementation Study (DIVIDS) we randomized 2079 neonates to weekly vitamin D or placebo until age 6 months. The supplementation increased length growth at 6 months. For the present DIVIDS-2 study we followed up the children, now aged 3–6 years, to measure bone structure and strength by quantitative ultrasound (QUS).The purpose of the study was (1) to determine the effect of vitamin D supplementation in infancy on bone structure and strength of children aged 3–6 years; (2) to evaluate the association of current vitamin D status with bone structure and strength.

Method: 796 DIVIDS children and their parents were contacted using their last known address and telephone number. They were invited to the study clinic for QUS of the distal radius and midshaft tibia, blood sampling for measurement of 25-hydroxy vitamin D_3_ (25OHD) by radioimmunoassay, as well as medical examination, anthropometry, and motor development testing. QUS data were expressed as Z scores and compared between treatment groups.

Results: Demographic characteristics did not differ at follow-up between children in the vitamin D and placebo groups. QUS Z scores for radius were mean −0.72 (95% CI −0.82 to −0.62, *n* = 398) for the vitamin D group and mean −0.60 (95% CI −0.70 to −0.50, *n* = 397) for the placebo group; *p* = 0.11. For tibia, values for the vitamin D group were mean −0.57 (95% CI −0.67 to −0.47, *n* = 398) and for the placebo group mean −0.53 (95% CI −0.63 to −0.43, *n* = 398); *p* = 0.62. Adjusting for factors associated with being followed up from the original trial gave almost identical results. Serum 25OHD concentrations were mean 25.7 nmol/L (95% CI 22.2 to 29.3, *n* = 187) for the vitamin D group and mean 28.9 nmol/L (95% CI 25.4 to 32.4, *n* = 185) for the placebo group; *p* = 0.22. Concurrent factors associated with higher QUS Z scores for radius were older age at follow-up, visiting a doctor in the last month, greater dietary diversity as indicated by number of food groups eaten, and for both radius and tibia Z scores, being interviewed at follow-up during July to September (the Monsoon season).

Conclusions: In a large randomized controlled trial, vitamin D supplementation in infancy did not affect bone structure and strength in childhood.

### 2.58. Vitamin D Binding Protein Does not Fully Explain Why Daily Doses of Vitamin D of 1000 IU but not 400 IU Attenuate Hip Bone Mineral Density Loss: Results from a 1 Year Placebo Controlled Randomized Controlled Trial

#### Macdonald, H.M.; Strachan, A.; Fraser, W.D.; Wood, A.D.

Background: Variation in Vitamin D Binding Protein (VDBP) may explain the inconsistencies between studies of vitamin D; either (**a**) through a mechanism by which the circulating half-life of 25-hydroxy vitamin D [25(OH)D] may be extended or (**b**) because non-VDBP bound or free metabolites of vitamin D may better reflect biological activity. Powe *et al.* found that free 25(OH)D (calculated using VDBP and serum albumin) but not total 25(OH)D predicted bone mineral density (BMD) in 49 young adults (Powe, C.E., et al*. J. Bone Miner. Res*. 2011, *26*, 1609-1616). We carried out a 1-year randomized controlled trial in 300 postmenopausal women (age 60–70 years) using two doses of vitamin D (Macdonald, H.M, et al*. J. Bone Miner. Res.* 2013, *28*, 2202-2213). It had been specifically designed to address the issue of season, by starting all participants at the beginning of the year. The aim of this analysis was to test whether VDBP-adjusted 25(OH)D could explain more of the variation in BMD compared to total 25(OH)D. 

Methods: Total 25(OH)D was the sum of 25(OH)D_3_ and 25(OH)D_2_, measured using tandem mass spectrometry aligned to the NIST standard in a laboratory certificated through the DEQAS scheme. VDBP was measured by ELISA (R&D Biosystems^®^) and bone mineral density was assessed by a Lunar iDXA. Free 25(OH)D (total non-bound) and bioavailable 25(OH)D (includes albumin bound 25(OH)D) were calculated from serum VDBP and albumin (1). Results are given as mean [SD].

Results: Women were healthy and had low initial 25(OH)D (34 [15] nmol/L, with 30% below 25 nmol/L and over half <50 nmol/L). A total of 287 women (87%) completed the trial. For the placebo there was an initial 2-month lag followed by an increase in 25(OH)D to 55[18] nmol/L in summer, returning to 32[15] nmol/L in fall/winter. In contrast, both vitamin D doses caused a rapid increase in 25(OH)D, reaching a plateau of 65[20] nmol/L for 400 IU, and 76[19] nmol/L for 1000 IU vitamin D. We reported that 1000 IU vitamin D_3_ but not 400 IU attenuated bone mineral density loss, whereas the increase in 25(OH)D for the 1000 IU dose was only 25% above that for the 400 IU dose (2). We reported no effect on bone turnover markers, but vitamin D treatment resulted in a reduction in PTH and a small increase in serum calcium (the latter only with 1000 IU). Our recent analysis showed no association between calculated free 25(OH)D or bioavailable 25(OH)D and BMD (*n* = 264 women) at baseline. The final-visit free and bioavailable 25(OH)D but not total 25(OH)D were significantly but weakly associated with 1-year BMD change (Free: *r* = +0.15 *p* = 0.02; bioavailable: *r* = +0.16 *p* = 0.01; total *r* = +0.11 *p* = 0.08). When comparing those whose BMD had increased (*n* = 100) with those who had lost BMD (*n* = 146) it was observed that VDBP was lower in the group that had gained BMD (4.6 (1.8) µg/mL *vs.* 5.1 (1.6) µg/mL, *p* = 0.04).

Conclusion: Our data do not show a cross-sectional association between VDBP-adjusted 25(OH)D and BMD. VDBP may only partly explain the discrepancy between 25(OH)D increase and BMD change. The vitamin D in the 1000 IU dose that was not converted to 25(OH)D may be stored or converted to other metabolites, or excreted. It is hypothesised that transient changes in serum calcium may be responsible for remineralisation of marginally under-mineralised bone; and this might explain why vitamin D most benefits those who are at risk of deficiency.

### 2.59. Vitamin D Supplementation in an Edinburgh General Practice Population and Anecdotal Evidence

#### Rhein, H.M.; Johnson, G.

Background: After we became aware of the widespread vitamin D deficiency in our Scottish general practice population in Edinburgh at a latitude of 56', our patients have received for the past few years the offer of prescriptions for vitamin D supplements: 20,000 IU weekly for most adults, 2000 IU daily for pregnant women, 1000 IU daily for children aged 5–12, 400 IU daily for babies and children aged 0–5. All cancer patients with new or previous diagnosis were particularly targeted for the past 4–5 years.

Methods: Some adults, including all cancer patients, had their 25(OH)D estimated after supplementing for at least 3 months. The sizes of supplements were then adapted to aim for levels between 75 and 150 nmol/L. Collected figures are displayed.

Results: Of 124 adults tested 71 took 20,000 IU weekly, 45 took 40,000 IU weekly and 8 took 60,000 IU weekly in order to reach a satisfactory 25(OH)D concentration of above 75 nmol/L. Of 35 patients who took vitamin D at the start of their cancer diagnosis, 23 are still alive, 12 have died. Types of cancer and months of survival are charted. Of interest might be that the 5 lung cancer patients who died survived 60, 11, 8 and 8 months respectively, one survived only 1 month because of a heart attack. Two lung cancer patient are still alive and have survived 43 and 8 months so far. This compares well with the known lower median Scottish lung cancer survival of 6 months after diagnosis. We have received frequently positive patient feedback: less aches and pains, fewer cold and coughs, less fatigue, one significant improvement of severe IBS. Several nurses in the health centre’s treatment room were found to be profoundly deficient and improved significantly after taking supplements. Most of the cancer patients tested at the start of their diagnosis were severely vitamin D deficient. It is our impression that quality of life in most cancer patients was good after vitamin D supplementation, and in those who died this was right up until their final stage.

Conclusions: In a Scottish general practice (56' latitude) most adults need 20,000 IU weekly to reach an optimal range of 75–150 nmol/L, but a significant number (43% in our sample) might need more, 40,000 IU or 60,000 IU weekly. Cancer patients appear to have an improved quality of life after vitamin D supplementation. In the absence of any known negative effects of vitamin D supplementation and while waiting for conclusive trials it could be beneficial for many cancer patients to be tested and treated for vitamin D deficiency.

### 2.60. Randomized Controlled Trial of Vitamin D to Prevent Exacerbation of Asthma in School Children

#### Hidetoshi, M.; Hiroshi, T.; Takaaki, S.; Mitsuyoshi, U.

Background: In a prior influenza prevention randomized trial, we found that incidence of asthma attack was less in the vitamin D_3_ group than among those on placebo on secondary endpoint. It has been hypothesized that asthma symptom rates are higher in low serum 25-hydroxy vitamin D (25(OH)D) concentration than in high 25(OH)D concentration children. To investigate the effect of vitamin D_3_ supplementation on the incidence and control of asthma among school age children.

Method: We included six to fifteen years old children diagnosed with asthma who visited Jikei University Hospital or Fuji City General Hospital for consultation during October 2010 to April 2013. They were randomly assigned at a ratio of 3 to 2 to 60 days of administration with vitamin D or placebo, respectively, and followed up for 120 days after administration. Asthma Control Test (ACT) score, spirometry and SCORAD were performed at the ends of administration and the follow-up. We compared exacerbation of asthma and changes in ACT score, spirometry and SCORAD between groups.

Results: We assigned 89 children. After administration, the variation of 25(OH)D concentration was 6.0 (95% confidence interval [CI]: 3.4–8.6) in vitamin D group and −0.4 (95% CI: −3.7–2.9) in placebo, showing a significantly increase in vitamin D group (*p* = 0.0029), but after follow-up (visit 3), no significant difference was found between the groups with variations of 2.9 (95% CI: −0.3–6.0) in vitamin D group and 0.7 (95% CI: −3.8–5.2) in placebo (*p* = 0.4040). Exacerbation of asthma was observed in four cases with no significant difference between the groups (*p* = 0.555). While changes in spirometry and SCORAD also showed no significant difference between the groups, changes in ACT score showed a significant improvement in Group C (*p* = 0.0032). A similar trend was observed in the groups untreated with low 25(OH)D group (<30 ng/mL) (*p* = 0.012) and non inhaled corticosteroid user (*p* = 0.001).

Conclusions: Vitamin D_3_ may improve daily symptoms of asthma in well-controlled asthma school age children.

### 2.61. Vitamin D Status and Insulin Resistance in Healthy, Overweight People at High Risk of Cardiovascular Disease: Cross-Sectional Analysis from a Clamp Study

#### Wallace, I.R.; McEvoy, C.T.; Hunter, S.J.; Hamill, L.L.; Ennis, C.N.; Bell, J.V.; Woodside, P.M.; Young, I.S.; McKinley, M.C.

Background: Vitamin D is a fat soluble steroid molecule which may be obtained in the diet or synthesised by skin cells following exposure to sunlight. Low vitamin D concentrations are associated with an increased risk of type 2 diabetes mellitus (DM), as shown in longitudinal and cross-sectional studies. However the mechanism is unclear, with studies examining the relationship between vitamin D and insulin resistance showing conflicting results. We aimed to examine the association between vitamin D concentration and insulin resistance in healthy, non-diabetic, overweight individuals at high risk of cardiovascular disease using gold-standard techniques for assessment of both vitamin D concentration and insulin resistance.

Methods: Vitamin D was measured using an ultra performance liquid chromatography technique (UPLC) with tandem mass spectrometry. Insulin resistance was assessed using a two-step euglycaemic hyperinsulinaemic clamp technique. Statistical analysis was performed using Pearson’s correlation coefficients and partial correlation.

Results: Our study population comprised 92 overweight, non-diabetic individuals with no history of cardiovascular disease—mean age 56 years (range 40–77 years), 64% males, 36% females, body mass index 30.9 kg/m^2^ (range 26.4–36.9 kg/m^2^), fasting plasma glucose 5.8 mmol/L (range 4.9–7.0 mmol/L). Mean total vitamin D concentration was 32.2 nmol/L. Thirty (33%) were deficient (<25 nmol/L), 43 (47%) insufficient (26–50 nmol/L) and 19 (20%) adequate (>50 nmol/L) in vitamin D. Pearson’s correlation coefficients for vitamin D and GIR step 1 were −0.003 (*p* = 0.98), GIR step 2 –0.036 (*p* = 0.73) and HOMA-IR −0.163 (*p* = 0.13). Partial correlation analysis did not elicit any significant correlations after correction for potential anthropometric, seasonal or gender confounders. Further subgroup analysis of deficient, pre-diabetes and impaired glucose tolerance subgroups did not detect any significant correlations.

Conclusions: Using gold standard techniques this cross-sectional analysis did not reveal a significant association between vitamin D and measures of whole-body, peripheral or hepatic insulin resistance in healthy overweight individuals at high risk of cardiovascular disease. Robust randomised controlled trials are needed to determine whether improving vitamin D status has a positive effect on insulin resistance or other pathophysiological indicators of DM including beta-cell function.

### 2.62. Suppressive Effects of Vitamin D on Brain Steroidogenesis

#### Emanuelsson, I.

Background: Vitamin D has the ability to effect gene expression in basically all cell types and has therefore several functions in the body. Vitamin D has also been linked to several diseases including neurodegenerative disorders. Cholesterol and steroid hormones including estrogens are synthesized in the brain and the levels of these steroids may be linked to neurodegenerative diseases. There is no information about vitamin D: s effect on brain steroidogenesis. The aim of this study was therefore to investigate the effects of vitamin D on the gene expression of important enzymes involved in brain steroidogenesis.

Methods: Primary rat astrocytes and human neuroblastoma SH-SY5Y cells were treated with 10 nM 1,25(OH)_2_D_3_, the active form of vitamin D. The gene expression of cytochrome P450 side chain cleavage enzyme (CYP11A1), cytochrome P450c17 (CYP17A1), 3β-hydroxysteroid dehydrogenase (3βHSD) and aromatase (CYP19A1) were measured using realtime RT-PCR.

Results: The gene expression of CYP11A1, CYP17A1, 3β-HSD and CYP19A1 were all decreased in astrocytes by the 1,25(OH)_2_D_3_ treatment. In SH-SY5Y cells the gene expression of CYP19A1 and CYP17A1 were decreased. All these enzymes are important for conversion of cholesterol to sex steroids. CYP19A1 is required for formation of estrogens. The strongest suppressive effect was seen on CYP11A1, 3β-HSD and CYP19A1 where the vitamin D treatment decreased the gene expression to 30% compared to control. The effects on CYP17A1 as well as CYP19A1 suggest that the levels of estrogens may be affected by vitamin D.

Conclusions: The results indicate that vitamin D has a suppressive effect on some of the genes for enzymes in the brain steroidogenesis implicating that the products of these enzymes may be decreased. These data can contribute to the understanding of vitamin D: s complex function in the body and in the brain. Future studies will be directed to analysis of effects of vitamin D on enzyme activities.

### 2.63. Genetic Variations in the Vitamin D Receptor Predict Type II Diabetes Mellitus in a General Population. The Tromsø Study

#### Zostautiene, I.; Jorde, R.; Schirmer, H.; Kamycheva, E.

Background: Recent observational studies have revealed associations between low serum 25-hydroxy vitamin D (25(OH)D) levels and overweight, metabolic syndrome and atherosclerosis. On the other hand, there are indications of possible harmful effects of 25(OH)D levels >100 nmol/L on cardiovascular outcomes and vascular health. To complicate the picture, genetic differences in the vitamin D receptor (VDR) may also be of importance. In the present study we aimed to study if the single nucleotide polymorphism (SNP) rs7968585 in the VDR gene predicts type II diabetes mellitus (T2DM).

Methods: DNA was prepared from subjects who participated in the fourth survey of the Tromsø Study in 1994–1995 and who were registered with T2DM (endpoint register complete till the end of 2010), as well as a randomly selected control group. Serum 25(OH)D was measured and genotyping performed for rs7968585. Cox regression, adjusted for sex, age and body mass index, was used to study the hazard ratios (HR) for risk of T2DM with the major homozygote of rs7968585 as reference and with observation time from birth until 2010.

Results: 1197 subjects were registered with T2DM and 3586 were included as control subjects. Serum 25(OH)D did not differ significantly between subjects with T2DM and controls, neither between the rs7968585 genotypes. In the Cox regression model, subjects with the minor homozygote genotype for rs7968585 had a HR of 1.30 (95% CI 1.08; 1.53) and subjects with the heterozygote genotype 1.21 (95% CI 1.05; 1.40) for risk of T2DM with the major homozygote genotype as reference.

Conclusions: The VDR SNP rs7968585 predicts the occurrence of T2DM, which may indicate a role of vitamin D in the pathogenesis of T2DM.

### 2.64. 25-Hydroxy Vitamin D Assay Requests—Are They All Necessary?

#### Hayden, K.E.; Sandle, L.N.; Berry, J.L.

Background: There is perceived excess in 25-hydroxy vitamin D (25OHD) assay requesting from primary care. In the absence of a consensus national strategy for this, and against the background of a ten fold increase in requests in the last ten years we sought to explore whether local patterns of 25OHD assay requesting were appropriate.

Methods: Data on 25OHD assay requesting from primary care was collected from laboratory databases at Manchester Royal Infirmary for the year January–December 2012 and Trafford General Hospital for 6 months July–December 2012 (when samples were sent to Central Manchester Foundation Trust for measurement). Samples were assayed using an ABSciex 5500 tandem mass spectrophotometer and the Chromsystems 25OHD kit for LC-MS/MS following the manufacturers’ instructions (intra- and inter-assay CV 3.7% and 4.8% respectively).

Results: There were 9143 requests for 25OHD measurement received from Central Manchester General Practitioners (GPs). Repeat requests accounted for 10% of this workload. 31.5% of all patients were profoundly deficient (<25 nmol/L) and a similar percentage (30.1%) were insufficient (25–50 nmol/L) and at increased risk of disease. 2217 requests were received from Trafford GPs in 6 months, of which 5.2% were repeat requests. 27.4% of Trafford patients were deficient and 29.4% were at increased risk of disease. In both Central and Trafford approximately 50% of all requests came from just 6 GP practices. The majority of deficient or insufficient patients showed an improvement on follow-up. Those already sufficient remained the same or showed a reduction. Analysis of the data by electoral ward, from the latest available census information, showed considerable variation in the ethnic mix, categorised here as White, Asian, Black, Chinese, Mixed/Other. As expected, electoral wards with the highest proportion of Asian people had the highest number being deficient and insufficient. There were more predominantly White areas in Trafford compared to Central. Despite this, there were still more than 40% of patients deficient/insufficient in Trafford wards where over 90% of occupants were White.

Conclusion: Over 56% of all patients in this urban area showed increased risk of bone, and potentially other diseases, based on their 25OHD assay results alone. This was an unselected population presenting at GPs for a variety of reasons and these patients will not necessarily show any signs or symptoms of inadequate levels of 25OHD. This is of concern since it may reflect undiagnosed levels of vitamin D deficiency/insufficiency in the wider population. Given the rapid increase in requests in the last decade, we believe that a 56% abnormality rate (<50 nmol/L) does not indicate an inappropriate rate of requesting.

### 2.65. Vitamin D Status Is Related to Erectile Dysfunction Severity and Cardiovascular Risk in Ageing New Zealand Men

#### Quilter, M.L.; von Hurst, P.; Hodges, L.; Coad, J.

Background: Epidemiological studies suggest an association between vitamin D status and cardiovascular disease (CVD) risk. Suboptimal vitamin D status appears to increase an individual’s risk of developing CVD, although the optimal level required is unclear and the results of intervention studies remain heterogeneous and inconclusive. Erectile dysfunction (ED) is increasingly recognised as an early harbinger of CVD and can be assessed using the validated International Index of Erectile Function 5-Item questionnaire (IIEF-5 scores ranging 5–25). Vasculogenic ED is synonymous with endothelial dysfunction and may be valuable in identifying men at risk of CVD at an early stage, supporting timely intervention. This study investigated whether vitamin D status predicts ED and other CVD markers in ageing men.

Methods: We conducted an observational study in 100 self-selected healthy men aged 40–70 years in the Manawatu region, New Zealand. Medical history, height, weight, waist: hip (WHR), body fat (% BF), blood pressure (BP), fasting lipid profile (TG, TC, HDL-c, LDL-c), glucose, insulin, 25(OH)D) and ED score (IIEF-5) were assessed. Framingham and HOMA2-IR scores were calculated.

Results: The mean serum 25(OH)D level was 82.80 ± 27.38 nmol/L. 42 men had suboptimal (<79.9 nmol/L) and 58 had optimal status (≥80 nmol/L), based on currently debated optimal cut-off levels for cardiometabolic health. Log 25(OH)D was significantly correlated with date of appointment (*r* = 0.390, *p* = 0.000), BMI (*r* = −0.220, *p* = 0.028), WC (*r* = −0.252, *p* = 0.012), WHR (*r* = −0.270, *p* = 0.007), waist:height (*r* = −0.273, *p* = 0.006), % BF (*r* = −0.338, *p* = 0.001), HDL-c (*r* = 0.287, *p* = 0.004), LDL-c (*r* = −0.250, *p* = 0.013), TC:HDL-c (*r* = −0.292, *p* = 0.003) and 10yr Framingham risk scores (CHD *r* = −0.353, *p* < 0.001). The mean IIEF-5 score was 21.66 ± 4.69 with 29 cases of ED, 69 cases of no ED. IIEF-5 score was significantly correlated with age (rs = −0.444, *p* < 0.001), WC (rs = −0.255, *p* = 0.011), WHR (rs = −0.281, *p* = 0.005), waist:height (rs = −0.230, *p* = 0.022), % BF (rs = −0.205, *p* = 0.043), syst.BP (rs = −0.225, *p* = 0.026), insulin (rs = −0.249, *p* = 0.013), HOMA2-IR (rs = −0.237, *p* = 0.019) and 10yr Framingham risk scores (CHD rs = −0.386, *p* < 0.001). IIEF-5 was significantly positively correlated with log 25(OH)D (rs = 0.203, *p* = 0.045) and ANOVA indicated significant differences between men with and without ED respectively for age (59.28 ± 8.84 *vs.* 52.19 ± 8.56, f = 13.724, *p* = 0.000), Framingham risk scores (*p* < 0.05) and log 25(OH)D (1.854 ± 0.140 *vs.* 1.914 ± 0.130, f = 4.159, *p* = 0.044).

Conclusions: Vitamin D status was significantly correlated with both ED severity and markers for CVD such as central adiposity, fasting lipid profile and Framingham risk scores. Men with ED were significantly older, had higher CVD risk scores and significantly lower levels of 25(OH)D than men without ED. This study supports a potential role for vitamin D in CVD development, however further research is required to establish causality. There is a need for randomised controlled human intervention trials to investigate whether improving vitamin D status in men ameliorates vasculogenic ED and reduces the risk of CVD.

### 2.66. Investigating the Effect of Vitamin D Status on Cognitive Function Using a Mendelian Randomisation Approach

#### Maddock, J.; Cavadino, A.; Kjærgaard, M.; Schöttker, B.; Perna, L.; Saum, K.U.; Kumari, M.; Sanchez, A.; Räikkönen, K.; Lahti, J.; Kilander, L.; Byberg, L.; Melhus, H.; Ingelsoon, E.; Sen, A.; Petrovic, K.E.; Schmidt, H.; Schmidt, R.; Lind, L.; Michaëlsson, K.; Eriksson, J.; Kivimaki, M.; Engmann, J.; Dieffenbach, A.K.; Brenner, H.; Schirmer, H.; Jorde, R.; Power, C.; Llewellyn, D.; Hyppönen, E.

Background: Recent epidemiological and experimental studies have linked vitamin D deficiency to a range of non-skeletal conditions including reduced cognitive function. While some observational studies have indicated that there is an association between hypovitaminosis D and poor cognitive function in mid- to later- life, the influence of confounders and the potential for reverse causality remains a concern. Mendelian randomisation (MR) is an approach that uses genetic variants as instruments representing an environmental exposure, in this case 25-hydroxy vitamin D (25(OH)D), to estimate the relationship between 25(OH)D and cognitive function. Since the genetic variant is randomly assigned from parents to offspring during meiosis and is present from birth, the possibility of confounding and reverse causality is minimised.

Methods: This study used data from 43,954 participants from nine European cohorts. DHCR7 (rs12785878), CYP2R1 (rs12794714) and their combined “synthesis” score were chosen to act as a proxy for 25(OH)D. Serum 25(OH)D concentrations were available in six studies and naturally log transformed when used as an outcome. Cognitive tests in each cohort were combined into standardised global and memory scores. As associations for 25(OH)D and cognitive function were non-linear, all analyses were stratified by study-specific 25(OH)D tertiles. Meta-analysis was used to assess the phenotypic and genetic associations between 25(OH)D concentrations and cognitive function. Where there was evidence of heterogeneity between studies (pheterogeneity <0.05) random effects models were used, otherwise fixed effects meta-analysis was applied.

Results: In meta-analysis, the association of 25(OH)D with global and memory cognition was non-linear (pcurvature <0.003 for both). When stratified by 25(OH)D tertiles, coefficients for global cognition per 10 nmol/L increase in 25(OH)D were 0.05 (95% CI 0.003 to 0.09) in the lowest third (T1), 0.07 (0.01 to 0.12) for T2 and −0.02 (−0.05, 0.01) for the highest third (T3). The coefficients for memory cognition per 10 nmol/L increase in 25(OH)D were 0.01 (−0.03 to 0.06) for T1, 0.03 (−0.02 to 0.09) for T2 and −0.03 (−0.04 to −0.01) for T3. DHCR7, CYP2R1, and the synthesis score were associated with 25(OH)D (*p* < 0.001). However, coefficients for the association with global or memory cognition were in the opposing direction for DHCR7 and CYP2R1, with no overall association observed for the synthesis score (global cognition: *p* = 0.19, 0.08 and 0.47; memory cognition *p* = 0.04, 0.29 and 0.97 respectively). There was no evidence of heterogeneity in any of the genetic analyses, with the coefficients for the association of the synthesis score with global and memory cognition being similar across 25(OH)D tertiles.

Conclusions: We found no evidence for 25(OH)D concentrations acting as a causal factor for cognitive performance in mid- to later life. However, the phenotypic association between 25(OH)D and cognition was strongly non-linear, and our genetic analyses may have been underpowered to detect small causal effects operating at the extremes of 25(OH)D distribution.

### 2.67. Dietary Vitamin D_2_—Is it a Potentially Under-Estimated Contributor to Vitamin D Nutriture at a Population Level?

#### Walton, J.; Kinsella, M.; McNulty, B.A.; Gibney, M.J.; Flynn, A.; Kiely, M.; Cashman, K.D.

Background: It has been suggested that vitamin D_2_ is not very prevalent in the human food chain. However, data from a number of recent intervention studies suggest that the majority of subjects had measurable serum 25-hydroxy vitamin D_2_ (25(OH)D_2_) concentrations. Serum 25(OH)D_2_, unlike 25(OH)D_3_, is not directly influenced by sun exposure of skin, and thus has dietary origins, however, quantifying dietary vitamin D_2_ is difficult due to limitations of food compositional data. Our objective is to characterize serum 25(OH)D_2_ concentrations in participants of the National Adult Nutrition Survey (NANS) in Ireland, and to use these serum concentrations to estimate vitamin D_2_ intakes using a mathematical approach.

Methods: LC-tandem MS from NANS was measured by a liquid chromatography-tandem mass spectrometry (LC-tandem MS) method, which resolves 25(OH)D_2_, 25(OH)D_3_ and the C3-epimer of 25(OH)D_3_. The performance of our LC-tandem MS method has been compared to that of a Reference Measurement Procedure (Stepman, H.C.M., et al. *Clin. Chem*. 2011, *57*, 441–448) and it is very comparable (Y = X × 1.0083 (95% CI, 0.9939, 1.0277) − 0.1704 (95% CI, −0.2579, −0.0827); *r*^2^ = 0.999; *n* = 17). The limit of detection (LoD) and limit of quantification (LoQ) of serum 25(OH)D_2_ for our method was 0.44 and 1.43 nmol/L, respectively. Information on diet as well as subject characteristics was accessed from NANS database. Data from a meta-analysis and meta-regression (Cashman, K.D., et al. *Br. J. Nutr.* 2011, *106*, 1638-1648) as well as a RCT with vitamin D_2_ and D_3_ (Logan, V.F., et al. *Br. J. Nutr.* 2013, *109*, 1082-1088) was used to estimate the slope response of serum 25(OH)D_2_ to vitamin D_2_ intake assuming equal potency as vitamin D_3_ and a lesser potency. These slopes were used to convert serum 25(OH)D_2_ data to vitamin D_2_ intake estimates.

Results: Of the entire NANS population for which serum 25(OH)D_2_ concentrations were measured (*n* = 1123), only two subjects had serum 25(OH)D_2_ concentrations below the LoD, 237 subjects had concentrations between the LOD and LoQ, and 78.7% (n 884) had serum 25(OH)D_2_ concentrations above the LoQ. The mean, 10th, 50th (median) and 90th percentile of serum 25(OH)D_2_ concentration in those subjects who had serum 25(OH)D_2_ concentrations above the LoQ was 3.69, 1.71, 2.96, 6.36 nmol/L, respectively; whereas the maximum serum 25(OH)D_2_ concentration was 27.64 nmol/L. The projected 5th to 95th percentile suggested adults had intakes of vitamin D_2_ in the range of 0.5–0.7 to 4–6 μg/day, respectively, and a median intake of 1.4–1.9 μg/day.

Conclusion: The present data demonstrates that 25(OH)D_2_ exists in the sera of adults in this nationally representative sample. Vitamin D_2_ may be having an impact on nutritional adequacy at a population level and thus warrants further investigation.

### 2.68. Real-Life Use of Vitamin D_3_-Fortified Bread and Milk during a Winter Season: The Effects of CYP2R1 and GC Genes on 25-Hydroxy Vitamin D Concentrations in Danish Families

#### Nissen, J.; Vogel, U.; Ravn-Haren, G.; Andersen, E.W.; Nexø, B.A.; Andersen, R.; Mejborn, H.; Madsen, K.H.; Rasmussen, L.B.

Background: Low blood concentrations of serum 25-hydroxy vitamin D (s-25(OH)D) are common during winter months. Using twin and family-based studies, genome wide association studies and candidate gene studies, it has been showed that genetic factors may influence s-25(OH)D concentrations considerably. Thus, genetic factors may help to identify who is most at risk of developing low vitamin D status.

Methods: We used data from the vitamin D study, a double-blinded, randomized placebo-controlled intervention trial with healthy Danish families (*n* = 782). Participants were allocated to either vitamin D_3_-fortified bread and milk or non-fortified placebo bread and milk during a 6-month winter period in Denmark. The participants were genotyped for 25 SNPs in the vitamin D pathway and two common single nucleotide polymorphisms (SNPs) in the CYP2R1 gene (rs10741657 and rs10766197) and two common SNPs in the GC gene (rs4588 and rs842999) were found to predict baseline s-25(OH)D levels. S-25(OH)D concentrations before and after intervention was measured by LC-MS/MS. We estimated total vitamin D intake as the sum of dietary vitamin D, usage of multivitamin and vitamin D supplementation and intake of vitamin D_3_-fortified bread and milk for the fortification group. Genetic risk score (GRS) was calculated as the sum of number of risk alleles for CYP2R1 gene (rs10741657 and rs10766197) and GC gene (rs4588 and rs842999).

Results: At the end of the winter season we found that CYP2R1 (rs10741657) and GC (rs4588 and rs842999) were statistically associated with serum 25(OH)D concentrations and CYP2R1 (rs10766197) was borderline significant (*p* = 0.0599) for the fortification group. No association was found for the control group and hence no difference in mean s-25(OH)D concentrations and carrying 0 to 8 risk alleles (*p* = 0.1428) of CYP2R1 gene (rs10741657 and rs10766197) and GC gene (rs4588 and rs842999) were observed for the control group. For the fortification group, there was a negative linear trend for s-25(OH)D concentrations and carrying 0 to 8 risk alleles (*p* < 0.0001). We found a significant positive linear relationship between carrying 0–2, 3, 4 or 5 risk alleles, total vitamin D intake and the increase in s-25(OH)D concentrations (*p* = 0.0012, 0.0001, 0.0118 and 0.0029), respectively. For participants carrying 6–8 risk alleles there was no association (*p* = 0.1051). Adequate s-25(OH)D concentrations were achieved for more participants carrying a low GRS compared to participants carrying a higher GRS.

Conclusions: At the end of a winter season, there was an association between genetic variation in CYP2R1 and GC genes and s-25(OH)D concentration for the fortification group but not for the control group. Carriers of a high GRS of CYP2R1 (rs10741657 and rs10766197) and GC (rs4588 and rs842999) were more prone to be vitamin D insufficient compared to carriers of low GRS. Carriers of a high GRS may need a higher amount of vitamin D supplementation to achieve adequate s-25(OH)D concentrations. Importantly, it is seemed that low risk carriers with adequate s-25(OH)D concentrations achieved even higher s-25(OH)D concentrations with increasing vitamin D intake whereas high risk carriers did not. In the future, genetic factors might be taken into account when recommending vitamin D supplementation or food fortification.

### 2.69. Effects of Vitamin D on CMV Serology in Urban-Dwelling Minority Ethnic Groups

#### Webber, J.; Gill, P.S.; Moss, P.A.H.

Background: Vitamin D is known to affect immune function; however it is uncertain if vitamin D can alter the immune response towards the persistent herpesviruses, EBV and CMV. Observational studies show that vitamin D deficiency may increase the likelihood of EBV reactivation, and that there is a weak negative correlation between vitamin D and anti-EBNA1 titre. Further, vitamin D supplementation may specifically lower anti-EBNA-1 titres. These studies address EBV only, and in predominantly white populations. We investigate for the first time whether vitamin D levels may alter CMV serology in a cohort of non-white adults resident in the UK.

Methods: Serum samples were obtained from the Ethnic Echocardiographic Heart of England Screening (E-ECHOES) study. This was a cross-sectional study designed to assess the prevalence of heart failure in South-Asian or Afro-Caribbean participants living in Birmingham, UK. Study participants were 45 or older. The study protocol was approved by the Local Research Ethics Committee and written consent was obtained. Total vitamin D measurements were made on 1908 serum samples, via LC-MS methodology in a DEQAS-accredited clinical biochemistry lab. CMV antibody titre was determined on a subset (*n* = 902) of these serum samples, via an in-house anti-CMV IgG ELISA which reports semi-quantitative units. Intra- and inter-assay coefficients of variation were 6.9% and 9.6% respectively. Vitamin D displays a pattern of seasonal variation, which was described by cosinor analysis. Multivariate linear regression was used to investigate relationships between continuous variables.

Results: Unadjusted mean levels of vitamin D for South-Asian and Afro-Caribbean participants were 9.6 and 14.3 ng/mL, respectively. Addition of cosinor terms showed a significantly improved fit when compared to a mean model (*r* = 0.241, *p* = 0.001). Addition of demographic variables such as age, ethnicity and interaction terms improved the fit further (*p* = 0.422, *p* = 0.001). 98% of participants were seropositive for CMV. When examined via linear regression, there was a weak but significant negative correlation between vitamin D and CMV antibody titre (*r* = 0.102, *p* = 0.005). However, when the previously defined covariates (age, ethnicity, cosinor terms) were added to the linear regression model, vitamin D ceased to be a significant predictor of CMV antibody titre.

Conclusions: Upon bivariate analysis, we observed a correlation between vitamin D and CMV antibody titre, similar in magnitude to a previously published correlation between vitamin D and EBNA1 titres (Salzer J, Nystrom M, Hallmans G, Stenlund H, Wadell G, Sundstrom P. Epstein-Barr virus antibodies and vitamin D in prospective multiple sclerosis biobank samples. Mult Scler J. 2013 Oct; 19(12): 1587-91). However, upon multivariate analysis, we could observe no independent association between these variables. The apparent discrepancy between CMV and EBV serology could reflect the underlying differences between ethnic groups, or differences between the biology of CMV and EBV infection.

### 2.70. Vitamin D Insufficiency and CD4 Trajectory in People with HIV Viraemia: Preliminary Results

#### Klassen, K.M.; Fairley, C.K.; Kimlin, M.G.; Chen, M.; Ebeling, P.R.

Background: Vitamin D is essential for calcium absorption and bone mineralization. There is also evidence that vitamin D plays an important role in other aspects of health, including infection and immunity. Vitamin D deficiency has been associated with an increased progression of HIV disease and mortality in people with HIV (Sudfeld, C.R., et al*. PLoS One* 2012, *7*, e40036. Viard, J.P., et al*. AIDS* 2011, *25*, 1305-1315). Vitamin D may aid in supporting the innate and adaptive immune responses, and henceforth CD4 T-cell preservation; studies have found that vitamin D insufficiency was associated with CD4 changes after antiretroviral therapy (ART) initiation (Aziz, M., et al*. AIDS* 2013, *27*, 573-578). As most studies examining vitamin D status and disease progression to date have included individuals receiving ART, the aims of this study are to evaluate the effect of vitamin D status over time on immunological outcomes, such as CD4+ cell count and CD4+ percentage in HIV-infected individuals with HIV viraemia.

Methods: This study is a retrospective cohort study of people with HIV attending for care at a large urban clinic in Melbourne, Australia. Patients with at least one 25-hydroxy vitamin D measurement taken between 1 January 2008 and 31 December 2012, who had an HIV viral load >50 copies/mL and were not receiving ART at the time of vitamin D measurement were included in this study. Absolute CD4+ cell count, CD4% and HIV viral load are measured 3–6 monthly and will be collected from the time of vitamin D measurement until the 31st December 2013. Severe vitamin D deficiency was defined as <25 nmol/L, vitamin D deficiency as <50 nmol/L and vitamin D insufficiency as <75 nmol/L. A proportional hazards model will be constructed to identify the hazard of an individual’s decrease in CD4+ cell count to <350 cells/uL for those with vitamin D insufficiency. Generalised estimating equations will be used to examine the relationship between vitamin D status and changes in absolute CD4+ cell count and CD4% over time.

Results: Of those individuals with a vitamin D measurement, 312 were found to have an HIV viral load >50 copies/mL and not receiving antiretroviral therapy. Characteristics of participants included in this study: Baseline characteristics *n* = 312*; Age, years 34(10); Male sex 290 (93%); Europid country of birth 222 (71%); CD4 (cells/µL) 516 (254); CD4 (% of T-lymphocytes) 25 (8); CD4<200 21 (7%); CD4<350 81 (26%); Antiretroviral naïve 144 (46%); Season of vitamin D measurement:Winter 113 (36%); Spring 94 (30%); Summer 62 (20%); Autumn 43 (14%); Vitamin D (nmol/L) 57 (28); Vitamin D ≥ 75 nmol/L 75 (24%); Vitamin D < 50 nmol/L 142 (45%); Vitamin D < 25 nmol/L 29 (9%); *mean(SD) or *n* (%). Results from the proportional hazards and generalized estimating equations models will be available in early 2014 once this data has been collected.

Conclusions: Vitamin D status has been associated with disease progression and mortality in people with HIV. This has not been well described in people not receiving antiretroviral therapy, therefore the results from this study will help to fill this gap.

### 2.71. Effect Modification of Vitamin D Status and Antiretroviral Therapy on Bone Mineral Density in People with HIV

#### Klassen, K.M.; Fairley, C.K.; Kimlin, M.G.; Haskelberg, H.; Emery, S.; on behalf of the STEAL Study Group; Anderson, P.; Ebeling, P.R.

Background: Vitamin D has an important role in calcium absorption and its deficiency has also been associated with reduced bone mineral density (BMD) and increased risk of falls. Tenofovir (TDF), a commonly prescribed antiretroviral medicine, has been associated with reduced BMD, proximal renal tubular dysfunction excessive urinary phosphate losses 3, increased parathyroid hormone (PTH) levels, increased 1,25(OH)sD and increased bone formation and bone resorption levels compared to those not using TDF in people with HIV. Several studies have found an interaction between vitamin D status and antiretroviral therapy on calcaemic outcomes such as 1,25(OH)_2_D and PTH levels. It is still unclear whether the altered 25(OH)D levels seen affect the active vitamin D metabolite 1,25(OH)_2_D and health outcomes (such as bone mineralization). The aim of this study is to evaluate vitamin D, calcium and phosphate metabolites and their impact on bone mineral density in HIV-infected antiretroviral-experienced adults.

Methods: STEAL was an open-label, prospective, randomized, noninferiority study that compared simplification of current NRTIs to fixed-dose combination tenofovir (TDF)-FTC or abacavir-3TC over 96 weeks in 357 adults with plasma HIV viral load >50 copies/mL. 160 participants with baseline visit plasma samples available were randomly selected from this study for this analysis. Multiple linear regression models were constructed for baseline BMD at the lumbar spine and right hip. Interaction effects between vitamin D deficiency (<50 nmol/L) and antiretroviral therapy were explored. 25(OH)D was analysed in one batch at South Australia Pathology using IDS-iSYS 25(OH)D assay. BMD was measured using dual X-ray absorptiometry.

Results: Baseline characteristics were: 159 (99%) were male, 138 (86%) white, 53 (33%) and 90 (56%) were previously receiving abacavir and TDF respectively. Mean duration of TDF use was 1.8 years prior to baseline visit. 39 (24%) were receiving protease inhibitors prior to baseline visit and the remaining 121 (76%) were receiving an NNRTI. Eighty-five (53%) were vitamin D deficient (defined as <50 nmol/L). A linear regression model was constructed which included an interaction term between vitamin D deficiency and previous TDF use. Baseline covariates significantly associated with lower hip BMD adjusting for age were previous TDF use, but only in those with vitamin D sufficiency (mean difference −0.07 g/cm3 95% confidence interval (CI) −0.01 to −0.13; *p* = 0.02), lean body mass (7 gram increase in lean body mass for every 1 g/cm3 increase in BMD, 95% CI 4.28,9.84; *p* < 0.001), vitamin D deficiency (mean difference −0.04 g/cm3 95% CI −0.009 to 0.09; *p* = 0.03), vitamin D deficiency in those not on TDF (mean difference between vitamin D sufficiency and insufficiency in those not on TDF-0.09 g/cm3 95% CI −0.15 to −0.01; *p* = 0.006), and white ethnicity (−0.07, 95% CI −0.007 to −0.13). In a linear regression model for BMD of the spine, this interaction effect was not significant.

Conclusions: TDF has the predominant effect on BMD irrespective of vitamin D status. However, vitamin D deficiency (<50 nmol/L) appears to amplify BMD loss in the hip in those not on TDF. Vitamin D supplementation may ameliorate bone loss seen in patients not on TDF and should be evaluated in longitudinal studies.

### 2.72. Value Assignment of Vitamin D Metabolites in Vitamin D Standardization Program (VDSP) Serum Samples

#### Phinney, K.W.

Background: A primary objective of the Vitamin D Standardization Program (VDSP) is the standardization of total 25-hydroxy vitamin D (25(OH)D) measurements over time, location, and laboratory procedure. Standardization of these measurements is necessary for comparing data across different populations, interpreting the results of research studies, and for ensuring appropriate decision making by medical professionals. As part of this VDSP effort, concentrations of total 25(OH)D, meaning the sum of 25(OH)D_2_ and 25(OH)D_3_, were determined in 50 single donor serum samples. These assigned values serve as the foundation for several aspects of the VDSP, including assessment of the performance of assays for total 25(OH)D, evaluating the commutability of reference materials and quality assurance testing materials, and in developing study designs for standardizing data from completed national health surveys.

Methods: Concentrations of 25(OH)D were determined by two independent isotope-dilution liquid chromatography/tandem mass spectrometry (ID LC-MS/MS) methods developed by the National Institute of Standards and Technology (NIST) and by Ghent University. Both methods are recognized as reference measurement procedures by the Joint Committee for Traceability in Laboratory Medicine (JCTLM). Each sample was analyzed in triplicate by Ghent University and in duplicate by NIST. Standard Reference Material (SRM) 972 Vitamin D in Human Serum was employed as a control material by both laboratories.

Results: Values for 25(OH)D_2_ and 25(OH)D_3_ were assigned to each of the 50 samples based upon the average of the averages of determinations made by NIST and Ghent. Results for 3-epi-25(OH)D_3_ were based solely on the average of results from NIST. For 25(OH)D_2_, only 17 samples had concentrations that were above the limit of quantitation (LOQ) for both laboratories. Values for total 25(OH)D were based upon the sum of 25(OH)D_2_ and 25(OH)D_3_ and included those values for 25OHD_2_ that were below the LOQ. Results from each laboratory for SRM 972 were used to evaluate bias, and none of the biases differed significantly from zero. An uncertainty evaluation was also performed for each of the assigned values, including 25(OH)D_2_, 25(OH)D_3_, and total 25(OH)D. The median coefficient of variation for total 25(OH)D was 1.8%.

Conclusions: Standardization of 25(OH)D measurements is important for accurate and consistent identification of inadequate and/or deficient vitamin D levels and related health consequences in individuals and populations. Value assignment of the 50 single donor samples described here represents a fundamental component of the VDSP and supports its goal to improve clinical and public health practice worldwide.

### 2.73. Increased Risk of Upper Respiratory Infection with Addition of Intermittent Bolus-Dose Vitamin D Supplementation to a Daily Low-Dose Regimen

#### Witt, K.D.Y.; Patel, M.; Balayah, Z.; Syed, A.; Stevens, N.; Jolliffe, D.A.; Hooper, R.L.; Timms, P.M.; Clark, D.A.; Eldridge, S.; Barnes, N.; Griffiths, C.J.; Martineau, A.R.

Background: Meta-analysis of clinical trials of vitamin D supplementation for the prevention of acute respiratory infection (ARI) shows a protective effect in the general population, but there is controversy regarding the optimal dosing regimen. Low-dose vitamin D supplementation is already recommended in older adults for prevention of fractures and falls, but clinical trials investigating whether higher doses could provide additional protection against ARI are lacking.

Methods: We conducted a double-blind cluster-randomised placebo-controlled trial of high- *vs.* low-dose vitamin D supplementation in residents and staff of sheltered accommodation schemes in London, UK. 108 schemes were allocated to receive the intervention (vitamin D_3_ 2.4 mg 2-monthly + 10 μg daily for residents; 3 mg 2-monthly for staff) or control (vitamin D_3_ 10 μg daily for residents, nil for staff) over the course of one year. The primary endpoint of the trial was time from first dose of study medication to date of first ARI, determined by a validated acute respiratory symptom score recorded prospectively in a symptom diary. Secondary outcomes included time to first upper/lower respiratory infections (URI/LRI) and mean serum 25-hydroxy vitamin D (25(OH)D) concentration.

Results: 240 participants were included in the intention-to-treat analysis (137 participants in 54 schemes allocated to intervention, mean baseline 25(OH)D 43.8 nmol/L *vs.* 103 participants in 54 schemes allocated to control, mean baseline 25(OH)D 43.8 nmol/L). Median time to ARI was 203 days in the intervention arm and 227 days in the control arm (adjusted HR 1.18, 95% CI 0.80 to 1.74, *p* = 0.42). Allocation to the intervention arm of the trial was associated with increased risk of URI (adjusted HR 1.48, 95% CI 1.02 to 2.16, *p* = 0.04), but not with altered risk of LRI (adjusted HR 1.12, 95% CI 0.73 to 1.70, *p* = 0.61). Mean 25(OH)D at 1 year was 84.8 nmol/L *vs.* 58.5 nmol/L in intervention *vs.* control arms (*p* < 0.0001).

Conclusions: Addition of intermittent bolus-dose vitamin D supplementation to a daily low-dose regimen improved vitamin D status in older adults and their carers, but it did not influence risk of ARI, and was less effective at preventing URI.

### 2.74. Vitamin D Metabolism Is Dysregulated in Patients with Tuberculosis

#### Witt, K.D.; Jolliffe, D.A.; Thummel, K.; Wang, Z.; Timms, P.M.; Griffiths, C.J.; Martineau, A.R.

Background: Vitamin D deficiency associates with active tuberculosis (TB), but the question of whether this arises as a cause or as a consequence of disease is controversial. Paired comparison of serum concentrations of vitamin D metabolites in TB patients at diagnosis *vs.* following recovery has potential to inform the debate, but such studies have not previously been conducted.

Methods: We conducted a longitudinal study comparing serum concentrations of vitamin D metabolites in TB patients at long-term follow-up *vs.* diagnosis. Participants diagnosed with pulmonary TB in 2007-9 were invited to attend a follow-up visit in 2012-3. Concentrations of 25-hydroxy vitamin D (25(OH)D), 1α,25-dihydroxy vitamin D (1,25(OH)_2_D), 24R,25-dihydroxy vitamin D (24,25(OH)_2_D) and 4β,25-dihydroxy vitamin D (4,25(OH)_2_D) were determined in serum samples collected at follow-up and at the time of TB diagnosis. Ratios of 1,25(OH)_2_D, 4,25(OH)D and 24,25(OH)_2_D to 25(OH)D were calculated to determine activity of CYP27B1, CYP3A4 and CYP24 respectively. Mean values of metabolite concentrations and ratios were compared between the two time points using Student’s paired *t*-tests.

Results: Thirty-one participants were followed up between August 2012 and February 2013. Mean serum concentrations of both 25(OH)D and 24,25(OH)_2_D were significantly lower at diagnosis *vs.* post-recovery (for 25(OH)D, 12.2 *vs.* 29.7 nmol/L respectively, *p* < 0.0001; for 24,25(OH)_2_D, 1.5 *vs.* 3.2 nmol/L respectively, *p* = 0.004), but no statistically significant differences in serum concentrations of 1,25(OH)_2_D or 4,25(OH)_2_D between the two time points were seen. Mean ratios of 1,25(OH)_2_D: 25(OH)D and 4,25(OH)_2_D: 25(OH)D were both elevated at diagnosis *vs.* post-recovery (for 1,25(OH)_2_D: 25(OH)D, 10.0 × 10^−3^ *vs.* 4.9 × 10^−3^ respectively, *p* = 0.003; for 4,25(OH)_2_D: 25(OH)D, 2.4 × 10^−3^ *vs.* 1.6 × 10^−3^ respectively, *p* = 0.02 ), indicating increased activity of CYP27B1 and CYP3A4 at diagnosis. No statistically significant difference in mean 24,25(OH)_2_D: 25(OH)D ratio was seen between the two time points. The difference in mean serum 25(OH)D concentration at follow-up *vs.* baseline remained statistically significant after exclusion of 14 participants who were taking supplemental vitamin D at follow-up and/or who had increased their sun exposure since time of diagnosis (*p* = 0.005), and after exclusion of 17 participants whose baseline sample was taken from March to July inclusive (*p* = 0.0003).

Conclusions: Mean serum 25(OH)D concentration of TB patients increased after resolution of tuberculosis. This phenomenon was associated with evidence of increased baseline activity of CYP27B1 and CYP3A4, but not of CYP24. Differences in mean serum 25(OH)D concentration between the two time points were not explained by differences in use of vitamin D supplements, self-reported sun exposure or season of sampling at follow-up *vs.* baseline. Our findings raise the possibility that vitamin D deficiency may be a consequence, as well as a cause, of active tuberculosis.

### 2.75. Vitamin D Deficiency and Delayed Sputum Smear Conversion in Pulmonary TB in Pakistan

#### Junaid, K.; Rehman, A.; Saeed, T.

Background: Tuberculosis (TB) constitutes a major health problem in a developing country like Pakistan. There are many factors that contribute in development of TB drug resistance and late sputum smear conversion and one of the most important among them is immunity. It has been proven in many studies that Vitamin D has a strong immunomodulator effect and vitamin D receptor (VDR) increases the expression of certain anti-mycobacterial compound in macrophages called cathecidilin. The aim of this study was to compare the degree of vitamin D insufficiency (VDI) and vitamin D deficiency (VDD) in TB patients and delay in sputum conversion for AFB.

Method: Serum 25-hydroxy vitamin D_3_ level was measured in 180 pulmonary TB patients with no co-morbidity. Meanwhile sputum smear conversion for Mycobacterium tuberculosis was also examined after every two weeks till sputum converted negative for AFB.

Results: Results indicate that most of the TB patients had vitamin D deficiency *i.e.*, 50.6% and 45.1% patients had vitamin D insufficiency. Only few patients (3.3%) had sufficient vitamin D level. The 103 cases had delayed sputum conversion and 77 had early sputum conversion. When level of vitamin D was compared with sputum smear conversion time high significant *p*-value (0.00) indicates that level of vitamin D has a significant association with early sputum conversion in pulmonary TB patients.

Conclusion: Most of the TB patients in Pakistan have hypovitaminosis D and that found to be a strong factor in delayed sputum conversion. Further we are also working on to see the role of vitamin D receptor polymorphism in these patients.

### 2.76. Prevalence of Vitamin D Deficiency in Local Adult Females of Pakistan

#### Junaid, K.; Rehman, A.

Background: Many epidemiological studies in recent years suggest that vitamin D is associated with a high risk of osteoporotic fractures, malignancy of breast, colon, prostate and many respiratory diseases. As sun is a rich source of vitamin D so once it was thought that vitamin D deficiency is rare in Asia. But many studies in India proved vitamin D deficiency in healthy subjects. Then later some researchers in Pakistan also reported vitamin D deficiency in some regions of Pakistan. This study was aimed to investigate the prevalence of vitamin D deficiency in adult female of local population in Pakistan and to find out social and genetic factors that may contribute in hypovitaminosis.

Method: For the above said purpose blood samples from the local population has been collected, before sample collection a detailed interview was done to ask about their life style particularly about sun exposure. Renal and liver function test was done for screening any pseudo cause of hypovitaminosis. Serum Calcium and Phosphorus was done as a biomarker of vitamin D deficiency. Serum 25 hydroxy vitamin D estimation was done by EIA method.

Results: It has been observed that in adult female of Pakistan most of them have vitamin D insufficiency and some of them were deficient while serum calcium is not a significant biomarker to check the level of vitamin D. In obese female vitamin D insufficiency is more than non obese. Life style is a more significant factor that contributes in vitamin D insufficiency even in a sun rich country like Pakistan.

Conclusion: Subjects that had vitamin D deficiency not showed any significant clinical sign of any disease so this situation of vitamin D deficiency could be ignore at early stage that may lead to some drastic health effects in later stages that may be other then bone disorders. Further we are working on VDR genotyping of these subjects.

### 2.77. Prevalence, Determinants and Clinical Correlates of Vitamin D Deficiency among UK Adults with Asthma

#### Kilpin, K.; MacLaughlin, B.; Jolliffe, D.A.; Timms, P.M.; Griffiths, C.J.; Martineau, A.R.

Background: Vitamin D deficiency has been reported to be common in patients with asthma, and to be associated with poor control of asthma symptoms in several settings. Data on the prevalence of vitamin D deficiency and its clinical correlates among asthma patients in the UK are lacking. We therefore conducted a study to determine the prevalence of vitamin D deficiency in a cohort of adults with asthma who were screened for participation in a clinical trial in London, UK, and to identify correlates of poor asthma control and low vitamin D status in this population.

Methods: A cross-sectional analysis was performed on a population screened for an asthma clinical trial in London, UK (*n* = 297). Patients were recruited primarily through conducting mailshots from local GP practices, and a number of clinical outcomes and demographic variables were assessed and collected. Multivariate logistic regression was performed to identify potential determinants of vitamin D status and of six clinical markers of asthma control (Asthma Control Test score, British Thoracic Society treatment step, number of exacerbations in preceding 12 months, % predicted FEV1, exhaled nitric oxide concentration and degree of reversibility of airway obstruction following administration of inhaled salbutamol).

Results: The prevalence of vitamin D deficiency (serum 25-hydroxy vitamin D concentration [25(OH)D] <50 nmol/L) was 54%; median serum 25(OH)D concentration was 47 nmol/L. 25(OH)D levels were not found to be associated with any of the six markers of asthma control. Consuming alcohol was found to be associated with improved control (OR 0.37, *p* = 0.009). Correlates of poor asthma control were increased body mass index (BMI) (OR 8.15, *p* = 0.048), increased age (OR 3.00, *p* = 0.003), male gender (OR 2.89, *p* ≤ 0.001) and Black/Black British ethnic origin (OR 4.21, *p* = 0.008), which influenced four of the six markers. The major determinants of vitamin D status were season of sampling, sun-seeking behaviour, use of vitamin D supplements and BMI. Ethnicity and intake of fortified foods were not found to influence 25(OH)D levels.

Conclusion: Vitamin D deficiency was common in this population of patients with asthma, but it did not associate with poor control of asthma symptoms.

### 2.78. The Effect of DBP Polymorphisms on Estimates of Free 25OHD and Its Relationship with 25OHD_3_ Half-Life

#### Jones, K.S.; Bouillon, R.; Assar, S.; Lambrechts, D.; Vanderschueren, D.; Prentice, A.; Schoenmakers, I.

Background: Free-25OHD (f25OHD) is proposed as a biomarker of 25OHD availability to tissues. DBP (Gc) genotype may affect DBP affinity and influence plasma 25OHD and f25OHD and is known to vary by population. We investigated the effect of DBP genotype on estimates of f25OHD (Gc-f25OHD), and on their relationship with 25OHD_3_ t1/2, a measure of 25OHD tissue usage, in populations known to differ in DBP genotype and vitamin D supply.

Methods: Plasma 25OHD (LCMSMS), DBP (radial immunodiffusion) and albumin (Kone Lab 20i) were measured. Genotyping of DBP SNPs (rs7041 and rs4588) was performed (MassARRAY Compact Analyser) and combined genotypes determined. f25OHD was derived from plasma 25OHD, DBP and albumin (Powe, C.E., et al, *Hypertension* 2010, *56*, 758-763). Gc-f25OHD was derived from f25OHD with adjustments for DBP-25OHD affinity by genotype (Chun, R.F., et al, *PLoS One* 2012, *7*, e30773). 25OHD_3_ t1/2 were measured in healthy men in The Gambia (latitude 13°, *n* = 16) and in the UK during late-winter (*n* = 12). Relationships between f25OHD and Gc-f25OHD and 25OHD_3_ t1/2 were investigated with regression by country, and for the countries combined, with country as an independent variable.

Results: Plasma 25OHD was 68 (14) and 30 (11) nmol/L (*p* < 0.0001), plasma DBP, 262 (34) and 271(25) µg/mL (*p* = 0.4), and plasma albumin, 36(2) and 41(2) g/L (*p* < 0.0001) in The Gambia and UK, respectively. There was no difference in 25OHD_3_ t1/2 between countries. f25OHD was (20(4) pmol/L in The Gambia participants compared to 9(3) pmol/L in the UK (*p* < 0.0001). Combined DBP allele frequencies differed between countries (χ2 *p* < 0.0001) (Gc1f: 0.78 *vs.* 0.21; Gc1s 0.19 *vs.* 0.71; Gc2 0.03 *vs.* 0.08 for The Gambia and UK, respectively). Gc-f25OHD (12(6) pmol/L) was higher than f25OHD (9(3) pmol/L) in UK participants (*p* < 0.003), but not in Gambians (20(6) *vs.* (20(4) pmol/L). However, Gc-f25OHD remained higher in Gambian compared to UK participants (*p* = 0.0008). There was no significant relationship between 25OHD_3_ t1/2 and f25OHD (*p* = 0.8) or Gc-f25OHD (*p* = 0.6) in the combined country model, or for the countries analysed separately.

Conclusions: Group differences in f25OHD and Gc-f25OHD were primarily determined by 25OHD status and to a lesser extent by plasma albumin concentration. The Gambian group had a higher frequency of the higher affinity Gc1f genotype and consequently similar f25OHD and Gc-f25OHD. Gc-f25OHD was higher than f25OHD in the UK, which has a higher frequency of the lower affinity Gc genotypes, Gc1s and Gc2. In this study, f25OHD and Gc-f25OHD, as estimates of 25OHD availability, did not predict vitamin D usage as assessed by 25OHD_3_ half-life.

This research is jointly funded by the Medical Research Council (MRC) (programme codes U105960371, U123261351, MC-A760-5QX00) and the Department for International Development (DFID) under the MRC/DFID Concordat agreement.

### 2.79. High Vitamin D Status Is Associated with Common Health Complaints in a Group of Healthy Kuwaiti Women

#### Alyahya, K.O.

Background: The public continues to receive the advice that sustaining a high level of serum vitamin D is important in maintaining good health, regardless of the presence of an illness. Many studies have shown an inverse association between 25OHD levels and a wide range of disorders, thus vitamin D supplementation has been promoted to support good health. Continuous studies in the Middle East, especially in the GCC countries, show extremely low 25OHD levels compared to the rest of the world. This is expected to have a negative impact on the health of the population. Thus, the aim was to assess the association between 25OHD levels and common health complaints in a group of clinically healthy Kuwaiti women.

Methods: The study was conducted between December 2011 and March 2012 and was granted ethical approval. Mothers of students whom were a part of a previous study, and undergraduate female students were invited to participate. Excluded were post-menopausal, younger than 19 and older than 48 yearrs, non-Kuwaitis, pregnant, and breast-feeding women, those with a medical diagnosis, and taking medication or vitamin/mineral supplements/injection for the past 6 months. On the day of examination, consent forms were signed prior to a 20 min interview to collect data including common health complaints. A 15 ml blood sample was collected. Serum samples was separated and shipped to The Doctors Lab (TDL, London, UK) on dry ice to measure 25OHD by radio-immunoassay. Binary logistic regression was used to assess the most common predictors of a low 25OHD (*i.e.*, <50 nmol/L). Statistical analysis was carried out using SPSS, version 17.

Results: A total of 118 healthy Kuwaiti premenopausal women, aged 19–47 years old, were recruited. Median 25OHD was 16.5 nmol/L. Levels >50 nmol/L were obtained by 18.6%, among which 72% had taken a vitamin D injection between 6–48 months prior to the examination. The logistic regression model was able to explain 50% of the variation in 25OHD. The significant predictors were food allergy, neck & shoulder ache, and female infection. Interestingly, women with 25OHD ≥ 50 nmol/L were more likely to complain of food allergies (OR: 15, 95% CI: 3.5–67), neck and shoulder ache (OR: 9.3, 95% CI: 2–44), and female infection (OR: 6, 95% CI: 1.7–21) than women who had levels <50 nmol/L.

Conclusions: Common health complaints were found associated with higher 25OHD levels not with lower levels in a group of Kuwaiti women. This could be due to:

- Middle-eastern women may have a lower upper limit for 25OHD.
- Vitamin D injection in a dose of 600 thousand IU/dose might have adverse effects.

### 2.80. Effect of Cotinine Blood Serum Level on Vitamin D Blood Serum Level Among Women Smokers in the United States

#### Manavi, K.R.; Kukhareva, P.V.; Dong, L.; Alston-Mills, B.P.; Swallow, W.H.

Background: The suffering from lung cancer as the disease becoming the usual cause of cancer mortality has been on the rise rapidly in the world since the 20th century. Results from epidemiological investigations have correlated cigarette smoking with the causation of lung cancer in the 1950s clearly. As we have taken the first step into the 21st century, the frequency of lung cancer has not only been dubious to decline, but the burden of the cancer has shifted from the developed to the less developed countries also. According to International Association of Cancer Registries (IACR), the lung cancer is accountable for 1.2 million new cases annually as well as responsible for 18% of all cancer death worldwide. The suffering from lung cancer as the disease becoming the usual cause of cancer mortality has been on the rise rapidly in the world since the 20th century. Results from epidemiological investigations have correlated cigarette smoking with the causation of lung cancer in the 1950s clearly. As we have taken the first step into the 21st century, the frequency of lung cancer has not only been dubious to decline, but the burden of the cancer has shifted from the developed to the less developed countries also. According to International Association of Cancer Registries (IACR), the lung cancer is accountable for 1.2 million new cases annually as well as responsible for 18% of all cancer death worldwide. The epidemiological data indicate that vitamin D deficiency is relatively common, at least in some parts of the United States and Europe, and that inadequate serum levels of the Vitamin D are associated with an increased risk and poor prognosis of several types of cancer. Cotinine is the main metabolite of nicotine, and its serum or plasma level is a useful marker of tobacco smoking.

Methods: National Health and Nutrition Examination Survey (NHANES) datasets of Gender, Race, Cotinine and Vitamin D from the years 2001–2002, 2003–2004 and 2005–2006 have been used for the purpose of epidemiology study for the relationship between Cotinine and Vitamin D in women among different ethnicities in the United States.

Results: The data analysis from the NHANES 2001–2002, 2003–2004 and 2005–2006 showed the Vitamin D is lowest in active smoker women in whom the cotinine level is high, compared with non-smoker (low cotinine level) and passive-smoker (mid-range cotinine level) groups.

Conclusion: This data analysis has suggested us that race, gender and cotinine play an important factor on Vitamin D level among women smokers, yet more research need to be done to explore more.

### 2.81. Infant Vitamin D Status and Its Impact on Allergy Development: Follow up in the German LINA Cohort Study

#### Junge, K.M.; Geissler, S.; Herberth, G.; Röder, S.; Diez, U.; Borte, M.; Stangl, G.I.; Lehmann, I.

Background: Within the LINA cohort study maternal as well as newborn vitamin D levels were shown to be positively associated with allergic sensitisation and food allergy development within the first two years of life. The aim of the present study was to follow up the study participants investigating whether the effect of vitamin D is restricted to the prenatal period or can also be seen in one or two year old infants independently from the maternal status during pregnancy.

Methods: In total, blood samples of 374 pregnant mothers, 378 newborns, 466 one year old and 304 two years old children from the LINA cohort study (Lifestyle and environmental factors and their Influence on Newborns Allergy risk) were available for serum 25(OH)D_3_ analyses. Information about allergic manifestations during the first 4 years of life as well as confounding factors were obtained from standardized questionnaires filled out by the parents during pregnancy and annually thereafter. Serum IgE levels were analysed according to the same schedule.

Results: The median maternal 25(OH)D_3_ level during pregnancy was 22.19 ng/mL (interquartil range (IQR) 14.40–31.19 ng/mL), the median cord blood 25(OH)D_3_ 10.95 ng/mL (IQR 6.99–17.39 ng/mL). The median 25(OH)D_3_ levels at year one and two were 33.20 ng/mL (IQR 28.20–39.10 ng/mL) and 22.25 ng/mL (IQR 16.9–28.30 ng/mL), respectively. A high correlation was seen between maternal and newborn 25(OH)D_3_ levels (*r* = 0.812, *p* < 0.001), both showing a significant seasonal distribution (*p* < 0.050). A correlation was also seen when we compared 25(OH)D_3_ levels of one and two years old children (*r* = 0.499, *p* < 0.001). However, 25(OH)D_3_ levels at year one showed an independent trend: due to the general rickets prophylaxis within the first year (87% of the children were supplemented with vitamin D at least during the first 3 months), absolute 25(OH)D_3_ was generally higher with almost no variations due to seasonal sunlight exposure. In two year old children 25(OH)D_3_ levels had a similar seasonal pattern seen for pregnancy and cord blood. According to associations with allergic outcomes we could show comparable effects seen earlier within the LINA cohort: there was no evidence that vitamin D has allergy-protective effects. For wheezing, rather an association to higher 25(OH)D_3_ at year or two was observed.

Conclusions: Our study demonstrates that in East Germany region (latitude: 51.3667) the vitamin D status of one year old children is sufficient due to a general rickets prophylaxis, whereas levels of two years old infants drop back to levels similar seen for pregnant mothers. However, according to allergic outcomes our data rather point to a promoting than a protecting effect of vitamin D.

### 2.82. Vitamin D among Polish Centenarians

#### Kupisz-Urbańska, M.; Katarzyna, B.K.; Mossakowska, M.

Background: Vitamin D deficiency is common among elderly people. Among the oldest old it causes muscle strength reduction, may lead to increased number of falls and osteoporotic fractures. Due to pleiotropic vitamin D effects it influences not only physical and mental health status but also the quality of life. The aim of the present study was to evaluate and compare chosen elements of calcium-phosphorous metabolism especially 25-hydroxycholecalciferol (25(OH)D), 1,25-dihydroxycholecalciferol (1,25(OH)D) plasma level among centenarians and 65 years old subjects.

Methods: The study group consisted of 97 centenarians: 81 women aged 99.9 to 108 years (mean age 1013 years). The group of 65 years old subjects consisted of 35 women and 22 men, mean age 66 years. The study was part of Polish Centenarians Program and PhD thesis grant of Polish Ministry of Science. Methods applied in the study included: medical history and physical examination, and also laboratory tests: blood haemoglobin, albumin, protein and creatinine serum levels, calcium, magnesium, inorganic phosphorous serum levels and alkaline phosphatase activity. To achieve the goal of the study 25-hydroxycholecalciferol (25(OH)D), 1,25-dihydroxycholecalciferol (1,25(OH)D) plasma level were also performed.

Results: Mean calcium, magnesium serum levels and alkaline phosphatase activity levels were significantly lower in centenarians than in 65 year-old subjects. Mean serum calcium level was 8.67 mg/dL ± 1.09 in centenarians and 9.46 mg/dL ± 0.48 in 65 year-old subjects (*p* = 0.000), magnesium 1.98 mg/dL ± 0.29 in centenarians and 2.11 mg/dL ± 0.24 in 65 year-old subjects (*p* = 0.003), alkaline phosphatase activity was 258 j.m ± 180 in centenarians and 205 j.m ± 90 in 65 year-old subjects (*p* = 0.006). Organic phosphorous level was slightly lower in centenarians (3.06 mg/dL ± 0.68 compared to 65 year-old subjects (3.14 mg/dL ± 0.52), but the difference was not significant (*p* = 0.09)). It was also shown that in majority of centenarians (87%) plasma 25(OH)D levels were under the laboratory reference range. 13% of centenarians constituted the part of the group who had the plasma—25(OH)D levels in the normal range, in majority they were in the inferior normal range quartile. In 48%—of 65 year-old subjects plasma 25(OH)D were under the laboratory reference range. Statistical significance (*p* < 0.0001) between the plasma 25(OH)D levels in two groups was found. It was also found out that there existed significant differences (*p* < 0.0001) between the plasma 1,25(OH)D levels in centenarians and in 65-year old subjects. Among the majority of younger group (98.1%) plasma 1,25(OH)D levels were in the normal range. In 27% of centenarians plasma 1,25(OH)D levels under the normal range were found.

Conclusions: Insufficient 25(OH)D plasma levels were common among elderly, more significantly among centenarians, which may suggest vitamin D deficiency with age, also among oldest old. In some of the elderly subjects lower plasma 25(OH)D levels coexisting with high alkaline phosphatase activity may indicate developing osteomalacia.Taking into account lower plasma 25(OH)D, 1,25(OH)D and calcium levels among the majority of centenarians, the sufficient vitamin D and calcium supplementation should be systematically applied also in the oldest old.

### 2.83. Prenatal Vitamin D Levels and Adult Schizophrenia: Evidence from A Danish Societal Experiment

#### Raymond, K.; Knop, J.; Heitmann, B.L.

Background: Of late, the influence of prenatal nutrition on the long term health of progeny has received increased scrutiny. The Dtect project, aims to elicit the role of prenatal Vitamin D levels on future health outcomes such as the development of schizophrenia amongst progeny later in life. The first step in the Dtect protocol is to study the potential association between prenatal Vitamin D levels and the development of adult schizophrenia by examining a societal experiment undertaken by the Danish government from 1961 to 1985. During this period, the Danish government mandated the fortification of margarine with vitamin D, 1.25 mg/100 g. In this talk we will analyze and discuss the benefits associated with prenatal consumption of vitamin D fortified foods in terms of the development of schizophrenia amongst the progeny later in life.

Methods: In order to assess the potential health effects of consuming vitamin D fortified foods, we performed a registry based study, taking advantage of the extensive Danish public health registries. At birth, every Dane is assigned a CPR number and information pertaining to date of birth, current living status and gender is stored in the CPR registry. By linking the CPR registry with the National Psychiatric Research Register, we were able to collect information pertaining to every Dane born 2 years prior to and after each change in the Danish fortification policy between 1961 and 1985. Using Age-Period-Cohort models we examined the incidence rate of schizophrenia amongst the birth cohorts prior to and after termination of the fortification policy in 1985. Further subdividing the birth cohorts by month, we were able to examine differences in the incidence rates of schizophrenia by season of birth. This is of particular importance due to the seasonal dependence on dietary sources of vitamin D in Denmark. To test the hypothesis of an association between the prenatal exposure to vitamin D fortified foods and the risk of the progeny developing schizophrenia later in life, Cox regression models were fit. Cox regression models were also used to test the hypothesis of interactions between season of birth and exposure to vitamin D fortified foods.

Results: The results of the Cox regression analysis indicated that there was a significant interaction between the season of birth and exposure to vitamin D fortified margarine amongst children born between 1983 and 1987. Specifically, in regards to children born after the cessation of vitamin D fortification, children born from May–July have an increased risk, (hazard ratio 1.40 (1.05,1.85)), of developing schizophrenia compared to children born between November and January. In contrast, there was no evidence to suggest a similar association amongst the children born while the vitamin D fortification policy was in place.

Conclusion: The results of our study suggest that the vitamin D fortification policy enacted in Denmark until June 1st, 1985 had a protective effect amongst children born during the spring in regards to later development of schizophrenia.

### 2.84. The Effects of 25(OH)_2_D_3_ on Vascular Endothelial Cell Proliferation and Gene Expression

#### Al-Harbi, L.; Brameld, J.M.; Parr, T.

Background: It is well known that calcitriol, the active form of vitamin D, is essential for bone development and maintenance. However, in recent years, it has been shown that the role of vitamin D goes beyond calcium homeostasis and skeletal health. Numerous studies have reported that vitamin D deficiency plays a significant role in the regulation of blood pressure and cardiovascular health. Moreover, it has been recognized that vitamin D has a wide range of biological functions, including cell differentiation, inhibition of cell growth and regulation of gene expression. However, it is unclear whether the non-active form of vitamin D_3_, 25(OH)_2_D_3_, has any effects on endothelial cells. This study aimed to investigate the effect of 25(OH)_2_D_3_ on the growth and gene expression of vascular endothelial cells.

Methods: Human umbilical vein endothelial cells (HUVECs) were cultured in EBM-2 media with 2% foetal bovine serum containing increasing concentrations (0, 10^−13^, 10^−11^, 10^−9^ or 10^−7^ M) of 25(OH)_2_D_3_ for 24 h, and DNA contents or key marker genes of blood pressure regulation were measured.

Results: The highest supraphysiological concentration (10^−7^ M) of 25(OH)_2_D_3_ significantly decreased cell numbers (*i.e.*, DNA content) relative to control (*p* < 0.001), possibly suggesting toxic effects at this highest concentration. A high physiological concentration (10^−11^ M) appeared to increase endothelial Nitric oxide synthase (eNOS) mRNA relative to control (*p* = 0.06). In contrast, all concentrations (10^−13^–10^−7^ M) of 25(OH)_2_D_3_ significantly decreased endothelin-1 (EDN-1) mRNA relative to control (*p* = 0.005).

Conclusions: Despite it supposedly being an inactive metabolite of vitamin D, 25(OH)_2_D_3_, appears to have biologically relevant effects on cultured HUVECs. The results demonstrate that physiological doses (10^−11^ M) of 25(OH)_2_D_3_ alter gene expression, increasing eNOS mRNA and decreasing EDN-1 mRNA. This would be expected to result in a potent vasodilation and therefore reduced blood pressure.

### 2.85. Use of Diverse Food Matrices Do not Affect the Bioavailability of Vitamin D_3_ and Subsequent 25OHD Status in Women

#### Tripkovic, L.; Wilson, L.; Hart, K.; Smith, C.P.; Bucca, G.; Penson, S.; Chope, G.; Hyppönen, E.; de Lusignan, S.; Berry, J.L.; Lanham-New, S.

Background: Maintaining a healthy vitamin D status is problematic within the UK, largely due to a combination of two factors: required UV exposure for dermal production of vitamin D is limited by seasonality and there are few vitamin D-rich foods readily available within the diet. With the implications of a low vitamin D status known to include poor bone and muscle function, establishing strategies to improve the availability of vitamin D sources to the population is vital. Whilst food fortification is a sustainable solution for the prevention of vitamin D deficiency, the food industry needs to determine suitable means of carriage if it is to maximise the effectiveness of fortification. Thus the aim of this sub-study of the D_2_–D_3_ Study (a 12-week, double blind, food fortification RCT) was to compare the efficacy of 15 µg/day Vitamin D_3_ in raising 25OHD levels in both Caucasian and South Asian women during the winter and early spring months via the use of two differing food matrices, a juice and a biscuit.

Methods: In this sub-study, a cohort of 137 healthy women (Caucasian n99, South Asian n38) with a mean age of 43.4 ± 12.7 years and BMI of 23.8 ± 3.7 kg/m^2^ were recruited to the D_2_–D_3_ Study over the course of two consecutive winters (October 2011–January 2012 and October 2012–January 2013). Participants were required to consume 1 juice and 1 biscuit per day for 12 weeks, with the vitamin D_3_ contained within one of the products. The products were assigned on a randomised basis, with investigators and participants blinded. At baseline, week 6 and week 12 study visits, anthropometric measurements and a fasting blood sample were taken and compliance assessed.

Results: At baseline, there were no significant differences between the Juice (D3J) and Biscuit (D3B) groups for total serum 25OHD levels: D3J 49.17 ± 26.45 nmol/L, D3B 51.21 ± 31.07 nmol/L. Subsequently, no significant differences were found between groups for total serum 25OHD levels at week 6 (mid-intervention, D3J 78.76 ± 23.88 nmol/L, D3B 79.49 ± 26.66 nmol/L) and week 12 (end of intervention, D3J 83.92 ± 23.28 nmol/L, D3B 85.95 ± 27.96 nmol/L). When compared to baseline, both the D3J and D3B groups achieved similar significant increases in total 25OHD levels over the duration of the intervention—D3J Δ30.83 ± 23.43 nmol/L, *p* < 0.001; D3B Δ30.81 ± 21.47 nmol/L, *p* < 0.001. The mean percentage increase in total serum 25OHD over the intervention was 106% for D3J and 103% for D3B (ns between groups). There was no significant difference in compliance for the two products between groups; mean percentage completion of consumption for the juice was 93.3% and 94.9% for the biscuit.

Conclusions: This study has important implications for the future of food fortification in helping to raise the vitamin D status of the population. Unequivocal evidence has been provided that shows the use of two distinctly diverse food matrices with differing nutritional compositions—such as juice and biscuits—do not have a detrimental impact upon the bioavailability of vitamin D_3_ and thus the resultant 25OHD levels. By proving the effectiveness of food fortification in this manner, the opportunity now exists to increase the diversity and range of foods available to the population that could provide adequate vitamin D. Population groups typically resistant to traditional methods of supplementation (*i.e.*, adolescents) could vastly benefit from a flexible approach to food fortification with the implementation to both staple and appropriate snack food.

### 2.86. The Nutritional Impact of Replacing Cows’ Milk with a Vitamin D Fortified Growing-up Milk, in the Diets of Young UK Children

#### Pean, J.; Olivier, L.; Warren, J.M.; Lluch, A.

Background: The Reference Nutrient Intake (RNI) of young children between the ages of 6 months to 3 years is 7 µg/day (Dietary Reference Values for Food Energy and Nutrients for the United Kingdom, Department of Health, HMSO: London, 1991). The UK National Diet and Nutrition Survey (NDNS) showed that the average UK toddler is getting just 27% of their daily dietary intake of vitamin D (Bates, B., *et al.*, National Diet and Nutritional Survey: Headline results from Years 1 and 2 (combined) of the Rolling Programme (2008/2009-2009/2010); HMSO: London, 2010). This has spurred discussion on the best way to improve the vitamin D status of children. A universal approach to vitamin D supplementation has recently been suggested by the Chief Medical Officer (Davies, D.C. Annual Report of the Chief Medical Officer 2012, “Our Children Deserve Better: Prevention Pays” Department of Health, HMSO: London, 2013), but targeted supplementation strategies have had limited success (Davies, H.H., et al. *Arch. Dis. Child*. 2010, doi: 10-1136/adc. 2010, 191627), which questions the cost effectiveness of this approach. Canada has mandatory fortification of vitamin D to cow’s milk. Maguire and colleagues showed that most Canadian children could maintain their vitamin D status by consuming vitamin D fortified milk (Maguire, J.L., *et al.*, *Pediatrics* 2013, *131*, 739-753), suggesting this could be a more effective way of increasing and maintaining children’s vitamin D status. In the UK cows’ milk contains trace amounts of vitamin D whilst growing-up milk (GUM) contains around 1.7 µg/100 mL. This simulation study aimed to assess the impact of replacing non fortified cow’s milk with vitamin D fortified GUM in the diets of young UK children.

Methods: Analyses were based on individual dietary data from NDNS, 2008–2011. Children consuming cows’ milk only and aged 18–36 months (*n* = 159) were divided into 2 subgroups, 18–23 months (*n* = 41) and 24–36 months (*n* = 118). Simulations were conducted using Creme Food^®^ software and cow’s milk was replaced with GUM in individual diets, either at observed consumption (scenario 1) or with an intake of 300 mL/d (scenario 2). Nutritional intakes from the observed data and after simulations were compared and evaluated against UK nutrient recommendations.

Results: In both scenarios and subgroups, replacing cows’ milk with GUM led to a significant increase in vitamin D intakes (+278% to +356%). Mean intakes of vitamin D were 1.82 ± 0.28 µg/day (mean ± SEM) for cows’ milk consumers aged 18–24 m, and 1.71 ± 0.15 µg/day for cows’ milk consumers aged 24–36 m. This corresponds to 4.1% of the 18–24 m having intakes of vitamin D at or above the RNI, and 2.8% of the 24–36 m. After replacement from cows’ milk by GUM at observed consumption (scenario 1), mean intakes respectively corresponded to 48.1% and 43.0% of the children having intakes of vitamin D at or above the RNI. For scenario 2, 24.1% of 18–24 m children & 23.6% of 24–36 m children had intakes at or above the RNI level.

Conclusion: The role of vitamin D fortified foods in the UK, especially in young children, could be a useful mechanism for increasing the nutritional intake of this essential nutrient. From this simulation we conclude that a daily consumption of 300 mL fortified GUM can help increase nutritional intakes of vitamin D in young UK children.

### 2.87. High Prevalence of Vitamin D Deficiency in Patients with Neurological Disorders: Results from a Hospital-Based Study

#### Triggiani, L.; Barracchini, A.; Minisola, G.

Background: Recent researches showed that normal Vitamin D levels are correlated with physiologic process of the Nervous System and that Vitamin D deficiency plays a role in numerous disorders of the Nervous System.

Methods: Vitamin D levels were investigated in patients of a Rehabilitation Day-Hospital in order to estimate the prevalence of Vitamin D deficiency and its possible linkage with neurological disorders. All patients hospitalized between 1 April 2010 and 31 March 2013 underwent blood sample to test Vitamin D (25OH-D) level. The range of the laboratory were: normality between 31 and 100 ng/mL, moderate deficient state between 20 and 30 ng/mL, while levels below 20 ng/mL indicated a deficient state. Two subgroups of patients, respectively with neurologic or osteoarticular disorders, were considered. Logistic regression analysis was performed.

Results: 520 patients underwent blood test, there were respectively 272 males (52.3%) and 248 females (47.7%). Mean age was 59.3 ± 16.3: respectively 58.4 ± 17.2 in males and 60.4 ± 15.2 in females (*p* = 0.15). There were 356 patients (68.5%) with neurological disorders and 164 patients (31.5%) with osteoarticular disorders. The two subgroups were homogeneous by age (*p* = 0.34) but not by sex (*p* = 0.03): there was a higher number of males among patients with neurological disorders (M* vs.* F: 55.6* vs.* 44.4%) and a higher number of females among patients with osteoarticular disorders (M* vs.* F: 45.1* vs.* 54.9%). Mean level of Vitamin D was 19.2 ± 12.6 ng/mL, respectively 19.2 ± 12.3 ng/mL in men and 19.1 ± 12.9 ng/mL in women (*p* = 0.86). Mean level of vitamin D was lower in patients with neurologic disorders with respect to patients with osteoarticular disorders (18.0 ± 11.7 ng/mL* vs.* 21.8 ± 13.9 ng/mL; *p* = 0.001). Deficient state was detected in 305 patients (58.6%) who showed a mean level of 10.4 ± 4.5 ng/mL, without differences between sexes (*p* = 0.64). The prevalence of Vitamin D deficiency was higher in patients with neurologic disorders with respect to patients with osteoarticular disorders (62.1% * vs.* 51.2%, OR 1.56, 95% CI 1.05–2.31; *p* < 0.05). The highest prevalence of Vitamin D deficiency was detected in patients with cerebrovascular disorders (44.8%, 95% CI 38.1–51.6), Parkinson’s disease (10.4%, 95% CI 6.7–15.2), or spinal lesions (9.5%, 95% CI 6.0–14.2). Linear regression analysis showed a significant association between Vitamin D levels and the type of disorder (*p* < 0.001) or the age of the patients (*p* < 0.001).

Conclusions: The results of the present study may have been biased by the type of the study population (hospital-based) and the small size of the sample. However, the frequent association between Vitamin D deficiency and neurologic disorders indicates the role of this Vitamin in metabolic processes different from those of the osteoarticular system and specifically in physiologic and pathological processes of the Nervous System. The high prevalence of Vitamin D deficiency in patients with neurological disorders suggests that it is not only related to the age or the degree of disability but could be disease-specific. Vitamin D deficiency should be investigated routinely in clinical practice as the detection of this parameter may have important therapeutic implications.

### 2.88. Two Site Comparison of a Liquid Chromatography Mass Spectrometry and a Competitive Binding Assay for Measurement of 25OH Vitamin D

#### Blake, L.; McCormack, W.; Norton, C.; Jakeman, P.; O’Shea, P.

Background: Our two laboratories compared the results of 25OH Vitamin D measurements on serum samples using two analytical methods. Our objective was to compare these two methods in routine settings.

Methods: Liquid chromatography/mass spectrometry (LC/MS) is used as the method of measuring 25OH Vitamin D in the hospital setting in Galway. The MassChrom 25-OH-Vitamin D_3_/D_2_ kit (Chromsystems Munich) is used with an Agilent 1290 UPLC and 6440 Triple Quad LC/MS to measure 25OH Vitamin D_3_ and 25OH Vitamin D_2_ in patients serum samples,. The sum of these parameters is reported as 25OH Vitamin D. On-line solid phase extraction after protein precipitation is employed. The Elecsys Vitamin D Total (Roche Diagnostics, Mannheim, Germany) for measurement of 25OH Vitamin D is used in the research laboratory in the University of Limerick. This assay employs a competitive test principle using recombinant Vitamin D Binding Protein (VDBP) as a capture protein to bind 25OH Vitamin D_3_ and 25OH Vitamin D_2_.

Results: 98 patient, and 97 study participant, serum samples were assayed at both sites. For all samples (*n* = 195) the correlation coefficient was 0.94 between the assays. Passing Bablock regression analysis yielded a slope of 1.045; 95% confidence interval (CI) 1.00–1.03; intercept, 6.2 nmol/L (95% CI 9.38 3.00). For samples with 25OH Vitamin D_2_ and 25OH Vitamin D_3_ detected (*n* = 32) by the LC/MS method, the correlation coefficient was 0.92 between the assays. Passing Bablock regression analysis yielded a slope of 0.964 (95% CI 0.750–1.180); intercept, 7.5 nmol/L (95% CI 18.1 3.3). For samples with only 25OH Vitamin D_3_ detected by the LC/MS method (*n* = 163), the correlation coefficient was 0.94 between the assays. Passing Bablock regression analysis yielded a slope of 1.043 (95% CI 1.000–1.103); intercept, 5.3 (95% CI 8.7 3.0).

Conclusions: When compared across two sites, the Roche Elecsys Vitamin D Total assay correlates well with the MassChrom 25-OH-Vitamin D_3_/D_2_ kit on the Agilent platform.

### 2.89. Effect of Food and Vitamin D Supplements on the Serum 25-Hydroxy Vitamin D Concentration in Children between October and April in a Northern Country

#### Brekhoff, L.; van der Gaag, E.J.

Background: The main source of vitamin D for people is exposure to sunlight (Holick, M.F. *J. Investig. Med.* 2011, *59*, 872-880. Webb, A.R. *Prog. Biophys. Mol. Biol.* 2006, *92*, 17-25). However; in northern countries such as the Netherlands; the skin only synthesizes vitamin D_3_ between April and October (Holick, M.F. *J. Investig. Med.* 2011, 59, 872-880. Weggemans, R.M., et al. *Eur. J. Clin. Nutr*. 2009, *63*, 1455-1457). Because of the importance of vitamin D for many functions in the human body and the limited vitamin D synthesis in the skin between October and April; it is important to have another source of vitamin D during these months. Since vitamin D is also obtained from dietary sources and vitamin D supplements; this study investigated the influence of food and vitamin D supplements on the serum 25-hydroxy vitamin D (25(OH)D) concentration in children in the Netherlands between October and April.

Methods: Children aged 1–18 years who visited the general pediatrician with a complaint whereby serum 25(OH)D concentration was determined, were selected. The intake of vitamin D was calculated based with a dietary questionnaire.

Results: 51.1% of the 174 children had a serum 25(OH)D concentration below 50 nmol/L, 9.2% had a serum 25(OH)D concentration below 30 nmol/L. Adolescents showed lower concentrations compared to younger children. There was a positive correlation between the total amount of vitamin D obtained from food and the serum 25(OH)D concentration (*r* = 0.218, *p* = 0.004). The intake of milk contributed more to the serum 25(OH)D concentration compared to the intake of artificial supplementation, butter or fish. There was a positive trend between the intake of whole milk and the serum 25(OH)D concentration (*r* = 0.116, *p* = 0.127).

Conclusion: In the absence of vitamin D synthesis by sunlight, vitamin D obtained from food has a significant influence on the serum 25(OH)D concentration in children. Therefore, we should pay more attention to food as a natural source of vitamin D.

### 2.90. Serum 25-Hydroxy Vitamin D Concentration, Chronic Lower Respiratory Disease and Cardiac Outcomes: Findings from a National Prospective Study of 15,772 Adults in the United States

#### Liu, L.

Background: Chronic lower respiratory disease (CLRD), which includes asthma, bronchiectasis, and chronic obstructive pulmonary disease, has surpassed stroke as the third leading cause of death in the United States (U.S) since 2010. Although there are several approaches to treat CLRD, such as anti-infections, anti-inflammations and antibiotics, the merging drug resistance and the complexity of CLRD limit these clinical therapies. Thus, additional ways to prevent the risk of CLRD are needed. Recent evidence suggests that vitamin D may have a wide range of protective effect on human health. The present study aims to examine the associations of serum 25-hydroxy vitamin D levels [25(OH)D, a biomarker of vitamin D in blood] with CLRD and outcomes using data from a large-scale prospective cohort study in the U.S.

Methods: A total of 15,772 (M: 7,414, F: 8,358) subjects aged ≥20 year old who participated in the U.S. third National Health and Nutrition Examination Survey, had measures of serum 25(OH)D at baseline (1988–1994), and had been followed up through December of 2006 were analyzed. Causes of death were classified using ICD-10 code, CLRD (ICD-10: J40–J47), total respiratory disease (J00–J99) and cardiovascular disease (CVD, I00–I78). Serum 25(OH)D levels were classified as normal (≥30 ng/mL), insufficiency (20–29 ng/mL), and deficiency (<20 ng/mL). Cox’s proportional hazards regression models were used in multivariate analyses.

Results: At baseline, of the total participants, 1,850 had CLRD (13.0%), and 1,295 had CVD (5.8%). Subjects who had 25(OH)D deficiency had significantly higher prevalence of CLRD (14.9% *vs.* 12.3%, *p* = 0.02), and CVD (7.0% *vs.* 4.1%, *p* < 0.001) than those with normal 25(OH)D levels. In an average 13-year (SD: 4.1) follow-up, 167 died from CLRD (1.0%), 370 from total respiratory disease (1.8%), 1,738 from CVD (7.3%), and 3,950 from all causes (17.4%). After adjustment for age, sex, race, smoking status, seasons of serum 25(OH)D measures, blood pressure, dyslipidemia, dysglycemia and baseline CLRD and CVD, multivariate Cox’s regression models showed that serum 25(OH)D deficiency were significantly associated with risk of death from CLRD, total respiratory disease, CVD and all-causes. The population attributable risks of vitamin D deficiency was 24.1% for death from CLRD, 16.4% from total respiratory disease, 7.4% from CVD, and 7.3% from all-cause mortality.

Conclusion: Using a nationally representative study sample, this study is the first to indicate that decreased serum 25(OH)D levels significantly predict the risk of death from CLRD and total respiratory disease. This study adds new evidence to the research body of vitamin D deficiency in relation to increased risks of CLRD, CVD and all-cause mortality.

### 2.91. No Association between Serum 25(OH) Vitamin D and Symptoms of Depression and Anxiety in Adult Danes

#### Husemoen, L.; Ebstrup, J.; Thuesen, B.; Skaaby, T.; Jørgensen, T.; Linneberg, A.

Background: Mental health has become a major public health issue, and mental diseases such as depression and anxiety affect an increasing number of Danes. Vitamin D receptors and vitamin D metabolizing enzymes are present in the brain and in the central nervous system at sites responsible for regulation of emotions and behaviour. Also, there is evidence suggesting that vitamin D affects the biosynthesis of numerous neurotransmitters and neurotrophic factors relevant for mental health. This raises the hypothesis that low vitamin D is related to poor mental health in the adult general population. Our aim was to examine the association between serum 25(OH) vitamin D and symptoms of depression and anxiety.

Methods: Data are from three Danish study populations (Health2008, Health2006, and Inter99) including a total of 5430 adults aged 18–64 years retrieved from the general population. Serum 25(OH) vitamin D concentrations were measured on fasting blood samples by either HPLC or a chemiluminescence immunoassay. Symptoms of depression and anxiety were assessed by the Symptom Check List (SCL)-90-R. The depression and anxiety symptom scales consists of 13 and 10 questions, respectively, with answer categories ranging from zero (not at all) to four (very much). Average scores ranging from zero to four were calculated. High risk of symptoms of depression and anxiety, respectively, were defined as scores >90 percentiles. Data are cross-sectional and were analysed by logistic regression and quantile median regression analyses adjusted for potential confounders (sex, age, history of chronic disease, lifestyle and socioeconomic factors, and seasonality). Quantile regression is less sensitive to distribution assumptions, and can be considered a non-parametric equivalent of linear regression. Fixed effect meta-analyses were used to combine data from the three individual study populations.

Results: Median serum 25(OH) vitamin D concentrations were 70.3, 41.9, and 51.0 nmol/L in the Health2008, Health2006, and Inter99 study populations, respectively. Median scores were 0.23 for depression and 0.10 for anxiety in all three study cohorts. High risks of symptoms of depression and anxiety were not associated with low serum 25(OH)D in sex- and age-adjusted meta-analyses. The odds ratio (OR) and 95% confidence interval (CI) per 10 nmol/L serum vitamin D were 0.97 (0.93–1.01) for depression (*p* = 0.10), and 1.01(0.97–1.05) for anxiety (*p* = 0.56). Also no associations were observed when analysing data by quantile median regression. The sex- and age-adjusted beta coefficients and 95% CI were 0.00 (−0.01–0.00) for depression (*p* = 0.14) and 0.00 (0.00–0.00) for anxiety (*p* = 1.00). Similar results were obtained in analyses adjusted for potential confounders, except for the logistic regression analysis with anxiety, which became statistical significant (OR (95% CI) = 1.05 (1.00–1.10), *p* = 0.031). However, the estimated effect was in the opposite direction of what we hypothesised.

Conclusions: Our results suggest that low serum 25(OH) vitamin D is not associated with symptoms of depression and anxiety in adult Danes from the general population.

### 2.92. The Effect of Vitamin D_3_ Supplementation on 25OHD Status, Blood Pressure and Blood Lipid Concentrations: A Pre-Menopausal vs. Post-Menopausal Comparison

#### Wilson, L.; Hart, K.; Robertson, F.; Griffin, B.; Smith, C.P.; Bucca, G.; Penson, S.; Chope, G.; Hyppönen, E.; Berry, J.L.; Lanham-New, S.; Tripkovic, L.

BACKGROUND: Epidemiological research has shown a close association between vitamin D deficiency (25OHD < 25 nmol/L) and cardiovascular mortality and morbidity. However, a causal relationship between vitamin D status and cardiovascular disease (CVD) risk factors, such as hypertension or an atherogenic lipid profile, has not been established. Post-menopausal (PoM) women are at an increased risk of CVD compared to pre-menopausal (PrM) women. Therefore this study aimed to compare the response to vitamin D_3_ supplementation between PrM and PoM women, with respect to changes in 25OHD status and key CVD risk factors.

METHODS: A total of 146 Caucasian women (n90 PrM, n56 PoM) were recruited onto the D_2_–D_3_ Study and randomised to receive either placebo or vitamin D_3_ (15 μg/600 IU) daily for 12-weeks. Anthropometrics, blood pressure and a fasted blood samples (for 25OHD and lipid analysis) were collected from each participant at week 0 (baseline) and week 12.

RESULTS: At baseline, the PrM women had a significantly lower body mass index (BMI) and blood pressure (BP) than the PoM women (22.9 ± 3.1 vs 24.7 ± 3.8 kg/m^2^, 116 ± 12/76 ± 9 mmHg *vs.* 128 ± 17/83 ± 10 mmHg respectively; *p* < 0.001), and significantly lower cholesterol, high-density lipoprotein (HDL) and low density lipoprotein (LDL) levels (5.1 ± 0.9 *vs.* 6.0 ± 1.0 mmol/L, 1.81 ± 0.34 *vs.* 1.91 ± 0.46 mmol/L, 2.89 ± 0.77 *vs.* 3.62 ± 0.89 mmol/L respectively; *p* < 0.001). In the placebo group there was a significant decrease in 25OHD status between baseline and week-12 in both PrM and PoM women (PrM n32: −16.5% ± 12.8%, PoM n16: −12.7% ± 7.0%; *p* < 0.001), whereas in the vitamin D_3_ group there was a significant increase in 25OHD status (PrM n58: 28.6% ± 20.0%, PoM n40: 32.6% ± 25.2%; *p* < 0.001). The percentage change in 25OHD status within each group was not significantly different between the PrM and PoM women. The placebo group also saw a significant increase in BMI and waist circumference between baseline and week 12 in PrM women only (23.7 ± 3.4 *vs.* 24.0 ± 3.6 kg/m^2^, 78.9 ± 10.0 *vs.* 81.0 ± 10.2 cm respectively, *p* < 0.04), whereas in the vitamin D_3_ group, both PrM and PoM women had a significant increase in triglycerides (0.98 ± 0.44 *vs.* 1.15 ± 0.73 mmol/L, 1.11 ± 0.53 *vs.* 1.25 ± 0.47 mmol/L, *p* < 0.01). When looking at absolute change from baseline, the only correlation was a weak positive correlation between change in 25OHD status and change in BMI within the PrM women in the vitamin D_3_ group (28.6% ± 20% and 0.31% ± 2.4% respectively, *r* = −0.265, *p* < 0.050).

Conclusion: These results suggest that changes in 25OHD status, BP and blood lipids in response to vitamin D_3_ supplementation are not affected by menopausal status, as any significant changes were seen in both PrM and PoM groups. The increase in triglycerides within the vitamin D_3_ treatment group is of interest because, although the study did not shift participants above the clinically “at-risk” cut off of 1.7 mmol/L, the results contradict previous literature which, although non-significant, have shown a reduction in triglycerides. The data also contradicts previous data suggesting that BMI is negatively correlated with 25OHD status which warrants further investigation, however the mean change in BMI was minimal (0.31%) and the correlation was weak so although the finding was statistically significant, it may not be of clinical importance.

### 2.93. Recent Clinical Studies on Vitamin D Status in Ireland Interpreted in the Light of the IOM 2011 Report: The Dublin Vitamin D Group

#### McKenna, M.; Murray, B.; Kilbane, M.T.; O’Keane, M.; Morrin, M.; Molloy, E.; McCarthy, R.; Onwuneme, C.; McAuliffe, F.; McGowan, J.; Toher, C.; Murphy, N.; Carroll, A.; Flynn, M.

Background: We have been conducting clinical studies on vitamin D status since the 1970s. One of our early observations was to note the primacy of oral intake over sunlight exposure in both the prevention and correction of hypovitaminosis D. We have demonstrated immense improvement in vitamin D status since that time, due to ready availability of low dose vitamin D supplements and due to the increasing fortification of foodstuffs with vitamin D, starting with the fortification of milk in the mid-1980s. Despite these interventions, a substantial portion of our population is at-risk of suboptimal vitamin D status. The only at-risk group in whom a national strategy has been implemented is infants: It is recommended that all infants, from birth to 12 months, whether breastfed or formula fed, be given a daily supplement of 5 μg (200 IU) vitamin D_3_ by providing a supplement containing vitamin D_3_ exclusively. The recent IOM 2011 Report on Dietary Reference Intakes for Calcium and Vitamin D for North America is apt for the Irish population by providing the most comprehensive evidence-based report on vitamin D.

Methods: Since 2007, we have conducted studies of vitamin D status by measuring serum 25-hydroxy vitamin D (25OHD) in: preterm infants shortly after birth (*n* = 274) and after 15 weeks (*n* = 148); paired term infants and mothers at birth (*n* = 39); children and adolescents from age 1 to 18 years (*n* = 252); mothers during pregnancy and infants at birth divided according to winter birth (*n* = 30) and summer birth (*n* = 30); pregnant mothers and ethnicity (*n* = 116); patients attending an osteoporosis clinic (*n* = 100); patients with cystic fibrosis (*n* = 114); patients with multiple sclerosis (*n* = 331) compared with healthy relatives (*n* = 229) during winter months in 3 different regions in Ireland; and patients with psoriasis undergoing phototherapy during the winter months (*n* = 29) compared with controls (*n* = 29). Serum 25OHD levels were interpreted according the IOM report as follows: a 25OHD level below 30 nmol/L (12 ng/mL) was defined as at-risk of deficiency, a level of 30-to- <50 nmol/L (12–20 ng/mL) as within the range of adequacy, and ≥50 nmol/L (20 ng/mL) as sufficient; and >125 nmol/L as at-risk of harm.

Results: Prevalence for vitamin D status (25OHD nmol/L < 30, 30–50, > 50–125, > 125) was noted for the following groups: preterm at 3 weeks (14%, 65%, 21%, 0%); preterm at 15 weeks on 400 IU/day (3%, 11%, 86%, 8%); pregnant women at term (21%, 34%, 45%, 0%) with cord blood at term (59%, 28%, 13%, 0%); children ranging from 1–18 years (22%, 33%, 45%, 0%); osteoporosis on 400–800 IU/day (1%, 8%, 91%, 2%); and cystic fibrosis on 400–1600 IU/d (23%, 28%, 49%, 3%); healthy adults in winter (21%, 46%, 33%, 0%); and multiple sclerosis in winter (40%, 32%, 28%, 1%). Season had a distinct effect on vitamin D status during pregnancy but not on cord blood. In the ethnicity study during pregnancy, the proportion at risk of deficiency was significantly higher among both Middle-Eastern/North African (88%) and Sub-Saharan African women (68%) than Irish women (36%). Phototherapy increased 25OHD levels from 58 (range: 22–115) nmol/L up to 126(79–279) nmol/L.

Conclusions: Vitamin D status in Ireland has improved immensely in Ireland over the past 40 years, but targeted public health policies of low-dose supplementation are needed to address at-risk groups, and mandated fortification to safe levels should maintain vitamin D adequacy in the population.

### 2.94. Evaluation of Vitamin D Status in Relation to Immune Response in Children with Lower Respiratory Tract Infections

#### Gori, M.; Vierucci, F.; Fanos, M.; Del Pistoia, M.; Erba, P.; Saggese, G.

Background: In addition to its known skeletal effects, vitamin D plays a role in the regulation of immune response. Only a limited number of studies examined the relationship between vitamin D status and the prevalence and severity of respiratory infections in children. The aim of this study was to assess vitamin D status in a group of children and adolescents with lower respiratory tract infections and to evaluate the relationship between 25-hydroxy vitamin D (25-OH-D) levels and the severity of infection.

Methods: We enrolled 87 patients (0.1–17.5 years) admitted to the Pediatric Department, University of Pisa, Italy between January 2011 and April 2012 for lower respiratory tract infections (LRTI) (bronchiolitis: *n* = 30; wheezing: *n* = 15; pneumonia: *n* = 42). We also enrolled a control group of children admitted to the Pediatric Department due to non-infectious diseases (*n* = 60). Cases and controls were distributed into age groups (group A: 0.1–1.9 years; group B: 2.0–5.9 years; group C: ≥6 years). Vitamin D endocrine system was evaluated in all children: 25-OH-D (25-OH-D125I RIA Kit, DiaSorin), 1,25-dihydroxy-vitamin D (1,25-OH_2_-D) (1,25-OH-D125I RIA Kit, DiaSorin), Parathormone (PTH) (N-tact PTH SP Irma Kit, DiaSorin^®^), calcium and phosphorus levels were measured following blood withdrawal. Vitamin D status was defined as follows: deficiency, 25-OH-D < 20 ng/mL; insufficiency, 20 ng/mL < 25-OH-D < 30 ng/mL; hypovitaminosis, 25-OH-D < 30 ng/mL; sufficiency, 25-OH-D ≥ 30 ng/mL. Severity of infection was evaluated with specific scores and clinical criteria.

Results: Infants younger than 2 years with LRTI (group A) had lower 25-OH-D levels than controls (23.93 ± 16.39 ng/mL *vs.* 32.40 ± 14.31 ng/mL; *p* = 0.05) and were less likely to receive vitamin D supplementation (45.2% *vs.* controls 75.0%, *p* = 0.03). 1,25-OH_2_-D levels were higher in children with LRTI than in controls in all age groups (group A: 64.68 ± 34.75 pg/mL *vs.* controls 31.54 ± 18.66 pg/mL; *p* = 0.0001; group B: 58.07 ± 31.64 pg/mL *vs.* controls 39.75 ± 18.38 pg/mL; *p* = 0.02; group C: 53.75 ± 20.66 pg/mL *vs.* controls 32.40 ± 22.44 pg/mL; *p* = 0.03). A higher prevalence of severe bronchiolitis was observed in children with hypovitamosis D (25-OH-D < 30 ng/mL) than in children with vitamin D sufficiency (87.5% *vs.* 12.5%, *p* = 0.04). Vitamin D status was not related to the severity of wheezing. We observed a higher prevalence of dyspnea in children with pneumonia and vitamin D deficiency compared to subjects with vitamin D levels ≥20 ng/mL (85.6% *vs.* 14.3%, *p* = 0.01). Disorders of calcium and phosphorus metabolism were occasionally observed in children with LRTI (7 cases (bronchiolitis: *n* = 4; wheezing: *n* = 2; pneumonia: *n* = 1) of secondary hyperparathyroidism (PTH ≥ 65 ng/L), 2 of which showed rickets (wheezing: *n* = 1; pneumonia: *n* = 1)).

Conclusions: The results of this study suggest an important role for vitamin D in the modulation of immune response in children with lower respiratory tract infections. The report of elevated 1,25-OH_2_-D levels in children with LRTI is of undefined significance and may merit further consideration in future studies.

### 2.95. Vitamin D: The Need for Public Health Awareness about Too Much as well as Too Little

#### Kilbane, M.T.; O’Keane, M.; Morrin, M.; Flynn, M.; McKenna, M.J.

Background: Vitamin D plays a fundamental role in bone metabolism and calcium homeostasis. A wide range of non-skeletal conditions have been associated with vitamin D deficiency, but evidence for a causal link is inconclusive and inconsistent. In view of the concerns that unsubstantiated claims were being made concerning the levels of vitamin D intake needed for health, the governments of US and Canada commissioned a report by the IOM. The 2011 IOM report concluded (i) that the estimated average vitamin D requirement from all dietary sources is 10 µg (400 IU) daily in those with minimal sunlight exposure, corresponding to a median serum 25OHD level of 40 nmol/L; (ii) that a 25OHD level below 30 nmol/L indicated risk of deficiency; (iii) that a 25OHD level above 50 nmol/L indicated sufficiency in 97.5%; and (iv) that 25OHD levels in excess of 125 nmol/L could be associated with harm. Ireland’s high northerly latitude, which prevents skin production for 6 months of the year, places our population at risk of vitamin D deficiency. We sought to determine the prevalence of both low and high 25OHD levels in samples routinely tested in our hospital laboratory in 2013.

Methods: This was a retrospective study of 25OHD measurements. A search of the laboratory information system was performed to determine the number and concentration of clinically requested 25OHD samples. Serum 25OHD concentrations were measured in 10,181 samples using the Elecsys vitamin D total automated competitive binding protein assay (Roche^®^ Diagnostics). In order to ensure a high standard of analysis, we participate in the UK DEQAS Vitamin D External Quality Assessment Scheme. Results are presented as median and interquartile range (IQR).

Results: The median 25OHD concentration for the population sample was 54.4(31.1–81.7) nmol/L, with values ranging from 7.5–1.389 nmol/L. According to IOM classification, 71.4% (*n* = 7.271) of 25OHD results fell within the range of adequacy and sufficiency. 23.8% (*n* = 2.422) were at increased risk of deficiency, 4.8% (*n* = 486) were at risk of harm. Of particular concern, we received samples from three individuals: one from a young sportsman whose 25OHD level was 750 nmol/L; a second from an individual undergoing treatment for prostate cancer with a 25OHD of 1.389 nmol/L; and a third from a multiple sclerosis sufferer with a 25OHD of 915 nmol/L. External hospital referral and outpatient sources accounted for the highest prevalence of hypovitaminosis D. General practitioners, external bone metabolic clinics and paediatric sources accounted for the highest prevalence of hypervitaminosis cases.

Conclusions: Groups at risk of hypovitaminosis D need to consume vitamin D-rich or fortified foods and would benefit from taking a daily low dose vitamin D supplement of 5–10 µg (200–400 IU) in keeping with the IOM specifications for total daily vitamin D intake. Higher doses of vitamin D as a means to prevent or correct privational vitamin D deficiency or to attain supraphysiological levels should be avoided. The estimated average vitamin D requirement from all dietary sources is strongly evidence-based and should represent the target total daily amount for everyone including supplement users. Therefore we advocate choosing low dose supplements. At risk groups should be assessed by measurement of 25OHD prior to recommending higher doses.

### 2.96. Association of Low Vitamin D Levels with Increased Risk of Stroke in Older Adults

#### Busch, M.A.; Scheidt-Nave, C.; Diehm, C.; Burghaus, I.; Trampisch, H.J.; Meves, S.H.; Mensink, G.B.M.; Berger, K.; Thiem, U.

Background: Accumulating evidence suggests that vitamin D deficiency may be a risk factor for cardiovascular disease but its association with the risk of stroke is uncertain.

Methods: In a prospective cohort study, serum concentration of 25-hydroxy vitamin D (25(OH)D) were measured in 6803 ambulatory primary-care patients aged ≥65 years (mean age 73 years; 58% women) who were included in the German Epidemiological Trial on Ankle-Brachial Index (getABI) in October 2001 and followed for up to 7 years for the occurrence of stroke.

Results: During a mean follow-up of 5.7 years, 249 participants had a stroke (84% ischemic), a stroke rate of 6.5 per 1000 person-years. The risk of stroke increased with decreasing baseline 25(OH)D levels (*p* for trend across quartiles <0.001). Compared to participants in the highest 25(OH)D quartile (>53.7 nmol/L), participants in the lowest quartile (<24.5 nmol/L) had twice the risk of any stroke (hazard ratio 2.0, 95% CI 1.4–2.9) and a 70% higher risk of ischemic stroke (1.7, 1.1–2.6) in Cox proportional hazards models adjusting for age, sex, education, smoking status, body mass index, renal function, and prior stroke. Additional adjustment for conventional risk factors and cardiovascular disease at baseline slightly attenuated these associations (any stroke: 1.8, 1.2–2.6; ischemic stroke: 1.5, 1.0–2.3). Results were similar when 310 participants with prior stroke were excluded.

Conclusions: Low vitamin D levels were associated with an increased risk of stroke in this prospective cohort study of older primary care patients.This association was independent of several important confounders and only partly explained by conventional risk factors and cardiovascular disease as potential causal intermediates.

### 2.97. Current Vitamin D Status of Adults in Germany and Its Association with Season, Latitude, and Other Determinants

#### Rabenberg, M.; Busch, M.A.; Scheidt-Nave, C.; Mensink, G.B.M.

Background: Previous studies based on national health survey data from 1998 have shown that serum 25-hydroxy vitamin D (25(OH)D) concentrations among adults in Germany were low (Hintzpeter *et al.* 2008). A main reason is the inadequate UVB radiation during fall and winter in northern latitudes, which limits the endogenous vitamin D production. The “German Health Interview and Examination Survey for Adults” (DEGS1) (Hintzpeter, B., et al*. Eur. J. Clin. Nutr.* 2008, *62*, 1079-1089) provides representative data to assess the current vitamin D status of adults in Germany according to latitude of residence and to identify additional determinants of vitamin D status.

Methods: DEGS1 is a comprehensive, nationwide health survey representative for the age group 18–79 years. It was conducted by the Robert Koch Institute from November 2008 to December 2011. Overall, 8151 adults participated in DEGS1. The survey instruments comprised questionnaires (including a semi-quantitative food frequency questionnaire; FFQ), interviews, physical examinations, and measurements in blood samples. Information on use of vitamin D supplements and oral contraceptives was obtained in computer-assisted personal interviews. Data on physical activity, media consumption, socio-economic status, and marital status was collected by self-administered questionnaires. A vitamin D intake index was constructed from FFQ information. Appointment dates of the participants where used to categorize the season of examination; latitudes (47°–49°, 50°–51° and 52°–54°) were derived according to region of residence. Participants with valid serum 25(OH)D measurements were included in the analyses (3694 women and 3422 men). Mean serum 25(OH)D values were calculated by gender and according to season and latitude. Determinants of vitamin D status were analyzed in gender-specific multiple linear regression models. Results were weighted to improve the representativeness.

Results: The majority of German adults had serum 25(OH)D concentrations below 50 nmol/L (61.7%). A total of 21.7% had levels <25 nmol/L, while 11.8% had levels ≥75 nmol/L. Nearly all year round, people living in southern Germany had higher mean serum 25(OH)D levels than people living in other parts of the country. At latitudes 47°–49°, the mean 25(OH)D levels exceeded the threshold of 50 nmol/L from May to October (except August), at latitudes 50°–51° and 52°–54° from June to September. In multivariate analyses, sunnier season, lower latitude, higher vitamin D intake index, vitamin D supplement use, higher physical activity, lower BMI, and marital status were significantly associated with higher serum 25(OH)D levels in both sexes. Additional factors independently associated with higher 25(OH)D levels included younger age, use of oral contraceptives, and higher socio-economic status among women, and lower media consumption among men.

Conclusion: The results confirm that the majority of German residents 18–79 years have serum 25(OH)D values below a recommended threshold of 50 nmol/L, especially during less sunny months and at higher latitudes. The identified determinants of serum 25(OH)D may contribute to tailor evidence-based recommendations for optimizing vitamin D status to specific target groups.

### 2.98. Vitamin D Deficiency Induced Muscle Wasting Occurs through the Ubiquitin Proteasome Pathway

#### Bhat, M.; Syed Qadri, S.Y.H.; Ismail, A.

Background: Vitamin D deficiency is known to lead to muscle weakness and bone fractures. Studies done in animals and humans have reported muscle atrophy/wasting in vitamin D deficiency. Muscle atrophy results due to an imbalance between protein degradation and synthesis pathways. Intracellular protein degradation occurs by three different proteolytic pathways namely, the ubiquitin proteasome pathway (UPP), the lysosomal pathway and the calpain pathway. The role of these pathways in vitamin D deficiency induced muscle wasting has not been studied. In the present work we assessed the involvement of these three proteolytic systems in vitamin D deficiency induced muscle wasting.

Methods: A vitamin D deficient rat model was employed for the studies.Vitamin D deficient rats were fed a diet devoid of vitamin D, while control rats were given the same diet containing 1000 IU vitamin D_3_/Kg diet. Vitamin D deficiency was confirmed by measuring the serum calcium and 25(OH)D_3_, an indicator for vitamin D status.Vitamin D deficient rats had undetectable serum 25(OH)D_3_ and hypocalcaemia. Vitamin D deficient rats were further subdivided into three groups: one group continued on deficient diet, second group was rescued with control diet to see for reversal of effects observed in D-deficiency, and the third group of rats was supplemented with high calcium diet alone to delineate the role of calcium per se on the muscle atrophy seen in vitamin D deficiency.

Results: Total protein degradation as measured by the release of tyrosine from muscle was significantly increased in the deficient group than the controls.Urinary excretion of the amino acid 3-methylhistidine, an *in vivo* index for muscle protein degradation was also increased in the vitamin D deficient group compared to vitamin D sufficient group. Type II fibre area was decreased in vitamin D deficient muscle and this was reversed upon rehabilitation with vitamin D. The 20S proteasomal enzyme activities namely chymotrypsin-like (Ch-L), trypsin-like (T-L) and caspase-like (Cp-L) were significantly increased in the deficient muscle than the control muscle. All the three enzyme activities were inhibited (>95%) by clasto-lactacystin, a specific inhibitor of 26S proteasome. Also, the protein levels of the E2- ubiquitin conjugating enzyme and high molecular weight ubiquitin conjugates were higher in deficient group than the control group. Furthermore, the expression of muscle atrophy marker genes; Atrogin-1 and MuRF1, and proteasomal subunit genes PSC2 and PSC8 were significantly increased in deficient muscle compared to the control muscle. Both rescue with vitamin D and supplementation with high calcium diets corrected all the muscle changes observed. On the other hand, neither the activities nor gene expression levels of lysosomal and calpain enzymes were altered between the groups.

Conclusions: In summary, this study demonstrates that during vitamin D deficiency induced muscle wasting, the activity of the ubiquitin proteasome pathway is elevated.

### 2.99. Effect of Vitamin D on Blood Pressure—A Systematic Review and Meta-Analysis of Randomised Controlled Trials

#### Beveridge, L.A.; Struthers, A.D.; Khan, F.; Jorde, R.; Scragg, R.; Macdonald, H.; Witham, M.D.; on behalf of the D-PRESSURE Collaboration

Background: Low 25-hydroxy vitamin D levels are associated with higher prevalent blood pressure and incident hypertension in observational studies. In contrast, a growing number of small randomised trials testing the effect of vitamin D supplementation on blood pressure have reported mixed results.

Methods: Systematic review and meta-analysis. We performed a systematic review and metaanalysis to examine whether vitamin D reduces blood pressure. Databases including MEDLINE, EMBASE, CINAHL and the Cochrane library were searched, supplemented by searches of grey literature, unpublished trials and references from included studies. Studies were assessed by two reviewers independently according to a prespecified protocol. Interventions included activated vitamin D, unactivated vitamin D_2_ and D_3_ and vitamin D analogues including paricalcitol. Studies were included only if intervention was compared to placebo, although identical cointervention in each arm was permitted.

Results: 28 eligible trials were identified, of which 23 trials with mean blood pressure data could be included in meta-analysis (2313 participants). Study size ranged from 9 to 330 participants. Study quality was variable; most studies reported adequate allocation concealment and balanced study groups, but not all studies clearly adhered to intention to treat analysis. The overall treatment effect for systolic blood pressure was 0.2 mmHg (95% CI −1.3 to 1.6; *I*^2^ = 37%) and for diastolic blood pressure was 0.1 mmHg (95% CI −0.6 to 0.8; *I*^2^ = 18%). No relationship was evident between size of blood pressure reduction and either baseline systolic blood pressure or 25-hydroxy vitamin D level on meta-regression. No significant differences were seen between supplementation with ergocalciferol, cholecalciferol, 1-alpha hydroxylated vitamin D derivatives, or paricalcitol.

Conclusion: This meta-analysis suggests vitamin D supplementation does not significantly reduce blood pressure at the doses tested to date. Further work including individual patient data analysis is underway.

### 2.100. Vitamin D Therapy to Reduce Blood Pressure and Left Ventricular Hypertrophy in Resistant Hypertension A Randomised Controlled Trial

#### Witham, M.D.; Ireland, S.E.; Houston, J.G.; Gandy, S.J.; Waugh, S.; MacDonald, T.M.; Mackenzie, I.S.; Struthers, A.D.

Background: Low 25-hydroxy vitamin D levels are associated with higher incident hypertension rates and higher prevalent blood pressure. We tested whether high-dose intermittent oral vitamin D therapy could reduce blood pressure and left ventricular mass in patients with hypertension resistant to conventional treatment.

Methods: We conducted a parallel-group, double-blind, randomised placebo-controlled trial. Patients with supine office blood pressure >140/90 mmHg on 3 or more antihypertensive agents and baseline 25-hydroxy vitamin D levels <75 nmol/L received 100,000 units oral vitamin D_3_ or matching placebo every 2 months. Office and 24 h ambulatory blood pressure, glucose and cholesterol were measured at baseline, 2, 4 and 6 months; left ventricular mass index was measured by cardiac magnetic resonance imaging on a subgroup at baseline and 6 months. The primary outcome was between-group difference in mean 24 h ambulatory blood pressure at 6 months.

Results: 68 participants were randomised, 34 to each group. Mean age was 63 (SD 11) years, mean baseline office blood pressure was 154/84(13/10) mmHg and mean baseline 25-hydroxy vitamin D level was 42(16) nmol/L. Treatment with vitamin D did not reduce 24 h ambulatory blood pressure (adjusted treatment effects: systolic +3 mmHg, 95% CI −4 to +11, *p* = 0.33; diastolic −2 mmHg, 95% CI −6 to +2, *p* = 0.29); similar results were seen for office blood pressure. Left ventricular mass index was measured in a subgroup (*n* = 25); no reduction was seen with vitamin D treatment (adjusted treatment effect +4 g/m^2^, 95% CI 0 to +7, *p* = 0.04). There was no significant change in cholesterol or glucose levels.

Conclusion: Six months of intermittent, high-dose oral vitamin D_3_ did not reduce blood pressure or left ventricular mass in patients with resistant hypertension.

### 2.101. Effect of High-Dose Intermittent Vitamin D_3_ Supplementation on Symptoms and Markers of Vascular Function in Patients with Chronic Fatigue Syndrome

#### Witham, M.D.; Adams, F.; McSwiggan, S.; Kennedy, G.; Kabir, G.; Belch, J.J.F.; Khan, F.

Background: Low 25-hydroxy vitamin D levels are common in patients with chronic fatigue syndrome; such patients also manifest impaired vascular health. We tested whether high-dose intermittent oral vitamin D therapy could improve markers of vascular health and fatigue in patients with chronic fatigue syndrome.

Methods: We conducted a parallel-group, double-blind, randomised placebo-controlled trial. Patients with chronic fatigue syndrome according to the Fukuda (1994) and Canadian (2003) criteria and baseline 25-hydroxy vitamin D levels <75 nmol/L were randomised to receive 100,000 units oral vitamin D_3_ or matching placebo every 2 months. Outcomes were measured at baseline, 2 and 6 months. Endothelial function was measured using flow-mediated dilatation of the brachial artery, along with pulse wave velocity, blood pressure, cholesterol, insulin resistance measured using HOMA-IR, interleukin 6, tumour necrosis factor alpha, markers of oxidative stress, and the Piper Fatigue scale. The primary outcome was between-group difference in arterial stiffness measured by carotid-femoral pulse wave velocity at 6 months, adjusted for baseline values.

Results: 50 participants were randomised, 25 to each group. Mean age was 49 (SD 13) years, mean baseline pulse wave velocity was 7.8 m/s (SD 2.3), mean baseline office blood pressure was 128/78 (18/12) mmHg and mean baseline 25-hydroxy vitamin D level was 46(18) nmol/L. Treatment with vitamin D did not improve pulse wave velocity at 6 months (adjusted treatment effect 0.0 m/s; 95% CI −0.6 to 0.6; *p* = 0.93). No improvement was seen in blood pressure, endothelial function, cholesterol, insulin resistance, oxidative stress or inflammatory markers. There was no improvement in the Piper Fatigue scale at 6 months (adjusted treatment effect 0.2 points; 95% CI −0.8 to 1.2; *p* = 0.73).

Conclusion: Six months of intermittent, high-dose oral vitamin D_3_ did not improve markers of vascular health or fatigue in patients with chronic fatigue syndrome.

### 2.102. Increased Food Allergy with Vitamin D: A Randomized, Double-Blind, Placebo-Controlled Trial

#### Urashima, M.; Norizoe, C.; Akiyama, N.; Segawa, T.; Tachimoto, H.; Mezawa, H.; Ida, H.

Background: To elucidate whether maternal vitamin D supplementation during lactation improves infantile eczema and other subsequent allergic disorders, a randomized, double-blind, placebo-controlled trial was performed.

Methods: Mothers (*n* = 164) of infants with facial eczema at one-month checkup were randomly assigned to receive vitamin D_3_ supplements (*n* = 82; 800 IU/day) or placebo (*n* = 82) for 6 weeks from May 2009 to January 2011. The primary outcome was infantile eczema quantified by Scoring Atopic Dermatitis (SCORAD) index at the three-month checkup, and the secondary outcomes were atopic dermatitis, food allergy, and wheeze diagnosed by doctors up to 2 years of age.

Results: There was no significant difference in SCORAD at 3-month checkup between two comparative groups. Doctor-diagnosed food allergy was significantly more common up to age 2 years in vitamin D group (10/39: 25.7%) than in placebo group (3/40: 7.5%; RR = 3.42, 95% CI = 1.02 to 11.77, *p* = 0.030). Moreover, at least one secondary outcome was also significantly more common in vitamin D group (17/39: 43.6%) than in placebo group (7/40: 17.5%; RR = 2.49, 95% CI = 1.16 to 5.34, *p* = 0.012).

Conclusions: These results suggest that vitamin D supplementation may not decrease the severity of infantile eczema at three months of age, but may rather increase the risk of later food allergy up to two years of age. Because a large number of subjects was lost to follow-up, further study is needed to confirm the findings.

### 2.103. Randomized, Double-Blind, Placebo-Controlled Trial of Vitamin D Supplement in Parkinson’s Disease

#### Urashima, M.; Suzuki, M.; Yoshioka, M.; Hashimoto, M.; Murakami, M.; Noya, M.; Takahashi, D.

Background: In our previous study, higher serum 25-hydroxy vitamin D (25OHD) levels and the vitamin D receptor (VDR) FokICC genotype were associated with milder Parkinson’s disease (PD). Whether vitamin D_3_ supplementation inhibits progression was evaluated based on patient VDR subgroup.

Methods: Patients with PD (*n* = 114) were randomly assigned to receive vitamin D_3_ supplements (*n* = 56; 1200 IU/day) or placebo (*n* = 58) for 12 months in double-blind setting. Outcomes were clinical changes from baseline and the percentage of patients not worsening in modified Hoehn & Yahr (HY) stage and Unified PD Rating Scale (UPDRS).

Results: Compared with placebo, vitamin D_3_ significantly prevented deterioration of the HY stage in patients (difference between groups: *p* = 0.005; within vitamin D_3_, mean (SD): +0.02 (0.62), *p* = 0.79; within placebo group, mean: +0.33 (0.70), *p* = 0.0006). Interaction analyses showed that VDR FokI genotypes modified the effect of vitamin D_3_ on changes in HY stage (Pinteraction = 0.045), UPDRS total (Pinteraction = 0.039), and UPDRS Part II (Pinteraction = 0.021). Compared with placebo, vitamin D_3_ significantly prevented deterioration of the HY stage in patients with FokITT (difference between groups: *p* = 0.009; within vitamin D_3_, mean (SD): −0.38 (0.48), *p* = 0.91; within placebo group, mean: +0.63 (0.77), *p* = 0.009) and FokICT (difference between groups: *p* = 0.020; within vitamin D_3_, mean (SD): ± 0.00 (0.60), *p* = 0.78; within placebo group, mean: +0.37 (0.74), *p* = 0.014) but not FokICC. Similar trends were observed in UPDRS total and Part II.

Conclusions: Vitamin D_3_ supplementation may stabilize PD for a short period in patients with FokITT or CT genotypes without triggering hypercalcemia, although this effect may be non-specific for PD.

### 2.104. Effect of Vitamin D Supplement on Influenza A Illness during the 2009 H1N1 Pandemic: A Randomized Controlled Trial

#### Urashima, M.; Mezawa, H.; Noya, M.; Camargo, C.A., Jr.

Background: In a prior randomized trial, we found that the incidence of influenza A was less in the vitamin D_3_ group than among those on placebo, but total incidence of either influenza A or B did not differ between groups. In a post hoc analysis of this trial, we found that the incidence of influenza A or B was less in the vitamin D_3_ group than in the placebo group only during the first half of the study. To elucidate whether vitamin D_3_ had preventive actions against influenza A, we conducted another trial during the 2009 pandemic of the H1N1 subtype of influenza A.

Methods: Students (*n* = 247) of a Japanese high school were randomly assigned to receive vitamin D_3_ supplements (*n* = 148; 2000 IU/day) or placebo (*n* = 99) in double-blind setting for 2 months from October 18 to December 17, 2009. The primary outcome was incidence of influenza A diagnosed with rapid influenza diagnostic test (RIDT).

Results: In the first month, influenza A occurred significantly less in the vitamin D_3_ group (2/148: 1.4%) compared with the placebo group (8/99: 8.1%) (risk ratio, 0.17; 95% confidence interval, 0.04 to 0.77; *p* = 0.009). However, during the second month, the vitamin D_3_ group experienced more events and effectively caught up with the placebo group. Ultimately, influenza A was equally likely in the vitamin D_3_ group (20/148: 13.5%) compared with the placebo group (12/99: 12.1%).

Conclusions: Vitamin D_3_ supplementation did not lower the overall incidence of influenza A during the 2009 H1N1 pandemic. A post hoc analysis suggests that the initial benefit during the first month of treatment was lost during the second month. The impact of vitamin D_3_ supplement on non-pandemic respiratory viruses requires further study.

### 2.105. Association of Vitamin D Receptor Gene Polymorphism (Fok1:rs2228570) with Risk of Coronary Artery Disease

#### Abu el Maaty, M.A.; Hassanein, S.I.; Gad, M.Z.

Background: Despite the identification of several genetic polymorphisms in the vitamin D receptor gene (VDRG), the rs2228570 polymorphism, identified by the Fok1 restriction enzyme, appears to be the only one influencing the size of the translated protein. The Fok1 polymorphism has been previously associated with numerous diseases such as cancer however; its association with coronary artery disease (CAD) has never been previously investigated.

Methods: Male patients (*n* = 98), 35–50 years of age, with verified CAD were recruited alongside age- and sex-matched controls (*n* = 55). Genotyping was performed by PCR-RFLP and plasma 25-Hydroxy vitamin D levels were assessed by HPLC-UV.

Results: The distribution of the subjects among the genotypes TT (wild type), CT and CC were 15.3%, 31.6% and 53.1% respectively for patients whereas 32.8%, 23.6% and 43.6% respectively for controls. The C-variant (mutant) was predominantly expressed in patients compared to controls (68.9% *vs.* 55.5%; *p* = 0.025). None of the observed genotypes were significantly associated with circulating 25-Hydroxy vitamin D levels in patients.

Conclusion: Results present the Fok1 polymorphism of the VDRG as a novel genetic marker for CAD. A lack of association between the genotypes and 25-Hydroxy vitamin D in patients suggests an absence of influence of this polymorphism on circulating levels of vitamin D.

### 2.106. Vitamin D Receptor Gene Polymorphism (TaqI and ApaI) in Egyptian Patients with Coronary Artery Disease

#### Abu el Maaty, M.A.; Hassanein, S.I.; Gad, M.Z.

Background: The latter part of the twentieth century has witnessed the identification of nuclear vitamin D receptors in almost every cell of the human body, thereby explaining both skeletal and proposed extra-skeletal activities. It is thus conceivable that polymorphisms in the gene encoding this protein, the vitamin D receptor gene (VDR), may alter both its structure and functions and hence, may influence one’s susceptibility to various debilitating diseases. Previous studies have illustrated the association of the ApaI and TaqI polymorphisms of the VDR, located in non-coding and coding regions respectively, with diseases such as cancer and cardiovascular disease, however investigating such association in Egyptian patients with coronary artery disease (CAD) has never been formerly attempted.

Methods: Male patients (*n* = 137), 35 to 50 years of age, with verified CAD, were recruited alongside age- and sex-matched controls (*n* = 58). Genotyping was performed by PCR-RFLP.

Results: The genotypic distribution of the ApaI polymorphism in patients was 56.25% (AA), 21.17% (Aa) 22.63% (aa) whereas 62% (AA), 20.69% (Aa) and 17.31% (aa) in controls, the comparison of which yielded insignificant results (*p* = 0.67). The genotypic distribution of the TaqI polymorphism in patients was 26.28% (TT), 44.53% (Tt), and 29.19% (tt) whereas 29.41% (TT), 31.37% (Tt), 39.22% (tt) in controls, which resulted in insignificant results upon comparison (*p* = 0.26). Comparison of the allelic distribution of the ApaI polymorphism in patients (66.7% “A”-allele) and controls (33.3% “A”-allele) yielded an insignificant result (*p* = 0.29). Additionally, insignificant results (*p* = 0.64) were reached upon the comparison of the TaqI polymorphism allelic distribution between patients (51.46% “t”-allele) and controls (48.54% “t”-allele).

Conclusions: This study presents the ApaI and TaqI polymorphisms of VDR as non-influencing players in the pathogenesis of CAD in Egyptian males. Investigating such association in various populations will conclude the validity of these polymorphisms as genetic markers for CAD.

### 2.107. Insights on Vitamin D’s Role in Cardiovascular Disease: Investigating the Association of 25-Hydroxy Vitamin D with the Dimethylated Arginines

#### Abu el Maaty, M.A.; Hassanein, S.I.; Hanafi, R.S.; Gad, M.Z.

Background: Accumulating evidence has stipulated a strong correlation between vitamin D (vitD) deficiency and cardiovascular disease (CVD) however a mechanistic link is missing. This study investigated the association of vitD with endothelial dysfunction parameters.

Methods: Subjects comprised male patients with verified coronary artery disease (CAD) (*n* = 69) and age- and sex-matched controls (*n* = 20). 25-Hydroxy vitamin D [25(OH)D] was determined using high performance liquid chromatography with ultraviolet detection whereas asymmetric and symmetric dimethylarginine (ADMA and SDMA, respectively) were determined by liquid chromatography-mass spectrometry. Nitric oxide (NO) was determined spectrophotometrically and high-sensitivity C-reactive protein (hs-CRP) was determined using enzyme-linked immunosorbent assay (ELISA).

Results: Comparison of mean 25(OH)D concentrations of patients and controls yielded a significant result (*p* = 0.0002). 25(OH)D_2_ was dominant in patients whereas 25(OH)D_3_ was dominant in controls (*p* = 0.003 and 0.001, respectively). Comparison of mean ADMA and SDMA concentrations of patients exhibiting normal and suboptimal vitD yielded insignificant results (*p* = 0.692 and 0.998, respectively). Significant results were obtained from the comparison of mean hs-CRP and NO concentrations of patients exhibiting normal and suboptimal vitD (*p* = 0.035 and 0.031, respectively).

Conclusions: Results suggest involvement of vitD with the NO system, however not via modulation of the dimethylated arginines. A potential anti-inflammatory activity for vitD is also raised.

### 2.108. Age and Milk Consumption are Associated with Vitamin D Status in Pre-Menopausal Saudi Women

#### Aljohani, N.J.; Abu Zaid, L.; Abbas, M.A.; Alkaabba, A.F.; Alghamdi, M.; Alothman, H.O.; Mohammed, G.; Alshahrani, F.

Background: There is little evidence published on prevalence of vitamin D deficiency among Saudi women, in spite of the widespread food fortification and the excellent opportunity of available sun light all over the year. The present cross-sectional study aims to determine the prevalence and risk factors of vitamin D deficiency among premenopausal women visiting commercial centers in Riyadh City.

Methods: A quasi-random technique was employed in the recruitment of subjects from various commercial Malls in Riyadh last May-November, 2012. A total of 256 subjects filled a general questionnaire, height and weight were measured and blood extracted ascertaining total 25-hydroxy vitamin D, calcium, phosphorous and alkaline phophatase from a vitamin D External Quality Assessment (DEQAS)-certified laboratory.

Results: Vitamin D deficiency (<50 nmol/L) was noted in 200 (77.6%) of subjects. Age and milk consumption were the significant predictors of vitamin D status, with 33.9% of variance perceived (*p* < 0.001). Increased BMI, being married and the presence of muscle pain were all significantly associated with vitamin D deficiency.

Conclusion: Nearly 4 out of 5 premenopausal Saudi women shoppers harbor vitamin D deficiency and this is influenced not by sun exposure, but by age and milk consumption. It is clear that general female public faces an imminent threat of vitamin D deficiency-related diseases unless aggressive public awareness is conducted.

### 2.109. Differences and Associations of Metabolic and Vitamin D Status among Patients with and without Sub-Clinical Hypothyroid Dysfunction

#### Aljohani, N.; Al-Daghri, N.M.; Al-Attas, O.S.; Alokail, MS.; Alkharfy, K.M.; Al-Othman,; Yakout, S.; Alkabba, A.F.; Al-Ghamdi, A.S.; Almalki, M.; Buhary, B.M.; Sabico, S.

Background: Sub-clinical hypothyroid dysfunction, a relatively understudied disorder in the Kingdom of Saudi Arabia (KSA), has significant clinical implications if not properly monitored. Also from KSA, more than 50% of the population suffer from hypovitaminosis D (<50 nmol/L). In this cross-sectional case-control study, we described the differences and associations in the metabolic patterns of adult Saudis with and without hypothyroid dysfunction in relation to their vitamin D status, PTH, calcium and lipid profile.

Methods: A total of 94 consenting adult Saudis (52 controls (without subclinical hypothyroidism), 42 cases (previously diagnosed subjects)) were included in this cross-sectional study. Anthropometrics were obtained and fasting blood samples were taken for ascertaining lipid and thyroid profile, as well as measuring PTH, 25(OH) vitamin D and calcium.

Results: Cases had a significantly higher body mass index than the controls (*p* < 0.001). Circulating triglycerides was also significantly higher in cases than the controls (*p* = 0.001). A significant positive association between HDL-cholesterol and PTH (*R* = 0.56; *p* = 0.001), as well as a negative and modestly significant negative association between LDL-cholesterol and PTH (*R* = −20.0; *p* = 0.04) were observed. FT3 was inversely associated with circulating 25(OH) vitamin D (*R* = −0.25; *p* = 0.01).

Conclusion: Patients with hypothyroid dysfunction possess several cardiometabolic risk factors that include obesity and dyslipidemia. The association between PTH and cholesterol levels as well as the inverse association between vitamin D status and FT3 needs to be reassessed prospectively on a larger scale to confirm these findings.

### 2.110. Vitamin D Supplementation in Patients with Diabetes Mellitus Type 2 on Different Therapeutic Regimens: A One-Year Prospective Study

#### Alkharfy, K.M.; Al-Daghri, N.M.; Sabico, S.B.; Al-Othman, A.; Moharram, O.; Alokail, M.S.; Al-Saleh, Y.; Kumar, S.; Chrousos, G.P.

Background: Little or no research has determined the effect of vitamin D_3_ supplementation in conjunction with pharmacological and non-pharmacological approaches in the diabetes mellitus type 2 (DMT2) patients. The objective of this study was to determine the effect of vitamin D_3_ supplementation in a cohort of Saudi DMT2 population on diet, insulin and/or different oral hypoglycemic agents and compare them with a non-DMT2 control cohort.

Methods: A total of 499 randomly selected Saudi subjects divided into 8 groups (Non-DMT2 Control = 151; Rosiglitazone alone = 49; Diet = 15; Insulin alone = 55; Insulin + Orals = 12; Metformin alone = 121; Oral agents combination = 37; Sulphonylurea alone = 59) were included in this 12-month interventional study. All DMT2 patients were given 2000 IU vitamin D_3_ daily, while the control group received none but were advised to increase sun exposure. Anthropometrics, glucose, lipid profile and 25-hydroxy vitamin D (25-OHVitD) were measured at baseline, 6 and 12 months.

Results: Circulating 25-OHVitD concentrations improved in all patient groups. The metformin group showed the highest change in circulating vitamin D levels both at 6 months (62.6%) and 12 months (50.6%) as compared to baseline (*p* < 0.001). No significant changes were observed in the BMI and glucose in any of the DMT2 groups. In contrast, the insulin + oral agents group showed more significant improvements in the metabolic profile, which included triglycerides and total cholesterol, as well as systolic blood pressure and HDL-cholesterol in males. Also, significant decreases in triglycerides were observed in the rosiglitazone and insulin + oral hypoglycemic agent groups both at 6 and 12 months of supplementation (both *p*-values < 0.001).

Conclusion: While in all DMT2 groups circulating levels of 25-OHVitD increased after supplementation, in DMT2 patients on insulin in combination with other drugs benefitted the most in improving cardiovascular risk. Metformin improves 25-hydroxy vitamin D levels but did not seem to confer other added cardiometabolic benefits.

### 2.111. Vitamin D Supplementation as an Adjuvant Therapy for Patients with T2DM: An 18-Month Prospective Interventional Study

#### Al-Daghri, N.M.; Alkharfy, K.M.; Al-Othman, A.; El-Kholie, E.; Moharram, O.; Alokail, M.S.; Al-Saleh, Y.; Sabico, S.; Kumar, S.; Chrousos, G.P.

Background: Vitamin D deficiency has been associated with impaired human insulin action, suggesting a role in the pathogenesis of diabetes mellitus type 2 (T2DM). In this prospective interventional study we investigated the effects of vitamin D_3_ supplementation on the metabolic profiles of Saudi T2DM subjects pre- and post-vitamin D supplementation over an 18-month period.

Methods: T2DM Saudi subjects (men, *n* = 34: Age: 56.6 ± 8.7 years, BMI, 29.1 ± 3.3 kg/m^2^; women, *N* = 58: Age: 51.2 ± 10.6 years, BMI 34.3 ± 4.9 kg/m^2^;) were recruited and given 2000 IU vitamin D_3_ daily for 18 months. Anthropometrics and fasting blood were collected (0, 6, 12, 18 months) to monitor serum 25-hydroxy vitamin D using specific ELISA, and to determine metabolic profiles by standard methods.

Results: In all subjects there was a significant increase in mean 25-hydroxy vitamin D levels from baseline (32.2 ± 1.5 nmol/L) to 18 months (54.7 ± 1.5 nmol/L; *p* < 0.001), as well as serum calcium (baseline = 2.3 ± 0.23 nmol/L *vs.* 18 months = 2.6 ± 0.1 mmol/L; *p* = 0.003). A significant decrease in LDL-[(baseline = 5.4 ± 0.2 mmol/L *vs.* 18 months = 3.6 ± 0.8mmol/L, *p* < 0.001] and total cholesterol (baseline = 5.4 ± 0.2mmol/L *vs.* 18 months = 4.9 ± 0.3mmol/L, *p* < 0.001) were noted, as well as a significant improvement in HOMA-β function (*p* = 0.002). Majority of the improvements elicited were more prominent in women than men.

Conclusion: In the Saudi T2DM population receiving oral vitamin D_3_ supplementation (2000 IU/day). Circulating 25-hydroxy vitamin D levels remained below normal 18 months after the onset of treatment. Yet, this “sub-optimal” supplementation significantly improved lipid profile with a favorable change in HDL/LDL ratio, and HOMA-β function, which were more pronounced in T2DM females.

### 2.112. Effect of Non-Pharmacologic Vitamin D Status Correction on Circulating Bone Markers in Healthy Overweight and Obese Saudis

#### Al-Daghri, N.M.; Alkharfy, K.M.; Al-Othman, A.; Yakout, S.; Al-Saleh, Y.; Fouda, M.; Sabico, S.

Background: While moderate to severe vitamin D deficiency is prevalent in Saudi Arabia, skeletal effects associated with this deficiency are not common in this population. In this interventional study we measured the effects of improving vitamin D status on bone biochemical markers in overweight and obese adult Saudis. Methods: A total of 47 volunteers (21 males, 26 females) out of the initial 95 subjects were given verbal advice to expose themselves to sunlight for 5–30 min twice weekly and were encouraged to increase their intake of vitamin D–rich foods. Serum 25(OH)D, osteocalcin, and type 1 collagen cross-linked C-telopeptide (CTx), were measured at baseline and after one year.

Results: A significant decrease in the prevalence of vitamin D deficiency was observed (44% to 27%) after one year follow-up (*p* = 0.025). Also, a parallel significant increase in osteocalcin and a decrease in CTX and osteoprotegerin were observed.

Conclusion: The results suggest that a modest increase in vitamin D levels among overweight and obese subjects through the promotion of lifestyle changes for one year have marginal effects in bone turnover markers as well as obesity itself.

### 2.113. Increased Vitamin D Supplementation Recommended during Summer Season in the Gulf Region: A Counterintuitive Seasonal Effect in Vitamin D Levels in Adult, Overweight and Obese Middle-Eastern Residents

#### Al-Daghri, N.M.; Al-Attas, O.S.; Alokail, M.S.; Alkharfy, K.M.; El-Kholie, E.; Yousef, M.; Al-Othman, A.; Al-Saleh, Y.; Sabico, S.; Kumar, S.; Chrousos, G.P.

Background: Seasonal variations in circulating vitamin D levels provide vital information as to the most appropriate time to either start or increase vitamin D supplementation in order to maintain optimal vitamin D levels. In this follow-up study we determined seasonal differences in serum 25(OH)D vitamin D levels, as well as parallel changes in metabolic parameters, in a cohort of adult overweight and obese Saudis.

Methods: 121 adult, overweight and obese, consenting Saudis aged 18–70 years old were randomly recruited from 4 (Primary Health Care Centers) PHCCs in Riyadh. They were divided according to the season when baseline measurements were made [74 Summer (April–October); 47 Winter (November–March)]. Anthropometrics were performed and fasting blood samples were taken at baseline and every 3 months for 1 year. Fasting blood glucose, corrected calcium and lipid profiles were measured routinely. Serum 25(OH)D was quantified using a specific enzyme-linked immunosorbent assay (ELISA).

Results: Age- and BMI-matched mean 25(OH) vitamin D levels from the winter group were significantly higher than those of the summer group (*p* < 0.001). In both groups, HDL-C levels improved significantly as 25(OH) vitamin D levels increased with subsequent follow-ups, even after adjusting for age, gender and BMI (*p* < 0.001).

Conclusion: Seasonal differences in serum 25(OH) vitamin D levels in Saudi Arabia are counterintuitive, with circulating levels being higher during the winter rather than the summer season. Increased vitamin D supplementation is thus recommended to maintain optimal serum 25(OH) vitamin D levels during the summer season.

### 2.114. Longitudinal and Seasonal Changes in Serum 25-Hydroxy Vitamin D (25-OHD) Levels in Different Age Groups, and Clinical Implications

#### Van Schoor, N.M.; Sohl, E.; Knol, D.L.; Lips, P.

Background: Longitudinal changes in serum 25-OHD levels during aging have not been studied extensively. When describing the longitudinal change, it is highly important to adequately adjust for seasonal variation in serum 25-OHD levels. The research aims of the current study are: (1) To examine longitudinal changes in serum 25-OHD levels in different age groups; (2) To describe the seasonal variation in different age groups using a cosine function; (3) To examine whether optimal serum 25(OH)D levels for physical functioning differ according to season.

Methods: Two answer the first two research aims, data of the Longitudinal Aging Study Amsterdam (LASA) were used, an ongoing cohort study. Two different cohorts were included: (1) younger cohort: aged 55–65 years at baseline, *n* = 738, follow-up of six years; (2) older cohort: aged 65–88 years at baseline, *n* = 1320, follow-up of thirteen years. Linear Mixed Models was used to examine the longitudinal changes within the two cohorts; seasonal variation was modeled by adding a cosine function with a period of one year to the model. To answer the third research aim, baseline data of the B-Vitamins for the PRevention Of Osteoporotic Fractures (B-PROOF) study were used, an RCT on B-vitamins and fracture risk in community-dwelling persons aged 65+. Physical functioning was assessed using three different tests: the walking test, the chair stands test and the tandem stand. Restricted cubic spline functions and plots were used to estimate the optimal cut-off of serum 25-OHD in the relationship with physical functioning stratified by winter (*n* = 1372) and summer season (*n* = 1441).

Results: At baseline, average serum 25-OHD levels were 56.5 nmol/L in the younger cohort and 51.1 nmol/L in the older cohort. In the younger cohort, a longitudinal increase in mean serum 25-OHD levels of 5 nmol/L in six years was observed; in the older cohort, a longitudinal decrease in mean serum 25-OHD levels of 5 nmol/L in thirteen years was observed. The seasonal variation was ±11 nmol/L in the younger cohort, and ±7 nmol/L in the older cohort. The optimal serum 25-OHD level in the relation with physical functioning was between 60 and 70 nmol/L for both seasons.

Conclusions: Serum 25-hydroxy vitamin D levels changed during follow-up with increasing levels in persons aged 55–65 years, and decreasing levels in persons aged 65–88 years. On average, the seasonal variation was larger than the longitudinal change. Our findings implicate that vitamin D supplementation becomes more important in older age groups and during wintertime. Another implication might be that serum 25-OHD measurements during summertime should be repeated during wintertime, or that a higher threshold for vitamin D deficiency (*i.e.*, 60–70 nmol/L) should be used in summer.

### 2.115. A Systematic Review and Meta-Analysis of the Effect of Vitamin D in Pregnancy on Offspring Health Outcomes

#### Harvey, N.C.; Holroyd, C.; Ntani, G.; Javaid, M.K.; Cooper, P.; Moon, R.J.; Cole, Z.A.; Tinati, T.; Bishop, N.J.; Godfrey, K.M.; Dennison, E.M.; Baird, J.; Cooper, C.; the UK Vitamin D in Pregnancy Working Group.

Background: We performed a systematic review to explore whether (1) low maternal circulating 25(OH)-vitamin D (25(OH)D) during pregnancy is associated with impairment of offspring health; and (2) maternal supplementation with vitamin D in pregnancy might ameliorate these effects.

Methods: Major electronic databases were searched up to June 2012 covering both published and grey literature. Bibliographies of selected papers were hand-searched for additional references. Relevant authors were contacted for any unpublished findings and additional data if necessary. All reviews, data extraction and quality assessments were performed by two reviewers according to CRD guidelines. Eligible studies included pregnant women and their offspring, and one or more relevant exposures (either assessment of vitamin D status (dietary intake, sunlight exposure, circulating 25(OH)D) or supplementation of participants with vitamin D or vitamin D containing food e.g., oily fish) and outcomes (offspring birth weight, birth length, head circumference, bone mass, anthropometry and body composition, risk of asthma and atopy, small for gestational dates, preterm birth, type 1 diabetes, low birth weight, serum calcium concentration, blood pressure and rickets). Maternal health outcomes were also addressed.

Results: 76 studies were included. There was considerable heterogeneity between the studies and for most outcomes there was conflicting evidence. Indeed, no convincing evidence was found for any association between maternal vitamin D status and offspring asthma, atopy, type 1 diabetes or blood pressure. However, modest positive relationships were identified between maternal 25(OH)D and (1) offspring cord blood or postnatal calcium concentrations (meta-analysis of 6 intervention studies, mean difference 0.05 mmol/L (95% CI 0.02, 0.05); studies all had high risk of bias); (2) offspring birth weight (meta-analysis of 3 observational studies using log-transformed 25(OH)D concentrations, pooled regression coefficient adjusting for potential confounding factors 5.63 g/10% change in maternal 25(OH)D (95% CI 1.11,10.16), but no association in 4 studies using natural units, or across intervention studies); and (3) offspring bone mass (in observational studies judged to be of good quality, but which did not permit meta-analysis).

Conclusions: There was modest evidence to support associations between maternal 25(OH)-vitamin D status and offspring serum calcium concentrations, birth weight and bone mass. However, these findings were limited by their observational nature or risk of bias. High-quality intervention studies to investigate these outcomes are now required, as the current evidence base cannot adequately inform clinical practice.

Acknowledgements: The UK Vitamin D in Pregnancy Working Group.

### 2.116. Childhood Bone Mineral Content is Associated with Methylation Status of the RXRA Promoter at Birth

#### Harvey, N.C.; Sheppard, A.; Godfrey, K.M.; McLean, C.; Garratt, E.; Ntani, G.; Davies, L.; Murray, R.; Inskip, H.M.; Gluckman, P.D.; Hanson, M.A.; Lillycrop, K.A.; Cooper, C.

Background: RXRa is an important part of the binding and nuclear action of several molecules, including vitamin D, thyroxin, glucocorticoids and PPAR. We have previously demonstrated that maternal lifestyle, body build, physical activity and vitamin D status during pregnancy are associated with offspring bone mass. In this study we aimed to use a population based mother-offspring cohort, the Southampton Women’s Survey, to explore the relationships between methylation status of the promoter of the RXRa gene in umbilical cords at birth, and bone size and density measured by DXA in childhood.

Methods: To identify potentially informative genomic regions in a panel of gene promoters, including RXRa, we undertook Methyl-DNA Immunoprecipitation followed by a commercial tiled oligomer microarray in 15 human umbilical cords. This located genomic regions with strong correlations between methylation status and childhood bone size and density assessed by DXA (Hologic Discovery); we used Sequenom MassARRAY to carry out in-depth analysis of the methylation status of 6 CpG’s in this part of the RXRa promoter in umbilical cords of 230 children at 4 years old from the Southampton Women’s Survey, with appropriate institutional ethics committee approval and participants’ informed consent.

Results: Higher % methylation at 4 out of 6 RXRA CpG sites measured was correlated with BMC corrected for body size (β = −2.1 to −3.4g/SD, *p* = 0.002 to 0.047) and lower offspring % bone mineral content (%BMC) (β = −0.02 to −0.04%/SD, *p* = 0.002 to 0.043). Similar relationships for %BMC were observed in a second independent cohort (*n* = 64). Maternal free 25(OH)-vitamin D index was negatively associated with methylation at one of these RXRA CpG sites (β = −3.3 SD/unit, *p* = 0.03).

Conclusions: In addition to the mechanistic insights afforded by associations between maternal free 25(OH)-vitamin D index, RXRA methylation in umbilical cord DNA, and childhood BMC, such epigenetic marks in early life might represent novel biomarkers for adverse bone outcomes in the offspring.

### 2.117. Variations in Vitamin D Deficiency among TB Patients by Ethnic Group and Country of Origin; Evidence from an Ethnically Diverse South London Population

#### Penn, N.; Pilarski, A.; Randhawa, J.; Milburn, H.

Background: Vitamin D deficiency is more common in tuberculosis (TB) patients, and within certain ethnic groups. Contemporary and historical evidence indicates the value of vitamin D in treating TB and genetic variation in vitamin D receptors has been linked to ethnic variations in TB. 70% of TB patients in the UK are foreign-born and a majority of TB cases occur in London.

Methods: The 25(OH)D (vitamin D) level of TB patients diagnosed at two South London hospitals between 2004 and 2012 was collected retrospectively from electronic hospital records (*n* = 728). 25(OH)D deficiency was defined as serum 25(OH)D level <20 nmol/L, 20–60 nmol/L was considered insufficient and >60 nmol/L normal. Logistic regression was used to calculate crude and adjusted odds ratios for 25(OH)D deficiency. Due to the small size of the population sub-group, chi-squared tests were used to identify factors associated with 25(OH)D deficiency in children.

Results: Only 9% (*n* = 67) of patients had a 25(OH)D level >60 nmol/L. 78% of patients (*n* = 555) were born outside of the UK. Having adjusted for age, sex, TB site, HIV and time of year, Black (aOR 6.75 95% CI 1.81–25.10) and Indian Asian (aOR 38.55 95% CI 8.06–184.3) patients were more likely to be deficient than White patients. Patients born in the Horn of Africa (Somalia, Sudan, Ethiopia, Eritrea, Kenya) and Indian subcontinent (India, Sri Lanka, Pakistan, Bangladesh) had 8–9 times the odds of deficiency compared to patients from the rest of Africa (aOR 8.98 95% CI 4.11–19.58) and Asia (aOR 8.43 95% CI 1.91–37.23) respectively. Extra-pulmonary TB was crudely associated with 25(OH)D deficiency (OR 1.77 95% CI 1.31–2.40) however the association was entirely explained by the country of origin of patients. Patients from Africa, Horn of Africa, Asian, and Indian subcontinent all had significantly increased odds of extra-pulmonary TB compared to European born patients having adjusted for other factors (*p* ≤ 0.002). For adults born in the UK there was no evidence that 25(OH)D deficiency was associated with ethnic group. 27% (*n* = 19) of children had a 25(OH)D > 60 nmol/L. 25(OH)D deficiency in children was strongly associated with being born outside of the UK (*p* = 0.001). There were no children with TB of White ethnicity and no evidence of a difference in the 25(OH)D levels between children of other ethnic groups.

Conclusion: Vitamin D deficiency is very common in this population of TB patients. Healthcare providers should be aware that vitamin D deficiency is likely to be currently under-diagnosed in TB patients. Country of origin appears to be a more important determinant of deficiency than ethnicity, and patients born in the Horn of Africa and Indian subcontinent are at particularly high risk of deficiency. Further studies to identify the underlying mechanisms relating to the very high levels of vitamin D deficiency in TB patients from the Horn of Africa and Indian subcontinent will have implications for understanding the role of vitamin D in TB aetiology as well as for the prevention and treatment of active tuberculous disease in these patients.

### 2.118. Cutaneous Vitamin D Synthesis Plus Dietary Vitamin D Intake Does not Reach Current UK Recommendations among Ethnic Minorities Living in North-east Scotland

#### Jamil, N.A.; Gray, S.R.; Macdonald, H.M.

Background: It has been assumed that most of the population in the UK obtain sufficient vitamin D from casual exposure to summer sunlight. However it is becoming clear that subgroups of the population are at risk of vitamin D deficiency because of inadequate sunlight exposure. The aim of this study was to determine the contribution from sunlight and diet to total vitamin D in a mixed ethnicity population living in North-east Scotland.

Methods: A total of 40 healthy adults (18 males, 22 females) aged 19–41 years from Southeast Asia, Middle East and Africa, currently residing in Aberdeen (57°N) took part in this study. All participants attended visits in spring (March–May), summer (June–August) and autumn (September–November). At each visit their skin colour was measured on the face and inner arm (the latter was used to determine skin type) using a CM-2600d spectrophotometer. Body surface exposure and dietary vitamin D intake were assessed by questionnaire. The participants wore polysulphone film badges for one week to estimate standard erytherma dose (SED). Total vitamin D was taken as the sum of sunlight-derived (calculated from SED, corrected for skin type and body surface area exposed) and dietary vitamin D intake.

Results: Participants from Africa had skin types V and VI and those from the Middle East had skin types II, III and IV. The Southeast Asians had the widest range of skin types (type II to type V). The SED was skewed for all groups. Non parametric Friedman’s ANOVA analysis (repeated measures) showed a significant seasonal variation in sunlight exposure with the highest in summer (median = 0.43 SED per day, IQR = 0.41), followed by spring (median = 0.12 SED per day, IQR = 0.40) and lowest in autumn (median = 0.06 SED per day, IQR = 0.01). Median facial skin colour was darkest in the summer and lighter in spring and autumn (Friedman’s ANOVA *p* = 0.011). Marginally more people exposed their arms or legs in summer compared to other seasons. When comparing skin types II and III with skin type IV to VI, there was no difference in SED or dietary vitamin D intake. As participants with higher pigmented skin require a longer period of sunlight exposure to synthesise the same amount of vitamin D as lighter skins, the former group will have produced less vitamin D. Diet provided 70–90 IU vitamin D per day (median) across the seasons and sunlight exposure was the major contributor (accounting for 70%) to total vitamin D in summer as shown below (median (IQR)).
**Season****Sun IU****Diet IU****Total IU****% Sun/Total****Spring**77(190)91(112)233(241)61(48)**Summer**235(311)81(88)323(319)74(28)**Autumn**22(36)75(118)91(123)23(34)


There was sporadic use of vitamin D supplements (*n* = 6 in spring; *n* = 7 for summer and autumn; with supplements providing between 57 IU and 5714 IU vitamin D daily). Of the participants who did not take supplements, none achieved the current UK recommended dietary intake of 400 IU vitamin D per day for those at risk of deficiency; and for the supplement users the recommendation was only achieved by one person in spring and 2 people in summer and autumn.

Conclusions: Low sunlight exposure in summer among ethnic minorities living at northerly latitudes poses a potential health risk that needs to be addressed urgently. Use of vitamin D supplements is recommended and needs to be consistent to avoid risk of vitamin D deficiency in this population.

### 2.119. Vitamin D and Tuberculosis in Adults in Selected Districts in the Provinces Baghlan, Bamyan and Badakhshan, Afghanistan

#### Sarin, P.; Manaseki-Holland, S.; Duffy, J.C.

Background: Evidence for an association between vitamin D and tuberculosis (TB) is inconsistent and no previous studies have been conducted in Afghanistan. This study aims to explore an association between TB and vitamin D levels in a rural Afghan population and to examine possible factors related to TB and vitamin D levels.

Methods: This was an individually 1:1 matched case-control study with 90 pairs. All eligible newly-diagnosed sputum-positive TB patients were recruited between May and June, 2009, from nine districts of the rural provinces Bamyan, Baghlan and Badakhshan. Through them, age-and sex-matched healthy contacts (sputum-negative) were recruited. 25-hydroxy vitamin D (25OHD) concentration of their blood was measured. A questionnaire was administered to explore variables potentially related to TB and vitamin D levels.

Results: The mean age was 35.78 years for cases and 35 years for controls; 37.8% of cases and 54.4% of controls were male. Of the total study population, 17.8% had vitamin D deficiency (≤50 nmol/L) and a further 6.67% had vitamin D insufficiency (≤75 nmol/L). Although cases had a lower level of 25OHD, no statistically significant differences were found between the case (mean 133.6, 95% CI 127.8–140.9 nmol/L) and control (mean 145.17, 95% CI 141.8–152.9 nmol/L) groups (*p* = 0.267). Sex was found to be associated with 25OHD in controls; women had a lower level of 25OHD than men (*p* = 0.006). This finding was not present in the patient group (*p* = 0.941). Univariate analysis showed that controls had higher calcium intake, educational levels, more subjects who had “ever worked” and sun exposure at the time of the study, than cases. When adjusting for confounding factors, controls had a higher BMI (mean control BMI 21.50, mean case BMI 18.86, *p* = 0.002) and more people working at the time of the study: 31.1% of controls and 5.6% of cases (*p* = 0.003). In analysis conducted upon the 65 sex-matched case-control pairs, sun exposure was found to be lower in TB patients at the time of the study and more cases had never worked compared to the controls (as well as BMI and working now).

Conclusions: 25OHD levels are not associated with TB amongst Afghans living in these rural provinces. Control women had a lower 25OHD level than men and this may be as traditionally Afghan women mainly remain indoors and wear a “burka” limiting their sun-exposure compared to men, who are predominantly farmers. No such difference was detected amongst cases, perhaps as both male and female patients were too ill to go out. Factors associated with TB are in line with other studies including a low BMI at diagnosis and occupation levels. Analysis of sex-matched case-control pairs does not reveal if the disease symptoms caused the patient to stop gaining sun exposure outdoors or if the low sun exposure may have caused the symptoms of TB to manifest. More cases had never worked and it is plausible that the people who never worked had a lower socioeconomic status with associated disadvantages of poorer nutrition, low BMI, damp poorer housing etc which has been linked to TB previously.

Limitations:

- Reliance on self-reporting of levels of sun exposure and dietary intake and for 3 months ago recall may not have been accurate.
- Sex-matching was not entirely successful but nevertheless multivariate analysis accounted for sex and similar findings emerged.
- Limited generalisability of the study to the rest of Afghanistan, as districts were not randomly sampled and populations.

### 2.120. Vitamin D Supplementation Does not Affect Bone Mineral Accrual in Vitamin D Replete Female Adolescent Athletes

#### Jessup, W.; Mitchell, S.; Beck, K.L.; Conlon, C.; Kruger, M.C.; Foskett, A.; von Hurst, P.R.

Background: Adolescence is a critical time for accrual of bone mineral density. Key modifiable determinants of achievement of peak bone mass are calcium intake, physical activity and vitamin D status. Female ballet dancers and gymnasts are known to have poor vitamin D status possibly due to heavy indoor training schedules limiting sun exposure, however it is likely that their high levels of physical activity ameliorate the detrimental effects of low vitamin D and dietary calcium. The aim of this study was to investigate the effect of supplemental vitamin D on bone mineral accretion in adolescent female athletes.

Methods: Adolescent female ballet dancers and gymnasts (*n* = 61) living in Auckland, New Zealand (NZ) and training a minimum of 5 h/week, were randomised to receive vitamin D_3_ (50,000 IU per month) or placebo for 12 months, 45 completed the trial. Bone mineral density (BMD) and bone mineral content (BMC) (total body, lumbar spine, proximal femur) and body composition were measured by DXA scan (Hologic Discovery A) at 0 and 12 months. Dietary calcium intake was estimated from a 4-day food diary; serum 25(OH)D, PTH and oestradiol were measured from venous blood samples at 0, 6 and 12 months. Physical activity was monitored with a weekly training diary. All blood samples were stored and assayed at the end of the study.

Results: At baseline mean age (14.4 years), weight (52.1 kg) and BMI (19.9 kg/m^2^) were recorded. Mean (SD) dietary calcium intake was 898(329) mg per day; s25(OH)D was 72.9(19.1) nmol/L, range 36–129 nmol/L, 85% of girls were above 50 nmol/L (min. for adequacy in NZ). At 12 months there was a non-significant trend upwards in the intervention group and a significant difference between groups (76.4 *vs.* 68.3 nmol/L, *p* = 0.05). Baseline was PTH 2.55(1.40) pmol/L, and did not change. BMD and BMC at all sites increased in both groups, but there was no difference in change between groups. Key predictors of total BMD and BMC were age, calcium intake and lean mass.

Conclusions: Contrary to previous research findings in ballet dancers and gymnasts, the participants in this study were vitamin D replete. Moreover, the supplemental dose of 50,000 IU/month did not significantly raise the 25(OH)D concentrations in the intervention group over the 12 month period although there was a significant increase from baseline to 6 months. All the participants showed an increase in bone mineral accrual over the study period, as would be expected in girls of this age. Although calcium intake was below the recommended 1300 mg/day for this age group, it was above the mean for NZ women (2008 adult nutrition survey) and PTH levels were within reference range. These findings suggest that; (1) there is no additional benefit to calcium absorption or bone mineralisation from a blood level for 25(OH)D above ~75 nmol/L; (2)despite adequate vitamin D and regular physical activity lower dietary calcium intake is still a predictor of lower total body BMD and BMC; and (3) in certain population groups with baseline 25(OH)D > 75 nmol/L, vitamin D supplementation of 50,000 IU/month does not significantly increase 25(OH)D concentrations over a 12 month period, but possibly prevents seasonal decline.

### 2.121. The Dose Response Relationship of Vitamin D Supplementation and Plasma 25-Hydroxy Vitamin D among 8410 Healthy Volunteers

#### Veugelers, P.; Zwicker, J.D.; Ekwaru, P.J.

Background: Understanding of the effect of vitamin D supplementation on plasma 25(OH)D levels is essential to optimizing nutritional status and health. The incremental consumption of 40 IU/day of vitamin D_3_ has been estimated to raise plasma 25(OH)D by about 1 nmol/L (0.4 ng/mL). The dose response relationship between vitamin D supplementation and plasma 25(OH)D levels is thought to be biphasic (exponential at low intakes and linear at high), however this is not well documented for supplementation levels that exceed 2000 IU per day for large populations of healthy individuals. The objective of this study is to characterize the relationship of supplementation and plasma 25(OH)D level in a large sample of healthy volunteers.

Methods: We analyze clinical data from a large, not for profit preventative health care program in Alberta Canada. The dose response relationship was characterized using linear, quadratic, cubic, and piecewise generalized estimation equations (GEE) of 10,298 observations of 8410 healthy adult volunteers. Using these equations we further quantified the importance of age, gender, and body weight status, as well as the probability that supplementation would result in critical 25(OH)D levels.

Results: Participants reported to supplementation levels ranging from 400 to 20,000 IU per day. The relationship with 25(OH)D is best characterized with piece wise regression: supplementation levels below 7550 IU per day, produced a linear increase in plasma 25(OH)D of 7.57 nmol/L per 1000 IU of supplementation. Above 7550 IU per day, plasma 25(OH)D increased with 2.34 nmol/L per 1000 IU. Older age and excess body weight appeared important determinants of plasma 25(OH)D. Supplementation with 600 IU per day achieved for 90.9% of participants to reach 25(OH)D plasma levels of 50 nmol /L or more. For normal weight, overweight and obese participants this was 94%, 91%, and 87.4% respectively. Supplementation of 9862 IU per day is needed to achieve that 97.5% of participants reaches levels of 50 nmol /L or more. Supplementation with up to 20,000 IU per day did not contribute to a substantial risk for reaching toxic levels of 350 nmol 25(OH)D/L.

Conclusions: The findings suggest that the current IOM recommendations of 600 IU per day may not achieve that 97.5% of healthy adults achieve a plasma level of 50 25(OH)D nmol/L. This is particularly true for overweight and obese individuals. This gives rise to the suggestion that recommendations for supplementation should be body weight status specific. Existing estimates based on supplementation levels of less than 2000 IU per day include that 40 IU raises 25(OH)D by about 1 nmol/L. These estimates are three fold higher than our estimates for healthy volunteers who supplement with up to 7550 IU per day and nine fold higher for those who supplement with 7550 to 20,000 IU per day. Intakes of up to 20,000 IU per day are unlikely to result in vitamin D toxicity.

### 2.122. Vitamin D Supplementation Ranging from 400 to 20,000 IU per Day and Calcium Homeostasis among Healthy Volunteers

#### Veugelers, P.; Kimball, S.; Ekwaru, P.J.

Background: As vitamin D is essential to calcium regulation, the concern has been raised that high levels of Vitamin D may lead to hypercalcemia (plasma levels exceeding 2.6 mmol/L) that, in turn, may increase the risk of adverse health events such as cardiac arrest, renal failure, and hepatic injuries. Our objective is to examine the influence of vitamin D supplementation on serum calcium levels and the risk for hypercalcimia in healthy volunteers in the Pure North health program. We also studied the relationship between 25-hydroxy vitamin D (25(OH)D) and serum calcium in this sample.

Methods: The examination of the influence of vitamin D supplementation and 25(OH)D on serum calcium levels was based on 8906 baseline and follow-up assessments of vitamin D supplementation, serum 25(OH)D and calcium of healthy participants of the Pure North wellness program. We applied GEE regression methods to quantify the associations of vitamin D supplementation and 25(OH)D with serum calcium, while considering the confounding potential of age, gender, and body weight status.

Results: Serum calcium concentrations increased with 0.0008 mmol/L per increment of 1000 IU/day vitamin D (*p*-value < 0.008) and with 0.0001 mmol/L per increase of 1 nmol/L 25(OH)D (*p*-value < 0.001). The risk for hypercalcemia in this sample of healthy volunteers was low and did not increase as a result of higher levels of vitamin D supplementation. The risk for hypercalcemia was positively associated with higher 25(OH)D serum levels: at serum 25(OH)D levels above 300 nmol/L the average risk for hypercalcemia had increased to a level that 6% had serum calcium levels above 2.6 mmol/L. This risk was similar across body weight groups but higher for females and older participants.

Conclusion: These finding confirm that healthy subjects supplementing with vitamin D at modest levels are not at increased risk for hypercalcemia and suggest that the same applies for supplementation levels as high as 20,000 IU per day. However, where supplementation results in high 25(OH)D serum levels, the risk for hypercalcemia may increase and particularly among female and older individuals. We recommend that 25(OH)D and serum calcium be monitored when supplementing higher doses of vitamin D.

### 2.123. Environmental, Personal and Genetic Determinants of Response to Vitamin D Supplementation in Older Adults

#### Waterhouse, M.; Tran, B.; Armstrong, B.K.; Baxter, C.; Ebeling, P.R.; English, D.R.; Kimlin, M.G.; Venn, A.; Gebski, V.; Hill, C.; Lucas, R.; Whiteman, D.C.; Webb, P.M.; Neale, R.E.

Background: Suboptimal vitamin D status may be associated with a variety of adverse health outcomes. It can be simply and effectively treated through vitamin D supplementation. However, response to supplementation varies. We aimed to examine the environmental and genetic determinants of change in 25 hydroxy vitamin D (25(OH)D).

Methods: This study used data from a pilot randomised controlled trial of vitamin D supplementation (pilot D-Health) in which 644 Australian adults aged 60–84 years were randomly assigned to receive monthly doses of placebo or 30,000 IU or 60,000 IU vitamin D_3_ (cholecalciferol) for 12 months. Baseline characteristics potentially related to variability in circulating 25(OH)D were obtained from a self-reported questionnaire at study entry. Eighty-eight single nucleotide polymorphisms (SNPs) in 41 candidate genes related to vitamin D status were genotyped using Sequenom MassArray technology. Serum 25(OH)D levels before and after the intervention were measured using the Diasorin Liaison platform. Stepwise variable selection was used to generate multiple linear regression models based on environmental and personal factors, and/or SNPs, from which we identified significant predictors of change in serum 25(OH)D level.

Results: This analysis included 385 of the 430 participants randomised to one of the two vitamin D doses who provided post-supplementation blood samples and had adequate SNP data. The mean serum 25(OH)D level increased from 42 nmol/L (SD 13) to 64 nmol/L (SD 17) in the 30,000 IU group and from 42 (SD 14) to 78 nmol/L (SD 20) in those assigned to 60,000 IU/month. Determinants of response to supplementation were baseline level of 25(OH)D, supplement dose, body mass index, and self-reported health status. SNPs in the following genes were significant at *p* < 0.05: CYP2R1, IRF4, MC1R, VDR, HERC2. The proportion of variability explained by the model was marginally higher in the model with SNPs included than in that without them (32% *vs.* 26%).

Conclusion: This work shows that genetic factors are associated with change in 25(OH)D after vitamin D supplementation. This suggests that a “one dose fits all” approach to supplementation might not be appropriate, or that there is variability in the physiologically “normal” levels of 25(OH)D. Further work is needed to disentangle these possibilities.

### 2.124. Seasonality of Month of Birth in T1D Patients Exposed and Unexposed to Fortified with Vitamin D Food during Gestation

#### Jacobsen, R.; Heitmann, B.L.

Background: Type 1 diabetes (T1D) is one of the most common chronic diseases starting in childhood resulting from destruction of insulin-secreting beta cells. Incidence of type T1D has been increasing during the past decades. The exact pathogenesis of T1D remains unknown. Vitamin D was hypothesized to have a protective effect. Low vitamin D status is prevalent among industrialized populations. Status of pregnant women is of particular concern as low levels may have consequences for the health of the offspring. To date, the influence of vitamin D status during gestation on long-term risk of T1D has not been widely studied. The main source of vitamin D is its synthesis in the skin due to exposure to sunlight. Oral intake augmented by fortification or supplementation is necessary in countries of high latitudes and seasonal variation in sunlight. In Denmark, therefore, national programs for food fortification with vitamin D are considered. The health benefits of such programs have not been studied. The phenomenon of seasonality of month of birth in T1D was introduced by Rothwell *et al.* who found that more T1D patients were born during the spring and early summer and fewer during the winter months. It was concluded that environmental influences during gestation stand behind the phenomenon. Later, geographical latitude with its influence on vitamin D synthesis was listed among the reasons. Thus, our study aimed to assess if intake of foods fortified with vitamin D during gestation had an effect on the risk of T1D later in life. This sub-study tested the hypothesis that seasonality of month of birth in T1D is more pronounced among individuals who were not exposed to vitamin D fortification during gestation.

Methods: From 1972 to 1976 fortification of low fat milk (2.5–3.8 µg/100 g milk) in Denmark was permitted. Individuals born in two years after milk fortification start were considered as exposed to vitamin D fortification during gestation; individuals born in two years before milk fortification end were considered as unexposed. The civil registration numbers of all individuals born in Denmark in the exposure-related cohorts were received and their outcomes on T1D diagnosis were obtained through the linkage to the Danish National Patient Registry. The analyses were conducted by Cox regression adjusted for linear time trend in T1D, calculating T1D hazards ratio for those born at a particular month *vs.* the rest of the year separately in the exposure-related cohorts. Statistical significance between the exposure-related cohorts was tested in the models for interaction between the exposure and a month of birth.

Results: There were no statistically significant differences in seasonality of month of birth in T1D in individuals exposed or unexposed to vitamin D fortified milk during gestation. The differences between exposed and unexposed individuals were not statistically significant either.

Conclusions: To assess the impact of exposure to vitamin D during gestation for later T1D risk we studied seasonality of month of birth in a specific context of food fortification practices in Denmark. We hypothesized that gestational exposure to milk fortified with vitamin D would attenuate seasonality of month of birth. Our hypothesis was not confirmed. Absence of the seasonality in the first place, non-mandatory milk fortification policy, and too low doses of fortified milk may partially explain our findings.

### 2.125. The Effects of Vitamin D_2_ or D_3_ Supplementation on Glycaemic Control and Related Cardiometabolic Parameters in People at Risk of Type 2 Diabetes: Results from a Randomised Double-Blind Placebo-Controlled Trial in the UK

#### Menon, R.K.; Forouhi, N.G.; Sharp, S.J.; Mannan, N.; Timms, P.M.; Martineau, A.; Greenwald, S.; Rickard, A.P.; Boucher, B.J.; Chowdhury, T.A.; Griffiths, C.J.; Griffin, S.J.; Hitman, G.A.

Background: Convincing epidemiological evidence exists for an inverse association between 25-hydroxy vitamin D (25-OHD) concentration and risk of type 2 diabetes (T2D), but whether this is causal remains uncertain. We investigated the effect of vitamin D supplementation on the metabolic status of individuals at increased risk of developing T2D.

Methods: In a double-blind placebo-controlled randomised trial, 340 adults (172 from Cambridge and 168 from London) at increased risk of T2D (non-diabetic hyperglycaemia or positive diabetes risk score) were randomised into one of three groups of oral therapy: placebo, 100,000 IU Vitamin D_2_ (ergocalciferol) or 100,000 IU Vitamin D_3_ (cholecalciferol) at monthly intervals for four months. The primary outcome was change in glycated haemoglobin (HbA1c) level between baseline and 4 months. Secondary outcome measures included blood pressure, body mass index, waist circumference, fructosamine, lipid and apolipoprotein levels, liver function tests, C-reactive protein level and safety of supplementation. Participants in London also underwent measurement of pulse wave velocity (PWV). All analyses were by intention-to-treat.

Results: 25-OHD_2_ levels rose in the vitamin D_2_ supplemented group from (mean (SD)) 5.2 (4.1) to 53.9 (18.5), and 25-OHD_3_ levels rose from 45.8 (22.6) to 83.8 (22.7) in the vitamin D_3_ supplemented group. There were no differences between the placebo and vitamin D groups for HbA1c (HbA1c difference: D_2_ *vs.* placebo −0.51 mmol/mol (95% CI −1.16, 0.14); D_3_ *vs.* placebo 0.19 mmol/mol (−0.46, 0.83)). There were no meaningful differences by group in secondary outcomes. Compared with placebo, PWV was reduced in both D_2_ (−0.68 m/s (−1.31, −0.05)) and D_3_ (−0.73 m/s (−1.42, −0.03)) supplemented groups. There were no important safety issues related to supplementation.

Conclusions: Supplementation with vitamin D_2_ and D_3_ raised levels of 25-OHD_2_ and 25-OHD_3_ respectively, but had no effect on HbA1c level during the study period. The modest reduction in PWV with both D_2_ and D_3_ *vs.* placebo is suggestive of a beneficial effect of vitamin D supplementation on arterial stiffness.

### 2.126. Maternal 25-Hydroxy-Vitamin D Status in Late Pregnancy is Associated with Offspring Grip Strength at 4 Years of Age

#### Moon, R.J.; Aihie Sayer, A.; Ntani, G.; Davies, J.H.; Javaid, M.K.; Robinson, S.M.; Godfrey, K.M.; Inskip, H.M.; Cooper, C.; Harvey, N.C.

Background: Maternal 25-hydroxy-vitamin D (25(OH)D) status in pregnancy has been associated with offspring bone development and adiposity. There is evidence to support a role for vitamin D in muscle function, yet little is known about the role of antenatal 25(OH)D exposure in programming muscle development. We therefore investigated the associations between maternal serum 25(OH)D status at 34 weeks gestation and offspring muscle mass and strength at 4 year of age.

Methods: The Southampton Women’s Survey (SWS) is a population based prospective mother-offspring birth cohort study in Southampton, UK. Maternal serum 25(OH)D was determined at 34 weeks gestation by radioimmunoassay (Diasorin). At 4 years of age, offspring body composition was assessed by Dual Energy Xray Absorptiometry (Hologic Discovery) and hand grip strength measured by hand dynamometry (Jamar Dynamometer). In a subset of children, physical activity was assessed by 7 day accelerometry (Actiheart). Associations were explored using linear regression to yield standardised beta coefficients (SD/SD).

Results: 678 mother-child pairs were included in the analysis. Median maternal 25(OH)D was 61 nmol/L (IQR 43–88 nmol/L), and only 9.2% of mothers were using vitamin D supplementation (at least 400 IU/day) in late pregnancy. Maternal serum 25(OH)D was positively associated with offspring height-adjusted grip strength (β = 0.10 SD/SD, *p* = 0.013). This relationship persisted after adjustment for maternal potential confounding factors and duration of breastfeeding (β = 0.08 SD/SD, *p* = 0.040). Furthermore, the addition of the child’s physical activity (*n* = 326) to the model did not alter the relationship (β = 0.13 SD/SD, *p* = 0.011). There was no significant association between maternal 25(OH)D and offspring total lean mass (β = 0.06 SD/SD, *p* = 0.15), but a positive association with percent lean mass was identified (β = 0.11 SD/SD, *p* = 0.006). This was however attenuated by the addition of confounding factors (β = 0.07 SD/SD, *p* = 0.062).

Conclusions: Antenatal exposure to 25(OH)D might influence muscle development through an effect primarily on muscle strength rather than muscle mass. Intervention studies are now needed to confirm this finding.

### 2.127. Long-Term Monthly Vitamin D_3_ Supplementation and Blood Pressure in Healthy Adults: A Randomized Controlled Trial

#### Scragg, R.; Slow, S.; Stewart, A.W.; Jennings, L.C.; Chambers, S.T.; Priest, P.C.; Florkowski, C.M.; Camargo, C.A., Jr.; Murdoch, D.R.

Background: Elevated blood pressure (BP) is a major risk factor for cardiovascular disease. There is accumulating evidence from meta-analyses of cohort studies that vitamin D deficiency, as measured by low serum levels of 25-hydroxy vitamin D (25(OH)D), predicts increased risk of all-cause mortality, cardiovascular disease and hypertension. However, there is uncertainty as to whether low vitamin D status is a true cause of these outcomes, or simply a marker of other lifestyle variables. This uncertainty can only be resolved by well-designed randomized controlled trials (RCTs) to determine the effect of vitamin D supplementation on BP. Previous RCTs mainly have given vitamin D for short periods (<6 months) or at too low doses. This study aimed to determine if long-term high-dose vitamin D over 18 months lowered BP.

Methods: A double blind RCT of adults (*n* = 322), recruited from a health care organization or university and randomized to receive either vitamin D_3_ 200,000 IU for two months, followed by 100,000 IU monthly up to 18 months (*n* = 161), or placebo (*n* = 161). BP was measured at baseline, 5- and 18-months.

Results: Subjects had a mean (SD) age of 47.6(9.7) years, 75% were female, and 94% were of European ancestry (white). Mean (SD) 25(OH)D changed from 73 (22) nmol/L at baseline to 124(28) nmol/L at 18-months in the vitamin D group, and from 71(22) to 56(22) nmol/L in the placebo group. Mean BP was similar for the vitamin D and placebo groups at baseline (123.4/76.3 *vs.* 122.6/75.6 mmHg; respectively). The mean change (95% confidence interval) in BP from baseline in the vitamin D group compared with placebo group: at 5-months was −2.3(−4.3, −0.3) mmHg for systolic (*p* = 0.021) and −0.6(−2.8, 1.6) mmHg for diastolic (*p* = 0.61); and at 18-months was −0.6(−2.8, 1.6) mmHg for systolic (*p* = 0.61) and 0.5 (−1.1, 2.2) mmHg for diastolic (*p* = 0.53). The isolated decrease in systolic BP among the vitamin D group at 5-months follow-up is most likely a chance finding, since this small difference was not observed at 18-months.

Conclusions: Long-term monthly vitamin D supplementation, which increased mean 25(OH)D concentration above 100 nmol/L for 18 months, had no effect on systolic or diastolic BP in predominantly white, healthy adults without severe vitamin D deficiency. However, beneficial BP-lowering effects cannot be ruled out for other populations.

### 2.128. Direct-to-the-Public Vitamin D Testing Compared to GP Referrals

#### Shea, R.L.; Berg, J.D.

Background: In 2011 we introduced a direct-to-the-public vitamin D testing service using dried blood spots (DBS). Since then we have received samples from all over the country. We were interested to see how this group of non-typical “patients” vitamin D status compared with our local inner city Birmingham GP population.

Methods: All 25-hydroxyvitmain D (25OHD) results for DBS (*n* = 4480) and GP serum samples (*n* = 28,660) from June 2012 to May 2013 were reviewed. Age and sex were recorded and patients categorised into the following statuses: Severely Deficient: <15 nmol/L, Deficient: 15–30 nmol/L, Insufficient: 30.1–50 nmol/L, Adequate: 50.1–220 nmol/L, High to potentially toxic: 220.1–500 nmol/L, Toxic: >500 nmol/L.

Results: The populations were significantly different in the proportion of samples coming from each gender (*p* < 0.001) with women supplying 69.1% and 63.2% of GP and DBS samples respectively. The distribution of ethnicities between both populations was also significantly different (*p* < 0.001). The distribution of age and 25OHD results was not normally distributed for either population and there was significant differences between them (*p* < 0.001 and *p* < 0.001). Age ranged from 0–99 years (median = 48) for DBS and 0–100 years (median = 42) for GP samples. 90.2% of DBS samples came from Caucasians with only 4.4% of DBS samples coming from Asians whereas only 22.8% of GP samples came from Caucasians and 57.8% of serum samples came from Asians. The 25OHD concentration ranged from 10.3–700.8 nmol/L (median = 53.1 nmol/L) for DBS samples and 10.3–735.4 nmol/L (median = 27.7 nmol/L) for serum samples. The majority of serum GP samples were deficient (36.7%) with only 21.1% of patients tested displaying adequate levels of 25OHD. A significantly different pattern was found for the DBS samples, with 53.2% of patients showing adequate levels and only 14.3% in the severely deficient category (*p* < 0.001). There was a relationship between vitamin D status and ethnicity for serum and DBS samples (*p* < 0.001 and *p* < 0.001 respectively) with Caucasians more likely to have an adequate status and Asians more likely to be severely deficient. Only 0.07% of GP samples could be considered at risk of toxicity (>220 nmol/L) compared with 1.1% of DBS samples—a significant difference (*p* < 0.001). One GP sample was >500 nmol/L compared with 4 DBS samples.

Conclusions: The two populations are markedly different in age, sex, ethnicity, 25OHD concentration and status. The DBS population have a more adequate 25OHD status compared to the GP population. This may reflect the reasons for testing—GPs are looking for deficiency in order to treat, whereas many of the people using our DBS service are checking to see if they are deficient and then checking to see if they are adequate once they have commenced supplementation. This is also seen in the significantly higher rate of high to toxic samples found in the DBS population. The differences in ethnicity seen in the two populations will also contribute to the lower concentration of 25OHD seen in the GP population, which had a significantly higher proportion of Asians. A substantial number of the general public represented by our DBS population are still deficient and could benefit from increasing their levels, although a significant number may be inadvertently over-supplementing themselves or their relatives and so it is vital that public awareness is increased on the risks of self-administration and over-treatment.

### 2.129. Incidences of High to Toxic 25-Hydroxy Vitamin D Levels amongst Users of a Direct-to-the-Public Blood Spot Vitamin D Testing Service

#### Shea, R.L.; Berg. J.D.

Background: In 2011 we introduced a direct-to-the-public vitamin D testing service using dried blood spots (DBS). We noticed that the rate at which people were displaying a high to toxic (>220 nmol/L) level of 25-hydroxyvitmain D (25OHD) was much greater in this new population than what we saw in our traditional GP population. We wanted to find out more about the supplementation patterns of our DBS service users and to see if the high levels were due to prescription supplementation or self-prescribing.

Methods: Between January and November 2013, all DBS patients with 25OHD > 220 nmol/L were contacted to inform them of their level. During this contact we looked at the extent of medical supervision, amount of supplements being taken, brand and source of supplements and length of time supplementing.

Results: In total, 69 users (1.5%) of the DBS service had 25OHD > 220 nmol/L. Two users did not wish to discuss their results and 2 could not be contacted. Of the remaining 65 users, only 2 were under medical supervision. One had been prescribed vitamin D, the other was taking up to 100,000 IU/day against the advice of her consultant. The later patient was one of 6 (out of 69) whose 25OHD was >500 nmol/L and the only one under medical supervision. The daily consumption of supplements of these 6 users ranged from 11,000–100,000 IU/day. The range of supplements taken by all 65 users contacted ranged from 3000–100,000 IU/day. 55% of users with 25OHD > 220 nmol/L were taking 10,000 IU/day or less and the range of 25OHD seen on this level of supplement was 221–648 nmol/L. Of the 62 people who knew where they got their supplements from, 71% got them from the internet. 44% of users had previously had their 25OHD measured by our DBS service prior to having their high test result. Of these 43% had a less than adequate status initially (12–47 nmol/L). The rest had an adequate status ranging from 51–159 nmol/L.

Conclusions: Our results show the wide range of vitamin D supplement regimes used by members of the public that have resulted in them having high to toxic levels of 25OHD. A startlingly high proportion of users were taking supplements at a level higher than the no-observed-adverse-effect-level of supplement (10,000 IU/day) according to the Institute of Medicine. All but one were doing so without medical supervision. Of concern is that people taking 10,000 IU/day or less are showing high to toxic levels of 25OHD, although we do not know how many, if any, of these users are displaying signs of vitamin D toxicity. The vast majority of patients obtained their supplements from the internet. Many of the websites that patients bought their supplements from did not appear to clearly highlight the risk of toxicity from vitamin D, although they all described the potential health benefits of taking vitamin D supplements. Our results highlight the fact that it is vital that public awareness is increased on the risks of self-administration and over-treatment. How many people are over supplementing unknowingly as they have not had their levels checked?

### 2.130. Vitamin D Levels in Pregnant Women in Inner City Birmingham: A Four Year Overview

#### Shea, R.L.; Ford, L.; Berg. J.D.

Background: Maternal 25-hydroxy vitamin D (25OHD) is important for many reasons especially as it determines the risk of neonatal hypocalcaemia and early rickets. Pregnant women deficient in 25OHD can adversely affect foetal and infant skeletal growth, bone ossification and tooth enamel formation. There is also emerging, although inconsistent, evidence that maternal 25OHD status could be associated with other foetal health outcomes such as birth weight, head circumference and risk of baby being small for gestational age. An adequate maternal status may be important for proper foetal and placental development and proper immune response and function during pregnancy. Given its importance, we wanted to assess the 25OHD status of pregnant women in inner city Birmingham and to see if this was changing over time.

Methods: Aliquots of serum were taken from samples received in the laboratory for Downs testing (16–18 weeks gestation) during Autumn 2010–2013. Samples were analysed for 25-hydroxy vitamin D_2_ and 25-hydroxy vitamin D_3_ and results combined to give a total 25OHD concentration. Samples were prepared using a liquid/liquid extraction and analysed on a Waters ACQUITY Ultra-Performance LC and Quattro Premier XE MS/MS. Patient age and ethnicity were recorded and results completely anonymised. Ethnicity was recorded as Caucasian, Asian, Black/Afro-Caribbean or other. 25OHD status was defined as: Severely Deficient: <15 nmol/L, Deficient: 15–30 nmol/L, Insufficient: 30.1–50 nmol/L, Adequate: >50 nmol/L.

Results: In total 743 samples were analysed (2010 *n* = 204, 2011 *n* = 126, 2012 *n* = 204 and 2013 *n* = 209). Age and 25OHD were not normally distributed in any of the years analysed and the proportion of people in the different ethnic categories was significantly different (combined data *p* < 0.001). The distribution of age and ethnicity for the sample population did not change over the years (*p* = 0.924 and *p* = 0.397 respectively), however the proportion of people falling into the different 25OHD status categories did significantly change over the years (*p* < 0.001). In 2010, only 24% of the samples analysed were found to have adequate 25OHD status. This rose to 51.2% in 2013. The distribution of 25OHD concentration changed significantly over the years (*p* < 0.001), with the median total 25OHD rising from 31.5 nmol/L in 2010 to 50.6 nmol/L in 2013. The distribution of total 25OHD concentration across the different ethnicities was also significantly different (*p* < 0.001), with 59.6%, 30.1% and 31% of Caucasian, Asian and Black/Afro-Caribbean samples, respectively, being adequate.

Conclusions: Our data suggests that from 2010, the 25OHD status of pregnant women is slowly improving, with 2013 being the first year that just over half of the samples tested showed an adequate status. This may link in with the increased availability or awareness of vitamin D supplementation through the Healthy Start scheme, launched in 2006, and other initiatives. In 2011 the Healthy Start supplements for pregnant women were made free of charge in Birmingham to all women, not just to those receiving benefits. Despite this, our data shows that an unacceptably high proportion of pregnant women in inner city Birmingham have a less than adequate 25OHD status, especially those women from ethnic minorities. This raises the questions of whether enough pregnant women are taking supplements, or if they are taking the supplements, is the level of supplementation enough to increase their levels into the adequate status?

### 2.131. Do Serum Free and Bio-Available 25-Hydroxy Vitamin D Better Reflect Biological Activity than Serum Total 25-Hydroxy Vitamin D?

#### Jorde, R.; Johnsen, M.; Grimnes, G.; Figenschau, Y.; Torjesen, P.A.; Almås, B.

Background: The vitamin D metabolites are transported in the circulation by vitamin D-binding protein (DBP) which is a water-soluble carrier-protein. About 85%–90% of 25(OH)D and 1,25(OH)2D are bound to DBP. A considerable amount is also bound to albumin and less than 1% of 25(OH)D and 1,25(OH)_2_D circulate in the bloodstream freely. According to the “free-hormone-hypothesis” only the free form is biologically active. Because albumin binds 25(OH)D weakly one may assume that 25(OH)D dissociates from albumin during tissue perfusion. Therefore, bio-available 25(OH)D refers to the sum of the free and the albumin-bound fraction of 25(OH)D. Genetic differences in DBP may affect the binding to 25(OH)D and thereby the amount of free 25(OH)D. In the present study 25(OH)D, DBP and albumin were measured and the free and bio-available (free + albumin-bound) 25(OH)D calculated in sera obtained from 265 postmenopausal women with low bone mass density (BMD).

Methods: Based on genotyping, the six common DBP phenotypes were identified and the free and bio-available 25(OH)D calculated according to the corresponding binding coefficients. Linear regression with adjustment for age and BMI were used to evaluate relations between measures of 25(OH)D and PTH and BMD.

Results: The calculated amount of free and bio-available 25(OH)D was 0.03% and 13.1%, respectively, of the measured total serum 25(OH)D. Adjusting for DBP phenotype affected the calculated free and bio-available 25(OH)D levels up to 37.5%. All measures of 25(OH)D correlated significantly with PTH, whereas a significant association with BMD was only seen for the free and bio-available 25(OH)D measures. Adjusting for the DBP phenotypes improved the associations. These relations were almost exclusively seen in subjects not using vitamin D and/or calcium supplements.

Conclusions: The free and bio-available forms of 25(OH)D may be a more informative measure of vitamin D status than total 25(OH)D. Adjustment for DBP phenotype may improve this further. However, the findings need confirmation in larger studies.

### 2.132. Vitamin D Activation and TLR Signalling in Human Bladder Epithelial Cells; a Potential Mechanism for Regulation of Immune Responses

#### Jefferson, K.; MacDonald, D.; Bland, R.

Background: The urinary bladder needs to be impermeable to the constituents of urine and prevent invasion by infective organisms. Key to the immunological response to bacterial infection is pathogen recognition by toll-like receptors (TLR). The immune system also has a vital role to play in cancer prevention. Non muscle-invasive bladder cancer is highly immunoresponsive and treatment with intravesical bacillus Calmette-Guerin (BCG), which is TLR dependent, reduces tumour recurrence. Development of bladder cancer is associated with low levels of vitamin D. Both urinary tract infections and 90% of bladder cancers involve the transitional epithelium, which lines the urinary bladder. Evidence from other tissues indicates that local synthesis of active vitamin D (1,25-dihydroxy vitamin D; 1,25(OH)_2_D_3_) influences immune responses and in particular TLR signalling. We therefore investigated the expression of components of vitamin D (vitamin D receptor [VDR], 25-hydroxy vitamin D_3_-1α-hydroxylase [CYP27B1; 1α-OHase], 24-hydroxylase [CYP24A1; 24-OHase], RXR) and TLR (TLR 1,2,4, MyD88 and CD14) signalling in human urinary bladder epithelial cells.

Methods: Protein and mRNA expression was demonstrated by Western blotting, immunocytochemistry and RT-PCR. Enzyme activity was assessed by EIA. Studies used the human bladder transitional epithelial (urothelial) cell lines derived from well differentiated (RT4) and poorly differentiated (T24/83) transitional cell carcinomas. HKC-8 cells (human proximal tubule cells) were used as positive controls where required. Cells were incubated with 1,25(OH)_2_D_3_ (10 nM) or 25(OH)D_3_ (100 nM) for 4–48 h. Synthesis of 1,25(OH)_2_D_3_ was measured by EIA and induction of 24-OHase mRNA. Statistical analysis was performed using one-way ANOVA and Tukey’s multiple comparison tests.

Results: RT-PCR analyses demonstrated that both cell lines expressed VDR and 1α-OHase mRNA. Western blot analysis identified single protein bands (identical to HKC-8 cells), which corresponded to the sizes of the VDR and 1α-OHase. In addition, the cells expressed RXR and megalin mRNA. 24-OHase mRNA, which was almost undetectable in unstimulated cells, was significantly increased by treatment with 10 nM 1,25(OH)_2_D_3_ (3–24 h; *p* < 0.05). 24-OHase activity was confirmed as conditioned media from cells treated with 10 nM 1,25(OH)_2_D_3_ for 24 h failed to induce 24-OHase mRNA, demonstrating that metabolism of the 1,25(OH)_2_D_3_ had occurred. 24-OHase mRNA was also significantly increased by 25(OH)D_3_ (6–24 h; *p* < 0.05) indicating epithelial 1α-OHase activity. Synthesis of 1,25(OH)_2_D_3_ was confirmed by EIA and was attenuated following pre-treatment with ketoconazole. Both cell types expressed TLR 1, 2 & 4, mRNA and the TLR partners MyD88 and CD14. Cathelicidin mRNA (a vitamin D responsive antimicrobial peptide; hCAP-18/LL37) was undetectable in both cell lines, but was induced by 1,25(OH)_2_D_3_ and 25(OH)D_3_ in RT4 cells (6–24 h; *p* < 0.05).

Conclusions: These data demonstrate that urothelial cells express functional vitamin D signalling and are able to synthesize sufficient 1,25(OH)_2_D_3_ to stimulate a local immune response. This could facilitate active immune surveillance and may play a role in the detection and removal of urinary pathogens and abnormal cells. In addition, it could be vital for successful intravesical BCG immunotherapy. This suggests that maintaining adequate serum 25(OH)D_3_ levels is important for bladder epithelial function.

### 2.133. Validation of a Vitamin D Food Frequency Questionnaire Using the Method of Triads

#### Weir, R.R.; Carson, E.L.; Mulhern, M.S.; Strain, J.J.; Laird, E.; Healy, M.; Pourshahidi, L.K.

Background: Food frequency questionnaires (FFQ) are often used in large epidemiology studies to assess the habitual intake of foods and/or specific nutrients of interest, with relative ease and minimal cost. Although humans obtain most of their vitamin D via the action of ultraviolet (UV)-B radiation on 7-dehydrocholesterol in the skin (Willett, W. *Nutritional Epidemiology*, 3rd ed.; 2013, pp. 70–89), this endogenous synthesis is limited by a number of factors, most notably latitude and season. In the UK and Ireland (at approximately 50–60°N) (Ocké, M.C. et al. *AJCN* 1997, *65*, S1240–S1245), UV-B intensity is insufficient to synthesise vitamin D for 6 months of the year, spanning October-March. As other factors in today’s modern lifestyles (e.g., sunscreen/cosmetic use, indoor occupations, lack of outdoor activities) further limit such synthesis during the summer months, the general population is becoming increasingly reliant on dietary sources of vitamin D to maintain adequate vitamin D status (Gillie, O. *Mol. Nutr. Food Res*. 2010, *54*, 1–16). The aim of this study was to validate a new FFQ to assess habitual dietary vitamin D intake, using the method of triads (Ocké, M.C. et al. *AJCN* 1997, *65*, S1240–S1245).

Methods: A total of 49 apparently healthy adults (*n* = 23 males; *n* = 26 female) aged 18–65 years consented to take part in the current study, conducted between February and March 2013 to remove the confounding effect of sun exposure on circulating markers of vitamin D status. Dietary intakes of vitamin D were recorded using a 4-day weighed food record (WFR) and the newly developed FFQ. The FFQ was composed of 17 questions to document the habitual frequency of consumption and portion size of foods known to be sources of vitamin D (including natural sources, fortified foods and dietary supplements). Fasting serum 25-hydroxy vitamin D (25(OH)D) concentrations were quantified by liquid chromatography-tandem mass spectrometry (API 4000, AB SCIEX). The validity of the FFQ was established by applying the method of triads to the three intake measurements (FFQ, WFR and biomarker), which estimates the agreement between these three measurements and triangulates an estimate of true dietary intake.

Results: Dietary supplement use was reported by approximately one third of the group (*n* = 18). The mean daily total vitamin D intake reported (food + supplements) using the FFQ was 8.04 µg (range 1.0–36.1 µg), and that from the WFR was 5.64 µg (range 0.4–31.7 µg). Mean daily energy intake was 8486 kJ (range 4962–13807 kJ). Reported vitamin D intakes, however, were similar between the sexes after adjusting for energy intake. Mean serum 25(OH)D concentration of the group was 45.3 nmol/L (range 12.9–279.0 nmol/L). The mean difference between the vitamin D intake quantified from the WFR and FFQ equalled +1.62 (SD 3.86). Cross-classification analysis revealed >90% of the group were classified in the same or adjacent tertiles for vitamin D intakes when comparing results from the FFQ and WFR. Significant correlations were shown between the three intake measurements (FFQ and biomarker: rQB = 0.61; WFR and biomarker: rBR = 0.54; and, FFQ and WFR: rQR = 0.90, all *p* < 0.001). The overall validity coefficient (PQT) of the FFQ calculated using the method of triads was 0.59 (PQT = √rQBx(rQR ÷ rBR), indicating a high validity.

Conclusions: The vitamin D FFQ has now been validated for use in future studies interested in assessing habitual vitamin D intake within the general adult population.

### 2.134. Correlations between Vitamin D Serum Levels, Nailfold Microangiopathy and Clinical Features in Systemic Sclerosis Patients

#### Paolino, S.; Pizzorni, C.; Smith, V.; Decuman, S.; Sulli, A.; Seriolo, B.; Cutolo, M.

Background: Vitamin D (25-hydroxy vitamin D) [25(OH)D_3_] and its active metabolite [1,25(OH)D_3_] are involved in the regulation of both innate and adaptive immunity (Cutolo, M., *et al. Autoimmun. Rev.* 2011, *11*, 84-87). Some immune-mediated diseases, such as rheumatoid arthritis, systemic lupus erythematosus, and undifferentiated connective tissue diseases are characterized by low concentrations of 25(OH)D_3_, often related with disease (Cutolo, M., *et al. Autoimmun. Rev*. 2011, *11*, 84-87). Systemic sclerosis (SSc) is a severe connective tissue disease characterized by vascular damage, immune and fibrotic changes in the the skin and internal organs.The aim of this study was to evaluate the possible correlation between 25(OH)D_3_ serum concentration, nailfold videocapillaroscopic (NVC) markers and clinical manifestations of SSc.

Methods: 117 SSc patients were enrolled, 62 from the Department of Rheumatology, Ghent University Hospital, Belgium and 55 from the Academic Unit of Clinical Rheumatology, University of Genova, Italy. The mean age of patients was 67 ± 12SD years, 83% are female and the mean disease duration calculated from onset of Raynaud phenomenon 13 ± 13 years. All patients were evaluated by nailfold videocapillaroscopy (NVC) to score and classify the severity of the microangiopathy (“early”, “active” and “late” NVC patterns and microangiopathy evolution score [MES] were assessed), as previously reported (Cutolo, M., *et al. J. Rheumatol*. 2000, *27*, 155-160. Sulli, A., et al*. Ann. Rheum. Dis.* 2008, *67*, 885-887). 25(OH)D_3_ serum levels were evaluated by radioimmunoassay: vitamin D concentrations were classified as normal (>30 ng/mL), insufficient (30 < 25(OH)D_3_ < 10 ng/mL) or deficient (<10 ng/mL) (Holick, M.F. *N. Engl. J. Med.* 2007, *357*, 266-281). Clinical features of the disease were assessed using Medsger’s severity scale (score 0–4) (Medsger, T.A., Jr, et al*. Clin. Exp. Rheumatol.* 2003, *21*, S42-S46). Statistical analysis was performed by non parametric tests.

Results: Levels of 25(OH)D_3_ were significantly lower in patients with “late” NVC pattern of microangiopathy in comparison with patients showing either “active” or “early” pattern (17.1 ± 12.4 *vs.* 18.2 ± 13.3 *vs.* 20.2 ± 7.4, *p* < 0.005). A negative correlation was found between 25(OH)D_3_ concentrations and both MES (*r* = −0,49, *p* < 0.003) and peripheral vascular disease according to Medsger scale (*r* = −0.24, *p* < 0.01). There was no significant relationship between serum 25(OH)D_3_ and other clinical features of SSc, including skin, lung, gastrointestinal, renal, heart and joint involvement, assessed using the Medsger’s severity scale, probably because the study was underpowered. Any statistical significant differences between skin subsets of SSc or gender was not found.

Conclusion: Our data revealed that vitamin D serum concentration in SSc are lowest in patients with advanced NVC pattern and severity of peripheral vascular disease using Medsger’s severity scale.

### 2.135. Addition of Serum 25-Hydroxy Vitamin D Concentration to the Deyo-Charlson Comorbidity Index Improves 90-Day Mortality Prediction in Critically Ill Surgical Patients

#### Quraishi, S.A.; Blum, L.; McCarthy, C.M.; Giguere, P.; Young, S.I.; Camargo, C.A., Jr.

Background: Mortality prediction scores, such as the Acute Physiology and Chronic Health Evaluation (APACHE) II, are part of a holistic set of criteria used to set goals of care for intensive care unit (ICU) patients. In a research setting, such scores are used to control for severity of illness in multivariable regression models. APACHE II relies on several physiologic measures within the first 24 h of critical illness, and therefore, is not always readily available to investigators. Mortality prediction scores based on medical history, such as the Deyo-Charlson Comorbidity Index (DCCI), in combination with administrative data is an increasingly popular alternative to APACHE II in the critical care literature. However, the combination of DCCI with biologically relevant biomarkers has not been well explored. Recently, vitamin D status during ICU admission has been shown to be associated with the risk of short-term (30-, 60-, and 90-day) and long-term (1-year) mortality in critically ill patients. As such, our goal was to determine whether the addition of serum 25-hydroxy vitamin D [25(OH)D] concentration to the DCCI improves mortality prediction in patients admitted to the surgical ICU.

Methods: We prospectively measured serum 25(OH)D concentration within 24 h of ICU admission in 200 critically ill surgical patients. Total 25(OH)D concentrations were measured by enzyme-linked immunoabsorbent assay, using commercially available kits (Abbott Laboratories, Abbott Park, IL, USA). APACHE II and DCCI were computed based on data from medical records. A stepwise regression analysis using forward and backward selection was performed to investigate the association of 25(OH)D levels with risk of 90-day mortality, while controlling for multiple biologically plausible covariates including either APACHE II (Model A) or DCCI (Model B). Receiver operating curves (ROCs) were constructed and the area under the curves (AUCs) for DCCI without and with the addition of 25(OH)D were compared to the AUC for 90-day mortality using APACHE II.

Results: Mean (± standard deviation) serum 25(OH)D concentration was 17 ± 6 ng/mL. Mean APACHE II and DCCI were 17 ± 8 and 4 ± 3, respectively. In Model A, both 25(OH)D (Odds ratio [OR] 0.85 per 1 ng/mL rise; 95% Confidence Interval [CI] 0.77–0.95) and APACHE II (OR 1.17 per 1 unit rise; 95% CI 1.08–1.26) were associated with the risk of 90-day mortality. In Model B, both 25(OH)D (OR 0.84; 95% CI 0.76–0.93) and DCCI (OR 1.26; 95% CI 1.05–1.61) were associated with the risk of 90-day mortality. AUC for 90-day mortality using APACHE II was 0.85 (95% CI 0.77–0.94). Addition of 25(OH)D to APACHE II resulted in an AUC of 0.89 (95% CI 0.81–0.96). The AUC for 90-day mortality using DCCI was 0.67 (95% CI 0.54–0.80). Addition of 25(OH)D to DCCI resulted in an AUC of 0.84 (95% CI 0.78–0.93).

Conclusion: In our prospective cohort of 200 critically ill patients in the surgical ICU, APACHE II was found to be a good predictor of 90-day mortality. The addition of 25(OH)D levels to APACHE II resulted in a modest improvement in mortality prediction. On the other hand, DCCI alone was found to be a poor predictor of 90-day mortality; however, the addition of 25(OH)D resulted in a clear improvement in ability to predict mortality, with an AUC that was comparable to APACHE II alone.

### 2.136. Investigating Polymorphisms in the NADSYN1/DHCR7 Locus (rs1790349 and rs12785878) as Novel Genetic Markers for Cardiovascular Disease

#### Abu El Maaty, M.A.; Hassanein, S.I.; Gad, M.Z.

Background: Recent Genome-Wide Association Studies (GWAS) have identified the rs1790349 and rs12785878 single nucleotide polymorphisms (SNPs), present in the NADSYN1/DHCR7 locus, as influencing circulating 25-Hydroxy vitamin D [25(OH)D] levels, which itself has been linked to various diseases including cardiovascular disease (CVD). This study aimed to investigate the association of these SNPs with: (i) CVD and (ii) 25(OH)D levels.

Methods: Sixty-three male patients with verified coronary artery disease (CAD) were recruited, as well as thirty-one age- and sex-matched controls. Genotyping was performed by sequencing and 25(OH)D levels were assessed by high performance liquid chromatography with ultra-violet detection.

Results: Statistical significance was observed in comparing the genotypic distribution of patients and controls for the rs12785878 polymorphism (*p* = 0.003) but not the rs1790349 (*p* = 0.9). Allelic distributions of rs1790349 and rs12785878 yielded insignificant results (*p* = 0.7; OR: 0.58 to 2.6 and *p* = 0.14; OR: 0.88 to 2.85, respectively). Taking together patients and controls, both SNPs were found to influence total 25(OH)D levels (*p* = 0.001 and <0.0001) as well as 25(OH)D_3_ levels only in controls.

Conclusion: This study presents the rs12785878 polymorphism as a novel genetic marker for CVD. It also adds to accumulating evidence suggesting the ability of the investigated SNPs to predict circulating 25(OH)D levels.

### 2.137. Vitamin D Sufficiency Slows the Progression of Premalignant Dysplasic Lesions in the NTCU Mouse Model of Lung Squamous Cell Carcinoma

#### Mazzilli, S.A.; Bogner, P.N.; Attwood, K.; Hershberger, P.A.; Trump, D.L.; Johnson, C.S.

Background: Progress has recently been made in identifying populations at risk for lung cancer using genetic, clinical and demographic information. The N-nitroso-tris-chloroethylurea (NTCU) mouse model, in which animals develop premalignant histopathology similar to that seen in humans, can be used to examine the potential efficacy of chemoprevention agents to be utilized in at risk populations. We identified 25 µL of 40 mM NTCU once per week as the optimal dosing regimen in SWR/J mice for chemoprevention studies. At this dose, topical treatments with NTCU induce predominantly low-grade dysplastic lesions in the large airways by 15 weeks (w) and high-grade dysplastic (HGD) lesions in the large airways by 25 w. Additionally we found that NTCU stimulates a state of chronic inflammation associated with the development of dysplasias. Epidemiologic studies indicate that there is an inverse relationship between vitamin D and the risk and prognosis of lung cancer. Vitamin D acts through the vitamin D receptor to promote cellular differentiation and inhibit proliferation and inflammation.

Methods: The effect of vitamin D on cancer progression was tested in the NTCU model, using dietary vitamin D_3_ (0 or 2000 IU/kg) alone and in combination with intraperitoneal injections of the active metabolite of vitamin D, calcitriol (80 µg/kg). Female mice were randomized to 6 treatment groups (*n* = 15 mice/group/time point (15 and 25 weeks)). Disease was evaluated by enumerating the percentage of HGD lesions, per the total area in serial H&E sections of the lung. Proliferation was measured using Ki-67 staining and 500 cells/sample (*n* = 5/group). Additionally, local and systemic inflammation was quantified through manual differentials and multi-plex and qRT-PCR were used to measure cytokine levels and expression.

Results: The percentage of HGD lesions in the large airways was reduced in the vitamin D sufficient mice (SN) (8.72%, *p* < 0.05), SN + calcitriol (SN + C) (6.59%, *p* < 0.05) and the vitamin D deficient + calcitriol (DN + C) (12.3%, *p* < 0.05) groups compared to the DN (22.67%) group after 15 w of NTCU. The percentage of HGD lesions in the SN groups remained significantly less than the DN (35.5%) and DN + C (21.3%) groups, with little increase in the SN (8.43%, *p* < 0.05) and SN + C (11.81% not significant (NS)) groups after 25 w. Furthermore, there was a significant increase in Ki-67 staining associated with increased HGD. Following 25 w of NTCU treatment there was a 2-fold increase in positive staining all groups compared all groups after 15 w of NTCU. However, the was 30% more staining in the DN (*p* < 0.001) group and addition of calcitriol reduced proliferation by 12% and 6% in the DN + C (*p* < 0.01) and SN + C (NS) groups. Moreover, vitamin D deficiency was associated with an increased systemic and local inflammation marked by a 3-fold increase in circulating white blood cells (WBCs) (*p* < 0.05), a 20% increase in IL-6 levels (*p* < 0.05) and a 4 -fold increase in WBCs in bronchial lavages (*p* < 0.05) in the DN group.

Conclusions: In conclusion this study indicates that vitamin D deficiency promotes the development of dysplasia, increases proliferation and inflammation, which is likely promote the development of frank squamous cell carcinoma, whereas sufficiency retards this development.

Supported by: NIH/NCCAM F31AT0006487 (Mazzilli, SA, USA) NIH/NCI CA067267 (Johnson, CS, USA).

### 2.138. Sunlight Exposure and Vitamin D Status of City Dwellers with Active Tuberculosis Compared with Contacts: Is the Relationship Clear?

#### Steel, S.C.; Martineau, A.R.; Mandami, M.; McDaid, M.; Johnson, N.; Malone-Lee, J.

Background: Adequate vitamin D status is an important modulator of immunity against Mycobacterium TB. Exposure to sunlight is probably the most significant source of Vitamin D yet no studies have directly measured sunlight exposure in TB patients. This study sought to establish the relationship between Vitamin D status and sunlight exposure using personal UVR dosimetry in an urban setting. A second study measured total potential UVR as urban variables potentially reduce exposure to UVR. Mechanisms cited include scattering and absorption elicited by low level ozone and pollution as well as shadowing from buildings and trees. UVR (UVB) levels were measured and compared with UVR data from a rural setting nearby, to assess any significant difference in potential exposures due to urban variables.

Methods: Subjects were invited to take part from a central London TB clinic. 12 index cases and 13 contacts completed the study. Both groups were tested for IGRA reactivity to determine their TB status. Dermal Melanin content was measured using light spectrometry. UVR exposure was measured using Polysulphone film over 8 weeks. Film measurements were translated into a standard erythemal dose (SED) or where subjects failed to return the films, these were estimated. Vitamin D levels were measured before and after the sunlight exposure period as was melanin content of the skin. Additionally over a period of 1 year polysuphone film badges were placed on top of a wood construction that was fixed to a roof above shadowing from other structures. The badges were changed daily or every other day in the winter months. The films were read pre and post exposure, in a spectrophotometer at 330 nm and the change in absorbance calculated to reflect the potential daily UVB measurement and expressed as a standard erythemal dose (SED). These data were compared with previous measurements taken at a rural setting nearby.

Results: Mean values for 25(OH) D in index cases were 31.6 nmols/L and 36.1 nmols/L in contacts cases, at the end of the study period. Sun exposure was not significantly related to 25(OH) D in index cases(*r* = 0.016, sig = 0.961, *p* = 0.05) and showed a very weak positive correlation in contacts (*r* = 0.233, sig = 0.44, *p* = 0.05). There were no significant changes in facultative melanin that could be indicative of tanning after the UVR monitoring period. There were no significant differences between the two UVR data sets regardless of differing measurement methods and years of measurement. City and rural data showed a strong correlation: *r* = 0.958, *n* = 12, (months) *p* = 0.01. Whilst London showed slightly higher monthly means. These results suggest that UVR exposure in the city is comparable to more suburban environments.

Conclusion: Both index and contact groups received exposures that should have predicted Vitamin D sufficiency yet both groups displayed Vitamin D deficiency and insufficiency. A seasonal correlation was not evident in the index group raising questions about the relationship between sunlight exposure and Vitamin D status in those with Tuberculosis. Additionally, city life may inhibit Vitamin D sufficiency in those without Tuberculosis due to living styles that prevent adequate UVR exposure, which may also be overestimated. Further study of the relationship between UVR exposure and Vitamin D status using personal sunlight measurements is required in both TB patients and the general population to determine the contribution of sunlight to Vitamin D status.

### 2.139. The Addition of Cholecalciferol in the Management of Secondary Hyperparathyroidism; A Haemodialysis Case Report

#### Parker, S.A.; Ting, S.M.; Fletcher, S.; Zehnder, D.; Bland, R.

Background: Secondary hyperparathyroidism (SHPT), a complication of Chronic Kidney Disease (CKD) is associated with increased fracture and cardiovascular risk. Routine treatment includes phosphate restriction ± binding agents and vitamin D receptor activators (VDRa) such as calcitriol [1,25(OH)D], alfacalcidol or paricalcitol. Those refractory to standard treatment are treated with calcimimetics (allosteric activators of the calcium sensing receptor) or if appropriate parathyroidectomy. Management with VDRa and calcimimetics is often complicated by hyperphosphataemia and hypercalcaemia. We report a case of a 43 year female of African-Caribbean origin with severe SHPT in the presence of hungry bone syndrome due to chronically depleted bone mineral stores. Having end stage renal disease (ESRD) since 2002 she was initially managed on peritoneal dialysis and transferred to haemodialysis in 2004. The patient refused parathyroidectomy. Calcimimetic treatment was titrated up to maximum dose and used alongside high dose VDRa, high calcium dialysate and high dose phosphate supplementation. This resulted in some improvement in serum iPTH (intact PTH) but not to within, or near, target (8–38 pmol/L). At this point the patient’s vitamin D status was assessed as insufficient and vitamin D_3_ supplementation commenced.

Methods: In April 2011 the patient’s serum 25(OH)D was measured as 65 nmol/L (optimal is ≥75 nmol/L). At this time the patient had a serum iPTH of 222 pmol/L, alkaline phosphatase (ALP) 4075 U/L (target 35–105 U/L), adjusted calcium of 2.28 nmol/L (target 2.10–2.58 nmol/L) and phosphate of 0.65 nmol/L (target 1.1–1.7 nmol/L). The patient was being managed with; calcitriol 6mcg/day, phosphate sandoz 2 tablets thrice daily, calcium sandoz 2 tablets thrice daily, and the calcimimetic cinacalcet 180 mg/day. Vitamin D_3_ 20,000 IU/week was added and bone and iPTH levels were monitored monthly. We report 3 time points; time zero (T0), 6 months (T6) and 15 months (T15). ALP and iPTH were measured using Roche modular assays.

Results: At T0 despite being on a maximal dose of cinacalcet and a high VDRa dose the patient’s iPTH remained high (222 pmol/L). However, addition of vitamin D_3_ resulted in a reduction of both iPTH and ALP levels. After 6 months (T6) of vitamin D_3_ supplementation serum iPTH had reduced by 29% to 158 pmol/L and ALP by 26% to 3008 IU/L. Adjusted calcium and phosphate levels remained stable at 2.35 mmol/L and 0.65 mmol/L respectively. At T15 serum 25(OH)D was 103 nmol/L (37% increase from T0). There had been a continued decrease in iPTH and ALP levels. Serum iPTH had reduced further to 63 pmol/L (72% reduction from T0) and ALP had decreased to 1955 IU/L (52% reduction from T0). Adjusted calcium had risen slightly (2.55 mmol/L; 8.5% increase from T0), and phosphate was reduced (0.26 mmol/L; 60% reduction from T0). The decrease in serum phosphate may be attributed to documented concordance problems with phosphate supplementation rather than a consequence of improved serum 25(OH)D.

Conclusions: Management of renal bone disease in ESRD has traditionally focused on treatment with VDRa. Improving serum 25(OH)D levels to >75 nmol/L may offer further benefit through promoting synthesis of local 1,25(OH)D in the parathyroid tissues, particularly in the presence of cinacalcet resistance. Assessment and correction of vitamin D status should be considered in addition to the current routine treatment of secondary hyperparathyroidism.

### 2.140. Vitamin D Deficiency in Advanced Chronic Kidney Disease Is not Corrected after Kidney Transplantation

#### Parker, S.; Ting, S.M.; Petchey, M.; Higgins, R.; Fletcher, S.; Zehnder, D.; Bland, R.

Background: Supplementation with calcitriol (1,25-dihydroxy vitamin D; or analogue) is used routinely in patients with chronic kidney disease (CKD). However this form of supplementation overlooks 25-hydroxy vitamin D [25(OH)D] deficiency, which is rarely screened for or treated. Vitamin D deficiency correlates with falling estimated glomerular filtration rate (eGFR) and is seen in up to 95% of people with end stage renal disease. This may be due to; reduced sunlight exposure, uraemia effecting liver hydroxylation, poor diet or higher activity of 24-hydroxylase. Successful kidney transplantation may therefore improve circulating 25(OH)D levels.

Methods: We assessed serum 25(OH)D in renal transplant patients within 4 weeks prior to kidney transplantation in parallel to CKD patients (CKD5) and a group of healthy medication-controlled hypertensive subjects at time zero (T0) and 1 year later (T1). 25(OH)D was measured using the Elecsys Vitamin D Total Assay (Roche). Statistical analysis was performed using Wilcoxon matched pairs, Kruskal-Wallis and Spearman correlation (SPSS). Data represent mean ± SEM.

Results: In total 99 patients were assessed. 32 who received a kidney transplant (mean age 45.2 ± 2.59 years; male 59%; BMI 24.7 ± 0.62 kg/m^2^), 33 patients with CKD (mean age 47.6 ± 2.34 years; male 73%; BMI 27.4 ± 1.00 kg/m^2^) and 34 in the hypertensive group (mean age 55.2 ± 1.24 years; male 44%; BMI 28.0 ± 0.58 kg/m^2^). At T0 a significant number of patients in each group had low serum 25(OH)D levels. As expected there was no significant difference in the levels between the CKD group and the patients just prior to transplant (34.6 ± 2.7 nmol/L *vs.* 35.6 ± 3.1 nmol/L respectively). 100% of patients had levels <75 nmol/L and 38.5% of patients were deficient (<30 nmol/L). In contrast the basal 25(OH)D level was significantly greater in the hypertensive group (62.4 ± 5.2 nmol/L; *p* < 0.001) with 32.4% having optimal levels (≥75 nmol/L) and only 17.6% were deficient. Levels were measured again one year later (T1). In the hypertensive patients serum 25(OH)D had not altered significantly (T1, 66.0 ± 5.0 nmol/L *vs.* T0, 62.4 ± 5.2 nmol/L). However, it was apparent that there were now differences between the CKD and transplanted patients. 25(OH)D levels in the CKD group had decreased still further (27.3 ± 3.2 nmol/L; *p* < 0.05) and were now significantly lower than the transplant patients (*p* < 0.05) and significantly more were deficient (66.7%; *p* < 0.05). In contrast, 25(OH)D levels in the patients who had received a transplant showed a small non-significant increase to 45.5 ± 4.6 nmol/L. However, this was still significantly lower than the hypertensive group (*p* = 0.004). Although levels increased in 47% of patients, only 15.6% achieved levels ≥75 nmol/L and 28.1% remained deficient. Post-transplant the 25(OH)D levels correlated with PTH (*p* < 0.05), but not eGFR. At T0, 56% of this group of patients were receiving alphacalcidol/calcitriol, and this reduced to 15.6% post-transplant. Although not positively correlated, it was interesting to note that of the 15 patients whose 25(OH)D levels increased only two remained on alphacalcidol/calcitriol.

Conclusions: All patients with CKD had low levels of serum 25(OH)D. In 47% of patients levels increased post-transplantation, but did not achieve those seen in the hypertensive group. Therefore, although kidney transplant may improve 25(OH)D levels in some patients they still remain insufficient and supplementation should be considered.

### 2.141. The C3-Epimer of 25-Hydroxy Vitamin D_3_ [3-epi-25OHD_3_)] Is Quantifiable in Almost All Neonates and Mirrors Variation in Serum 25OHD3

#### O’Donovan, S.M.; Zhang, Y.; Kinsella, M.; Murray, D.M.; Kenny, L.C.; Hourihane, J.O’B.; Kiely, M.

Background: There are no reference data for 25-hydroxy vitamin D [25(OH)D] concentrations in umbilical cord sera. Our aim was to quantify the determinants of cord serum 25(OH)D and propose reference intervals for 25OHD_3_, 25OHD_2_ and the 3-epi-25OHD_3_ in the Cork BASELINE Birth Cohort Study, which is a large, well-characterised birth cohort at Northerly latitude (52oN).

Methods: Umbilical cord blood was processed to serum within 3hrs of collection at delivery and stored at −80 °C. Serum 25(OH)D was quantified in 1050 maternal-infant dyads using liquid chromatography-tandem mass spectrometry (LC-MS/MS), using a method which is traceable to the NIST higher order reference measurement procedure (Tai, S.S., et al. *Anal. Chem.* 2010, *82*, 1942-1948. Sempos, C.T., et al*. Scand. J. Clin. Lab. Invest. Suppl*. 2010, *243*, 32-40). Due to the absence of reference thresholds for cord serum 25(OH)D, current IOM cut-offs for 25(OH)D in the population were used which specify deficiency <30 nmol/L and suggest that 97.5% of the population requirements would be met at ≥50 nmol/L (Institute of Medicine (2011). Washington, DC: The National Academies Press).

Results: Total serum 25(OH)D [sum of 25(OH)D_3_ plus 25(OH)D_2_] concentrations ranged from 4.7–111.3 nmol/L; the mean ± SD was 34.9 ± 18.1 nmol/L. The prevalence of 25(OH)D < 30 was 46% (62% in winter) and 80% were <50 nmol/L (89% in winter). Among the 42% of women who took a vitamin D-containing supplement at 15 weeks gestation, 31 and 72% of the cords were <30 and 50 nmol/L, respectively. 59 and 91% of infants born to women with serum 25(OH)D levels <50 nmol/L at 15 weeks gestation were <30 and 50 nmol/L, respectively. The main determinants of cord 25(OH)D [adjusted mean difference in nmol/L (95% CI)] were summer season of sampling [19.2 (17.4, 20.9), p < 0.0001], maternal 25(OH)D concentrations at 15 weeks [0.3 (0.26, 0.34), p < 0.0001] and the maternal use of a vitamin D containing supplement at 15 weeks [2.5 (0.6, 4.42), p = 0.011]. Smoking at 15 weeks gestation was a negative predictor of cord 25(OH)D [−4.8 (−7.8, −1.8), *p* = 0.002]. Serum 25(OH)D_2_ was detected in 98% of infants and the median concentration was 1.9 nmol/L, ranging from 0 to 38.9 nmol/L. The 3-epi-25(OH)D_3_ was detected in nearly all neonates (99.4%) with a range of 0 to 11.9 nmol/L; [median 2.9 nmol/L]. The median molar ratio of 25(OH)D_3_ to 3-epi-25(OH)D_3_ was 10.1 [interquartile range 8.8, 11.3], twice the molar ratio reported during pregnancy (Zhang, J.Y., *et al*. *Am. J. Clin. Nutr*. Under review). Concentrations of 3-epi-25(OH)D_3_ tracked 25(OH)D_3_ on a month-by-month basis (*r* = 0.880, *p* < 0.001).

Conclusions: We present the first large reference dataset of 25(OH)D in umbilical cord serum. The prevalence of very low 25(OH)D concentrations was high. These data show that the 3-epi-25(OH)D_3_ in infants at delivery tracks 25(OH)D_3_ and is present at twice the molar ratio than that evident in maternal serum. Should the C3-epimer be proven to have biological significance, this would have an impact on the quantification of 25(OH)D concentrations in cord sera.

### 2.142. No Improvement in Glucose Metabolism and Cardiovascular Risk Factors after 1 Year of Supplementation of High-Dose Vitamin D. 1 Year Results from a Double Blinded Randomized Clinical Trial in Subjects with Prediabetes

#### Sollid, S.T.; Hutchinson, M.S.; Fuskevåg, O.M.; Figenschau, Y.; Kamycheva, E.; Svartberg, J.; Jorde, R.

Background: Low serum-25-hydroxy vitamin D (25(OH)D) have been associated with increased plasma glucose and increased insulin resistance in several observational studies. Risk factors for cardiovascular disease have also been associated with low serum (25(OH)D. We aimed to investigate whether high-dose vitamin D supplementation can improve glucose metabolism in a population with impaired fasting glucose and/or impaired glucose tolerance.

Methods: 556 individuals with impaired fasting glucose and/or impaired glucose tolerance were randomly assigned to 20,000 IU of vitamin D_3_/week (*n* = 278) or placebo (*n* = 278). Oral glucose tolerance tests (OGTT) have been performed annually with the main outcome after 1 year being changes in glucose tolerance.

Results: Vitamin D supplementation increased serum 25(OH)D levels compared to placebo (45.8 ± 24.2 nmol/L compared with 3.4 ± 11.9 nmol/L; *p* < 0.000). There were no differences between the groups regarding fasting blood glucose, 2 h blood glucose after OGGT, HbA1c levels, insulin secretion and sensitivity and development of type 2 diabetes after 1 year. There were no differences between the groups in blood pressure or lipid concentration.

Conclusions: High dose vitamin D supplementations do not improve the glycaemic indices or the cardiovascular risk factors in a population with prediabetes.

### 2.143. Prenatal Vitamin D Supplementation and Child Respiratory Health: A Randomised Controlled Trial

#### Goldring, S.T.; Griffiths, C.J.; Martineau, A.R.; Robinson, S.; Yu, C.; Poulton, S.; Kirkby, J.C.; Stocks, J.; Hooper, R.; Shaheen, S.O.; Warner, J.O.; Boyle, R.J.

Background: Observational studies suggest high prenatal vitamin D intake may be associated with reduced childhood wheezing. We examined the effect of prenatal vitamin D on childhood wheezing in an interventional study.

Methods: We randomised 180 pregnant women at 27 weeks gestation to either no vitamin D, 800 IU ergocalciferol daily until delivery or single oral bolus of 200,000 IU cholecalciferol, in an ethnically stratified, randomised controlled trial. Supplementation improved but did not optimise vitamin D status. Researchers blind to allocation assessed offspring at 3 years. Primary outcome was any history of wheeze assessed by validated questionnaire. Secondary outcomes included atopy, respiratory infection, impulse oscillometry and exhaled nitric oxide. Primary analyses used logistic and linear regression.

Results: We evaluated 158 of 180 (88%) offspring at age 3 years for the primary outcome. Atopy was assessed by skin test for 95 children (53%), serum IgE for 86 (48%), exhaled nitric oxide for 62 (34%) and impulse oscillometry of acceptable quality for 51 (28%). We found no difference between supplemented and control groups in risk of wheeze (no vitamin D: 14/50 (28%); any vitamin D: 26/108 (24%) (risk ratio 0.86; 95% confidence interval 0.49, 1.50; *p* = 0.69)). There was no significant difference in atopy, eczema risk, lung function or exhaled nitric oxide between supplemented groups and controls.

Conclusion: Prenatal vitamin D supplementation in late pregnancy that had a modest effect on cord blood vitamin D level, was not associated with decreased wheezing in offspring at age three years.

Registered with the International Standard Randomised Controlled Trial Number Register, ISRCTN 68645785.

### 2.144. Vitamin D Levels and TMEM16J Host Variants are Associated with Tuberculosis and Death in HIV-Infected and -Exposed Infants

#### Spector, S.A.; Jubulis, J.; Zeldow, B.; Montepiedra, G.; Detrick, B.; Violari, A.; Madhi, S.A.; Mitchell, C.; Gupta, A.

Background: In developing countries, the HIV/AIDS epidemic has been accompanied with an increased risk for tuberculosis (TB) in young children. Although vitamin D has been associated with the development of TB in adults, little research has examined the importance of vitamin D levels in children. The current study was designed to identify the effect of vitamin D insufficiency and deficiency (VDID) and host genetic single nucleotide polymorphisms (SNPs) that alter Vitamin D receptor expression on the risk for TB in HIV-infected and –exposed infants.

Methods: A case-cohort study was performed (total *n* = 346 with a random subcohort of 270) in IMPAACT P1041, a trial performed in South Africa that evaluated the efficacy of isoniazid (INH) prophylaxis on reducing TB disease in 1351 HIV-infected and -exposed, uninfected infants. In the original trial, INH prophylaxis was found to be of no benefit in preventing TB. Vitamin D levels were determined by a chemiluminescent immunoassay. Five Vitamin D related SNPs (Bsm-1-G/A [rs1544410], Fok-I-C/T [rs2228570], GC-A/C [rs2282679], DHRC7/NADSYH1–G/T [rs12785878] and CYP2R1-G/A [rs10741657]), and three SNPs in the PKP3-SIGIRR-TMEM16J region recently found to be associated with TB susceptibility (PKP3 (rs10902158-A/G), PKP3 (rs7105848-T/C), and TMEM16J (rs7111432-G/A)) were assessed by real-time PCR. The primary outcome for the study was time to probable/definite TB by 192 weeks. Secondary outcomes included time to possible, probable, or definite TB, time to latent TB/TB disease, and time to TB or death. Prevalence of VDID, defined as <32 ng/mL, and host SNPs were determined using the random subcohort. To determine the associations between VDID and host SNPs with outcomes, Cox regression was performed.

Results: The median age when Vitamin D levels were determined was 8 months; 11% of subjects were ever breastfed; 51% were HIV-infected. VDID prevalence was 26%. There were 138 TB cases (43 definite/probable, and 95 possible) and 26 deaths included in this analysis. Children with VDID had a 77% greater risk of probable/definite TB (HR 1.77, *p* = 0.17). When possible TB was included in the outcome, VDID was associated with a significant increase in TB (HR 1.99, *p* = 0.005). VDID was also associated with any TB or death (HR 2.05, *p* = 0.002). Adjusting for HIV status, season, site, sex, weight-for age z score, breastfeeding, type of house, mother with previous TB diagnosis, age at vitamin D determination, and INH/placebo treatment arm, VDID was independently associated with any TB (aHR 1.75, 95% CI 1.01–3.05; *p* = 0.046) as well as any TB or death (aHR 1.76, 95% CI 1.03–3.00; *p* = 0.038). No polymorphism was associated with VDID. In both crude and adjusted models, however, having the TMEM16J G-allele was protective of probable/definite TB (aHR 0.53, *p* = 0.07), any TB (aHR 0.56, *p* = 0.017), any TB/death (aHR 0.59, *p* = 0.016) and any TB/latent TB (aHR 0.58, *p* = 0.009); having GG polymorphism of the PKP3 genetic variant was marginally associated with increased risk of any TB/death (aHR 1.44, *p* = 0.07).

Conclusions: In young HIV-infected or –exposed, uninfected infants, VDID was associated with nearly a 2-fold increased risk of TB or death. Additionally, specific SNPs that affect innate immunity altered the risk for TB. These findings suggest that Vitamin D supplementation of mothers and/or infants born to HIV-infected women may be useful in decreasing infant TB or death.

### 2.145. Vitamin D Status and Type I Hypersensitivity in Childhood

#### Jones, S.L.

Background: Recent studies have suggested possible links between vitamin D deficiency and type I hypersensitivity and atopic disease. However, data have been inconsistent and a causal relationship is yet to be confirmed. The objective of this retrospective case-control study was to further investigate the possible link between type I hypersensitivity and vitamin D status in an inner-city population known to have a high prevalence of vitamin D deficiency.

Methods: Total 25-Hydroxy vitamin D levels were assessed in 850 children (<16 years) undergoing routine assessment in primary and secondary care for clinically suspected allergy. Specific IgE’s were selected by requesting clinicians during routine practice based on clinical presentation of the participants. The effect of vitamin D deficiency on risk of sensitisation to various common allergens was assessed using multiple logistic regression analysis. The immunoassay methods used for 25-Hydroxy vitamin D (Siemen’s Centaur XP) and specific/total IgE (Siemen’s Immulite 2000 3gAllergy™) were fully evaluated prior to use in the study to verify analytical performance.

Results: Vitamin D deficiency was strikingly common with 37.1% of all participants showing 25-OHD < 25 nmol/L. Vitamin D deficiency was significantly associated with risk of sensitisation to any one allergen (OR 1.64 [95% CI 1.21–2.23]) but this could be entirely explained by the effect of confounding factors (OR after adjustment 0.95 [95% CI 0.68–1.34]). A similar result was obtained when data were analysed for association with sensitisation to individual allergens and total IgE levels.

Conclusions: These findings contrast with other recent studies which showed clear associations between IgE levels and vitamin D status. The very high prevalence of vitamin D deficiency in this population and subsequent small number of vitamin D replete participants may have reduced the power of this study to detect a statistically significant association between vitamin D deficiency and hypersensitivity.

### 2.146. A Systematic Review of the Impact on Vitamin D Status with Moderate Levels of Vitamin D Intakes (5 mcg–20 mcg)

#### Whiting, S.J.; Payen, F.; Rousseau, B.

Background: There is controversy surrounding the designation of vitamin D adequacy as defined by circulating levels of the metabolite 25-hydroxy vitamin D [25(OH)D]. Depending on the cutoff chosen, dietary intakes of vitamin D may or may not provide sufficient impact upon vitamin D status, in improving levels of 25(OH)D. Purpose: We sought to examine whether a supplemental dose of 10 mcg (400 IU) as found in fortified foods or as a supplement, has a measurable impact on vitamin D status, as defined by improving status from below to above 50 nmol/L, or from less than 30 nmol/L to above 30 nmol/L.

Methods: Published literature was searched for relevant articles of 10 mcg (400 IU) vitamin D supplementation or fortification. Exclusion criteria were: nonhuman studies (cell, animal); review articles; studies lacking blood level data pre- and post-treatment; no control group; bolus treatments (weekly, monthly, yearly); vitamin D < 5 mcg (200 IU) or >20 mcg (800 IU); baseline 25(OH)D > 75 nmol/L; patient studies (e.g., diabetes, cancer, CVD); studies < 2 months; age < 19 years. Of the 123 studies retrieved, 24 were initially selected: 7 food studies with doses 3.3 to 20 mcg and 17 publications on supplements 5 to 20 mcg. Of these 7 studies provided 10 mcg (400 IU) meeting all criteria.

Results: Studies involving addition of 10 mcg (400 IU) as fortified foods or supplements gave similar effects on 25(OH)D, so data were combined. After ≥2 mo intervention, mean 25(OH)D status rose either from “insufficient” (25–50 nmol/L) to “sufficient” (>50 nmol/L); or from “deficient” (<25 nmol/L) to “insufficient” (>25 but <50 nmol/L). These increases would not have been predicted using the rule of thumb of 1 mcg raises 25(OH)D by 1 nmol/L.

Conclusions: Our data suggest that an additional intake of 10 mcg (400 IU) can raise average 25(OH)D out of the insufficiency or deficiency range. This suggests fortification with moderate amounts of vitamin D may have positive effects on bone health of populations.

### 2.147. Serum Vitamin D Concentrations Are Associated with Markers of Intestinal but not Systemic Inflammation in Crohn’s Disease in Remission

#### Raftery, T.; Smith, S.; O’Morain, C.; Mahmud, N.; Healy, M.; Cox, G.; McNamara, D.; O’Sullivan, M.

Background: There are established associations between vitamin D (vitD) status and Crohn’s disease (CD). In addition to effects on bone, vitD also has immunomodulatory and anti-inflammatory roles (Raftery, T., et al. *Curr. Drug Metab*. 2012, *13*, 1294-1302). Emerging evidence also suggests a role in maintaining remission in CD. Previously we reported vitD supplementation may be associated with maintenance of intestinal barrier integrity in CD (Raftery, T., et al*. Proc. Nutr. Soc*. 2013, *72*, E1725(OH)D6). Direct measures of intestinal inflammation are typically invasive, while disease scores and systemic markers may not adequately reflect intestinal inflammation in CD. The aim of the current study was to investigate the association between 25(OH)D (nmol/L) and a non-invasive stool marker of intestinal inflammation (fecal calprotectin).

Methods: 85 patients with CD were recruited from February 2012–February 2013. Detailed clinical data was recorded; serum 25(OH)D was analysed by liquid chromatography-tandem mass spectrometry in a DEQAS affiliated laboratory. Fecal calprotectin (Epitope Diagnostics), disease activity [Crohn’s disease activity index (CDAI)] and C-Reactive Protein (CRP) were assessed. A CDAI score >150 was indicative of active disease. Calprotectin (µg/g) concentration of >43.2 µg/g was indicative of intestinal inflammation. VitD deficiency was defined as 25(OH)D < 50 nmol/L, insufficiency as 50–74 nmol/L and sufficient as ≥75 nmol/L.

Results: 39 males and 46 females of mean (sd) age 46.3 (11.8) years participated. The median (IQR) vitD conc. was 57.6 (47.0–84.6 nmol/L). 27.1% (*n* = 23) were vitD deficient; 38.8% (*n* = 33) were insufficient and 34.1% (*n* = 29) were sufficient. The group were in disease remission as indicated by CDAI [72 (45.3–117.5)] and CRP values [1.48(1.0–3.4) mg/L]. Median calprotectin values were 41.1(19.9–152.3) µg/g. Fecal calprotectin, as a marker of intestinal inflammation, was significantly lower in CD patients who were vitD sufficient (>75 nmol/L) compared to those vitD insufficient (<75 nmol/L) [39.0(18.0–103.0) vs. 43.1(21.9–211.2) µg/g, (p = 0.04)]. Consistent with this finding, 25(OH)D concs. significantly inversely correlated with fecal calprotectin levels (*r* = −0.22, *p* = 0.04). There was, however, no significant difference observed in CRP [1.2 (1.0–2.83) vs. 1.68 (1.0–3.68), (p = 0.449) or CDAI [96.5 (43.0–147.3) vs. 67.0 (43.0–104), p = 0.192] according to vitD status (>/<75 nmol/L), or based on associations with CRP (rho = −0.096, *p* = 0.434) or CDAI (rho = 0.194, *p* = 0.108).

Conclusion: This study demonstrates that higher 25(OH)D concentrations were associated with lower intestinal inflammation, as measured by fecal calprotectin, in CD patients in remission; this effect not observed with disease scores or CRP. Intervention studies are required to more fully explore the effects on vitamin D on intestinal inflammation in CD.

### 2.148. Vitamin D Status of Children in Rural and Urban Ethiopia: Predictors for Deficiency

#### Wakayo, T.; Belachew, T.; Vatanparast, V.; Whiting, S.J.

Background: There are few studies that look at vitamin D status in children living in sunny climates as it is assumed that they receive adequate vitamin D from sun exposure. Purpose: To determine vitamin D status and its predictors among school children aged 11–18 years in Ethiopia.

Method: A school-based comparative cross-sectional study was conducted in Adama Town (urban, *n* = 89) and Adama Woreda (rural, *n* = 85) for a total sample of 174 during May–June 2013. Children were randomly selected using multi-stage stratified sampling method. Socioeconomic, demographic, and sun exposure data were obtained; anthropometry measured; and capillary blood (finger prick) to determine serum 25(OH)D levels.

Results: Vitamin D deficiency (serum 25(OH)D < 50 nmol/L) was found in 42% of children. The proportion of deficiency was significantly higher among urban students as compared to those in rural setting (61.8% *vs.* 21.2%, respectively; *p* < 0.001). The significant predictors of lower vitamin D status identified using multivariable logistic regression model were: urban setting, female sex, high maternal education, greater triceps skinfold thickness, less sun exposure, less body surface area exposed, having television/computer in the home, and high socioeconomic status [AOR(2.74–19.57): 95% CI (1.23, 69.21)].

Conclusion: Vitamin D deficiency was prevalent in school children living close to the equator in Ethiopia, both in urban and rural settings, with the prevalence being significantly higher among urban school children. Knowing that modernization is bringing about a change in vitamin D can inform policy-makers in Ethiopia and other tropical countries about the need to implement public health measures to prevent escalation of vitamin D deficiency and its associated health outcomes.

### 2.149. Serum 25-Hydroxy Vitamin D Concentrations and Its Determinants in the Very old: The Newcastle 85+ Study

#### Hill, T.R.; Kirkwood, T.; Granic, A.; Davies, K.; Collerton, J.; Martin-Ruiz, C.; Mathers, J.C.; Adamson, A.J.; Francis, R.M.; Pearce, S.; Rasvi, S.; Jagger, C.

Background: Despite recent concerns about the high prevalence of low vitamin D status in much of the British adult and pediatric population, there is a dearth of data on vitamin D status and its predictors in very old adults, a sub-group of the population which is at increased risk of poor vitamin D status. The objective of the present study was to investigate the prevalence and predictors of vitamin D deficiency throughout the year among a large representative sample of 85 year-old men and women living in the North East of England (55°N).

Methods: Serum concentrations of 25-hydroxy vitamin D [25(OH)D] were analysed in 775 participants in the baseline phase of the Newcastle 85+ cohort study. Season of blood sampling, health, lifestyle, socio-economic and anthropometric data were collected and included as potential predictors of vitamin D status in regression models.

Results: Median serum 25(OH)D concentrations were 27, 45, 43 and 33 nmol/L during Spring, Summer, Autumn and Winter, respectively. The prevalence of vitamin D deficiency (serum 25(OH)D < 30 nmol/L) varied significantly with season with 51% and 23% of participants deficient during spring and autumn respectively (*p* < 0.001). Use of vitamin D supplements, living in an institution and season of blood sampling were significant independent predictors of 25(OH)D concentrations using linear regression models, while non-use of vitamin D containing supplements, season and low physical activity predicted vitamin D deficiency using logistic regression models.

Conclusion: There is an alarming high prevalence of vitamin D deficiency in 85 year-olds living in North East England at all times of the year but particularly during winter and spring. Future work will explore the relationship between 25(OH)D and dietary intake as well as the association between 25(OH)D and health outcomes.

### 2.150. Vitamin D Deficiency and Severity of Acute Respiratory Infection among New Zealand Children—A Case-Control Study

#### Ingham, T.R.; Jones, B.; Camargo, C.A., Jr.; Kirman, J.; Dowell, A.C.; Grimwood, K.; the Whiti Te Rā Study Group

Background: In high-income countries with a temperate climate, acute respiratory infections (ARI) are a major source of morbidity, resulting in excess winter hospitalisations and up to 50% of GP consultations in preschool-aged children (Craig, E., *et al. Med. J. Aust.* 1992, *157*, S26–S28). 25-hydroxy vitamin D_3_ (25(OH)D_3_) modulates innate immunity and recent evidence highlights associations between vitamin D deficiency and several respiratory illnesses. Previously we reported that low levels of cord blood 25(OH)D were associated with increased risk of ARIs and childhood wheeze (Camargo, C.A., Jr., et al*. Pediatrics* 2011, *127*, e180–e187). However, two prior case-control studies provided conflicting evidence, with one reporting an 11-fold increased risk (Wayse, V.,et al*. Eur. J. Clin. Nutr.* 2004, *58*, 563–567), whilst the other found no association (Roth, D.E., et al. *Eur. J. Clin. Nutr.* 2009, *63*, 297-299). To help explain these divergent results we hypothesised that low levels of 25(OH)D3 are associated with increased severity of ARI in early childhood.

Methods: An unmatched case-control study of children aged <2-years in Wellington, New Zealand (NZ, latitude 41oS) was conducted over three successive winter seasons. Cases (severe) were defined as children with ARI requiring hospitalisation to one of two paediatric units for their ARI. Disease controls (mild) were defined as having a current ARI by doctor diagnosis on presentation to one of five general practice clinics, and were without a history of prior hospitalisation for ARI. 25(OH)D_3_ was collected from each child by capillary venous sampling, and stored as dried blood spots before shipment to ZRT laboratories (Beaverton, OR, USA). Disks punched from the dried blood spots were reconstituted before LC-MS/MS analysis (Newman, M.S., *et al. J. Diabetes Sci. Technol*. 2009, *3*, 156–162). Multivariable logistic regression was performed using SAS v9.3. The Multicentre Health and Disability Ethics Committee approved the study; ref: MEC/11/01/008.

Results: In the first 2-years of the study 25(OH)D_3_ results were obtained for 316 participants: 150 cases and 166 disease controls. Median levels (interquartile ranges) were 45.5(20.6–64.3) nmol/L among cases and 50.0(30.4–63.1) nmol/L among controls. Only 40 children (13%) had levels ≥75 nmol/L. Overall, 77 (24%) children were deficient (defined as 25(OH)D_3_ levels <25 nmol/L), with 47 (33%) cases and 30 (18%) controls. Cases had significantly higher odds of deficiency than controls (OR = 1.93; 95% CI: 1.12–3.30, *p* = 0.02). After adjusting for age at admission, gender, ethnicity (European *vs.* Non-European), breastfeeding, and NZiDep (a categorical measure of individual deprivation), the odds of deficiency among cases did not materially change (OR = 1.84; 95% CI: 1.02–3.33, *p* = 0.04).

Conclusions: Levels of 25OHD_3_ were <75 nmol/L in the vast majority of severe ARI cases and mild ARI disease controls. However, cases had significantly higher prevalence of vitamin D deficiency (defined as 25OHD_3_ < 25 nmol/L). After multivariable modelling this association remained significant. These interim results suggest an independent role for vitamin D in determining ARI severity amongst NZ children.

### 2.151. The Effect of Vitamin D Deficiency on the Developing Fetus: A Pilot Study

#### Kyriakopoulou, V.; Vatansever, D.; Dodhia, S.K.; Allsop, J.M.; Fox, M.J.; Paramasivam, G.; Hajnal, J.V.; Rutherford, M.A.

Background: Vitamin D deficiency during pregnancy is a common yet under-recognised problem affecting >50% of Caucasian and >80% of Black/African-American and South Asian populations. Prenatal vitamin D deficiency in a rat pup model has been associated with ventriculomegaly and brain overgrowth and abnormal behaviour while a few studies have demonstrated an association between prenatal vitamin D deficiency and lower birthweight. To date, there is no study investigating the effect of maternal vitamin D deficiency on the human fetus. The aim of the study was to measure maternal Vitamin D levels in stored booking samples of healthy volunteers and correlate with total ventricular and supratentorial brain volumes and birthweight.

Methods: The study was approved by the West London Research Ethics Committee (07/H0707/105). Fetal brain MRI was performed in 40 healthy volunteers (mean 27.4 weeks; 21.3–37 weeks). Volumetric analysis of the lateral ventricles and supratentorial brain tissue was performed on 3D-reconstructed datasets. Birthweight was recorded and centiles were calculated using customised birthweight charts incorporating gestational age at birth, sex and maternal ethnicity. Vitamin D levels were measured retrospectively in stored booking serum samples using liquid chromatography-mass spectrometry.

Results: Vitamin D levels were below normal levels (<70 nmol/L) in 70% of women and were significantly lower in women of South Asian origin (*p* = 0.004). Vitamin D levels significantly and positively correlated with birthweight centiles (*r* = 0.331, *p* = 0.042) and left ventricular volume (*r* = 0.403, *p* = 0.003) in the entire cohort and with supratentorial brain volume (*r* = 0.543, *p* = 0.024) in the deficient population only.

Conclusions: While this is a small pilot study, our results suggest a positive association between maternal Vitamin D levels and birthweight, left ventricular size and supratentorial brain volume.

### 2.152. Trial of Vitamin D Supplementation for the Prevention of Exacerbations of COPD and Upper Respiratory Infections

#### James, W.Y.; Islam, K.; Jolliffe, D.A.; Hooper, R.L.; Timms, P.M.; Bhowmik, A.; Choudhury, A.; Simcock, D.; Barnes, N.; Corrigan, C.; Hawrylowicz, C.M.; Griffiths, C.J.; Martineau, A.R.

Background: Exacerbations of COPD are precipitated by viral upper respiratory infections (URI). Vitamin D metabolites support anti-viral activity *in vitro*, and a single-centre trial in patients with predominantly severe COPD has reported that vitamin D supplementation reduced exacerbation risk in patients with baseline vitamin D deficiency. Multi-centre trials of vitamin D supplementation for prevention of exacerbation and URI in patients with less severe COPD are lacking.

Methods: We conducted a multi-centre double-blind randomised placebo-controlled trial of vitamin D supplementation in adults with GOLD Stage I-IV COPD in London, UK. 240 patients were allocated to receive the intervention (vitamin D_3_ 3 mg 2-monthly per os) or control (placebo 2-monthly) over one year. Co-primary outcomes were time to first moderate/severe exacerbation (*i.e.*, one meeting the Anthonisen symptom criteria and requiring treatment with antibiotics and/or oral corticosteroids) and time to first URI (determined by a validated acute respiratory symptom score). Symptom severity and mean serum 25(OH)D concentration were secondary outcomes. A pre-specified sub-group analysis was conducted to determine whether effects of the intervention on co-primary outcomes were modified by the presence or absence of vitamin D deficiency (serum 25(OH)D < 50 nmol/L) at baseline.

Results: 122 participants were allocated to the intervention arm of the trial, and 118 to the control arm. Mean baseline serum 25(OH)D concentration was 46.1 nmol/L, and 174/240 (73%) participants had GOLD Stage I/II COPD. Vitamin D supplementation was effective in elevating serum 25(OH)D concentration in the intervention *vs.* control arm (67.3 *vs.* 46.8 nmol/L respectively at one year, *p* < 0.001), but it did not influence time to first moderate/severe exacerbation (adjusted Hazard Ratio [aHR] 0.86, 95% CI 0.60 to 1.24, *p* = 0.42) or time to first URI (aHR 0.95, 95% CI 0.69 to 1.31, *p* = 0.75) in the study population as a whole. Mean peak symptom severity score for exacerbations was lower among participants allocated to intervention vs control arms (5.47 *vs.* 5.94 respectively, *p* = 0.04). Sub-group analysis revealed that vitamin D supplementation was protective against moderate/severe exacerbation among the 148 participants who were vitamin D deficient at baseline (aHR 0.57, 95% CI 0.35 to 0.92, *p* = 0.02), but not among the 92 participants with baseline serum 25(OH)D ≥ 50 nmol/L (aHR 1.45, 95% CI 0.81 to 2.62, *p* = 0.21; *p* for interaction = 0.02).

Conclusions: In the study population as a whole, intermittent bolus-dose vitamin D supplementation did not influence time to exacerbation or time to URI, but it was associated with a modest reduction in severity of exacerbation symptoms. Pre-specified sub-group analysis revealed a protective effect of vitamin D supplementation against moderate/severe exacerbations among participants who were vitamin D deficient at baseline.

### 2.153. Does Vitamin D as a Risk Reduction Factor for Cardiovascular Disease Satisfy Hill’s Criteria for Causality?

#### Weyland, P.G.; Grant, W.B.; Howie-Esquivel, J.

Background: There is mounting evidence that higher levels of serum 25-hydroxy vitamin D [25(OH)D] are associated with reduced risk of cardiovascular disease (CVD) and its various components including congestive heart failure, coronary heart disease, peripheral arterial disease, and stroke. This evidence leads to the question: Is higher serum 25(OH)D level causally linked to lower risk of CVD?

Methods: This study is based on a review of the journal literature evaluated using the framework of the criteria for causality in a biological system.

Results: The relevant Hill’s criteria and the findings are given here. Strength of association. Observational studies of CVD incidence or mortality rates with respect to serum 25(OH)D levels prior to CVD events find strong inverse relations for 25(OH)D level below 75 nmol/L. Consistency. Such findings have been found in many different populations.Temporality. Such studies are prospective and determine serum 25(OH)D levels at the time of enrollment. Biological gradient. When combined into a meta-analysis, there is a relative risk of 2.2 at 19 nmol/L, 1.6 at 30 nmol/L, 1.4 at 40 nmol/L, 1.2 at 50 nmol/L, and 1.04 at 70 nmol/L. The 95% confidence intervals are near ±0.2. Plausibility (e.g., mechanisms). There are many mechanisms whereby vitamin D can reduce the risk of CVD including reduced risk of diabetes mellitus and metabolic syndrome, effects on blood pressure, cholesterol, cytokines, endothelial disruption, glucose regulation, insulin resistance, muscle strength, respiratory infections, and vascular calcification. Experiment (e.g., randomized controlled trials (RCTs)). Since the risk of CVD is highest for low serum 25(OH)D levels, RCTs on populations with average serum 25(OH)D levels (around 50–60 nmol/L) generally have not found beneficial effects. However, RCTs in Iran, where the mean serum 25(OH)D levels tend to be low, in part due to sun avoidance during the hot summers, have found beneficial effects of vitamin D supplementation on CVD risk factors. Analogy. The serum 25(OH)D level-CVD risk relation is similar to those for type 2 diabetes mellitus and breast and colorectal cancer. Vitamin D is considered to satisfy Hill’s criteria for reduced risk of breast and colorectal cancer. Confounding factors. This consideration was added after Hill’s paper. Confounding factors have generally been included in the various studies. However, some of the confounding factors have not been fully resolved. One is the seasonal variation in CVD risk, which parallels seasonal variations in serum 25(OH)D levels. Evidently low temperature also affects risk of CVD, as does very high temperature.

Conclusion: Vitamin D as a risk reduction factor for CVD generally satisfies Hill’s criteria for causality in a biological system.

### 2.154. The Role of Geographical Ecological Studies in Determining the Role of Vitamin D in Disease Risk and Health Outcomes

#### Grant, W.B.

Background: Since the publication of the ultraviolet B (UVB)-vitamin D-cancer hypothesis based on an ecological study of colon cancer mortality rates with respect to annual solar radiation doses in the United States in 1980, the approach has been extended to many types of cancer and various other health outcomes. However, not all health outcomes related to serum 25-hydroxy vitamin D [25(OH)D] levels have geographical variations related to geographical variations in solar UVB doses. However, some vitamin D-sensitive diseases have seasonal variations with highest rates in winter or spring. The question addressed in this study is: Which diseases have geographical variations in outcome related to solar UVB, which have seasonal variations, and why the difference?

Methods: The peer-reviewed literature was searched for diseases or health outcomes correlated with solar UVB doses and/or outdoor occupation and which have seasonal variations.

Results: The diseases related to geographical variations in solar UVB doses or outdoor occupation include anaphylaxis, autism, 15–20 types of cancer, Crohn’s disease, type 1 diabetes mellitus, multiple sclerosis, Parkinson’s disease, sarcoidosis, and sepsis. Those diseases or outcomes related to season include adverse pregnancy and birth outcomes, cardiovascular disease, Epstein-Barr virus diseases, falls and fractures, influenza and all-cause mortality rate. However, cold temperature is also a risk factor for seasonal diseases and outcome, making it difficult to separate the effects of solar UVB/vitamin D from those of temperature. One reason for diseases falling in geographical or seasonal variation categories is the length of time it takes for the disease to develop. Some diseases, such as cancer, develop slowly so summertime solar UVB doses can raise serum 25(OH)D levels to where they can effectively combat the disease each year. Other diseases, such as influenza, myocardial infarction, and stroke, can develop rapidly when both long-term and short-term factors align.

Conclusion: Geographical ecological studies have provided strong support for the role of solar UVB doses in reducing the risk of several types of disease. When additional evidence on effects related to vitamin D from observational studies with respect to serum 25(OH)D level or personal UVB irradiance, the role of skin pigmentation, and laboratory studies of mechanisms, Hill’s criteria for causality in a biological system (Hill AB. The environment and disease: Association or causation? Proc R Soc Med. 1965; 58: 295–300) can be used to assess whether vitamin D is casually linked to reduced risk of the disease or adverse health outcome. While a few vitamin D randomized controlled trials have supported the ecological and observational studies, many were not designed and/or conducted properly. When they are, they, too, will add to the understanding of the role of vitamin D in reducing the risk of many types of disease and adverse health outcomes.

### 2.155. Prevalence of Vitamin D Deficiency and Its Related Aspects: An Observational Study of Vitamin D Status in Pregnant Women and Newborns in China

#### Liao, X.P.; Xiao, J.P.; Zang, J.; Fei, X.; Zhu, Y.; Pei, J.J.

Background: Thereare little data about the status of vitamin D in pregnant women and their newborns in China, and we evaluated the vitamin D status in pregnant women and their newborns all the year around in Wuxi city (North latitude 32°), Jiangsu province, China.

Methods: In 3278 pregnant women of childbearing age in the second trimester, the concentrations of serum 25-hydroxy vitamin D (25OHD) were measured, and its relationships with season, age and air temperature were analyzed. Meanwhile, the concentrations of serum 25OHD in 32 maternal infant pairs were measured at birth in the winter.

Results: (1) The mean 25OHD of pregnant women is 38.0 ± 15.8 nmol/L (range: 10.1~108.7 nmol/L). Vitamin D deficiency (defined as serum 25OHD < 30 nmol/L) accounted for 40.1% of all pregnant women. Vitamin D inadequate (serum 25OHD of 30~50 nmol/L) accounted for 35.9% of all pregnant women, and only 24.0% of pregnant women were vitamin D sufficient. Moreover, in the winter, most (58.7%) of pregnant women were the deficiency of vitamin D, and only 7.5% of the pregnant women were adequate to vitamin D. Of the 32 newborns, all were deficient in vitamin D. (2) Maternal vitamin D levels among different seasons were significant different (χ^2^ = 326.15, *p* < 0.001), and the lowest level of vitamin D was in the winter, and the highest was in the summer. (3) Among different age groups of pregnant women, vitamin D levels had significant difference (χ^2^ = 11.82, *p* = 0.003). The vitamin D level in 30~35 age group of pregnant women (median: 36.2 nmol/L, range: 13.8~108.7 nmol/L) was higher than that in the 25~30 age group (median: 35.9 nmol/L, range: 10.4~89.8 nmol/L) and the 18~25 age group (median: 33.3 nmol/L, range: 10.1~85.2 nmol/L). (4) The vitamin D status of pregnant women showed the same trend as the air temperature fluctuations, but its phase lagged behind about two months, reaching its highest value in September. Meanwhile, the vitamin D level was correlated with air temperature and its partial correlation coefficient was 0.306 (*n* = 3278, *p* < 0.001). (5) There was significant positive correlation between the concentration of 25OHD in cord blood and that in maternal blood (*n* = 32, *r* = 0.682, *p* < 0.001).

Conclusions: Vitamin D deficiency is prevalent in pregnant women and their newborns, especially in winter and in young pregnant woman. And the vitamin D status in pregnant women is significantly influenced by air temperature which is related to the exposure of ultraviolet rays.

### 2.156. Relationships between Serum 25-Hydroxy Vitamin D and Quantitative Ultrasound Bone Mineral Density in 0–6 Years Old Children

#### Yu, X.; Zhang, J.; Yan, C.; Shen, X.

Background: The relationship between serum 25-hydroxy vitamin D and quantitative ultrasound bone mineral density in young children remains unclear. In addition, consensus has not been reached with regard to the concentration of 25(OH)D to define vitamin D deficiency for infants and children.

Methods: In the present study, 203 children 0–6 years old were recruited in Shanghai, China. The concentrations of serum 25(OH)D, weight, length, and quantitative ultrasound bone mineral density (BMD) of left mid-tibia were determined. Low BMD was defined as <20th percentile of given age and sex. Low 25(OH)D was defined as 25(OH)D < 20 ng/mL.

Results: The results showed that median serum 25(OH)D level was 19.0 ng/mL, and 58.6% had a serum 25(OH)D below 20 ng/mL. After adjusting for potential confounders, a linear relationship between serum 25(OH)D and BMD was observed. Serum 25(OH)D was positively associated with BMD (β = 323.3, 95% CI = 201.0–445.7, *p* < 0.001), and low 25(OH)D (<20 ng/mL) had a high risk for low BMD (OR = 5.5, 95% CI = 2.5–12). In addition, there is a nonlinear relationship between 25(OH)D and low BMD, and a threshold 25(OH)D of 20 ng/mL existed for low BMD. The prevalence of low BMD was 47.1% in the group of 25(OH)D < 20 ng/mL, much higher than 16.7% in the group of 25(OH)D ≥ 20 ng/mL (*p* < 0.05).

Conclusion: The results suggested that quantitative ultrasound BMD could be an indicator for vitamin D status in young children, and also provided further evidence to define vitamin D deficiency for infants and children.

### 2.157. Identifications of Novel Epigenetic Mechanisms of Vitamin D in African Americans: Cross-Sectional and Interventional Approaches

#### Zhu, H.; Bhagatwala, J.; Parikh, S.; Huang, Y.; Su, S.; Snieder, H.; Shi, H.; Wang, X.; Dong, Y.

Background: Peripheral leukocytes, which express vitamin D receptor (VDR) and are capable of locally yielding active vitamin D, play an important role in inflammatory responses. Our objectives are (1) to conduct unbiased genome-wide methylation profiling of vitamin D in human peripheral leukocytes; (2) to comprehensively search differential functional methylation sites underlying vitamin D deficiency; (3) to test the hypothesis that vitamin D supplementation increases global DNA methylation level by performing a randomized clinical trial (RCT) in overweight/obese African Americans with vitamin D deficiency.

Methods: We first performed a genome-wide methylation scan using the Illumina Human Methylation 27 Bead-Chip on leukocyte DNA of 11 young African American males with severe vitamin D deficiency (25(OH)D ≤ 10 ng/mL) and 11 matched controls (25(OH)D ≥ 30 ng/mL). Then, we conducted a 16-weeks randomized, placebo controlled clinical trial of vitamin D supplementation (NCT01583621). A total of 65 young overweight and obese African Americans with vitamin D deficiency (25(OH)D) ≤20 ng/mL were randomly assigned to receive a supervised monthly oral vitamin D_3_ dose of placebo (*n* = 16), 18,000 IU (~600 IU/day, the current RDA; *n* = 15), 60,000 IU (~2000 IU/day; *n* = 17), or 120,000 IU (~4000 IU/day, the deemed tolerable upper intake level; *n* = 17). Global DNA methylation level (percentage of 5-methylcytosine, %5-mC) was quantified using leukocyte DNA with the MethylFlash Methylated DNA Quantification kit (Epigentek).

Results: The genome-wide methylation scan revealed that African Americans with severe vitamin D deficiency had reduced levels of methylation compared with controls. A total of 79 differential CpG sites achieved raw *p* < 0.001. Of the 79 CpG sites, 2 CpG sites survived multiple testing: cg16317961 (raw *p* = 3.5 × 10^−6^, FDR = 0.078, in MAPRE2) and cg04623955 (raw *p* = 5.9 × 10^−6^, FDR = 0.078, in DIO3). Furthermore, 3 out of the 4 genes previously identified in the 2 Genome-Wide Association Studies were also significant at the methylation level (DHCR7: cg07487535, *p* = 0.015 and cg10763288, *p* = 0.017; CYP2R1: cg25454890, *p* = 0.040; and CYP24A1: cg18956481, *p* = 0.022), reflecting significant enrichment (*p* = 0.0098). In the RCT, a significant increase was observed in the changes of serum 25(OH)D from baseline to 16 weeks (0.9 ± 2.0, placebo; 8.6 ± 2.1, 600 IU/day; 20.1 ± 2.0, 2000 IU/day; and 21.2 ± 2.0 ng/mL, 4,000 IU/day; *p* < 0.01). Meanwhile, a significant dose-responsive increase was observed in the changes of %5-mC from baseline to 16 weeks (0.00 ± 0.18, placebo; 0.02 ± 0.20, 600 IU/day; 0.29 ± 0.17, 2000 IU/day; and 0.66% ± 0.18%, 4000 IU/day group; *p* < 0.01). Moreover, the changes in %5-mC were significantly correlated with the changes in serum 25(OH)D (*r* = 0.60, *p* = 0.02) in the 4000 IU group.

Conclusion: Severe vitamin D deficiency is associated with methylation changes in leukocyte DNA in young African Americans, which may be involved in systemic inflammation. The genomic and epigenomic approaches reinforce the crucial roles played by the DHCR7, CYP2R1, and CYP24A1 genes. Vitamin D supplementation increases global DNA methylation level in a dose-responsive manner. Increased DNA methylation in peripheral leukocytes may be one underlying mechanism by which vitamin D exerts its anti-inflammatory effects. Vitamin D can activate or silence gene expressions to attenuate inflammation related to peripheral leukocytes.

## 3. Affiliations

- Abbas, M.A., Faculty of Medicine, Suez Canal University, Suez 41522, Egypt
- Aboud, S., Department of Microbiology and Immunology, Muhimbili University of Health and Allied Sciences, Dar es Salaam, Tanzania
- Abu el Maaty, M.A., Biochemistry Department, Faculty of Pharmacy and Biotechnology, German University in Cairo, Egypt
- Abu Zaid, L., Faculty of Medicine, King Fahad Medical City, King Saud Bin Abdulaziz University for Health Sciences, Riyadh 11525, Saudi Arabia
- Adams, F., Vascular and Inflammatory Diseases Research Unit, University of Dundee, Ninewells Hospital, Dundee DD1 9SY
- Adamson, A.J., Human Nutrition Research Centre, Newcastle University, Newcastle NE1 7RU, UK
- Ahmed, B., Senior Instructor Research and Consultant Epidemiologist, Department of Medicine, Aga Khan University, Karachi 3500
- Aihie Sayer, A., MRC Lifecourse Epidemiology Unit, University of Southampton, Southampton, SO16 6YD, UK
- Akiyama, N., Department of Pediatrics, Fuji Chuo Hospital, Shizuoka, Japan
- Al-Attas, O.S., Biomarkers Research Program, Biochemistry Department, College of Science, King Saud University, Riyadh 11451, Kingdom of Saudi Arabia, KSA
- Al-Daghri, N.M., Biomarkers Research Program, Department of Biochemistry, College of Science, King Saud University, Riyadh, 11451 Saudi Arabia
- Al-Ghamdi, A.S., Specialized Diabetes and Endocrine Center, King Fahad Medical City, King Saud Bin Abdulaziz University for Health Sciences, Riyadh 11525, Saudi Arabia
- Alghamdi, M., Specialized Diabetes and Endocrine Center, King Fahad Medical City, King Saud Bin Abdulaziz University for Health Sciences, Riyadh 11525, Saudi Arabia
- Al-Harbi, L., Division of Nutritional Sciences, University of Nottingham, School of Biosciences, Loughborough, Leics. LE12 5RD, UK
- Alissa, E.M., Faculty of Medicine, King AbdulAziz University, Jeddah, Saudi Arabia
- Aljohani, N., Specialized Diabetes and Endocrine Center, King Fahad Medical City, King Saud Bin Abdulaziz University for Health Sciences, Riyadh 11525, Saudi Arabia
- Alkabba, A.F., Specialized Diabetes and Endocrine Center, King Fahad Medical City, King Saud Bin Abdulaziz University for Health Sciences, Riyadh 11525, Saudi Arabia
- Alkharfy, K.M., Biomarkers Research Program, Department of Biochemistry, College of Science, King Saud University, Riyadh, 11451 Saudi Arabia
- Allsop, J.M., Centre for the Developing Brain, Division of Imaging Sciences and Biomedical Engineering, King’s College London, St Thomas’ Hospital, London, SE1 7EH, UK
- Almaas, B., The Hormone Laboratory, Haukeland University Hospital and Institute of Medicine, University of Bergen, Norway
- Almalki, M., Specialized Diabetes and Endocrine Center, King Fahad Medical City, King Saud Bin Abdulaziz University for Health Sciences, Riyadh 11525, Saudi Arabia
- Almås, B., Hormone Laboratory, Haukeland University Hospital, 5021 Bergen, Norway
- Alokail, M.S., Biomarkers Research Program, Biochemistry Department, College of Science, King Saud University, Riyadh 11451, Saudi Arabia
- Al-Othman, A., Prince Mutaib Chair for Biomarkers of Osteoporosis, Biochemistry Department, King Saud University, Riyadh 11451, Saudi Arabia
- Alothman, H.O., Specialized Diabetes and Endocrine Center, King Fahad Medical City, King Saud Bin Abdulaziz University for Health Sciences, Riyadh 11525, Saudi Arabia
- Al-Saleh, Y., Prince Mutaib Chair for Biomarkers of Osteoporosis, Biochemistry Department, King Saud University, Riyadh 11451, Saudi Arabia
- Alshahrani, F. College of Medicine, King Abdulaziz Medical City, Riyadh 14611, Saudi Arabia
- Alston-Mills, B.P., North Carolina State University, Raleigh, NC. USA Department of Animal Sciences
- Alyahya, K.O., Science Department, College of Basic Education, Public Authority for Applied Education and Training, Kuwait
- Andersen, E.W., Department of Applied Mathematics and Computer Science, Technical Univercity of Denmark, DK-2800 Lyngby, Denmark
- Andersen, R., National Food Institutte, Technical Univercity of Denmark, DK-2860 Søborg, Denmark
- Anderson, P., School of Pharmacy and Medical Sciences, Division of Health Sciences, University of South Australia, Adelaide, SA, 5000 Australia
- Andersson, Å., Gottsunda primary health care center, Box 25024, S-750 25 Uppsala, Sweden
- Andorsdóttir, G., Genetic Biobank of the Faroes, Ministry of Health, Tórshavn 100, Faroe Islands
- Aquilina, J., Department of Obstetrics and Gynaecology, The Royal London Hospital, Barts Health NHS Trust, London E1 1BB, UK
- Armstrong, B.K., School of Public Health, University of Sydney
- Arora, H., Institute of Home Economics, Delhi University, Delhi, India
- Assar, S., MRC Human Nutrition Research, Elsie Widdowson Laboratory, Fulbourn Rd, Cambridge, CB1 9NL, UK
- Attwood, K., Roswell Park Cancer Institute, Elm and Carlton Streets, Buffalo, New York 14263, USA
- Ayoub, D., Clinical Radiologists SC, Springfield, Illinois, USA
- Bacon, C.J., Department of General Practice and Primary Health Care, University of Auckland, Private Bag 92019, Auckland 1142, New Zealand.
- Baglietto, L., Centre for Epidemiology and Biostatistics, Melbourne School of Population and Global Health, The University of Melbourne, Melbourne, Victoria 3010, Australia
- Baird, J., MRC Lifecourse Epidemiology Unit, University of Southampton, University Hospital Southampton NHS Foundation Trust, Southampton SO16 6YD, UK
- Balayah, Z., Queen Mary, University of London, London UK
- Banga, R., King’s College London, UK
- Barnes, N., Queen Mary, University of London, London UK
- Barracchini, A., Rheumatology Department, San Camillo—Forlanini Hospital, Roma, Italy
- Barton, S.J., MRC Lifecourse Epidemiology Unit, University of Southampton, Southampton SO16 6YD, UK
- Basatemur, E., General & Adolescent Paediatrics Unit, UCL Institute of Child Health, London WC1N 1EH
- Baxter, C., Population Health Department, QIMR Berghofer Medical Research Institute
- Beck, K.L., Nutrition and Dietetics Division, College of Health, Massey University, Auckland
- Belch, J.J.F., Vascular and Inflammatory Diseases Research Unit, University of Dundee, Ninewells Hospital, Dundee DD1 9SY
- Bell, J.V., Regional Centre for Endocrinology and Diabetes, Royal Victoria Hospital, Belfast, BT12 6BA.UK
- Ben-Israel, J., Chronic Mechanical Ventilation Department B. Medical Center- Shoham, Pardes-Hanna. Affiliated with Rappaport Faculty of Medicine, The Technion, Haifa, Israel
- Bennett, S.E., Nutrition & Metabolism Group, Centre for Public Health, Queen’s University Belfast, Belfast, BT12 6BJ, UK
- Berentzen, T.L., Institute of Preventive Medicine, Bispebjerg and Frederiksberg Hospital, The Capital Region, Copenhagen, Denmark
- Berg, J.D., Department of Clinical Biochemistry, Sandwell and West Birmingham Hospitals NHS Trust, Birmingham B18 7QH, UK
- Berger, K., University of Muenster, Institute of Epidemiology and Social Medicine, Muenster, Germany
- Berry, J.L., Department of Clinical Biochemistry, Central Manchester NHS Foundation Trust, Manchester M13 9WL, UK
- Bevan, M.A., King’s College London, UK
- Beveridge, L.A., Division of Cardiovascular and Diabetic Medicine, University of Dundee, UK
- Bhagatwala, J., Georgia Prevention Center, Institute of Public and Preventive Health, Georgia Regents University, Georgia 30912, USA
- Bhat, M., Department of Endocrinology & Metabolism, National Institute of Nutrition, Hyderabad, India
- Bhowmik, A., Homerton University Hospital, London UK
- Billing, G., MRC Human Nutrition Research, Elsie Widdowson Laboratory, Fulbourn Road, Cambridge, CB1 9NL, UK
- Bishop, N.J., Academic Unit of Child Health, Department of Human Metabolism, University of Sheffield, UK
- Björk, A., Family Medicine and Preventive Medicine, Department of Public Health and Caring Sciences, Uppsala university, BMC Box 564, S-751 22 Uppsala, Sweden
- Blake, L., Department of Clinical Biochemistry, Galway Roscommon University Hospitals, Ireland
- Bland, R., Warwick Medical School, The University of Warwick, Coventry, CV4 7AL, UK
- Blum, L., Department of Anesthesia, Critical Care and Pain Medicine, Massachusetts General Hospital, Boston, MA 02114, USA
- Bogner, P.N., Roswell Park Cancer Institute, Elm and Carlton Streets, Buffalo, New York 14263, USA
- Borte, M., Children’s Hospital, Municipal Hospital “St. Georg”, Leipzig, Germany
- Borzutzky, A., Division of Pediatrics, School of Medicine, Pontificia Universidad Católica de Chile, Santiago, Chile
- Boucher, B.J., Centre for Diabetes, Queen Mary University of London, London E1 2AT, UK
- Bouillon, R., Experimental Medicine and Endocrinology, KU Leuven, Belgium
- Boyle, R.J., Department of Paediatrics, Imperial College London, UK
- Brameld, J.M., Division of Nutritional Sciences, University of Nottingham, School of Biosciences, Loughborough, Leics. LE12 5RD, UK
- Brekhoff, L., Department of paediatrics, Ziekenhuisgroep Twente, Hengelo, The Netherlands
- Bremner, S., Asthma UK Centre for Applied Asthma Research, Centre for Primary Care and Public Health, Blizard Institute, Queen Mary University of London, London E1 2AB, UK
- Brenner, H., Division of Clinical Epidemiology and Aging Research, German Cancer Research Center, Heidelberg, Germany
- Brüske, I., Institute of Epidemiology I, Helmholtz Zentrum München- German Research Center for Environmental Health, Neuherberg, Germany
- Bucca, G., Department of Microbial and Cellular Sciences, School of Biosciences and Medicine, Faculty of Health and Medical Sciences, University of Surrey, Guildford, Surrey GU2 7XH, UK
- Buhary, B.M., Specialized Diabetes and Endocrine Center, King Fahad Medical City, King Saud Bin Abdulaziz University for Health Sciences, Riyadh 11525, Saudi Arabia
- Burghaus, I., University of Bochum, Department of Medical Informatics, Biometry and Epidemiology, Bochum, Germany
- Burild, A., Division of Food Chemistry, National Food Institute, Technical University of Denmark, Soeborg, DK
- Busch, M.A., Department of Epidemiology and Health Monitoring, Robert Koch Institute, 12101 Berlin, Germany
- Byberg, L., Department of Surgical Sciences, Uppsala University, Sweden
- Camargo, C.A. Jr., Department of Emergency Medicine, and Division of Rheumatology, Allergy, and Immunology, Department of Medicine, Massachusetts General Hospital, Harvard Medical School, Boston, USA
- Cantwell, M.M., Cancer Epidemiology & Health Services Research Group, Centre for Public Health, Queen’s University Belfast, Northern Ireland
- Carlisi, A., Clinical Chemistry, University Hospital of Liège, Liège, Belgium
- Carroll, A., Children’s University Hospital, Dublin, Ireland
- Carroll, D., Cancer Epidemiology & Health Services Research Group, Centre for Public Health, Queen’s University Belfast, Northern Ireland
- Carson, E.L., Northern Ireland Centre for Food and Health (NICHE), University of Ulster, Coleraine, BT52 1SA
- Casey, C., Nutrition & Metabolism Group, Centre for Public Health, Queen’s University Belfast, Belfast, BT12 6BJ, UK
- Cashman, K.D., School of Food and Nutritional Sciences, University College Cork, Cork, Ireland
- Cavadino, A., University College London, UK
- Cavalier, E., Department of Clinical Chemistry, University of Liège, CHU Sart-Tilman, Liège Belgium
- Cerda, J., Department of Public Health, School of Medicine, Pontificia Universidad Católica de Chile, Santiago, Chile
- Chaloner, C., Department of Clinical Biochemistry, Central Manchester NHS Foundation Trust, Manchester M13 9WL, UK
- Chambers, S.T., Department of Pathology, University of Otago, Christchurch, New Zealand
- Chandra, N., Indian Institute of Science, Bangalore, India
- Chaplin, G., Department of Geography, The Pennsylvania State University, PA 16802, USA
- Cheetham, T.D., Institute of Human Genetics, Newcastle University, International Centre for Life, Newcastle upon Tyne NE1 3BZ, UK
- Chen, M., Melbourne Sexual Health Centre, Carlton, Victoria 3053, Australia
- Chope, G., Campden BRI, Chipping Campden, Gloucestershire GL55 6LD, UK
- Choudhury, A., Queen’s Hospital, Romford, UK
- Chowdhury, T.A., Blizard Institute, Barts and the London School of Medicine and Dentistry, Queen Mary University of London E1 2AB, UK
- Chrousos, G.P., Biomarkers Research Program, Biochemistry Department, College of Science, King Saud University, Riyadh 11451, Kingdom of Saudi Arabia, KSA
- Chugh, R. Institute of Home Economics, Delhi University, Delhi, India
- Cifuentes, L., Division of Pediatrics, School of Medicine, Pontificia Universidad Católica de Chile, Santiago, Chile
- Clark, D.A., Barts Health NHS Trust, London UK
- Cleal, J.K., Institute of Developmental Sciences, University of Southampton, Southampton SO16 6YD, UK
- Coad, J., Institute of Food Nutrition and Human Health, Massey University, Palmerston North, New Zealand
- Cole, Z.A., MRC Lifecourse Epidemiology Unit, University of Southampton, University Hospital Southampton NHS Foundation Trust, Southampton SO16 6YD, UK
- Coleman, H.G., Cancer Epidemiology & Health Services Research Group, Centre for Public Health, Queen’s University Belfast, Northern Ireland
- Collerton, J., Institute for Ageing and Health, Newcastle University, Newcastle NE4 5PL, UK
- Conlon, C., Nutrition and Dietetics Division, College of Health, Massey University, Auckland
- Cooper, C., MRC Lifecourse Epidemiology Unit, University of Southampton, University Hospital Southampton NHS Foundation Trust, Southampton SO16 6YD, UK
- Cooper, P., MRC Lifecourse Epidemiology Unit, University of Southampton, University Hospital Southampton NHS Foundation Trust, Southampton SO16 6YD, UK
- Corrigan, C., King’s College London, London UK
- Coussens, A.K., Clinical Infectious Diseases Research Initiative, University of Cape Town, Observatory 7925, South Africa
- Cox, G., Department of Biochemistry, St. James’s Hospital, Dublin 8, Ireland
- Crane, J., Medicine, University of Otago, Wellington 6021, NZ
- Cristi, F., Division of Pediatrics, School of Medicine, Pontificia Universidad Católica de Chile, Santiago, Chile
- Cross, L., Asthma UK Centre for Applied Asthma Research, Centre for Primary Care and Public Health, Blizard Institute, Queen Mary University of London, London E1 2AB, UK
- Cutolo, M., Research Laboratories and Academic Division of Clinical Rheumatology, Department of Internal Medicine, University of Genova, Genova, Italy
- Czeizel, E., Foundation for the Community Control of Hereditary Diseases, Budapest, Hungary
- Da Silva, S., Clinical Department, Laboratoires SMB SA, Brussels Belgium
- Damas, P., Burn Centre and General Intensive Care Department, University Hospital of Liège, Liège, Belgium
- Darling, A.L., Department of Nutritional Sciences, Faculty of Health and Medical Sciences, University of Surrey, Guildford GU2 7XH, UK
- Davies, J.H., Paediatric Endocrinology, University Hospital Southampton NHS Foundation Trust, Southampton, SO16 6YD, UK
- Davies, K., Institute for Ageing and Health, Newcastle University, Newcastle NE4 5PL, UK
- Davies, L., MRC Lifecourse Epidemiology Unit, University of Southampton, UK
- de Jongh, R.T., Department of Internal Medicine and Endocrinology, VU University Medical Center, Amsterdam, The Netherlands
- de Lusignan, S., Department of Health Care Management and Policy, Faculty of Business, Economics and Law, University of Surrey, Guildford, Surrey GU2 7XH, UK
- De Niet, S., Clinical Department, Laboratoires SMB SA, Brussels Belgium
- Decuman, S., Department of Rheumatology, Ghent University Hospital, Ghent, Belgium
- del Pistoia, M., Pediatrics Department, University of Pisa, Pisa, Italy
- den Heijer, M., Department of Internal Medicine and Endocrinology, VU University Medical Center, Amsterdam, The Netherlands
- Dennison, E.M., MRC Lifecourse Epidemiology Unit, University of Southampton, University Hospital Southampton NHS Foundation Trust, Southampton SO16 6YD, UK
- Detrick, B., Johns Hopkins University, Pathology, Baltimore, MD, USA
- Dieffenbach, A.K., Division of Clinical Epidemiology and Aging Research, German Cancer Research Center (DKFZ), Heidelberg, Germany
- Diehm, C., SRH-Klinikum Karlsbad-Langensteinbach, Department of Internal Medicine/Vascular Medicine, Karlsbad, Germany
- Diez, U., Children’s Hospital, Municipal Hospital “St. Georg”, Leipzig, Germany.
- Dodhia, S.K., Centre for the Developing Brain, Division of Imaging Sciences and Biomedical Engineering, King’s College London, St Thomas’ Hospital, London, SE1 7EH, UK
- Dong, L., North Carolina State University, Raleigh, NC. USA Department of Food, Nutrition & Bioprocessing Sciences
- Dong, Y., Georgia Prevention Center, Institute of Public and Preventive Health, Georgia Regents University, Georgia 30912, USA
- Dowell, A.C., Department of Primary Health Care & General Practice, University of Otago Wellington, PO Box 7343 Wellington South, Wellington 6242, New Zealand
- Duffy, J.C., School of Health and Population Sciences, University of Birmingham, Birmingham, B15 2TT, UK
- Duggan, C., Department of Nutrition, Harvard School of Public Health, Boston, MA, USA
- Dundas, I., Blizard Institute, Barts and the London School of Medicine and Dentistry, Queen Mary University of London, London E1 2AT, UK
- Dyall, L., Department of General Practice and Primary Health Care, University of Auckland, Private Bag 92019, Auckland 1142, New Zealand
- Ebeling, P.R., Department of Medicine, NorthWest Academic Centre, The University of Melbourne and Western Health, St Albans, Victoria 3021, Australia
- Ebstrup, J., Research Centre for Prevention and Health, Glostrup Hospital, University of Copenhagen, Glostrup, Denmark
- Ekeroma, A., Obstetrics & Gynaecology, University of Auckland, Auckland 1142, NZ
- Ekwaru, P.J., Population Health Intervention Research Unit, School of Public Health, University of Alberta, Edmonton AB Canada
- Eldridge, S., Queen Mary, University of London, London UK
- Elgizouli, M., Center for Chronic Immunodeficiency, University Medical Center Freiburg, Freiburg, Germany
- El-Kholie, E., Biomarkers Research Program, Biochemistry Department, College of Science, King Saud University, Riyadh 11451, Kingdom of Saudi Arabia, KSA
- Emanuelsson, I., Department of Pharmaceutical Biosciences, Uppsala University, Uppsala biomedicinska centrum BMC, Husarg. 3, Box 591, 751 24 UPPSALA, Sweden
- Emaus, N., Department of Health and Care Sciences, The Arctic University of Norway, Tromsø, Norway
- Embleton, N.D., Newcastle Neonatal Service, Newcastle Hospitals NHS Foundation Trust, Newcastle upon Tyne, UK
- Emery, S., The Kirby Institute, University of New South Wales, Sydney, Australia
- English, D.R., Centre for Epidemiology and Biostatistics, Melbourne School of Population and Global Health, The University of Melbourne, Melbourne, Victoria 3010, Australia
- Engmann, J., Department of Epidemiology and Public Health, University College London, WC1E 6BT, UK
- Ennis, C.N., Regional Centre for Endocrinology and Diabetes, Royal Victoria Hospital, Belfast, BT12 6BA, UK
- Erba, P., Nuclear Medicine, University of Pisa, Pisa, Italy
- Eriksson, J., University of Helsinki, Department of General Practice and Primary Health Care, 00014 University of Helsinki, Finland
- Eyles, D.W., Queensland Brain Institute, The University of Queensland, Brisbane, Queensland 4072, Australia
- Fairley, C.K., Melbourne Sexual Health Centre, Carlton, Victoria 3053, Australia
- Fanos, M., Pediatrics Department, University of Pisa, Pisa, Italy
- Fawzi, W.W., Department of Global Health and Population, Harvard School of Public Health, Boston, MA, USA
- Fei, X., Wuxi Maternity and Child Health Care Hospital, Nanjing Medical University, Wuxi, 214002, China
- Fernell, E., Gillberg Neuropsychiatry Centre, University of Gothenburg, Gothenburg SE-411 19, Sweden
- Ferns, G., Division of Medical Education, University of Brighton, Brighton, UK
- Figenschau, Y., Tromsø Endocrine Research Group, Department of Clinical Medicine, University of Tromsø, 9037 Tromsø, Norway
- Filteau, S., London School of Hygiene and tropical Medicine. UK
- Fletcher, S., Department of Nephrology, University Hospitals of Coventry and Warwickshire NHS Trust, CV2 2DX, UK
- Florkowski, C.M., Department of Pathology, University of Otago, Christchurch, New Zealand
- Flynn, A., School of Food and Nutritional Sciences, University College Cork, Cork, Ireland
- Flynn, M., Food Safety Authority of Ireland, Dublin, Ireland
- Ford, L., Department of Clinical Biochemistry, Sandwell and West Birmingham Hospitals NHS Trust, Birmingham B18 7QH, UK
- Forouhi, N.G., MRC Epidemiology Unit, University of Cambridge School of Clinical Medicine, Box 285 Institute of Metabolic Science, Cambridge Biomedical Campus, Cambridge, CB2 0QQ, UK
- Foskett, A., School of Sport and Exercise, Massey University, Auckland
- Foster, T., Department of Paediatrics, Ealing Hospital, LONDON UB1 3HW, UK
- Fouda, M., Medicine Department, King Khalid University Hospital, Riyadh 11451, Saudi Arabia
- Fox, M.J., Centre for the Developing Brain, Division of Imaging Sciences and Biomedical Engineering, King’s College London, St Thomas’ Hospital, London, SE1 7EH, UK
- Francis, R.M., Institute for Ageing and Health, Newcastle University, Newcastle NE4 5PL, UK
- Frandsen, H.L., Division of Food Chemistry, National Food Institute, Technical University of Denmark, Soeborg, DK
- Fraser, W.D., Norwich Medical School, University of East Anglia, Norwich NR4 7TJ, UK
- Frazier, A.L., Brigham and Women’s Hospital, Boston, MA 02115, USA
- Fuskevåg, O.M., Department of Clinical Pharmacology, University of North Norway, Tromsø, Norway
- Gad, M.Z., Biochemistry Department, Faculty of Pharmacy and Biotechnology, German University in Cairo, Egypt
- Gadisseur, R., Clinical Chemistry, University Hospital of Liège, Liège, Belgium
- Galvin, K., School of Food and Nutritional Sciences, University College Cork, Cork, Ireland
- Gamborg, M., Institute of Preventive Medicine, Bispebjerg and Frederiksberg Hospital, The Capital Region, Copenhagen, Denmark
- Gandhi, B., Centre for Primary Care and Public Health, Barts and The London School of Medicine, Queen Mary University of London, London E1 2AB, UK
- Gandy, S.J., Dept of Medical Physics, NHS Tayside, Ninewells Hospital, Dundee DD1 9SY
- Garratt, E., Southampton Institute of Developmental Sciences, University of Southampton, Southampton, UK
- Gebski, V., NHMRC Clinical Trials Centre, Sydney Medical School, University of Sydney
- Geissler, S., Institute for Agricultural and Nutritional Sciences, Martin-Luther-University Halle-Wittenberg, Germany
- Gibney, M.J., UCD Institute of Food and Health, University College Dublin, Dublin, Ireland
- Giguere, P., Department of Anesthesia, Critical Care and Pain Medicine, Massachusetts General Hospital, Boston, MA 02114, USA
- Giles, G.G., Centre for Epidemiology and Biostatistics, Melbourne School of Population and Global Health, The University of Melbourne, Melbourne, Victoria 3010, Australia
- Gill, P.S., Primary Care Clinical Sciences, University of Birmingham, Birmingham, B15 2TT, UK
- Gillberg, C., Institute of Health and Wellbeing, University of Glasgow, Glasgow G3 8SJ, UK
- Gluckman, P.D., Liggins Institute, University of Auckland, New Zealand
- Godfrey, K.M., MRC Lifecourse Epidemiology Unit, University of Southampton, University Hospital Southampton NHS Foundation Trust, Southampton SO16 6YD, UK
- Goldberg, G.R., MRC Human Nutrition Research, Elsie Widdowson Laboratory, Fulbourn Road, Cambridge, CB1 9NL, UK
- Goldring, S.T., Department of Paediatrics, Imperial College London, UK
- Goliath, R., Clinical Infectious Diseases Research Initiative, University of Cape Town, Observatory 7925, South Africa
- González, G., Department of Endocrinology, School of Medicine, Pontificia Universidad Católica de Chile, Santiago, Chile
- Gori, M., Pediatrics Department, University of Pisa, Pisa, Italy
- Granic, A., Institute for Ageing and Health, Newcastle University, Newcastle NE4 5PL, UK
- Grant, C.C., Paediatrics: Child & Youth Health, University of Auckland, Auckland 1142, NZ
- Grant, W.B., Sunlight, Nutrition and Health Research Center, San Francisco, CA 94164-1603, USA
- Gray, S.R., Musculoskeletal Research Programme, University of Aberdeen, Aberdeen, UK
- Greenwald, S., Blizard Institute, Barts and the London School of Medicine and Dentistry, Queen Mary University of London E1 2AB, UK
- Greiller, C.L., Centre for Primary Care and Public Health, Blizard Institute, Queen Mary University of London, E1 2AB, UK
- Grieve, A., Environmental Research Group, MRC-PHE Centre for Environmental Health, King’s College London, London SE1 9NH, UK
- Griffin, B., Department of Nutritional Sciences, University of Surrey, Guildford GU2 7XH
- Griffin, S.J., MRC Epidemiology Unit, University of Cambridge School of Clinical Medicine, Box 285 Institute of Metabolic Science, Cambridge Biomedical Campus, Cambridge, CB2 0QQ, UK
- Griffiths, C.J., Asthma UK Centre for Applied Asthma Research, Centre for Primary Care and Public Health, Blizard Institute, Queen Mary University of London, London E1 2AB, UK
- Grigg, J., Blizard Institute, Barts and the London School of Medicine and Dentistry, Queen Mary University of London, London E1 2AT, UK
- Grigg, J., Centre for Paediatrics, Blizard Institute, Queen Mary University of London, E1 2AB, UK
- Grimnes, G.,Tromsø Endocrine Research Group, Department of Clinical Medicine, The Arctic University of Norway, Tromsø, Norway
- Grimwood, K., Griffith Health, Gold Coast Campus, Griffith University and the Gold Coast University Hospital, Southport, Queensland 4222, Australia
- Gupta, A., Johns Hopkins University, Medicine, Baltimore, MD, USA
- Habib. A., Pathology, Aga Khan University, Karachi 3500
- Hajnal, J.V., Centre for the Developing Brain, Division of Imaging Sciences and Biomedical Engineering, King’s College London, St Thomas’ Hospital, London, SE1 7EH, UK
- Hakim, O.A., Department of Nutritional Sciences, Faculty of Health and Medical Sciences, University of Surrey, Guildford GU2 7XH, UK
- Hamill, L.L., Nutrition and Metabolism Group, Centre for Public Health, Queen’s University Belfast, Institute of Clinical Science Block B, Belfast, BT12 6BJ.UK
- Hanafi, R.S., Pharmaceutical Chemistry Department, Faculty of Pharmacy and Biotechnology, German University in Cairo, Egypt
- Hanson, M.A., Southampton Institute of Developmental Sciences, University of Southampton, Southampton, UK
- Hart, K.H., Department of Nutritional Sciences, School of Biosciences and Medicine, Faculty of Health and Medical Sciences, University of Surrey, Guildford, Surrey GU2 7XH, UK
- Harvey, N.C., MRC Lifecourse Epidemiology Unit, University of Southampton, University Hospital Southampton NHS Foundation Trust, Southampton SO16 6YD, UK
- Hashimoto, M., Department of Neurology, Katsushika Medical Center, Jikei University School of Medicine
- Haskelberg, H., The Kirby Institute, University of New South Wales, Sydney, Australi
- Hassanein, S.I., Biochemistry Department, Faculty of Pharmacy and Biotechnology, German University in Cairo, Egypt
- Hawrylowicz, C.M., King’s College London, London UK
- Hayden, K.E., Department of Clinical Biochemistry, Directorate of Laboratory Medicine, Central Manchester University Hospitals NHS Foundation Trust, Manchester Royal Infirmary, Manchester, M13 9WL, UK
- Hayes, A., School of Food and Nutritional Sciences, University College Cork, Cork, Ireland
- Hayman, K.J., Department of General Practice and Primary Health Care, University of Auckland, Private Bag 92019, Auckland 1142, New Zealand
- Healy, M., Department of Biochemistry, St. James’s Hospital, Dublin 8, Ireland
- Heath, A.K., Centre for Epidemiology and Biostatistics, Melbourne School of Population and Global Health, The University of Melbourne, Melbourne, Victoria 3010, Australia
- Heinrich, J., Institute of Epidemiology I, Helmholtz Zentrum München- German Research Center for Environmental Health, Neuherberg, Germany
- Heitmann, B.L., Institute of Preventive Medicine, Bispebjerg and Frederiksberg Hospital, Nordre Fasanvej 57, 2000 Frederiksberg, Denmark
- Herberth, G., Department of Environmental Immunology, Helmholtz Centre for Environmental Research—UFZ, Leipzig, Germany
- Hershberger, P.A., Roswell Park Cancer Institute, Elm and Carlton Streets, Buffalo, New York 14263. USA
- Heymans, M.W., Department of Epidemiology and Biostatistics, EMGO Institute for Health and Care Research, VU University Medical Center, Amsterdam, The Netherlands
- Hidetoshi, M., Department of Pediatrics, Jikei University School of Medicine, 18-19-3 Nishishinbashi, Minato-ku, Tokyo, JP
- Higgins, R., Department of Nephrology, University Hospitals of Coventry and Warwickshire NHS Trust, CV2 2DX, UK
- Hill, C., Population Health Department, QIMR Berghofer Medical Research Institute
- Hill, T.R., School of Agriculture, Food and Rural Development, Newcastle University, Newcastle NE1 7RU, UK
- Hiroshi, T., Department of Pediatrics, Jikei University School of Medicine, 18-19-3 Nishishinbashi, Minato-ku, Tokyo, JP
- Hitman, G.A., Blizard Institute, Barts and the London School of Medicine and Dentistry, Queen Mary University of London E1 2AB, UK
- Hodge, A.M., Cancer Epidemiology Centre, Cancer Council Victoria, Carlton, Victoria 3053, Australia
- Hodges, L., School of Sport and Exercise, Massey University, Palmerston North, New Zealand
- Hofbauer, L.C., Division of Endocrinology, Diabetes, and Bone Diseases, Dresden Technical University Medical Center, Dresden, Germany
- Holroyd, C., MRC Lifecourse Epidemiology Unit, University of Southampton, University Hospital Southampton NHS Foundation Trust, Southampton SO16 6YD, UK
- Hooper, R.L., Asthma UK Centre for Applied Research, Centre for Primary Care and Public Health, Barts and the London, School of Medicine and Dentistry, Queen Mary University of London, UK
- Hoti, M., Genome Centre, Barts and The London School of Medicine and Dentistry, John Vane Science Centre, Charterhouse Square, London EC1M 6BQ, UK
- Hourihane, J.O’B., Department of Paediatrics and Child Health, University College Cork, Ireland
- Houston, J.G., Dept of Clinical Imaging, University of Dundee, Ninewells Hospital, Dundee DD1 9SY
- Howie-Esquivel, J., University of California San Francisco, Department of Physiological Nursing, San Francisco, CA 94143, USA
- Hoyos-Bachiloglu, R., Division of Pediatrics, School of Medicine, Pontificia Universidad Católica de Chile, Santiago, Chile
- Huang, C., Department of Paediatrics, Ealing Hospital ICO, London UB1 3HW, UK
- Huang, Y., Georgia Prevention Center, Institute of Public and Preventive Health, Georgia Regents University, Georgia 30912, USA
- Hunter, S.J., Regional Centre for Endocrinology and Diabetes, Royal Victoria Hospital, Belfast, BT12 6BA.UK
- Husemoen, L.L., Research Centre for Prevention and Health, The Capital Region of Denmark, Glostrup University Hospital, Denmark
- Hutchinson, M.S., Tromsø Endocrine Research Group, Institute of Clinical Medicine, University of Tromsø, Norway
- Hyppönen, E., Centre for Paediatric Epidemiology and Biostatistics, Institute of Child Health, University College London, London WC1N 1EH, UK
- Hyppönen, E., School of Population Health and Sansom Institute, University of South Australia, Adelaide, Australia
- Ida, H., Department of Pediatrics, Jikei University School of Medicine, Tokyo, Japan
- Ingelsoon, E., Department of Medical Sciences, Uppsala University, Sweden
- Ingham, T.R., Department of Medicine, University of Otago Wellington, PO Box 7343 Wellington South, Wellington 6242, New Zealand
- Inskip, H.M., MRC Lifecourse Epidemiology Unit, University of Southampton, UK
- Iqbal, R., Department of Community Health Sciences, Aga Khan University, Karachi 3500
- Ireland, S.E., Dept of Clinical Pharmacology, University of Dundee, Ninewells Hospital, Dundee DD1 9SY
- Islam, K., Queen Mary, University of London, London UK
- Ismail, A., Department of Endocrinology & Metabolism, National Institute of Nutrition, Hyderabad, India
- Iturriaga, C., Division of Pediatrics, School of Medicine, Pontificia Universidad Católica de Chile, Santiago, Chile
- Ivison, F.M., Department of Clinical Biochemistry, Central Manchester NHS Foundation Trust, Manchester M13 9WL, UK
- Jablonski, N., Department of Anthropology, The Pennsylvania State University, PA 16802, USA
- Jacobsen, R., Institute of Preventive Medicine, Bispebjerg and Frederiksberg Hospital, Nordre Fasanvej 57, 2000 Frederiksberg, Denmark
- Jagger, C., Institute for Ageing and Health, Newcastle University, Newcastle NE4 5PL, UK
- Jakeman, P., Physical Education and Sport Sciences Department, University of Limerick, Ireland
- Jakobsen, J., Division of Food Chemistry, National Food Institute, Technical University of Denmark, Soeborg, DK
- Jamaludin, J., Environmental Research Group, MRC-PHE Centre for Environmental Health, King’s College London, London SE1 9NH, UK
- James, W.Y., Queen Mary, University of London, London UK
- Jamil, N.A., Musculoskeletal Research Programme, University of Aberdeen, Aberdeen, UK
- Jandrain, B., Department of Clinical Pharmacology, ATC SA, Liège Belgium
- Jarjou, L.M.A., MRC Keneba, The Gambia, West Africa
- Javaid, M.K., NIHR Musculoskeletal Biomedical Research Unit, University of Oxford, UK
- Jefferson, K., Department of Urology, University Hospitals of Coventry and Warwickshire NHS Trust, CV2 2DX, UK
- Jennings, L.C., Department of Pathology, University of Otago, Christchurch, New Zealand
- Jensen, C.B., Institute of Preventive Medicine, Bispebjerg and Frederiksberg Hospital, The Capital Region, Copenhagen, Denmark
- Jessup, W., Nutrition and Dietetics Division, College of Health, Massey University, Auckland
- Johansson, G., Family Medicine and Preventive Medicine, Department of Public Health and Caring Sciences, Uppsala university, BMC Box 564, S-751 22 Uppsala, Sweden
- Johnsen, M., Tromsø Endocrine Research Group, Department of Clinical Medicine, University of Tromsø, 9037 Tromsø, Norway
- Johnson, C.S., Roswell Park Cancer Institute, Elm and Carlton Streets, Buffalo, New York 14263. USA
- Johnson, G., Sighthill Health Centre, 380 Calder Road, Edinburgh EH11 4AU, Scotland, UK
- Johnson, N., Department of Clinical Science and Technology, UCL, UK
- Johnston, S.L., National Heart and Lung Institute, Imperial College London, W2 1PG, UK
- Jolliffe, D.A., Centre for Primary Care and Public Health, Barts and The London School of Medicine and Dentistry, Queen Mary University of London, London E1 2AB, UK
- Jones, B., Department of Medicine, University of Otago Wellington, PO Box 7343 Wellington South, Wellington 6242, New Zealand
- Jones, K.S., MRC Human Nutrition Research, Elsie Widdowson Laboratory, Fulbourn Rd, Cambridge, CB1 9NL, UK
- Jones, S.L., Clinical Biochemistry department, King George’s Hospital, Barking, Havering and Redbrige University Hospitals NHS Trust, Essex, IG3 8YB, UK
- Jorde, R., Division of Internal Medicine, University Hospital of North Norway, Tromsø, Norway
- Jorde, R., Tromsø Endocrine Research Group, Department of Clinical Medicine, The Arctic University of Norway, Tromsø, Norway
- Jorde, R., Tromsø Endocrine Research Group, Department of Clinical Medicine, University of Tromsø, 9037 Tromsø, Norway
- Jørgensen, T., Research Centre for Prevention and Health, Glostrup Hospital, University of Copenhagen, Glostrup, Denmark
- Jubulis, J., Maine Medical Center/Tufts University, Pediatrics, Portland, ME, USA
- Judkins, A.M., Newtown Union Health Service, 14 Hall Avenue, Newtown, Wellington 6021, New Zealand
- Junaid, K., Department of Microbiology and Molecular Genetics, University of the Punjab, Lahore, Pakistan.
- Junge, K.M., Department of Environmental Immunology, Helmholtz Centre for Environmental Research—UFZ, Leipzig, Germany
- Jungert, A., Institute of Nutritional Science, Justus-Liebig-University of Giessen, Giessen, Germany
- Kabir, G., Vascular and Inflammatory Diseases Research Unit, University of Dundee, Ninewells Hospital, Dundee DD1 9SY
- Kalsi, H., Blizard Institute, Barts and the London School of Medicine and Dentistry, Queen Mary University of London, London E1 2AT, UK
- Kamycheva, E., Tromsø Endocrine Research Group, Institute of Clinical Medicine, University of Tromsø, Norway
- Kashif. W., Associate Professor Nephrology, Department of Medicine, Aga Khan University, Karachi 3500
- Katarzyna, B.K., Department of Geriatrics, Medical University of Warsaw. Oczki 4, 02-007 Warsaw, Poland
- Kaur, M., Institute of Home Economics, Delhi University, Delhi, India
- Kaur, S., Paediatrics: Child & Youth Health, University of Auckland, Auckland 1142, NZ.
- Kaykov, E., Chronic Mechanical Ventilation Department B. Medical Center- Shoham, Pardes-Hanna. Affiliated with Rappaport Faculty of Medicine, The Technion, Haifa, Israel
- Kelly, F.J., Environmental Research Group, MRC-PHE Centre for Environmental Health, King’s College London, London SE1 9NH, UK
- Kennedy, G., Vascular and Inflammatory Diseases Research Unit, University of Dundee, Ninewells Hospital, Dundee DD1 9SY
- Kenny, L.C., The Irish Centre for Fetal and Neonatal Translational Research, Department of Obstetrics and Gynaecology, University College Cork, Ireland
- Kepa, M., Department of General Practice and Primary Health Care, University of Auckland, Private Bag 92019, Auckland 1142, New Zealand
- Kerse, N., Department of General Practice and Primary Health Care, University of Auckland, Private Bag 92019, Auckland 1142, New Zealand
- Khan, F., Division of Cardiovascular and Diabetic Medicine, University of Dundee, UK
- Khan, K.S., Centre for Primary Care and Public Health, Barts and The London School of Medicine, Queen Mary University of London, London E1 2AB, UK
- Kiely, M., School of Food and Nutritional Sciences, University College Cork, Cork, Ireland
- Kilander, L., Department of Public Health and Caring Sciences, Uppsala University, Sweden
- Kilbane, M.T., Metabolism Laboratory, St. Vincent’s University Hospital, Dublin, Ireland
- Kilpin, K., Queen Mary, University of London, London UK
- Kim, S., King’s College London, UK
- Kimball, S., Trials by Design, Toronto, ONT, Canada
- Kimlin, M.G., Institute of Health and Biomedical Innovation, Queensland University of Technology, Brisbane, Queensland 4059, Australia
- Kinsella, M., School of Food and Nutritional Sciences, University College Cork, Cork, Ireland
- Kirkby, J.C., Portex Respiratory Unit, University College London, Institute of Child Health, London, UK
- Kirkwood, T., Institute for Ageing and Health, Newcastle University, Newcastle NE4 5PL, UK
- Kirman, J., Department of Microbiology and Immunology, University of Otago, PO Box 56 Dunedin 9054, New Zealand
- Kisenge, R., Department of Paediatrics and Child Health, Muhimbili University of Health and Allied Sciences, Dar es Salaam, Tanzania
- Kivimaki, M., Department of Epidemiology and Public Health, University College London, WC1E 6BT, UK
- Kjærgaard, M., Division of Internal Medicine, University Hospital of North Norway, Tromsø, Norway
- Klassen, K.M., NorthWest Academic Centre, University of Melbourne, St Albans, Victoria 3021, Australia
- Knol, D.L., EMGO Institute for Health and Care Research, Department of Epidemiology and Biostatistics, VU University Medical Center, Amsterdam, NL
- Knop, J., Institute of Preventive Medicine, Bispebjerg and Frederiksberg Hospital, The Capital Region, Copenhagen, Denmark
- Kočovská, E., Institute of Health and Wellbeing, University of Glasgow, Glasgow G3 8SJ, UK
- Kratzsch, J., Institute of Laboratory Medicine, Clinical Chemistry and Molecular Diagnostics, University Hospital Leipzig, Leipzig, Germany
- Kruger, M.C., College of Health, Massey University, Palmerston North
- Kukhareva, P.V., North Carolina State University, Raleigh, NC. USA Department of Food, Nutrition & Bioprocessing Sciences
- Kumar, S., Clinical Sciences Research Institute, Diabetes and Metabolism Unit, Coventry CV47AL, UK
- Kumari, M., Department of Epidemiology and Public Health, University College London, WC1E 6BT, UK
- Kunzmann, A.T., Cancer Epidemiology & Health Services Research Group, Centre for Public Health, Queen’s University Belfast, Northern Ireland
- Kupisz-Urbańska, M., Department of Geriatrics, Medical University of Warsaw. Oczki 4, 02-007 Warsaw, Poland
- Kupka, R., Department of Nutrition, Harvard School of Public Health, Boston, MA, USA
- Kurpad, A.V., St. John’s Medical College, Bangalore, India; (4) Sitaram Bhartia Institute of Science and Research, Delhi, India
- Kvaskoff, D., Queensland Brain Institute, The University of Queensland, Brisbane, Queensland 4072, Australia
- Kyriakopoulou, V., Centre for the Developing Brain, Division of Imaging Sciences and Biomedical Engineering, King’s College London, St Thomas’ Hospital, London, SE1 7EH, UK
- Lachmann, R., King’s College London, UK
- Lahti, J., Institute of Behavioural Sciences, University of Helsinki, 00014 University of Helsinki, Finland
- Laird, E., Institute of Molecular Medicine, School of Medicine, Trinity College, Dublin
- Lambrechts, D., Laboratory for Translational Genetics, Vesalius Research Center, VIB, KU Leuven, Belgium
- Lanham-New, S.A., Department of Nutritional Sciences, School of Biosciences and Medicine, Faculty of Health and Medical Sciences, University of Surrey, Guildford, Surrey GU2 7XH, UK
- Layla, A., Environmental Research Group, MRC-PHE Centre for Environmental Health, King’s College London, London SE1 9NH, UK
- Le Roy, C., Division of Pediatrics, School of Medicine, Pontificia Universidad Católica de Chile, Santiago, Chile
- Ledoux, D., Burn Centre and General Intensive Care Department, University Hospital of Liège, Liège, Belgium
- Lehmann, I., Department of Environmental Immunology, Helmholtz Centre for Environmental Research—UFZ, Leipzig, Germany
- Lewis, R.M., Institute of Developmental Sciences, University of Southampton, Southampton SO16 6YD, UK
- Liao, X.P., Wuxi Maternity and Child Health Care Hospital, Nanjing Medical University, Wuxi, 214002, China
- Lillycrop, K.A., Southampton Institute of Developmental Sciences, University of Southampton, Southampton, UK
- Lind, L., Department of Medical Sciences, Uppsala University, Sweden
- Linneberg, A., Research Centre for Prevention and Health, Glostrup Hospital, University of Copenhagen, Glostrup, Denmark
- Lips, P., Department of Internal Medicine and Endocrinology, VU University Medical Center, Amsterdam, The Netherlands
- Liu, L., Department of Epidemiology and Biostatistics, School of Public Health, and College of Medicine, Drexel University, Philadelphia, PA, 19102, United States
- Llewellyn, D., University of Exeter Medical School, Exeter EX1 2LU, UK
- Lluch, A., Danone Research, Centre Daniel Carasso, Palaiseau, France
- Logan, C., Institute of Epidemiology and Medical Biometry, Ulm University, Ulm, Germany
- Lucas, R., National Centre for Epidemiology and Population Health, Australian National University
- uczyńska, A., Center for Chronic Immunodeficiency, University Medical Center Freiburg, Freiburg, Germany
- Lukas, P., Clinical Chemistry, University Hospital of Liège, Liège, Belgium
- MacDonald, D., Department of Urology, University Hospitals of Coventry and Warwickshire NHS Trust, CV2 2DX, UK
- Macdonald, H.M., Musculoskeletal Research Programme, University of Aberdeen, Aberdeen, UK
- MacDonald, T.M., Hypertension Research Centre and Medicines Monitoring Unit (MEMO), University of Dundee, Ninewells Hospital, Dundee DD1 9SY
- Mackenzie, I.S., Hypertension Research Centre and Medicines Monitoring Unit (MEMO), University of Dundee, Ninewells Hospital, Dundee DD1 9SY
- MacLaughlin, B., Queen Mary, University of London, London UK
- Maddock, J., Centre for Paediatric Epidemiology and Biostatistics, Institute of Child Health, University College London, London WC1N 1EH, UK
- Madhi, S.A., University of the Witwatersrand; MRC: Respiratory and Meningeal Pathogens Research Unit, Johannesburg, South Africa
- Madsen, K.H., National Food Institutte, Technical Univercity of Denmark, DK-2860 Søborg, Denmark
- Mahmud, N., Department of Medicine, Trinity Centre for Health Science, St. James’s Hospital, Dublin 8, Ireland
- Malone-Lee, J., Department of Clinical Science and Technology, UCL, UK
- Manaseki-Holland, S., School of Health and Population Sciences, University of Birmingham, Birmingham, B15 2TT, UK
- Manavi, K.R., North Carolina State University, Raleigh, NC. USA Department of Food, Nutrition & Bioprocessing Sciences
- Mandami, M., Whittington Health, UK
- Manderson, J.G., Regional Centre for Endocrinology and Diabetes, Royal Victoria Hospital, Belfast, BT12 6BJ, UK
- Manji, K.P., Department of Paediatrics and Child Health, Muhimbili University of Health and Allied Sciences, Dar es Salaam, Tanzania
- Mannan, N., Blizard Institute, Barts and the London School of Medicine and Dentistry, Queen Mary University of London E1 2AB, UK
- Marleen, S., Department of Obstetrics and Gynaecology, The Royal London Hospital, Barts Health NHS Trust, London E1 1BB, UK
- Marlin, N., Asthma UK Centre for Applied Asthma Research, Centre for Primary Care and Public Health, Blizard Institute, Queen Mary University of London, London E1 2AB, UK
- Martineau, A.R., Asthma UK Centre for Applied Asthma Research, Centre for Primary Care and Public Health, Blizard Institute, Queen Mary University of London, London E1 2AB, UK
- Martineau, A.R., Centre for Primary Care and Public Health, Barts and The London School of Medicine and Dentistry, Queen Mary University of London, London E1 2AB, UK
- Martin-Ruiz, C., Institute for Ageing and Health, Newcastle University, Newcastle NE4 5PL, UK
- Mathers, J.C., Human Nutrition Research Centre, Newcastle University, Newcastle NE1 7RU, UK
- Mazzilli, S.A., Boston University School of Medicine, 72 E. Concord Street, Boston, MA 02118. USA
- McAuliffe, F., School of Medicine and Medical Science, University College Dublin, Ireland
- McCance, D.R., Regional Centre for Endocrinology and Diabetes, Royal Victoria Hospital, Belfast, BT12 6BJ, UK
- McCarthy, C.M., Department of Anesthesia, Critical Care and Pain Medicine, Massachusetts General Hospital, Boston, MA 02114, USA
- McCarthy, E.K., Vitamin D Research Group, School of Food and Nutritional Sciences, University College Cork, Ireland
- McCarthy, R., National Maternity Hospital, Dublin, Ireland
- McCormack, W., Physical Education and Sport Sciences Department, University of Limerick, Ireland
- McDaid, M., Whittington Health, UK
- McEvoy, C.T., Nutrition and Metabolism Group, Centre for Public Health, Queen’s University Belfast, Institute of Clinical Science Block B, Belfast, BT12 6BJ.UK
- McGinty, A., Nutrition & Metabolism Group, Centre for Public Health, Queen’s University Belfast, Belfast, BT12 6BJ, UK
- McGowan, J., National Maternity Hospital, Dublin, Ireland
- McKenna, M.J., Metabolism Laboratory, St. Vincent’s University Hospital, Dublin, Ireland
- McKinley, M.C., Nutrition and Metabolism Group, Centre for Public Health, Queen’s University Belfast, Institute of Clinical Science Block B, Belfast, BT12 6BJ.UK
- McLean, C., Liggins Institute, University of Auckland, New Zealand; (3) AgResearch, Ruakura Research Centre, Hamilton, New Zealand
- McNamara, D., Department Of Medicine, Trinity Centre for Health Science, Tallaght Hospital, Dublin 8, Ireland
- McNulty, B.A., UCD Institute of Food and Health, University College Dublin, Dublin, Ireland
- McPeake, J., Nutrition & Metabolism Group, Centre for Public Health, Queen’s University Belfast, Belfast, BT12 6BJ, UK
- McSwiggan, S., Tayside Clinical Trials Unit, Ninewells Hospital, Dundee DD1 9SY
- Mehmood, A., Physiotherapy, Aga Khan University, Karachi 3500
- Mein, C., Genome Centre, Barts and The London School of Medicine and Dentistry, John Vane Science Centre, Charterhouse Square, London EC1M 6BQ, UK
- Mejborn, H., National Food Institutte, Technical Univercity of Denmark, DK-2860 Søborg, Denmark
- Melhus, H., Department of Medical Sciences, Uppsala University, Sweden
- Menon, R.K., Blizard Institute, Barts and the London School of Medicine and Dentistry, Queen Mary University of London E1 2AB, UK
- Mensink, G.B.M., Department of Epidemiology and Health Monitoring, Robert Koch Institute, 12101 Berlin, Germany
- Merlijn, T., Department of General Practice, VU University Medical Center, Amsterdam, The Netherlands
- Meves, S.H., St. Josef Hospital, Department of Neurology, Bochum, Germany
- Mezawa, H., Division of Molecular Epidemiology, Jikei University School of Medicine, Tokyo, Japan
- Michaëlsson, K., Department of Surgical Sciences, Uppsala University, Sweden
- Michie, C.A., Department of Paediatrics, Ealing Hospital ICO, London UB1 3HW, UK
- Middleton, B., Chronobiology, Department of Biochemistry and Physiology, Faculty of Health and Medical Sciences, Guildford GU2 7XH, UK
- Milburn, H., Guy’s and St Thomas’ Foundation Trust
- Milne, Paediatrics: Child & Youth Health, University of Auckland, Auckland 1142, NZ
- Minisola, G., Rheumatology Department, San Camillo—Forlanini Hospital, Roma, Italy
- Minnis, H., Institute of Health and Wellbeing, University of Glasgow, Glasgow G3 8SJ, UK
- Mitchell, C., University of Miami Miller School of Medicine, Miami, FL, USA
- Mitchell, E., Paediatrics: Child & Youth Health, University of Auckland, Auckland 1142, NZ
- Mitchell, S., Nutrition and Dietetics Division, College of Health, Massey University, Auckland
- Mitsuyoshi, U., Division of Molecular epidemiology, Jikei University School of Medicine, 8-25-3 Nishishinbashi, Minato-ku, Tokyo, JP
- Mohammed, G., Specialized Diabetes and Endocrine Center, King Fahad Medical City, King Saud Bin Abdulaziz University for Health Sciences, Riyadh 11525, Saudi Arabia
- Moharram, O., King Abdulaziz University Hospital, King Saud University, Riyadh 11451, Saudi Arabia
- Molloy, E., School of Medicine and Medical Science, University College Dublin, Ireland
- Montepiedra, G., Harvard School of Public Health, Boston, MA, USA
- Moon, R.J., MRC Lifecourse Epidemiology Unit, University of Southampton, University Hospital Southampton NHS Foundation Trust, Southampton SO16 6YD, UK
- Morales, P., Immunology, Allergy and Rheumatology Unit, Division of Pediatrics, School of Medicine, Pontificia Universidad Católica de Chile, Santiago, Chile
- Morrin, M., Metabolism Laboratory, St. Vincent’s University Hospital, Dublin, Ireland
- Moss, P.A.H., School of Cancer Sciences, University of Birmingham, Birmingham B15 2TT, UK
- Mossakowska, M., Internatinal Institute of Molecular and Cell Biology in Warsaw. Ksiecia Trojdena 4, 02-109 Warsaw, Poland
- Moyes, S., Department of General Practice and Primary Health Care, University of Auckland, Private Bag 92019, Auckland 1142, New Zealand
- Mudway, I.S., Environmental Research Group, MRC-PHE Centre for Environmental Health, King’s College London, London SE1 9NH, UK
- Mughal, Z.M., Department of Paediatric Endocrinology, Royal Manchester Childrens Hospital, Manchester M13 9WL, UK
- Mulhern, M.S., Northern Ireland Centre for Food and Health (NICHE), University of Ulster, Coleraine, BT52 1SA
- Murakami, M., Department of Neurology, Katsushika Medical Center, Jikei University School of Medicine
- Murdoch, D.R., Department of Pathology, University of Otago, Christchurch, New Zealand
- Murphy, N., Department of Endocrinology, St. Vincent’s University Hospital, Dublin, Ireland
- Murray, B., Metabolism Laboratory, St. Vincent’s University Hospital, Dublin, Ireland
- Murray, D.M., Department of Paediatrics and Child Health, University College Cork, Ireland
- Murray, L.J., Cancer Epidemiology & Health Services Research Group, Centre for Public Health, Queen’s University Belfast, Northern Ireland
- Murray, R., Southampton Institute of Developmental Sciences, University of Southampton, Southampton, UK
- Narayan, R.S., Genotypic Ltd., Bangalore, India
- Naude, C., Centre for Evidence-based Health Care, Stellenbosch University, Tygerberg 7505, South Africa
- Navarrete, C., Department of Dermatology, School of Medicine, Pontificia Universidad Católica de Chile, Santiago, Chile
- Neale, R.E., Population Health Department, QIMR Berghofer Medical Research Institute
- Neuhäuser-Berthold, M., Institute of Nutritional Science, Justus-Liebig-University of Giessen, Giessen, Germany
- Nexø, B.A., Department of Biomedicine, Aarhus University, DK-8000 Aarhus, Denmark
- Ng, K., Department of Medical Oncology, Dana-Farber Cancer Institute, Harvard Medical School, 450 Brookline Avenue, Boston, MA 02215, USA
- ni Chaoimh, C., Vitamin D Research Group, School of Food and Nutritional Sciences, University College Cork, Ireland
- Nieters, A., Center for Chronic Immunodeficiency, University Medical Center Freiburg, Freiburg, Germany
- Nigdikar, S., MRC Human Nutrition Research, Elsie Widdowson Laboratory, Fulbourn Road, Cambridge, CB1 9NL, UK
- Nissen, J., National Food Institutte, Technical Univercity of Denmark, DK-2860 Søborg, Denmark
- Norizoe, C., Division of Molecular Epidemiology, Jikei University School of Medicine, Tokyo, Japan
- Norlin, M., Department of Pharmaceutical Biosciences, Uppsala University, Sweden
- Norton, C., Physical Education and Sport Sciences Department, University of Limerick, Ireland
- Noya, M., Division of Molecular Epidemiology, Jikei University School of Medicine, Tokyo, Japan
- Ntani, G., MRC Lifecourse Epidemiology Unit, University of Southampton, University Hospital Southampton NHS Foundation Trust, Southampton SO16 6YD, UK
- O’Donovan, S.M., Vitamin D Research Group, School of Food and Nutritional Sciences, University College Cork, Ireland
- O’Keane, M., Metabolism Laboratory, St. Vincent’s University Hospital, Dublin, Ireland
- O’Morain, C., Department Of Medicine, Trinity Centre for Health Science, Tallaght Hospital, Dublin 8, Ireland
- O’Shea, P., Department of Clinical Biochemistry, Galway Roscommon University Hospitals, Ireland
- O’Sullivan, M., Department of Medicine, Trinity Centre for Health Science, St. James’s Hospital, Dublin 8, Ireland
- Olivier, L., Danone Nutricia Early Life Nutrition, Trowbridge, BA14 0XQ, UK
- Onwuneme, C., National Maternity Hospital, Dublin, Ireland
- Paolino, S., Research Laboratories and Academic Division of Clinical Rheumatology, Department of Internal Medicine, University of Genova, Genova, Italy
- Paramasivam, G., Centre for Fetal Care, Queen Charlotte’s & Chelsea Hospital, Imperial College Healthcare Trust, Du Cane road, London, W12 0HS, UK
- Parikh, S., Georgia Prevention Center, Institute of Public and Preventive Health, Georgia Regents University, Georgia 30912, USA
- Parker, S.A., Department of Nutrition and Dietetics, University Hospitals of Coventry and Warwickshire NHS Trust, Coventry, CV2 2DX, UK
- Parr, T., Division of Nutritional Sciences, University of Nottingham, School of Biosciences, Loughborough, Leics. LE12 5RD, UK
- Patel, M., Queen Mary, University of London, London UK
- Patel, N., King’s College London, UK
- Paterson, C.R., Formerly Department of Medicine, University of Dundee, Dundee, Scotland
- Payen, F., Groupe de Recherche Nutritionnelle, Yoplait, Boulogne 92641 Cedex, France
- Pean, J., Danone Research, Centre Daniel Carasso, Palaiseau, France
- Pearce, S., Institute of Genetic Medicine, Newcastle University, Newcastle NE2 4HH, UK.
- Pearce, S.H., Institute of Human Genetics, Newcastle University, International Centre for Life, Newcastle upon Tyne NE1 3BZ, UK
- Pei, J.J., Wuxi Maternity and Child Health Care Hospital, Nanjing Medical University, Wuxi, 214002, China
- Penn, N., King’s College London School of Medicine
- Penson, S., Campden BRI, Chipping Campden, Gloucestershire GL55 6LD, UK
- Perna, L., Division of Clinical Epidemiology and Aging Research, German Cancer Research Center (DKFZ), Heidelberg, Germany
- Petchey, M., Biochemistry Department, University Hospitals of Coventry and Warwickshire NHS Trust, Coventry, CV2 2DX, UK
- Peters, B., King’s College London, UK
- Petrovic, K.E., Division of General Neurology, Department of Neurology, General Hospital and Medical University of Graz ,8036, Austria
- Phinney, K.W., Biomolecular Measurement Division, National Institute of Standards and Technology, Gaithersburg, Maryland, USA
- Pilarski, A., King’s College London School of Medicine
- Pizzorni, C., Research Laboratories and Academic Division of Clinical Rheumatology, Department of Internal Medicine, University of Genova, Genova, Italy
- Poulton, S., Department of Paediatrics, Imperial College London, UK
- Pourshahidi, L.K., Northern Ireland Centre for Food and Health (NICHE), University of Ulster, Coleraine, BT52 1SA
- Power, C., Centre for Paediatric Epidemiology and Biostatistics, Institute of Child Health, University College London, London WC1N 1EH, UK
- Prentice, A., MRC Human Nutrition Research, Elsie Widdowson Laboratory, Fulbourn Road, Cambridge, CB1 9NL, UK
- Priest, P.C., Department of Preventive and Social Medicine, University of Otago, Dunedin, New Zealand
- Quilter, M.L., Institute of Food Nutrition and Human Health, Massey University, Palmerston North, New Zealand
- Quraishi, S.A., Department of Anesthesia, Critical Care and Pain Medicine, Massachusetts General Hospital, Boston, MA 02114, USA
- Rabenberg, M., Department of Epidemiology and Health Monitoring, Robert Koch Institute, 12101 Berlin, Germany
- Raftery, T., Department of Medicine, Trinity Centre for Health Science, St. James’s Hospital, Dublin 8, Ireland
- Räikkönen, K., Institute of Behavioural Sciences, University of Helsinki, 00014 University of Helsinki, Finland
- Rajakulasingam, R.K., Homerton University Hospital, London UK
- Rajput, M., Institute of Home Economics, Delhi University, Delhi, India
- Randhawa, J., Guy’s and St Thomas’ Foundation Trust
- Rasmussen, L.B., National Food Institutte, Technical Univercity of Denmark, DK-2860 Søborg, Denmark
- Rasvi, S., Institute of Genetic Medicine, Newcastle University, Newcastle NE2 4HH, UK
- Ravn-Haren, G., National Food Institutte, Technical Univercity of Denmark, DK-2860 Søborg, Denmark
- Raymond, K., Institute of Preventive Medicine, Bispebjerg and Frederiksberg Hospital, The Capital Region, Copenhagen, Denmark
- Rehman, A., Department of Microbiology and Molecular Genetics, University of the Punjab, Lahore, Pakistan.
- Rehman, A.M., MRC Tropical Epidemiology Group, LSHTM, Keppel St, London, WC1E 7HT, UK
- Rhein, H.M., Sighthill Health Centre, 380 Calder Road, Edinburgh EH11 4AU, Scotland, UK
- Rickard, A.P., MRC Epidemiology Unit, University of Cambridge School of Clinical Medicine, Box 285 Institute of Metabolic Science, Cambridge Biomedical Campus, Cambridge, CB2 0QQ, UK
- Rifas-Shiman, S.L., Harvard Medical School, Boston, MA 02115, USA
- Robertson, F., Department of Nutritional Sciences, University of Surrey, Guildford GU2 7XH
- Robinson, S., Department of Medicine, Imperial College London, UK
- Robinson, S.M., MRC Lifecourse Epidemiology Unit, University of Southampton, Southampton, SO16 6YD, UK
- Röder, S., Core Facility Studies, Helmholtz Centre for Environmental Research—UFZ, Leipzig, Germany
- Roth, H.J., Department of Endocrinology/Oncology, Limbach Laboratory, Heidelberg, Germany
- Rothenbacher, D., Institute of Epidemiology and Medical Biometry, Ulm University, Ulm, Germany
- Rousseau, A.F., Burn Centre and General Intensive Care Department, University Hospital of Liège, Liège, Belgium
- Rousseau, B., Groupe de Recherche Nutritionnelle, Yoplait, Boulogne 92641 Cedex, France
- Rowden, J., Paediatrics: Child & Youth Health, University of Auckland, Auckland 1142, NZ
- Rowe, M., Clinical Biochemistry Department, Homerton University Hospital NHS Foundation Trust, Homerton Row, London E9 6SR, UK
- Rutherford, M.A., Centre for the Developing Brain, Division of Imaging Sciences and Biomedical Engineering, King’s College London, St Thomas’ Hospital, London, SE1 7EH, UK
- Sabico, S., Biomarkers Research Program, Department of Biochemistry, College of Science, King Saud University, Riyadh, 11451 Saudi Arabia
- Sachdev, H.P.S., Sitaram Bhartia Institute of Science and Research, Delhi, India
- Saeed, T., Gulab Devi hospital, Lahore, Pakistan
- Saggese, G., Pediatrics Department, University of Pisa, Pisa, Italy
- Sanchez, A., Department of Epidemiology and Public Health, University College London, WC1E 6BT, UK
- Sandle, L.N., Department of Chemical Pathology, Central Manchester University Hospitals NHS Foundation Trust, Trafford General Hospital, Manchester, M41 5SL, UK
- Sarin, P., College of Medical and Dental Sciences, University of Birmingham, Birmingham, B15 2TT, UK
- Sarwar, S., Resident Nephrology, Aga Khan University, Karachi 3500
- Saum, K.U., Division of Clinical Epidemiology and Aging Research, German Cancer Research Center (DKFZ), Heidelberg, Germany
- Scheen, A., Division of Diabetes, Nutrition and Metabolic Disorders, CHU Sart Tilman, University of Liege, Liège, Belgium
- Scheidt-Nave, C., Department of Epidemiology and Health Monitoring, Robert Koch Institute, 12101 Berlin, Germany
- Schirmer, H., Department of Clinical Medicine, Faculty of Health Sciences, The Arctic University, Tromsø, Norway
- Schmidt, H., Research Unit for Genetic Epidemiology, Institute of Molecular Biology and Biochemistry, Center of Molecular Medicine, Medical University of Graz, 8010 Graz, Austria
- Schmidt, R., Division of General Neurology, Department of Neurology, General Hospital and Medical University of Graz ,8036, Austria
- Schoenmakers, I., MRC Human Nutrition Research, Elsie Widdowson Laboratory, Fulbourn Rd, Cambridge, CB1 9NL, UK
- Schöttker, B., Division of Clinical Epidemiology and Aging Research, German Cancer Research Center, Heidelberg, Germany
- Scragg, R., School of Population Health, University of Auckland, Auckland, New Zealand
- Seamans, K.M., School of Food and Nutritional Sciences, University College Cork, Cork, Ireland
- Segawa, T., Department of Pediatrics, Fuji Chuo Hospital, Shizuoka, Japan
- Sempos, C.T., Office of Dietary Supplements, National Institutes of Health, Bethesda, MD 20852, USA
- Sen, A., Research Unit for Genetic Epidemiology, Institute of Molecular Biology and Biochemistry, Center of Molecular Medicine, Medical University of Graz, 8010 Graz, Austria
- Seriolo, B., Research Laboratories and Academic Division of Clinical Rheumatology, Department of Internal Medicine, University of Genova, Genova, Italy
- Shaheen, S.O., Asthma UK Centre for Applied Research, Centre for Primary Care and Public Health, Barts and the London, School of Medicine and Dentistry, Queen Mary University of London, UK
- Sharp, S.J., MRC Epidemiology Unit, University of Cambridge School of Clinical Medicine, Box 285 Institute of Metabolic Science, Cambridge Biomedical Campus, Cambridge, CB2 0QQ, UK
- Shea, R.L., Department of Clinical Biochemistry, Sandwell and West Birmingham Hospitals NHS Trust, Birmingham B18 7QH, UK
- Sheikh, A., Allergy & Respiratory Research Group, Centre for Population Health Sciences, The University of Edinburgh, Edinburgh EH8 9AG, UK
- Shen, X., XinHua Hospital, Shanghai Jiao Tong University School of Medicine, Shanghai, China
- Sheppard, A., Liggins Institute, University of Auckland, New Zealand
- Shi, H., Cancer Research Center, Georgia Regents University, Georgia 30912, USA
- Silva, S., Department of Dermatology, School of Medicine, Pontificia Universidad Católica de Chile, Santiago, Chile
- Simcock, D., Barts Health NHS Trust, London UK
- Simner, C.L., Institute of Developmental Sciences, University of Southampton, Southampton SO16 6YD, UK
- Skaaby, T., Research Centre for Prevention and Health, Glostrup Hospital, University of Copenhagen, Glostrup, Denmark
- Skene, D.J., Chronobiology, Department of Biochemistry and Physiology, Faculty of Health and Medical Sciences, Guildford GU2 7XH, UK
- Slow, S., Department of Pathology, University of Otago, Christchurch, New Zealand
- Smith, C.P., Department of Microbial and Cellular Sciences, School of Biosciences and Medicine, Faculty of Health and Medical Sciences, University of Surrey, Guildford, Surrey GU2 7XH, UK
- Smith, S., Department Of Medicine, Trinity Centre for Health Science, Tallaght Hospital, Dublin 8, Ireland
- Smith, V., Department of Rheumatology, Ghent University Hospital, Ghent, Belgium
- Smithers, H., Asthma UK Centre for Applied Asthma Research, Centre for Primary Care and Public Health, Blizard Institute, Queen Mary University of London, London E1 2AB, UK
- Snieder, H., Department of Epidemiology, University Medical Center Groningen, University of Groningen, Groningen, The Netherlands
- Sohl, E., Department of Epidemiology and Biostatistics, EMGO Institute for Health and Care Research, VU University Medical Center, Amsterdam, The Netherlands
- Sollid, S.T., Tromsø Endocrine Research Group, Institute of Clinical Medicine, University of Tromsø, Norway
- Sørensen, T.I.A., Institute of Preventive Medicine, Bispebjerg and Frederiksberg Hospital, The Capital Region, Copenhagen, Denmark
- Spector, S.A., University of California, San Diego, La Jolla, CA and Rady Children’s Hospital San Diego, CA USA
- Stangl, G.I., Institute for Agricultural and Nutritional Sciences, Martin-Luther-University Halle-Wittenberg, Germany
- Steel, S.C., Imperial College NHS Trust, UK
- Stevens, N., Queen Mary, University of London, London UK
- Stewart, A.W., School of Population Health, University of Auckland, Auckland, New Zealand
- Stocks, J., Portex Respiratory Unit, University College London, Institute of Child Health, London, UK
- Strachan, A., Musculoskeletal Research, University of Aberdeen, AB25 2ZD
- Strain, J.J., Northern Ireland Centre for Food and Health (NICHE), University of Ulster, Coleraine, BT52 1SA
- Struthers, A.D., Division of Cardiovascular and Diabetic Medicine, University of Dundee, UK
- Su, S., Georgia Prevention Center, Institute of Public and Preventive Health, Georgia Regents University, Georgia 30912, USA
- Sudfeld, C.R., Department of Global Health and Population, Harvard School of Public Health, Boston, MA, USA
- Sulli, A., Research Laboratories and Academic Division of Clinical Rheumatology, Department of Internal Medicine, University of Genova, Genova, Italy
- Suri, R., Centre for Paediatrics, Blizard Institute, Queen Mary University of London, E1 2AB, UK
- Sutcliffe, A., General & Adolescent Paediatrics Unit, UCL Institute of Child Health, London WC1N 1EH
- Suzuki, M., Department of Neurology, Katsushika Medical Center, Jikei University School of Medicine
- Svartberg, J., Tromsø Endocrine Research Group, Institute of Clinical Medicine, University of Tromsø, Norway
- Svirsky, B., Chronic Mechanical Ventilation Department B. Medical Center- Shoham, Pardes-Hanna. Affiliated with Rappaport Faculty of Medicine, The Technion, Haifa, Israel
- Swallow, W.H., North Carolina State University, Raleigh, NC. USA Department of Statistics
- Syed Qadri, S.Y.H., Department of Pathology, National Institute of Nutrition, Hyderabad, India
- Syed, A., Queen Mary, University of London, London UK
- Tachimoto, H., Department of Pediatrics, Jikei University School of Medicine, Tokyo, Japan
- Tak, L., MRC Asthma UK Centre in Allergic Mechanisms of Asthma, King’s College London, London SE1 9RT, UK
- Takaaki, S., Department of Pediatrics, Fuji City General Hospital, 50 Takashimacho, Fuji-shi, Shizuoka, JP
- Takahashi, D., Division of Molecular Epidemiology, Jikei University School of Medicine
- Talesnik, E., Immunology, Allergy and Rheumatology Unit, Division of Pediatrics, School of Medicine, Pontificia Universidad Católica de Chile, Santiago, Chile
- Teh, R., Department of General Practice and Primary Health Care, University of Auckland, Private Bag 92019, Auckland 1142, New Zealand
- Thiem, U., University of Bochum, Department of Medical Informatics, Biometry and Epidemiology, Bochum, Germany
- Thiering, E., Institute of Epidemiology I, Helmholtz Zentrum München- German Research Center for Environmental Health, Neuherberg, Germany
- Thuesen, B., Research Centre for Prevention and Health, Glostrup Hospital, University of Copenhagen, Glostrup, Denmark
- Thuesen, B.H., Research Centre for Prevention and Health, The Capital Region of Denmark, Glostrup University Hospital, Denmark
- Thummel, K., University of Washington, Seattle, USA
- Timms, P., Clinical Biochemistry Department, Homerton University Hospital NHS Foundation Trust, Homerton Row, London E9 6SR, UK
- Tinati, T., MRC Lifecourse Epidemiology Unit, University of Southampton, University Hospital Southampton NHS Foundation Trust, Southampton SO16 6YD, UK
- Ting, S.M., Department of Nephrology, University Hospitals of Coventry and Warwickshire NHS Trust, CV2 2DX, UK
- Toher, C., National Maternity Hospital, Dublin, Ireland
- Torjesen, P.A., Hormone Laboratory, Department of Endocrinology, Oslo University Hospital, Aker, 0424 Oslo, Norway
- Trampisch, H.J., University of Bochum, Department of Medical Informatics, Biometry and Epidemiology, Bochum, Germany
- Tran, B., Centre for Health Research, University of Western Sydney
- Trenholme, A., Women & Children’s Health, Middlemore Hospital, Auckland 2025, NZ
- Triggiani, L., Neurological Rehabilitation Department, San Giovanni Battista Hospital, Roma, Italy
- Trilok, K.G. , Institute of Home Economics, Delhi University, Delhi, India
- Tripkovic, L., Department of Nutritional Sciences, School of Biosciences and Medicine, Faculty of Health and Medical Sciences, University of Surrey, Guildford, Surrey GU2 7XH, UK
- Trump, D.L., Roswell Park Cancer Institute, Elm and Carlton Streets, Buffalo, New York 14263. USA
- Urashima, M., Division of Molecular Epidemiology, Jikei University School of Medicine, Tokyo, Japan
- van den Heuvel, E.G.H.M., FrieslandCampina, Amersfoort, The Netherlands
- van der Gaag, E.J., Department of paediatrics, Ziekenhuisgroep Twente, Hengelo, The Netherlands
- van Schoor, N.M., Department of Epidemiology and Biostatistics, EMGO Institute for Health and Care Research, VU University Medical Center, Amsterdam, The Netherlands
- Vanderbist, F., Clinical Department, Laboratoires SMB SA, Brussels Belgium
- Vanderschueren, D., Experimental Medicine and Endocrinology, KU Leuven, Belgium
- Vatansever, D., Centre for the Developing Brain, Division of Imaging Sciences and Biomedical Engineering, King’s College London, St Thomas’ Hospital, London, SE1 7EH, UK
- Venn, A., Menzies Research Institute, Tasmania
- Venton, T., Clinical Biochemistry Department, Homerton University Hospital NHS Foundation Trust, Homerton Row, London E9 6SR, UK
- Vera, C., Department of Dermatology, School of Medicine, Pontificia Universidad Católica de Chile, Santiago, Chile
- Veugelers, P., Population Health Intervention Research Unit, School of Public Health, University of Alberta, Edmonton AB Canada
- Vierucci, F., Pediatrics Department, University of Pisa, Pisa, Italy
- Violari, A., PHRU, University of the Witwatersrand, Johannesburg, South Africa
- Visser, M., Department of Epidemiology and Biostatistics, EMGO Institute for Health and Care Research, VU University Medical Center, Amsterdam, The Netherlands
- Vogel, U., National Research Centre of the Working Enviroment, DK-2100 Copenhagen, Denmark
- Vogl, C., Roche Diagnostics, Penzberg, Germany
- von Hurst, P., Institute of Food Nutrition and Human Health, Massey University, Albany, New Zealand
- von Hurst, P.R., Nutrition and Dietetics Division, College of Health, Massey University, Auckland
- Vyakarnam, A., King’s College London, UK
- Wall, C., Nutrition, University of Auckland, Auckland 1142, NZ
- Wallace, I.R., Regional Centre for Endocrinology and Diabetes, Royal Victoria Hospital, Belfast, BT12 6BA, UK
- Walton, J., School of Food and Nutritional Sciences, University College Cork, Cork, Ireland
- Walton, R., Asthma UK Centre for Applied Asthma Research, Centre for Primary Care and Public Health, Blizard Institute, Queen Mary University of London, London E1 2AB, UK
- Wang, X., Georgia Prevention Center, Institute of Public and Preventive Health, Georgia Regents University, Georgia 30912, USA
- Wang, Z., University of Washington, Seattle, USA
- Warner, J.O., Department of Paediatrics, Imperial College London, UK
- Warren, J.M., Nutricia Research, Utrecht, The Netherlands
- Waterhouse, M., Population Health Department, QIMR Berghofer Medical Research Institute
- Waugh, S., Dept of Medical Physics, NHS Tayside, Ninewells Hospital, Dundee DD1 9SY
- Waymouth, E., Paediatrics: Child & Youth Health, University of Auckland, Auckland 1142, NZ.
- Webb, P.M., Population Health Department, QIMR Berghofer Medical Research Institute
- Webber, J., School of Cancer Sciences, University of Birmingham, Birmingham, B15 2TT, UK
- Wegler, C., Department of Pharmaceutical Biosciences, Uppsala University, Sweden
- Weir, R.R., Northern Ireland Centre for Food and Health (NICHE), University of Ulster, Coleraine, BT52 1SA
- Weyland, P.G., University of California San Francisco, Department of Physiological Nursing, San Francisco, CA 94143, USA
- Whiteman, D.C., Population Health Department, QIMR Berghofer Medical Research Institute
- Whiting, S.J., College of Pharmacy and Nutrition, University of Saskatchewan, Saskatoon SK S7N 5C9, Canada
- Wikvall, K., Department of Pharmaceutical Biosciences, Uppsala University, Sweden
- Wilkinson, R.J., Clinical Infectious Diseases Research Initiative, University of Cape Town, Observatory 7925, South Africa
- Williams, D., University College London, UK
- Williamson, E.J., entre for Epidemiology and Biostatistics, Melbourne School of Population and Global Health, The University of Melbourne, Melbourne, Victoria 3010, Australia
- Wilson, L., Department of Nutritional Sciences, School of Biosciences and Medicine, Faculty of Health and Medical Sciences, University of Surrey, Guildford, Surrey GU2 7XH, UK
- Witham, M.D., Ageing and Health, University of Dundee, Ninewells Hospital, Dundee DD1 9SY
- Witt, K.D., Queen Mary, University of London, London UK
- Wood, A.D., Musculoskeletal Research, University of Aberdeen, AB25 2ZD
- Wood, C.L., Child Health, Newcastle Hospitals NHS Foundation Trust, Newcastle upon Tyne, UK
- Wood, H.E., Environmental Research Group, MRC-PHE Centre for Environmental Health, King’s College London, London SE1 9NH, UK
- Woodside, P.M., Nutrition and Metabolism Group, Centre for Public Health, Queen’s University Belfast, Institute of Clinical Science Block B, Belfast, BT12 6BJ.UK
- Xiao, J.P., Wuxi Maternity and Child Health Care Hospital, Nanjing Medical University, Wuxi, 214002, China
- Yakout, S., Prince Mutaib Chair for Biomarkers of Osteoporosis, Biochemistry Department, King Saud University, Riyadh 11451, Saudi Arabia
- Yan, C., XinHua Hospital, Shanghai Jiao Tong University School of Medicine, Shanghai, China
- Yoshioka, M., Department of Neurology, Katsushika Medical Center, Jikei University School of Medicine
- Young, I.S., Nutrition and Metabolism Group, Centre for Public Health, Queen’s University Belfast, Institute of Clinical Science Block B, Belfast, BT12 6BJ.UK
- Young, M.C., Harvard Medical School, Boston, MA 02115
- Young, S.I., Department of Anesthesia, Critical Care and Pain Medicine, Massachusetts General Hospital, Boston, MA 02114, USA
- Yousef, M., Health Affairs for Riyadh Region, Ministry of Health, Riyadh 11175, Saudi Arabia
- Yu, C., Department of Medicine, Imperial College London, UK; (4) Portex Respiratory Unit, University College London, Institute of Child Health, London, UK
- Yu, X., XinHua Hospital, Shanghai Jiao Tong University School of Medicine, Shanghai, China
- Zang, J., Wuxi Maternity and Child Health Care Hospital, Nanjing Medical University, Wuxi, 214002, China
- Zehnder, D., Department of Nephrology, University Hospitals of Coventry and Warwickshire NHS Trust, CV2 2DX, UK
- Zeldow, B., Harvard School of Public Health, Boston, MA, USA
- Zhang, J., XinHua Hospital, Shanghai Jiao Tong University School of Medicine, Shanghai, China
- Zhang, Y., School of Food and Nutritional Sciences, University College Cork, Cork, Ireland
- Zhu, H., Georgia Prevention Center, Institute of Public and Preventive Health, Georgia Regents University, Georgia 30912, USA
- Zhu, Y., Wuxi Maternity and Child Health Care Hospital, Nanjing Medical University, Wuxi, 214002, China
- Zostautiene, I., Tromsø Endocrine Research Group, Department of Clinical Medicine, University of Tromsø, 9037, Tromsø, Norway
- Zwicker, J.D., School of Public Policy, University of Calgary, Calgary AB Canada

